# Effectiveness and Safety of Methods to Prevent Bloodstream and Other Infections and Noninfectious Complications Associated With Peripherally Inserted Central Catheters: A Systematic Review and Meta-Analysis

**DOI:** 10.1093/cid/ciaf063

**Published:** 2025-02-12

**Authors:** Andreea Dobrescu, Alexandru Marian Constantin, Larisa Pinte, Andrea Chapman, Piotr Ratajczak, Irma Klerings, Robert Emprechtinger, Benedetta Allegranzi, Michael Lindsay Grayson, Joao Paulo Toledo, Gerald Gartlehner, Barbara Nussbaumer-Streit

**Affiliations:** Cochrane Austria, Department for Evidence-based Medicine and Evaluation, University for Continuing Education Krems, Krems, Austria; Department of Internal Medicine Clinical Hospital Colentina, University of Medicine and Pharmacy Carol Davila, Bucharest, Romania; Department of Internal Medicine Clinical Hospital Colentina, University of Medicine and Pharmacy Carol Davila, Bucharest, Romania; Cochrane Austria, Department for Evidence-based Medicine and Evaluation, University for Continuing Education Krems, Krems, Austria; Department of Pharmacoeconomics and Social Pharmacy, Poznan University of Medical Sciences, Poznan, Poland; Cochrane Austria, Department for Evidence-based Medicine and Evaluation, University for Continuing Education Krems, Krems, Austria; Berlin Institute of Health at Charité (BIH), BIH QUEST Center for Responsible Research, Berlin, Germany; Infection Prevention and Control Unit, Department of Integrated Health Services, World Health Organization, Geneva, Switzerland; Infection Prevention and Control Unit, Department of Integrated Health Services, World Health Organization, Geneva, Switzerland; Department of Medicine, University of Melbourne, Melbourne, Australia; Infectious Diseases and Immunology Department, Austin Health, Melbourne, Australia; High Impact Epidemics, WHO Health Emergencies Programme, World Health Organization, Geneva, Switzerland; Cochrane Austria, Department for Evidence-based Medicine and Evaluation, University for Continuing Education Krems, Krems, Austria; Center for Public Health Methods, RTI International, Research Triangle Park, North Carolina, USA; Cochrane Austria, Department for Evidence-based Medicine and Evaluation, University for Continuing Education Krems, Krems, Austria

**Keywords:** peripherally inserted catheters, PICC, infections, complication, prevention

## Abstract

**Background:**

Peripherally inserted central catheters (PICCs) have a 29% complication rate. This systematic review evaluated 25 interventions to prevent PICC-associated infectious and noninfectious complications in participants of all ages.

**Methods:**

We searched electronic databases (MEDLINE, Embase, Cochrane Library, World Health Organization Global Index Medicus, CINAHL) and reference lists for randomized (RCTs) and nonrandomized controlled trials published between 1 January 1980-8 May 2024. We dually selected studies, assessed risk of bias, extracted data, and rated certainty of evidence (COE). We included single interventions of interest and combinations of at least 2 (bundle/multimodal). If 3 or more RCTs existed, we conducted Bayesian random-effects meta-analyses.

**Results:**

Seventy-four studies met our eligibility criteria (60 evaluated single interventions, 14 bundle/multimodal), addressing 13 of 25 research questions. The majority were conducted in high-income countries; 36 focused on neonates. Evidence was very uncertain for 11 of the 13 research questions. Stronger COE showed that ultrasound-guided catheter insertion reduced phlebitis/thrombophlebitis in adults compared with non–ultrasound-guided (5 RCTs; risk ratio [RR], 0.19; 95% credible interval, .08–.50); silicone catheters increased phlebitis/thrombophlebitis compared with nonsilicone (1 RCT; RR, 2.00; 95% confidence interval [CI], 1.26–3.17). Bundle interventions decreased local infections (1 RCT; RR, 0.47; 95% CI, .31–.72) and phlebitis/thrombophlebitis in adults (1 RCT; RR, 0.35; 95% CI, .22–.56) compared with routine care.

**Conclusions:**

Ultrasound-guided catheter insertion and nonsilicone catheters effectively prevented PICC complications. The evidence for other comparisons was too uncertain to draw conclusions, highlighting the urgent need for additional studies on prevention and control interventions.

## Teaser

Data support 2 preventive measures for reducing peripherally inserted central catheter–associated complications: ultrasound-guided catheter insertion and nonsilicone catheters. Bundle interventions also appear effective. An urgent need exists for additional high-quality studies on this important topic.

Reliable intravenous access is vital to the administration of essential fluids and medications, especially in critically ill patients, making vascular access devices a fundamental element of hospital care [1] and central vein catheterization a routine procedure in clinical practice. Peripherally inserted central catheters (PICCs) achieve central vein access via placement in the upper arm instead of the neck, chest, or groin. Preferred over central catheters due to their easy insertion, short procedure time, reliable intravenous access, and high patient satisfaction rate [2], more than 2.5 million people receive PICCs annually [2]. Despite their versatility and widespread use [3], PICCs are associated with complications, both infectious and noninfectious, in 8%–30% of cases [4–6]. Infectious complications, which affect 16%–29% of cases, are particularly concerning [7]. Catheter-associated bloodstream infections (CABSIs) carry substantial life-threatening risks, affecting 0.5%–13% of PICCs [8].

Improving PICC management practices is essential for minimizing complications and ensuring patient safety during healthcare delivery. International organizations are actively working to identify effective interventions, applied alone or in combination (bundle), for preventing and controlling both infectious and noninfectious complications during catheter insertion, maintenance, access, and removal [9–11]. Thus, the World Health Organization (WHO) recently published its first global guideline focusing on PICC-associated infection prevention [10]. Implementing these interventions in real-world scenarios is challenging because human and material resources vary across countries, leading to differences in their effectiveness [12]. Complexity also arises from the need to implement multiple measures in combination, potentially presenting logistical and operational hurdles in healthcare settings. While the efficacy of combined interventions has been established for central lines, limited evidence exists for PICCs [13, 14].

In this systematic review, we evaluated 25 interventions to prevent PICC-associated infectious and noninfectious complications.

## METHODS

We conducted a systematic review based on 25 research questions (Table 1), each representing a unique intervention, following the Cochrane methodology [15] and adhering to the Preferred Reporting Items for Systematic Reviews and Meta-Analyses statement reporting guidelines [16]. The review protocol was registered on the Open Science Framework (https://osf.io/exdb4). This review is part of a larger systematic review addressing vascular catheters and included more than 200 studies for 50 comparisons conducted to provide the evidence basis for developing the above-mentioned WHO guideline. The peripheral intravenous catheter results are published elsewhere [17]. This article focuses on the PICC results.

**Table 1.** Research Questions and Interventions

Insertion
1. What is the impact of a sterile insertion technique compared with routine practice (technique without the specific requirement for sterility) on the rates of catheter-associated infectious and noninfectious complications and mortality?
2. What is the impact of chlorhexidine-containing skin disinfectant compared with non-chlorhexidine-containing skin disinfectant before catheter insertion on the rates of catheter-associated infectious and noninfectious complications and mortality?
3. What is the impact of catheter insertion by an individual with catheter insertion training/certification compared with an individual with no requirement for formal training/certification (“routine practice”) on the rates of catheter-associated infectious and noninfectious complications, mortality, and overall adverse events related to insertion?
4. What is the impact of catheter insertion by an individual wearing gloves (sterile or nonsterile) compared with an individual not specifically required to wear gloves (“routine practice”) on the rates of catheter-associated infectious and noninfectious complications, mortality, and overall adverse events related to insertion?
5. What is the impact of catheter insertion by an individual wearing sterile gloves compared with an individual wearing nonsterile gloves on the rates of catheter-associated infectious and noninfectious complications and mortality?
6. What is the impact of catheter insertion by an individual using a standardized insertion pack/kit compared with an individual not using a standardized insertion pack/kit (“routine practice”) on the rates of catheter-associated infectious and noninfectious complications and mortality?
7. What is the impact of catheter insertion with ultrasound-guided assistance compared with insertion without ultrasound-guided assistance (“routine practice”) on the rates of catheter-associated infectious and noninfectious complications and mortality?
8. What is the impact of catheter insertion in the distal section (below cubital fossa) compared with the proximal section (cubital fossa or above) of the upper limb on the rates of catheter-associated infectious and noninfectious complications and mortality?
9. What is the impact of catheter insertion in the upper limb (anywhere) compared with the lower limb (anywhere) on the rates of catheter-associated infectious and noninfectious complications, mortality, and overall adverse events related to insertion?
10. What is the impact of a catheter (cannula section) made of silicone material compared with nonsilicone material (eg, polyurethane) on the rates of catheter-associated infectious and noninfectious complications and mortality?
11. What is the impact of a catheter secured with an occlusive dressing (eg, semipermeable, transparent dressing) compared with a nonocclusive dressing (eg, gauze, other) on the rates of catheter-associated infectious and noninfectious complications, mortality, and overall adverse events related to catheter dressing?
12. What is the impact of catheter insertion by an insertion team compared with insertion by an individual who is not part of an insertion team on the rates of catheter-associated infectious and noninfectious complications, mortality, and complications related to insertion?
13. What is the impact of catheter insertion by a clinician who has used soap and water compared with a clinician who has used alcohol-based handrub on the rates of catheter-associated infectious and noninfectious complications and mortality?
14. What is the impact of catheter insertion using local anesthetic at the insertion site compared with no local anesthetic at the insertion site on the rates of catheter-associated infectious and noninfectious complications and mortality?
15. In neonates, what is the impact of a catheter inserted in the scalp compared with a catheter inserted anywhere else on the rates of catheter-associated infectious and noninfectious complications and mortality?
Maintenance
16. What is the impact of catheter maintenance using a formalized sterile dressing protocol compared with no formalized sterile dressing protocol (“routine practice”) on the rates of catheter-associated infectious and noninfectious complications and mortality?
17. What is the impact of catheter management with scheduled continuous intravenous fluid infusion compared with no scheduled continuous intravenous fluid infusion (intermittent or no infusion) on the rates of catheter-associated infectious and noninfectious complications and mortality?
18. What is the impact of systematic sterile flushing (saline or other) compared with no flushing after product administration on the rates of catheter-associated infectious and noninfectious complications and mortality?
19. What is the impact of saline flushing/locking compared with anticoagulant flushing/locking after product administration on the rates of catheter-associated infectious and noninfectious complications and mortality?
20. What is the impact of catheter maintenance with a schedule of regular administration (tubing) set changes compared with no specified schedule of regular administration (tubing) set changes on the rates of catheter-associated infectious and noninfectious complications and mortality?
Access
21. What is the impact of catheter access using a defined sterile/aseptic protocol compared with no formal sterile/aseptic protocol on the rates of catheter-associated infectious and noninfectious complications and mortality?
22. What is the impact of catheter access using a closed-access device system compared with an open-access device system on the rates of catheter-associated infectious and noninfectious complications and mortality?
23. What is the impact of single-lumen compared with multilumen catheters on the rates of catheter-associated infectious and noninfectious complications and mortality?
Removal
24. What is the impact of catheter removal based on a defined schedule compared with removal based only on clinical indication due to a suspected or confirmed complication on the rates of catheter-associated infectious and noninfectious complications and mortality?
25. What is the impact of catheter removal/replacement within 24 hours if inserted under emergency conditions compared with a catheter not removed/replaced within 24 hours if inserted under emergency conditions on the rates of catheter-associated infectious and noninfectious complications and mortality?

### Systematic Literature Search

An experienced information specialist (I. K.) searched Ovid MEDLINE, Embase.com, the Cochrane Database of Systematic Reviews and Cochrane Central Register of Controlled Trials (Cochrane Library/Wiley), the WHO Global Index Medicus (https://pesquisa.bvsalud.org/gim/), and CINAHL (Ebsco) for the period 1 January 1980–8 May 2024, using free-text and subject headings (see Supplementary Material A for detailed methodology and search strategies). We included only peer-reviewed publications without language or setting restrictions. Additionally, we reviewed references from relevant systematic reviews (2018–2022) and consulted experts for studies our search may have missed.

### Study Selection and Data Extraction

After pilot-testing the screening form using 50 abstracts and 5 full texts, pairs of 2 reviewers (A. d., A. M. C., L. P., A. C., P. R., B. N. S.) screened all records independently at the title/abstract and full-text level against the predefined eligibility criteria (Supplementary Material B, Table B1) using DistillerSR (Evidence Partners, Ottawa, Canada). Any decision conflict was resolved by discussion or involving a third reviewer (G. G.).

Our review focused on outpatient and inpatient populations of any age, race, and ethnicity that required a PICC. We aimed to investigate the impact of predefined interventions that targeted insertion, maintenance, access, or removal, either as a single intervention or in combinations of at least 2 (ie, a bundle) compared with standard of care (ie, no intervention of interest).

We developed, pilot-tested, and used a structured data extraction form to ensure uniformity in the data abstraction. One reviewer (A. d., A. M. C., L. P., A. C., P. R., B. N. S.) extracted relevant data related to the characteristics of the study populations, interventions, comparators, study designs, methods, and outcomes into standardized tables, which a second reviewer (A. d., A. M. C., L. P., A. C., P. R., B. N. S.) checked for completeness and accuracy.

### Classification of the Study Designs and Assessment of the Risk of Bias

Two independent reviewers (A. d., A. M. C., L. P., A. C., P. R., B. N. S.) classified the included study designs using the US Agency for Healthcare Research and Quality's classification [18]. They conducted risk-of-bias assessments at the study level, switching to outcome level when different methodological aspects imposed different risks of bias for different outcomes. We used the Cochrane Risk of Bias tool (RoB 2.0) for randomized, controlled trials (RCTs) [19], the Risk Of Bias In Non-randomized Studies of Interventions tool for non-RCTs with concurrent controls [20], and the Effective Public Health Practice Project tool for before–after studies and interrupted time series [21]. Discrepancies were solved by discussion or involving a third reviewer (G. G.). To ensure reporting consistency, we harmonized the tools’ ratings into 3 categories: low, some concerns, and high (Supplementary Material C, Table C1).

### Synthesis of the Evidence

When available, we prioritized RCTs and used other study designs when RCT data were unavailable. For outcomes with 3 or more RCTs, we conducted Bayesian meta-analyses using restrictive priors (μ∼Normal[0,1], τ∼Half Cauchy[0,0·5]). This approach, which deviated from our protocol (https://osf.io/exdb4), offered advantages such as easier interpretation and ability to address challenges with null hypothesis testing. Calculations were performed in R (v. 4.2.2) using brms and rstan packages, with each meta-analysis based on 16 000 iterations. Data wrangling and plot creation were executed using the tidyverse package. We handled zero events by replacing them with 0.5. For outcomes with only non-RCTs, we reported effect ranges.

### Rating the Importance of Outcomes

The WHO Guideline Development Group members assessed each outcome's relative importance using a modified Delphi method and 9-point Likert scale. Following the Grading of Recommendations, Assessment, Development, and Evaluations (GRADE) Working Group's guidance [22], we prioritized and assessed the certainty of evidence (COE) for the 7 outcomes rated as critical or important for decision-making.

### Certainty of Evidence

We assessed the COE using the GRADEpro online tool (https://www.gradepro.org/). Each key outcome's COE was initially assessed by 1 investigator (A. d., B. N. S.) and reviewed for plausibility and consistency by a senior investigator (G. G.). We focused on 7 key outcomes: 6 outcomes were applied across all comparisons (CABSI, CABSI–related mortality, sepsis, local infections, all-cause mortality, and phlebitis/thrombophlebitis), while 1 (catheter insertion–related complications) was specific to 4 comparisons (research questions 3, 4, 9, and 12; see Table 1). RCTs and non-RCTs started at high COE, while before–after studies, case-control studies, and interrupted time series started at low COE [22]. For each comparison, we developed summary of findings tables.

## RESULTS

**Figure 1.**
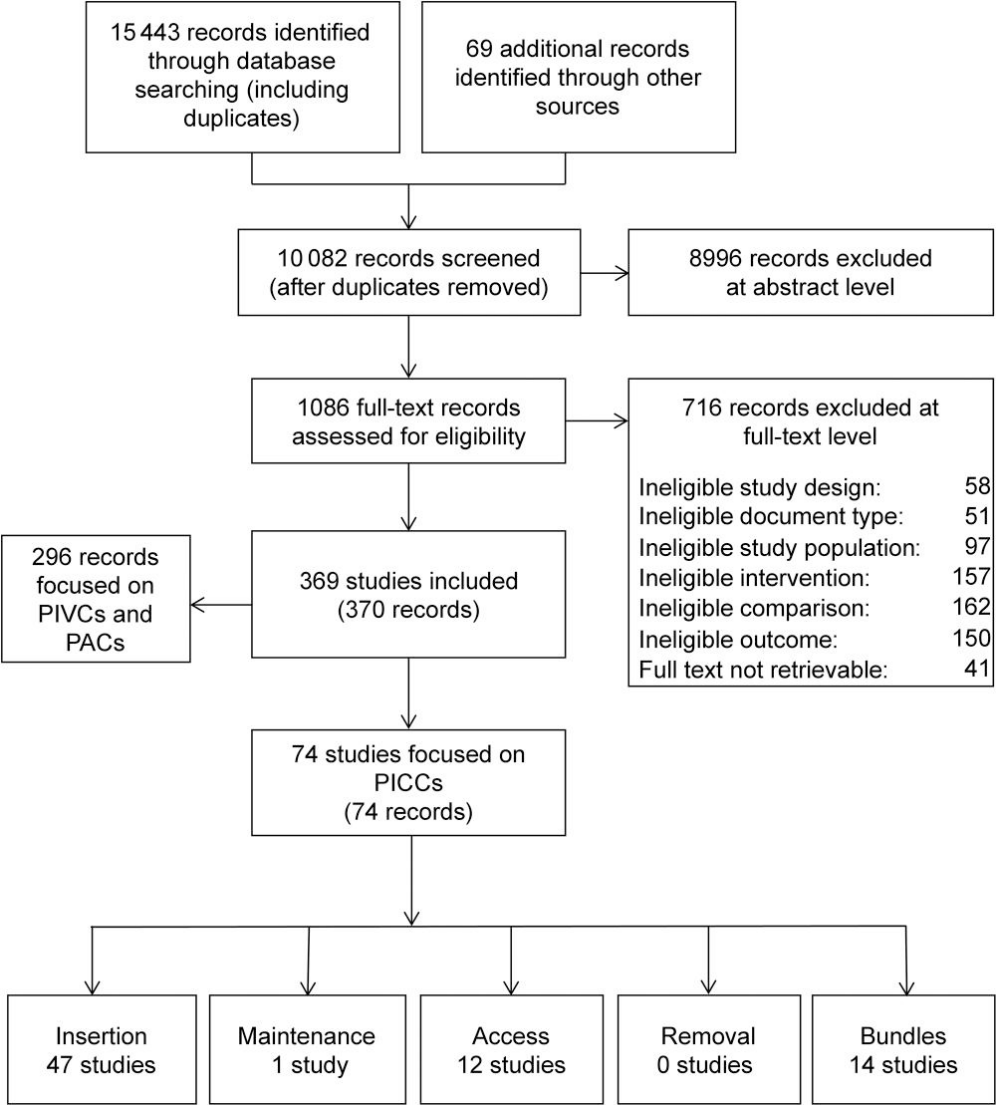
Flow diagram of the literature search. Abbreviations: PAC, peripheral arterial catheter; PICC, peripherally inserted central catheter; PIVC, peripheral intravenous catheter.

Of 10 082 references identified by our search, we included 74 studies (14 RCTs, 43 non-RCTs, 1 interrupted time series, 15 before–after, 1 case-control study; Figure 1), reporting on 51 834 participants (ranging from 38 to 11 135). Five studies (7%) did not report the number of participants. Sixty studies (81%) assessed the effect of individual interventions; 14 (19%) evaluated bundle interventions. Supplementary Material D lists the articles excluded during the full-text review and the reasons for exclusion. Supplementary Material E, Tables E1–E14 document the study characteristics and results.

**Figure 2.**
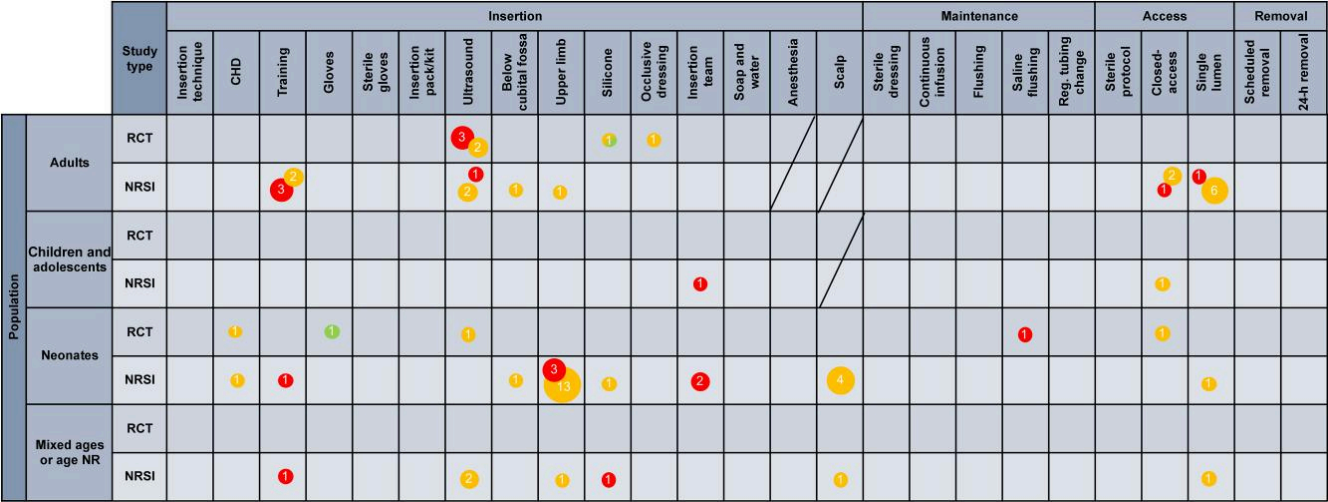
Distribution of studies addressing individual interventions. Each column represents a research question; rows represent population groups and study designs. Red circles represent studies with high risk of bias, yellow circles represent studies with some concerns, and green circles represent studies with low risk of bias. The yellow/green circle indicates studies with a low risk of bias for objective outcomes and some concerns for subjective outcomes. Numbers indicate the number of studies that addressed the specific research question. Crossed cells indicate that the intervention was not assessed for the age group. Abbreviations: CHD, chlorhexidine; NR, not reported; NRSI, nonrandomized, controlled trial; RCT, randomized, controlled trial; reg., regular.

Most of the included studies focused on neonates (36 studies, 49%), 1 (1%) focused on children and adolescents, and 30 (41%) evaluated adults. Seven (10%) did not report the age group or assessed a mixed population. Forty studies (54%) were conducted in high-income countries, and 34 (46%) were conducted in middle-income countries. We rated 29 studies (39%) as high risk of bias, 43 (58%) as some concerns, and 1 (1.5%) as low. One study (1.5%) was rated as low risk of bias for the objective outcomes and as some concerns for the subjective outcomes. Supplementary Material F and Figures F1–F3 detail the risk of bias assessments. The main methodological limitations were missing data and analysis not adjusted for confounders. Figure 2 illustrates the distribution of studies by designs, age groups, and research questions along with the associated risk of bias.

All results and corresponding COEs are presented in Table 2; the complete list of references is presented in Supplementary Material G. The complete GRADE assessments are detailed in Supplementary Material H and Tables H1–H14.

**Table 2.** Study Results and Certainty of Evidence

Comparisons		Number (N) of Participants Included in the Analysis for This Outcome Treatment Effect With 95% CI Certainty of Evidence (High, Moderate, Low, or Very Low) Number (K) of Studies^a^					
		CABSI/CRBSI	Sepsis	Local Infections	All-Cause Mortality	Phlebitis/Thrombophlebitis	Overall Adverse Events Related to Intravascular Catheter Insertion
Insertion							
Chlorhexidine versus nonchlorhexidine-containing skin disinfection	Neonates	K = 1 (Garland 2009) N = 48 4.2% versus 4.2% RR 1.00 (.07 to 15.08)	NA	NA	NA	NA	NA
		⨁◯◯◯					
Catheter inserted by an individual with catheter insertion training/certification versus routine practice	Adults	K = 2 (Sakai et al 2023; Zhang et al 2014) N = 1324 6.5% versus 7.8% aHR 0.82^b^ (0.44 to 1.54)	NA	NA	NA	K = 1 (Zhang et al 2014) N = 610 1.6% versus 6.7% RR 0.24 (0.09 to 0.64)	K = 1 (Sakai et al 2023) N = 2230 1.4% versus 5.1% RR 0.28 (0.17 to 0.48)
		⨁◯◯◯				⨁◯◯◯	⨁◯◯◯
	Neonates	K = 1 (Walters and Price 2019) N = 1631 CABSI/CRBSI/1000 peripheral line days: 9.11 versus 18.34	NA	NA	NA	NA	NA
		⨁◯◯◯					
Wearing gloves (either sterile or nonsterile) versus not specifically required to wear gloves	Neonates	K = 1 (Kaufman 2014) N = 120 6.7% versus 6.7% RR 1.00 (0.26 to 3.81)	NA	NA	K = 1 (Kaufman 2014) N = 120 10.0% versus 3.3% RR 3.00 (0.63 to 14.27)	NA	NA
		⨁◯◯◯			⨁◯◯◯		
Ultrasound-guided assistance versus insertion without ultrasound-guided assistance	Adults	K = 1 (Li et al 2014) N = 98 0.0% versus 2.1% RR 0.32 (0.01 to 7.67)	NA	K = 1 (Tan et al 2016) N = 319 1.3% versus 7.4% RR 0.17 (0.04 to 0.73)	K = 1 (Li et al 2014) N = 98 0.0% versus 2.1% RR 0.32 (0.01 to 7.67)	K = 5 (Li et al 2014; Tan 2016; Wang 2016; Zhang 2019; Qi 2012) N = 1744 1.1% versus 5.7% RR 0.17 (0.08 to 0.50)	NA
		⨁◯◯◯		⨁◯◯◯		⨁⨁◯◯	
	Neonates	NA	NA	NA	NA	K = 1 (Yin 2022) N = 94 0.9% versus 4.3% RR 0.20 (0.01 to 4.05)	NA
						⨁◯◯◯	
Insertion in distal section of the upper limb versus in the proximal section of the upper limb	Adults	K = 1 (Pongruangporn 2013) N = 647 NA OR 0.77 (0.47 to 1.20)	NA	NA	NA	NA	NA
		⨁◯◯◯					
	Neonates	K = 1 (Razavinejad 2023) N = 2500 NR OR 1.36 (0.78 to 2.35)	NA	NA	NA	NA	NA
		⨁◯◯◯					
Insertion in the upper limb versus insertion in the lower limb	Adults	NA	NA	NA	NA	K = 1 (Malinoski 2013) N = 124 20.3% versus 14.3% RR 1.42 (0.57 to 3.52)	NA
						⨁◯◯◯	
	Children and adolescents	K = 1 (Callejas et al 2016) N = 620 11.9% versus 5.4% RR 2.21 (1.08 to 4.54)	NA	NA	K = 1 (Callejas et al 2016) N = 620 9.5% versus 12.1% RR 0.79 (0.47 to 1.32)	K = 1 (Callejas et al 2016) N = 620 0.9% versus 0.7% RR 1.27 (0.14 to 11.23)	K = 1 (Callejas et al 2016) N = 620 12.6% versus 8.7% RR 1.44 (0.81 to 2.54)
		⨁◯◯◯			⨁◯◯◯	⨁◯◯◯	⨁◯◯◯
	Neonates	K = 6 (Gai 2022; Kinoshita 2019; Padilla-Sanchez 2019; Bashir 2016; Hu 2021; Ekaputri 2022) N = 1355 5%–10% versus 0%–25% Not estimable	K = 4 (Elmekkawi 2019; Wrightson 2013; Tsai 2009; López Sastre 2000) N = 2417 5%–12% versus 2%–23% Not estimable	K = 4 (Pet 2020; Ma 2015; Aggarwal 2001) N = 1388 0%–5% versus 0%–43% Not estimable	K = 3 (Elmekkawi 2019; Wrightson 2013; Tsai 2009) N = 1172 5%–6% versus 1%–6% Not estimable	K = 11 (Gai 2022; Pet 2020; Padilla-Sanchez 2019; Elmekkawi 2019; Bashir 2016; Ma 2015; Kisa 2015; Wrightson 2013; Tsai 2009; López Sastre 2000; Hoang 2008) N = 5837 0%–10% versus 0%–21% Not estimable	K = 5 (Pet 2020; Bashir 2016; Ma 2015; Bulbul 2010; Aggarwal 2001) N = 2325 3%–40% versus 20%–30% Not estimable
		⨁◯◯◯	⨁◯◯◯	⨁◯◯◯	⨁◯◯◯	⨁◯◯◯	⨁◯◯◯
Catheter made of silicone versus catheter made of nonsilicone material	Adults	K = 1 (Ong 2010) N = 392 2.6% versus 1.0% RR 2.55 (0.50 to 12.99)	K = 1 (Ong 2010) N = 392 2.1% versus 1.0% RR 2.04 (0.38 to 11.02)	NA	NA	K = 1 (Ong 2010) N = 392 23.2% versus 11.6% RR 2.00 (1.26 to 3.17)	NA
		⨁◯◯◯	⨁◯◯◯			⨁⨁◯◯	
	Children and adolescents	NA	NA	NA	NA	K = 1 (Linder 1984) N = 61 35.8% versus 18.2% RR 1.97 (0.74 to 5.26)	NA
						⨁◯◯◯	
	Neonates	NA	NA	NA	NA	K = 1 (Gomes de Souza 2021) N = 450 0.5% versus 2.4% RR 0.20 (0.02 to 1.67)	NA
						⨁◯◯◯	
Catheter secured with an occlusive dressing versus nonocclusive dressing	Adults	NA	NA	NA	NA	K = 1 (Chico-Padron 2011) N = 25 16.7% versus 7.7% RR 2.17 (0.22 to 20.94)	NA
						⨁◯◯◯	
Catheter inserted by an insertion team versus inserted by an individual not part of an insertion team	Children and adolescents	K = 1 (Pitts 2013) N = 669 per 1000 line days: 2.0 versus 9.12 Not estimable	NA	NA	NA	NA	NA
		⨁◯◯◯					
	Neonates	K = 1 (Levit 2020) N = 731 per 1000 line days (95% CI): 0.3 (.8 to 3.1) versus 1.6 (.01 to 1.2) Rate ratio 0.43 (0.08 to 2.34)	NA	NA	NA	K = 2 (Levit 2020; Yongshu 2019) N = 871 6.0% versus 17% RR 0.33 (0.11 to 0.98)^c^	K = 1 (Levit 2020) N = 731 Per 1000 line days (95% CI): 5.5 (4.0 to 7.7) versus 12.8 (10.1 to 16.1) Rate ratio 0.43 (0.29 0.65)
		⨁◯◯◯				⨁◯◯◯	⨁◯◯◯
Scalp versus other insertion site	Neonates	K = 1 (Padilla-Sanchez 2019) N = 140 0.0% versus 6.6% RR 0.38 (0.02 to 6.32)	K = 1 (López Sastre 2000) N = 123 2.4% versus 48.8% RR 0.05 (0.01 to 0.35)	K = 1 (Aggarwal 2001) N = 44 10.6% versus 16.0% RR 0.66 (0.13 to 3.22)	K = 1 (Callejas 2016) N = 689 13.0% versus 10.2% RR 1.28 (0.67 to 2.47)	K = 4 (Padilla-Sanchez 2019; Callejas 2016; Kisa 2015; López Sastre 2000) N = 2460 0%–16.1% versus 0.3%–9.4% Not estimable	NA
		⨁◯◯◯	⨁◯◯◯	⨁◯◯◯	⨁◯◯◯	⨁◯◯◯	
Maintenance							
Saline flushing versus anticoagulant flushing	Neonates	K = 1 (Araujo 2011) N = 133 0.0% versus 0.0% RR 2.75 (0.11 to 66.21)	K = 1 (Araujo 2011) N = 133 24.6% versus 17.2% RR 1.43 (0.73 to 2.82)	NA	NA	NA	NA
		⨁◯◯◯	⨁◯◯◯				
Access							
Closed-access device system versus open-access device system	Adults	K = 1 (Zerla 2015) N = 793 0.3% versus 2.9% RR 0.11 (0.02 to 0.57)	NA	NA	NA	NA	NA
		⨁◯◯◯					
	Neonates	K = 1 (Reiter 2006) N = 300 23.4% versus 19.3% RR 1.21 (0.83 to 1.96)	K = 1 (Rundjan 2015) N = 60 3.5% versus 26.7% RR 0.13 (0.02 to 0.94)	NA	NA	NA	NA
		⨁◯◯◯	⨁◯◯◯				
Single-lumen versus multilumen catheters	Adults	K = 4 (Rejane Rabelo-Silva 2022; Chopra 2014; Bae 2023; Barrigah-Benissan 2023) N = 12 725 0%–6% versus 0%–9.9% Not estimable	K = 1 (Liscynesky 2017) N = 187 5.0% versus 14.7% RR 0.34 (0.14 to 0.95)	NA	NA	NA	NA
		⨁◯◯◯	⨁◯◯◯				
	Neonates	K = 1 (Kinoshita 2019) N = 2383 NR aHR 0.39 (0.13 to 1.16)	NA	NA	NA	NA	NA
		⨁◯◯◯					
Bundle versus routine care/no intervention	Adults	K = 1 (Tian 2010) N = 234 1.8% versus 5.8% RR 0.31 (0.07 to 1.36)	NA	K = 1 (Liu 2013) N = 1490 4.0% versus 8.4% RR 0.47 (0.31 to 0.72)	NA	K = 1 (Liu 2013) N = 1490 3.1% versus 9.0% RR 0.35 (0.22 to 0.56)^b^	K = 1 (He 2022) N = 100 4.0% versus 18.0% RR 0.22 (0.05 to 0.98)^b^
		⨁◯◯◯		⨁⨁◯◯		⨁⨁◯◯	⨁◯◯◯
	Neonates	K = 1 (Royer 2010) N = NR per 1000 central line days: 0.0 versus 1.73^b^ Not estimable	K = 1 (Golombek 2002) N = 84 4.0% versus 40.0% RR 0.09 (0.01 to 0.64)	K = 1 (Ren 2022) N = 102 2.0% versus 3.9% RR 0.50 (0.05 to 5.34)	K = 1 (Bayoumi 2021) N = 1336 3.1% versus 2.4% RR 1.32 (0.63 to 2.74)^b^	K = 1 (Ren 2022) N = 102 0.8% versus 3.9% RR 0.20 (0.01 to 4.06)	K = 1 (Ren 2022) N = 102 0.8% versus 3.9% RR 0.20 (0.01 to 4.06)
		⨁◯◯◯	⨁◯◯◯	⨁◯◯◯	⨁◯◯◯	⨁◯◯◯	⨁◯◯◯
Bundle (single-lumen silicone catheters) versus bundle (multilumen nonsilicone catheters)	Neonates	K = 1 (Costa 2016) N = 401 7.1% versus 27.3% RR 0.26 (0.15 to 0.45)	NA	NA	NA	NA	K = 1 (Paiva 2013) N = 270 35.6% versus 45.7% RR 0.78 (0.57 to 1.05)
		⨁◯◯◯					⨁◯◯◯

^a^Complete list of references is presented in Supplementary Material G.

^b^Based on the larger or methodologically better study.

^c^Self-calculated.

⨁◯◯◯ very low certainty of evidence; ⨁⨁◯◯ low certainty of evidence. Abbreviations: aHR, adjusted hazard ratio; CABSI, catheter-associated bloodstream infection; CI, confidence interval; CRBSI, catheter-related bloodstream infection; NA, not applicable; NR, not reported; OR, odds ratio; RR, risk ratio.

We identified evidence on 13 of 25 research questions. The following sections present the evidence for preventive measures during catheter insertion, maintenance, and access. We did not find any eligible study that addressed removal. Additionally, we provide a summary of studies that evaluated bundle/multimodal interventions.

### Insertion

We identified 47 studies (24 399 participants) that addressed 10 of 15 research questions on catheter insertion (10 RCTs, 28 non-RCTs, 8 before–after studies, 1 case-control study): chlorhexidine disinfection, insertion training, glove use, ultrasound-guided insertion, insertion below the antecubital fossa, insertion in the upper limb, silicone catheters, occlusion dressing, insertion team use, and scalp-inserted catheters.

Seventeen studies focused on adults, 1 focused on children and adolescents, and 24 focused on neonates. Five evaluated mixed groups or did not report the age group. For 8 research questions, the COE was rated as very low for all outcomes (see Figure 2). For 2, the COE of 1 outcome of interest was rated as low.

In adults, the evidence indicated a lower phlebitis/thrombophlebitis incidence with ultrasound-inserted compared with non–ultrasound-inserted catheters (meta-analysis of 5 RCTs [23–27], 1744 participants; 1.1% versus 5.7%; risk ratio [RR] [95% credible interval], .19 [.08–.50]; low COE; Supplementary Material I/Figure I1). Silicone material catheters may increase phlebitis/thrombophlebitis compared with nonsilicone material catheters (1 RCT [28]; 392 participants; 23.2% versus 11.6%; RR [95% confidence interval [CI], 2.00 [1.26–3.17]; low COE), and catheter insertion by an individual with training/certification may reduce the incidence of phlebitis/thrombophlebitis (1.6% versus 6.7%; RR [95% CI], .24 [.09–.64]; very low COE) [29] and overall adverse events (1.4% versus 5.1%; RR [95% CI], .28 [.17–.48]; very low COE) [30] compared with no insertion training. In neonates, insertion training/certification may reduce CABSI (9.11 versus 18.34 per 1000 peripheral line days, very low COE) [31].

A very low COE indicates that catheter insertion in the upper limb may lead to a higher CABSI incidence (11.9% versus 5.4%; RR [95% CI], 2.21 [1.08–4.54]) in children and adolescents compared with lower limb insertion [32], while catheters inserted by an insertion team may decrease CABSI (2.0 versus 9.12 per 1000 line days) in children and adolescents [33] and reduce phlebitis/thrombophlebitis (6.0% versus 17%; RR [95% CI], .33 [.11–.98]) [34, 35] and overall adverse events (5.5 versus 12.8 per 1000 line days) [34] in neonates compared with no insertion team. Additionally, a very low COE suggests that scalp-inserted catheters may result in a lower sepsis incidence in neonates (2.4% versus 48.8%; RR [95% CI], .05 [.01–.35]) compared with other insertion places [36].

The remaining outcomes indicated little to no difference in effectiveness compared with standard of care (see Table 2).

### Maintenance

We identified 1 RCT (133 participants) that focused on neonates and addressed 1 (saline flushing) of 5 research questions on catheter maintenance. Very low COEs indicated little to no difference in CABSI and sepsis with lock-off saline and anticoagulant flushing [37].

### Access

We identified 12 studies (17 689 participants) that addressed 2 of 3 research questions on catheter access (1 RCT, 11 non-RCTs): closed-access systems and single-lumen catheters. One study focused on adults, 9 focused on neonates, and 1 did not report the age group [38]. Based on very low COE, closed-access system catheters may reduce CABSI in adults (0.3% versus 2.9%; RR [95% CI], .11 [.02–.57]) [39] and sepsis in neonates (3.5% versus 26.7%; RR [95% CI], .13 [.02–.94]) [40] compared with open-access system catheters. Similar results were reported for single-lumen compared with multilumen catheters. The remaining outcomes indicated little to no difference compared with routine care (see Table 2).

### Interventions Implemented Through Bundle and/or Multimodal Strategies

**Figure 3.**
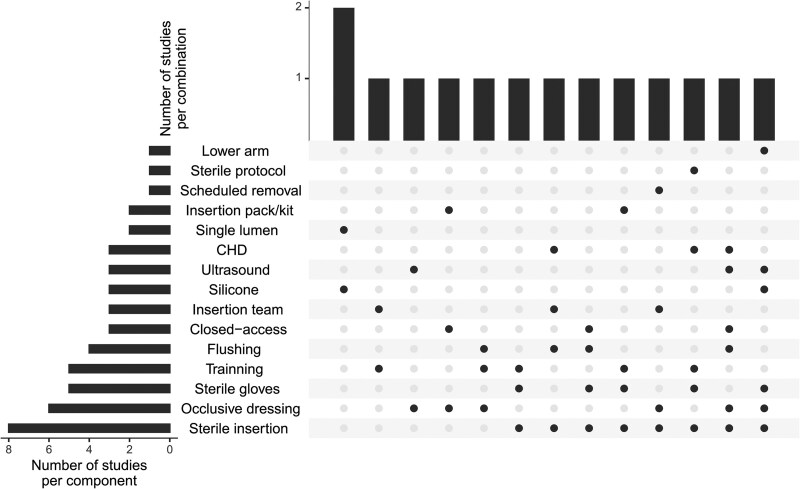
Combination of interventions within bundle studies. The bars from the upper graph indicate the number of studies addressing each combination of interventions. The left-hand side graph displays the number of studies that included each specific intervention of interest. Each bubble column represents a unique combination of interventions; black bubbles represent the bundle components. Abbreviation: CHD, chlorhexidine.

Fourteen studies (2 RCTs, 12 non-RCTs; 9613 participants) evaluated various intervention combinations in adults and neonates (Figure 3); 1 focused on a mixed population of children and adults. One study did not report the number of evaluated participants or catheters; another reported only the number of catheters. Studies combined 2 or more interventions during PICC insertion and maintenance; insertion and access; insertion and removal; and insertion, maintenance, and access (see Supplementary Material E, Table E14). Twelve studies used no intervention/routine care as a comparator; 2 compared 2 catheter types (single-lumen silicone compared with double-lumen polyurethane).

One RCT (1490 participants) indicated a lower incidence of local infections (3.96% versus 8.44%; RR [95% CI], .47 [.31–.72]; low COE) and phlebitis/thrombophlebitis in adults (3.14% versus 8.97%; RR [95% CI], .35 [.22–.56]; low COE) with a bundle insertion intervention (combination of sterile insertion, sterile gloves, ultrasound-guided insertion, lower arm insertion, silicone catheter, and occlusive dressing) compared with routine care [41].

Evidence rated as very low COE suggested that bundle intervention/multimodal strategies (ultrasound-guided insertion and occlusive dressing [42] or sterile insertion, chlorhexidine, insertion training, sterile gloves, and sterile access protocol [43]) may also decrease overall adverse events in adults compared with routine care (4.5% versus 18%; RR [95% CI], 0.22 [.05–.98]) [42]. One controlled-cohort study also reported fewer adverse events in neonates when a bundle/multimodal strategy was used (training, nighttime saline or heparin flushing, occlusive dressing; 7.8% vs 25.5%; RR [95% CI], .31 [.11–.88]; very low COE) [44].

Two controlled-cohort studies reported lower CABSI rates (7.0% versus 27.3%; RR [95% CI], .27 [.15–.48]; very low COE) [45] and numerically fewer overall adverse event risks (35.4% versus 45.6%; RR [95% CI], .78 [.57–1.05]; very low COE) [46] in neonates with single-lumen silicone compared with double-lumen polyurethane catheters. Other outcomes indicated little to no difference compared with routine care.

## DISCUSSION

To the best of our knowledge, this is the first systematic review to provide an extensive evaluation of interventions to prevent infectious and noninfectious catheter-associated complications in patients who require a PICC.

We included 74 studies that addressed 13 of the 25 research questions identified for the review. To ensure real-world applicability, we included both individual and combinations of at least 2 interventions of interest. Based on the strongest available evidence, ultrasound-guided catheter insertion may decrease phlebitis/thrombophlebitis incidence in adults compared with non–ultrasound-guided insertion. Additionally, catheters made of nonsilicone material may decrease the phlebitis/thrombophlebitis incidence in adults compared with those made of silicone material. The evidence for other outcomes was rated as very low COE, indicating limited confidence in the results. In bundle studies, 1 RCT reported a lower local infection incidence in the bundle/multimodal group compared with routine care. Further, single-lumen silicone catheters may reduce CABSI compared with double-lumen polyurethane catheters when used in a bundle/multimodal strategy. The overall adverse events incidence was also lower with bundle/multimodal interventions. These results align with existing evidence [13, 47].

For 12 research questions, no eligible evidence was identified. Even when some interventions of interest (sterile insertion techniques, sterile gloves, standardized insertion pack/kit use, flushing, access using a sterile protocol, scheduled removal) were part of bundle/multimodal strategies, the evidence was very uncertain. While most studies evaluated neonates, their methodological limitations compromised the evidence's reliability.

Putting our results in context with other evidence syntheses, we highlight Chen et al's systematic review in which they investigated PICC complications according to insertion site, specifically comparing upper with lower limb insertion [48]. The authors found no statistically significant differences in catheter-related infections or phlebitis. In our review, we included 4 additional studies using a more recent search strategy. Despite similar trends in the results, we were unable to draw conclusions due to the very low COE. No other similar evidence syntheses were identified.

Our review and its evidence base have several limitations. First, most studies focused on neonates and adults; only 1 evaluated children and adolescents. The findings cannot be extrapolated to these age groups, as intravenous access differs due to variations in underlying conditions, activity levels, vein size and location, and appropriate catheter diameters [49]. Second, most studies were published in high-income countries, complicating the results’ generalizability due to significant variability in human and financial resources. Also, our review covered an extensive time period during which hospital practices have evolved. Changes in hospital conditions and practices may lead to differences in the comparator condition “standard of care” and limit the applicability of older study results to current practice [9, 50]. Third, although we included studies that focused on bundle/multimodal strategies, reflecting real-world clinical practice where multiple interventions are often combined, the variability in these bundles’ components across studies makes it challenging to generalize the results and should be interpreted with caution. Fourth, we found no evidence to assess the impact of 12 preventive measures. Similarly, for the 13 research questions where data were available, reliable evidence was lacking for nearly all outcomes of interest. Fifth, we were able to conduct meta-analyses to combine only the RCT results. Although most of the included studies were non-RCTs, we provided narrative descriptions of these due to their methodological restrictions. Finally, despite our rigorous methods and comprehensive searches, we cannot exclude publication bias and selective outcome reporting.

Together with our previous publication on peripheral intravenous catheters [17], this systematic review addresses and underscores significant, critical research gaps, including the lack of evidence on this topic and methodological limitations in the available studies. An urgent need exists for high-quality studies that use a prospective controlled approach, ideally RCTs, to encompass all age groups from countries across a diverse range of income levels. Multicenter studies could address limited statistical power for rare but serious complications such as CABSI. Future studies should also investigate preventive interventions to provide the necessary data for informed decision-making. Bundle/multimodal strategies offer a practical alternative to single interventions, effectively reducing complications without increasing risks. While insertion-focused interventions show promising results, it remains unclear which are most critical for optimal outcomes. Future studies should systematically compare different bundle combinations to identify the safest and most effective approaches. We acknowledge the significant variability in PICC use regarding clinical indications, types, scope, catheter duration, and patient comorbidities, which poses challenges in conducting such studies. Given the widespread PICC use in clinical practice and the concerning high rates of associated complications, a compelling argument exists to expand the research in this area. Finally, given the limited evidence, healthcare providers and policymakers should also consider clinical experience, resource availability, and patient values and preferences to make informed decisions that better address patients’ needs and circumstances.

## Supplementary Material

ciaf063_Supplementary_Data

## Supplementary Data

Supplementary materials are available at *Clinical Infectious Diseases* online. Consisting of data provided by the authors to benefit the reader, the posted materials are not copyedited and are the sole responsibility of the authors, so questions or comments should be addressed to the corresponding author.

## Notes

***Author contributions.***  A. d. contributed to the funding acquisition, developed the systematic review protocol, collected data, interpreted the results, and wrote the first draft of the manuscript, which all the other authors reviewed. A. M. C., L. P., A. C., and P. R. collected data. I. K. performed systematic review searches. R. E. performed the statistical data analysis. B. A., M. L. G., and J. T. assisted in developing the research questions and study eligibility criteria and selected the outcomes of interest. G. G. contributed to supervision and validation of the results. B. N. S. contributed to the funding acquisition, reviewed the systematic review protocol, collected data, and interpreted the results. All authors read and approved the final manuscript and reviewed all documents and materials. a. d. is the guarantor for this work.

***Acknowledgments.*** The authors gratefully acknowledge Edith Kertesz from Cochrane Austria for project administration and formatting and Walter Zingg, chair of the Guideline Development Group, for his contributions to developing the research questions, study eligibility criteria, and selected outcomes of interest. The authors acknowledge the use of a large language model (ChatGPT 4) for editorial assistance in preparing the manuscript. They used it to improve the language of the abstract, introduction, and discussion sections. Specifically, they asked ChatGPT 4 to improve clarity and correct spelling and grammatical errors. The authors checked and ensured that the suggestions from ChatGPT 4 that were made to the text did not alter its meaning.

***Disclaimer.*** The opinions expressed here are those of the authors and do not reflect the official position of World Health Organization (WHO). WHO takes no responsibility for the information provided or the views expressed in this article. The WHO team assisted in developing the research questions, study eligibility criteria, selected outcomes of interest, and final manuscript completion but was not involved in the literature searches, study selection, data extraction, study appraisal, or data synthesis.

***Data availability.*** Data are available in the Supplementary Material.

***Financial support.*** This work was supported by the World Health Organization (WHO) (Open Science Framework; https://osf.io/exdb4).
