# Supplementary material for: Effectiveness and Safety of Methods to Prevent Bloodstream and Other Infections and Noninfectious Complications Associated With Peripherally Inserted Central Catheters: A Systematic Review and Meta-Analysis
Source: Clin Infect Dis. 2025 Feb 12;82(3):459–72. doi: 10.1093/cid/ciaf063 (PMC13016633; doi:10.1093/cid/ciaf063)
Supplement: ciaf063_Supplementary_Data [file ciaf063_supplementary_data.docx]

| Effectiveness and safety of methods to prevent bloodstream- and other infections associated with peripherally inserted central catheters: A systematic review and meta-analysis |
| --- |
| Supplementary Material |
|  |

Table of contents

[Supplement A- Search Strategy 1](#_Toc188271858)

[Supplement B- Study Eligibility Criteria 12](#_Toc188271859)

[Supplement C- Harmonisation of Rob Assessments 13](#_Toc188271860)

[Supplement D- List of Excluded Studies and Reasons of Exclusion 14](#_Toc188271861)

[Supplement E- Outcome Tables 44](#_Toc188271862)

[Supplement F- Risk of Bias Ratings of Included Studies 83](#_Toc188271863)

[Supplement G- Complete list of references for Table 2 86](#_Toc188271864)

[Supplement H- Summary of findings tables 90](#_Toc188271865)

[Supplement I- Forest plot 121](#_Toc188271866)

[References 122](#_Toc188271867)

**List of Tables:**

[Table B1: Study eligibility criteria 12](#_Toc175571997)

[Table C1: Risk of bias ratings 13](#_Toc175571998)

[Table E1: Chlorhexidine-containing antiseptic for skin preparation vs non-chlorhexidine containing antiseptics 44](#_Toc177383507)

[Table E2: Training vs no specified formal training 45](#_Toc177383508)

[Table E3: Gloves vs no gloves 48](#_Toc177383509)

[Table E4: Ultrasound-guided insertion vs non-ultrasound-guided guided 49](#_Toc177383510)

[Table E5: Lower vs upper arm 54](#_Toc177383511)

[Table E6: Upper vs lower limb 55](#_Toc177383512)

[Table E7: Silicone vs nonsilicone 63](#_Toc177383513)

[Table E8: Occlusive vs non-occlusive dressings 64](#_Toc177383514)

[Table E9: Insertion team vs no specific insertion team 65](#_Toc177383515)

[Table E10: Scalp inserted vs anywhere other than the scalp 67](#_Toc177383516)

[Table E11: Saline flushing/locking after product administration vs anticoagulant flushing 69](#_Toc177383517)

[Table E12: Use of closed-access device system (e.g., luer lock) vs open-access system 70](#_Toc177383518)

[Table E13: Single lumen vs. multi-lumen 72](#_Toc177383519)

[Table E14: Bundle studies 76](#_Toc177383520)

[Table H1: A catheter inserted only by an individual with catheter insertion training/certification compared to insertion by an individual with no requirement for formal training/certification ("routine practice") in participants requiring a PICC 89](#_Toc188342699)

[Table H2: A catheter inserted by an individual wearing gloves (either sterile or non-sterile) compared to insertion by an individual not specifically required to wear gloves in participants requiring a PICC 92](#_Toc188342700)

[Table H3: Catheter insertion with ultrasound-guided assistance compared to insertion without ultrasound-guided assistance in participants requiring a PICC 94](#_Toc188342701)

[Table H4: Catheter insertion in distal section of the upper limb compared to insertion in the proximal section of the upper limb in participants requiring a PICC 96](#_Toc188342702)

[Table H5: Catheter insertion in the upper limb compared to insertion in the lower limb in participants requiring a PICC 98](#_Toc188342703)

[Table H6: Catheter made of silicone material compared to catheter made of non-silicone material in participants requiring a PICC 101](#_Toc188342704)

[Table H7: Catheter secured with an occlusive dressing compared to catheter secured with non-occlusive dressing in participants requiring a PICC 103](#_Toc188342705)

[Table H8: Catheter inserted by an insertion team compared to catheter inserted by an individual not part of a specific insertion team in participants requiring a PICC 105](#_Toc188342706)

[Table H9: Catheter inserted in the scalp compared to catheter inserted anywhere other than the scalp in participants requiring a PICC 107](#_Toc188342707)

[Table H10: Saline flushing compared to anticoagulant flushing in participants requiring a PICC 109](#_Toc188342708)

[Table H11: Catheter access using a closed-access device system compared to catheter access using an open-access device system in participants requiring a PICC 111](#_Toc188342709)

[Table H12: Single-lumen catheter compared to multi-lumen catheter in participants requiring a PICC 113](#_Toc188342710)

[Table H13: Bundle compared to routine in participants requiring a PICC 115](#_Toc188342711)

[Table H14: Single lumen silicon catheter compared to double-lumen polyurethan catheter in participants requiring a PICC 118](#_Toc188342712)

**Table of Figures**

[Figure F1. Risk of Bias Ratings for RCTs (RoB2 tool) 83](#_Toc177481996)

[Figure F2. Risk of Bias Ratings for observational studies (Robins I tool 84](#_Toc177481997)

[Figure F3. Risk of Bias Ratings for before-after studies 85](#_Toc177481998)

[Figure I1. Phlebitis/thrombophlebitis in adults 120](#_Toc188342739)

# Supplement A- Search Strategy

An experiences information specialist (IK) designed and conducted database searches. Search filters for RCTs and non-randomised controlled studies were adapted from Haynes et al.[1] and Waffenschmidt et al.[2] respectively. The WHO Guideline Development Group provided references of interest, which the author team supplemented with preliminary searches. These references were used to assess and refine the MEDLINE search strategy. Due to time constraints, no formal peer review of the search strategy took place. However, the WHO Guideline Development Group and the first author reviewed the strategy. Polyglot Search Translator[3] was used for the initial translation of the MEDLINE strategy to other databases, followed by manual revisions.

Database searches were originally designed and conducted in March 2023 and updated 7-8 May, 2024. The full search strategies are reported below. As this review is part of a larger project concerning several types of vascular catheters, they also contain search terms only relevant to peripheral intravenous catheters and peripheral arterial catheters.

**Search strategies overview**

| **Database name, time span, and host** | **Date searched** |
| --- | --- |
| Ovid MEDLINE(R) ALL 1946 to May 06, 2024 | 7 May 2024 |
| Embase.com (Elsevier) | 7 May 2024 |
| Cochrane Database of Systematic Reviews Issue 3 of 12, March 2023, Cochrane Central Register of Controlled Trials Issue 2 of 12, February 2023 (Cochrane Library/Wiley) | 7 May 2024 |
| WHO Global Index Medicus (https://pesquisa.bvsalud.org/gim/) | 7 May 2024 |
| CINAHL (Ebsco) | 8 May 2024 |

**Ovid MEDLINE(R) ALL 1946 to May 06, 2024**

|  | **#** | **Searches** | **Results** |
| --- | --- | --- | --- |
| **A1. PICC** | 1 | catheterization, central venous/ | 17254 |
|  | 2 | Central Venous Catheters/ | 3844 |
|  | 3 | (peripheral* or percutaneous*).ti,hw,kf. | 338413 |
|  | 4 | (1 or 2) and 3 | 2952 |
|  | 5 | ((peripheral* or percutaneous*) adj6 central venous adj3 (catheter* or line?)).ti,ab,kf. | 1215 |
|  | 6 | ((peripheral* or percutaneous*) adj3 central adj (catheter* or line?)).ti,ab,kf. | 2294 |
|  | 7 | PICC?.ti,ab,kf. | 2726 |
|  | 8 | or/4-7 | 5614 |
| **A2. PIVC** | 9 | exp catheterization, peripheral/ | 13485 |
|  | 10 | ((peripheral* or midline? or intravenous or intravascular) adj3 catheter*).ti,ab,kf. | 9532 |
|  | 11 | (peripheral* adj3 (cannula* or port? or intravenous or IV)).ti,ab,kf. | 2007 |
|  | 12 | Peripheral line?.ti,ab,kf. | 186 |
|  | 13 | (PIVC? or PIC line?).ti,ab,kf. | 422 |
|  | 14 | or/9-13 | 21861 |
| **A3. PAC** | 15 | Vascular Access Devices/ | 2453 |
|  | 16 | (artery or arteries or arterial*).ti,hw,kf. | 710368 |
|  | 17 | 15 and 16 | 900 |
|  | 18 | (arterial adj3 (catheter* or line? or Cannula*)).ti,ab,kf. | 9147 |
|  | 19 | art line?.ti,ab,kf. | 9 |
|  | 20 | 17 or 18 or 19 | 9954 |
| **A1 or A2** | 21 | 8 or 14 | 23832 |
| **all A: A1 or A2 or A3** | 22 | 8 or 14 or 20 | 32315 |
| **B1. general interventions** | 23 | exp *Catheters/st [Standards] | 528 |
|  | 24 | exp *Catheterization/st | 1446 |
|  | 25 | Patient Care Bundles/ | 1263 |
|  | 26 | bundle?.ti,ab,kf. | 79724 |
|  | 27 | (multimodal or multi-modal).ti,ab,kf. | 65387 |
|  | 28 | ((standard? or management or routine?) adj3 (practice? or care? or process* or strateg*)).ti,kf. | 38579 |
|  | 29 | or/23-28 | 185004 |
| **B1+A** | 30 | 22 and 29 | 665 |
| **B2. search terms for "Issues relevant to both PIVCs & PICCs"** | 31 | (insertion or inserting or securement or placement or fixation).ti,kf. | 125139 |
|  | 32 | exp antisepsis/ or exp sterilization/ | 38550 |
|  | 33 | Equipment Contamination/pc [Prevention & Control] | 4177 |
|  | 34 | Chlorhexidine/ | 9700 |
|  | 35 | Povidone-Iodine/ | 3403 |
|  | 36 | exp Anti-Infective Agents, Local/ | 268407 |
|  | 37 | ((steril* or nonsteril* or asepsis or aseptic or antisep* or disinfect* or alcohol or chlorhexidine or iodine) adj6 (skin? or insert* or clean* or prepar* or site?)).ti,ab,kf. | 20055 |
|  | 38 | exp Anesthetics, Local/ | 112904 |
|  | 39 | ((local* or topical* or insert* or site?) adj3 an?esth*).ti,ab,kf. | 58590 |
|  | 40 | exp Ultrasonography, Interventional/ | 32616 |
|  | 41 | ((ultrasound* or ultrason* or sonograph* or USGVA or POCUS) adj3 (guid* or directed or insert*)).ti,ab,kf. | 51886 |
|  | 42 | ((upper or lower) adj3 (limb? or arm? or leg? or extremit*)).ti,ab,kf. | 207985 |
|  | 43 | ((select* or choos* or choice) adj6 (site? or location?)).ti,ab,kf. | 46795 |
|  | 44 | (hand? or forearm? or cubital fossa or chelidon or grivet or elbow).ti,ab,kf. | 590955 |
|  | 45 | exp Silicones/ | 29979 |
|  | 46 | Polyurethanes/ | 10806 |
|  | 47 | (Silicon* or Polyurethan* or polytetrafluoroethylen*).ti,ab,kf. | 125058 |
|  | 48 | ((catheter* or Cannula* or tube? or tubing or line? or PICC? or PIC? or PIVC?) adj6 material?).ti,ab,kf. | 9987 |
|  | 49 | bandages/ or occlusive dressings/ | 23745 |
|  | 50 | (dressing? or gauze or occlusive or nonocclusive or transparent or semipermeable or semi-permeable or bandage?).ti,ab,kf. | 120914 |
|  | 51 | patient care team/ or nursing, team/ | 72336 |
|  | 52 | team?.ti,kf. | 40137 |
|  | 53 | ((catheter* or Cannula* or line? or PICC? or PIC? or PIVC? or insert* or access or special* or multidisciplinary or multi-disciplinary or care or trained) adj3 (team? or service)).ti,ab,kf. | 85718 |
|  | 54 | ((specialist? or nurse? or surgeon? or clinician? or technician?) adj3 led).ti,ab,kf. | 7956 |
|  | 55 | professional competence/ or clinical competence/ | 131655 |
|  | 56 | exp Catheterization/ed [Education] | 157 |
|  | 57 | education/ or exp curriculum/ | 121664 |
|  | 58 | (training or education or accreditation or certification or clinical competenc*).ti,kf. | 410779 |
|  | 59 | ((training or education or accreditation or certification or competenc*) adj6 (insert* or place* or secur* or fixa* or catheter* or Cannula* or PICC? or PIC? or PIVC?)).ti,ab,kf. | 13677 |
|  | 60 | exp Gloves, Protective/ | 5199 |
|  | 61 | Hygiene/ or Hand Hygiene/ | 19846 |
|  | 62 | glove?.ti,ab,kf. | 12886 |
|  | 63 | ((set? or pack* or kit?) adj6 (insertion or infusion or catheter* or Cannula* or PICC? or PIC? or PIVC?)).ti,ab,kf. | 3000 |
|  | 64 | (standard* adj6 (set? or pack* or kit? or equipment)).ti,ab,kf. | 24794 |
|  | 65 | (maintenance or patency).ti,kf. | 56970 |
|  | 66 | Infusions, Intravenous/is, nu, st [Instrumentation, Nursing, Standards] | 2070 |
|  | 67 | ((Continuous* or intermittent*) adj6 (infus* or medication? or administat* or therap*)).ti,ab,kf. | 59777 |
|  | 68 | Saline Solution/ | 1680 |
|  | 69 | exp Heparin/ | 69338 |
|  | 70 | (flush* or locking or lock).ti,ab,kf. | 52601 |
|  | 71 | ((saline or NaCl or heparin) adj6 (lock* or manag* or maintenance)).ti,ab,kf. | 2459 |
|  | 72 | replacement.ti,kf. | 95807 |
|  | 73 | ((chang* or replac*) adj6 (tube? or tubing or catheter* or Cannula* or PICC? or PIC? or PIVC?)).ti,ab,kf. | 10364 |
|  | 74 | Device Removal/ | 15155 |
|  | 75 | (removal or extraction or disconnection).ti,kf. | 181165 |
|  | 76 | ((remov* or extract* or disconnect*) adj6 (tube? or tubing or catheter* or cannula* or PICC? or PIC? or PIVC? or planned or unplanned or schedul*)).ti,ab,kf. | 25184 |
|  | 77 | (access* adj6 (protocol? or practice? or clean* or steril* or nonsteril* or asepsis or aseptic or antisep* or disinfect*)).ti,ab,kf. | 11501 |
|  | 78 | ((device? or catheter* or Cannula* or PICC? or PIC? or PIVC? or port?) adj6 (clean* or steril* or nonsteril* or asepsis or aseptic or antisep* or disinfect*)).ti,ab,kf. | 7124 |
|  | 79 | ((open or closed or integrated) adj6 (access or system? or port? or catheter* or device or cannula* or PICC? or PIC? or PIVC?)).ti,ab,kf. | 83173 |
|  | 80 | luer.ti,ab,kf. | 377 |
|  | 81 | or/31-80 | 3030936 |
| **B2+(A1 or A2)** | 82 | 21 and 81 | 9821 |
| **B3. search terms for "PICO questions relevant to PICCs only"** | 83 | lumen?.ti,ab,kf. | 82039 |
|  | 84 | ((single or dual or double or triple or multi*) adj3 (tube? or tubing or cathet* or cannula* or line?)).ti,ab,kf. | 39690 |
|  | 85 | exp Anti-Bacterial Agents/ad [Administration & Dosage] | 103985 |
|  | 86 | (antibiotic* or antibacterial* or antimicrobial* or anti-biotic* or anti-bacterial* or anti-microbial*).ti,ab,kf. | 689216 |
|  | 87 | exp Antineoplastic Agents/ad [Administration & Dosage] | 235143 |
|  | 88 | chemotherap*.ti,ab,kf. | 522830 |
|  | 89 | (antineoplas* or anticancer* or antitumo?r or anti-neoplas* or anti-cancer* or anti-tumo?r).ti,ab,kf. | 358908 |
|  | 90 | or/83-89 | 1770330 |
| **A1+B3** | 91 | 8 and 90 | 1252 |
| **B4. search terms for "PICO questions relevant to Peripheral Arterial Catheters"** | 92 | 31 or 32 or 33 or 34 or 35 or 36 or 37 or 49 or 50 or 56 or 57 or 58 or 59 or 66 or 67 or 77 or 78 | 1133257 |
| **A3+B4** | 93 | 20 and 92 | 858 |
| **B5. Interventions to prevent BSI** | 94 | *Catheter-Related Infections/pc or *bacterial infections/pc or exp *bacteremia/pc or *Cross Infection/pc or *Infection Control/ | 32890 |
|  | 95 | ((infection? or BSI) adj6 (prevent* or reduc* or decreas* or improv* or control or risk? or rate?)).ti,kf. | 65293 |
|  | 96 | 94 or 95 | 88113 |
| **A+B5** | 97 | 22 and 96 | 830 |
| **Total w/o filters** | 98 | 30 or 82 or 91 or 93 or 97 | 11580 |
| **humans** | 99 | limit 98 to "humans only (removes records about animals)" | 10982 |
| **date limit** | 100 | limit 99 to yr="1980 -Current" | 10896 |
| **SR-Filter** | 101 | Systematic Review.pt. | 259836 |
|  | 102 | review.pt. | 3318032 |
|  | 103 | (medline or medlars or embase or pubmed or cochrane or (scisearch or psychinfo or psycinfo) or (psychlit or psyclit) or cinahl or ((hand adj2 search$) or (manual$ adj2 search$)) or (electronic database$ or bibliographic database$ or computeri?ed database$ or online database$) or (pooling or pooled or mantel haenszel) or (peto or dersimonian or der simonian or fixed effect)).tw,sh. or (retraction of publication or retracted publication).pt. | 587202 |
|  | 104 | 102 and 103 | 246191 |
|  | 105 | meta-analysis.pt. or meta-analysis.sh. or (meta-analys$ or meta analys$ or metaanalys$).tw,sh. or (systematic$ adj5 review$).tw,sh. or (systematic$ adj5 overview$).tw,sh. or (quantitativ$ adj5 review$).tw,sh. or (quantitativ$ adj5 overview$).tw,sh. or (quantitativ$ adj5 synthesis$).tw,sh. or (methodologic$ adj5 review$).tw,sh. or (methodologic$ adj5 overview$).tw,sh. or (integrative research review$ or research integration).tw. | 535516 |
|  | 106 | 101 or 104 or 105 | 634161 |
| **SR-Results** | 107 | 99 and 106 | 402 |
| **SRs limited to last 5 years** | 108 | limit 107 to yr="2018 -Current" | 207 |
| **RCT-Filter** | 109 | exp randomized controlled trial/ or (random* or placebo).mp. [mp=title, book title, abstract, original title, name of substance word, subject heading word, floating sub-heading word, keyword heading word, organism supplementary concept word, protocol supplementary concept word, rare disease supplementary concept word, unique identifier, synonyms, population supplementary concept word, anatomy supplementary concept word] | 1824398 |
| **RCT-Results** | 110 | 100 and 109 | 1651 |
| **cNRS-Filter** | 111 | exp cohort studies/ or exp epidemiologic studies/ or exp clinical trial/ or exp evaluation studies as topic/ or exp statistics as topic/ | 6787386 |
|  | 112 | ((control and (study or group*)) or (time and factors) or cohort or program or comparative stud* or evaluation studies or survey* or follow-up* or ci).mp. | 8935723 |
|  | 113 | 111 or 112 | 11736911 |
|  | 114 | comment/ or editorial/ or exp review/ or meta analysis/ or consensus/ or exp guideline/ or hi.fs. or case report.mp. or (Systematic Review or review).pt. or ((review or meta-analy*) not (study or trial)).ti. | 5848990 |
|  | 115 | 113 not 114 | 10550086 |
| **cNRS-Results** | 116 | 100 and 115 | 5957 |
| **before-after studies filter** | 117 | Interrupted Time Series Analysis/ | 2049 |
|  | 118 | time series.mp. | 50272 |
|  | 119 | (case adj3 series).mp. | 116053 |
|  | 120 | (before adj6 after).ti,ab,kf. | 493855 |
|  | 121 | or/117-120 | 652815 |
| **B-A-Results** | 122 | 100 and 121 | 462 |
| **Total with filters** | 123 | 108 or 110 or 116 or 122 | 6506 |

**Embase.com**

| **No.** | **Query** | **Results** |
| --- | --- | --- |
| #1 | 'peripherally inserted central venous catheter'/exp/mj | 2114 |
| #2 | ((peripheral* OR percutaneous*) NEAR/6 'central venous' NEAR/3 (catheter* OR line$)):ti,ab,kw | 1791 |
| #3 | ((peripheral* OR percutaneous*) NEAR/3 central NEXT/1 (catheter* OR line$)):ti,ab,kw | 3885 |
| #4 | picc:ti,ab,kw OR piccs:ti,ab,kw | 4483 |
| #5 | #1 OR #2 OR #3 OR #4 | 6821 |
| #6 | 'catheterization'/mj | 14989 |
| #7 | peripheral*:ti,kw | 222288 |
| #8 | #6 AND #7 | 756 |
| #9 | ((peripheral* OR midline$ OR intravenous OR intravascular) NEAR/3 catheter*):ti,ab,kw | 14018 |
| #10 | (peripheral* NEAR/3 (cannula* OR port$ OR intravenous OR iv)):ti,ab,kw | 3226 |
| #11 | 'peripheral line$':ti,ab,kw | 352 |
| #12 | pivc$:ti,ab,kw OR 'pic line$':ti,ab,kw | 627 |
| #13 | #8 OR #9 OR #10 OR #11 OR #12 | 17211 |
| #14 | 'artery catheter'/mj | 838 |
| #15 | (arter* NEAR/3 (catheter* OR line$ OR cannula*)):ti,ab,kw | 30835 |
| #16 | 'art line$':ti,ab,kw | 23 |
| #17 | #14 OR #15 OR #16 | 31207 |
| #18 | #5 OR #13 | 19804 |
| #19 | #5 OR #13 OR #17 | 49919 |
| #20 | 'care bundle'/mj | 915 |
| #21 | bundle$:ti,ab,kw | 104253 |
| #22 | multimodal:ti,ab,kw OR 'multi modal':ti,ab,kw | 89387 |
| #23 | ((standard$ OR management OR routine$) NEAR/3 (practice$ OR care$ OR process* OR strateg*)):ti,kw | 52337 |
| #24 | #20 OR #21 OR #22 OR #23 | 244970 |
| #25 | #19 AND #24 | 760 |
| #26 | insertion:ti,kw OR inserting:ti,kw OR securement:ti,kw OR placement:ti,kw OR fixation:ti,kw OR 'cannulation'/mj OR 'catheter accessory'/exp/mj | 154808 |
| #27 | 'asepsis'/mj OR 'antisepsis'/mj OR 'disinfection'/mj OR 'hand disinfection'/mj OR 'instrument sterilization'/mj | 29099 |
| #28 | 'chlorhexidine'/mj OR 'povidone iodine'/mj OR 'topical antiinfective agent'/mj | 15609 |
| #29 | ((steril* OR nonsteril* OR asepsis OR aseptic OR antisep* OR disinfect* OR alcohol OR chlorhexidine OR iodine) NEAR/6 (skin$ OR insert* OR clean* OR prepar* OR site$)):ti,ab,kw | 28066 |
| #30 | 'local anesthetic agent'/exp/mj | 126829 |
| #31 | ((local* OR topical* OR insert* OR site$) NEAR/3 an$esth*):ti,ab,kw | 81390 |
| #32 | 'interventional ultrasonography'/mj OR 'endoscopic ultrasonography'/mj | 18141 |
| #33 | ((ultrasound* OR ultrason* OR sonograph* OR usgva OR pocus) NEAR/3 (guid* OR directed OR insert*)):ti,ab,kw | 82666 |
| #34 | ((upper OR lower) NEAR/3 (limb$ OR arm$ OR leg$ OR extremit*)):ti,ab,kw | 302885 |
| #35 | ((select* OR choos* OR choice) NEAR/6 (site$ OR location$)):ti,ab,kw | 54651 |
| #36 | hand$:ti,ab,kw OR forearm$:ti,ab,kw OR 'cubital fossa':ti,ab,kw OR chelidon:ti,ab,kw OR grivet:ti,ab,kw OR elbow:ti,ab,kw | 798994 |
| #37 | 'silicon'/mj OR 'polyurethan'/mj OR 'polytetrafluoroethylene'/mj | 29507 |
| #38 | silicon*:ti,ab,kw OR polyurethan*:ti,ab,kw OR polytetrafluoroethylen*:ti,ab,kw | 122678 |
| #39 | ((catheter* OR cannula* OR tube$ OR tubing OR line$ OR picc$ OR pic$ OR pivc$) NEAR/6 material$):ti,ab,kw | 18756 |
| #40 | 'bandage'/mj | 6042 |
| #41 | 'occlusive dressing'/mj OR 'wound dressing'/mj | 6459 |
| #42 | dressing$:ti,ab,kw OR gauze:ti,ab,kw OR occlusive:ti,ab,kw OR nonocclusive:ti,ab,kw OR transparent:ti,ab,kw OR semipermeable:ti,ab,kw OR 'semi permeable':ti,ab,kw OR bandage$:ti,ab,kw | 151273 |
| #43 | 'collaborative care team'/mj | 933 |
| #44 | 'patient care team'/mj OR 'collaborative care team'/mj OR 'team nursing'/mj | 1214 |
| #45 | team$:ti,kw | 52375 |
| #46 | ((catheter* OR cannula* OR line$ OR picc$ OR pic$ OR pivc$ OR insert* OR access OR special* OR multidisciplinary OR 'multi disciplinary' OR care OR trained) NEAR/3 (team$ OR service)):ti,ab,kw | 142730 |
| #47 | ((specialist$ OR nurse$ OR surgeon$ OR clinician$ OR technician$) NEAR/3 led):ti,ab,kw | 13065 |
| #48 | 'clinical competence'/mj OR 'nursing competence'/mj | 26023 |
| #49 | 'education'/mj OR 'medical education'/mj OR 'education program'/mj OR 'nursing education'/mj OR 'accreditation'/mj OR 'certification'/mj | 324110 |
| #50 | training:ti,kw OR education:ti,kw OR accreditation:ti,kw OR certification:ti,kw OR 'clinical competenc*':ti,kw | 492026 |
| #51 | ((training OR education OR accreditation OR certification OR competenc*) NEAR/6 (insert* OR place* OR secur* OR fixa* OR catheter* OR cannula* OR picc$ OR pic$ OR pivc$)):ti,ab,kw | 19074 |
| #52 | 'glove'/exp/mj OR 'protective glove'/exp/mj | 3391 |
| #53 | glove$:ti,ab,kw | 17193 |
| #54 | ((set$ OR pack* OR kit$) NEAR/6 (insertion OR infusion OR catheter* OR cannula* OR picc$ OR pic$ OR pivc$)):ti,ab,kw | 5149 |
| #55 | (standard* NEAR/6 (set$ OR pack* OR kit$ OR equipment)):ti,ab,kw | 36080 |
| #56 | maintenance:ti,kw OR patency:ti,kw | 78104 |
| #57 | 'continuous infusion'/mj | 1510 |
| #58 | ((continuous* OR intermittent*) NEAR/6 (infus* OR medication$ OR administat* OR therap*)):ti,ab,kw | 87015 |
| #59 | 'sodium chloride'/mj OR 'heparin'/mj | 94289 |
| #60 | flush*:ti,kw OR locking:ti,kw OR lock:ti,kw | 16118 |
| #61 | ((saline OR nacl OR heparin) NEAR/6 (lock* OR manag* OR maintenance)):ti,ab,kw | 3730 |
| #62 | replacement:ti,kw | 131819 |
| #63 | ((chang* OR replac*) NEAR/6 (tube$ OR tubing OR catheter* OR cannula* OR picc$ OR pic$ OR pivc$)):ti,ab,kw | 16192 |
| #64 | 'device removal'/mj OR 'catheter removal'/mj | 5715 |
| #65 | removal:ti,kw OR extraction:ti,kw OR disconnection:ti,kw | 219475 |
| #66 | ((remov* OR extract* OR disconnect*) NEAR/6 (tube$ OR tubing OR catheter* OR cannula* OR picc$ OR pic$ OR pivc$ OR planned OR unplanned OR schedul*)):ti,ab,kw | 41841 |
| #67 | (access* NEAR/6 (protocol$ OR practice$ OR clean* OR steril* OR nonsteril* OR asepsis OR aseptic OR antisep* OR disinfect*)):ti,ab,kw | 15506 |
| #68 | ((device$ OR catheter* OR cannula* OR picc$ OR pic$ OR pivc$ OR port$) NEAR/6 (clean* OR steril* OR nonsteril* OR asepsis OR aseptic OR antisep* OR disinfect*)):ti,ab,kw | 10815 |
| #69 | ((open OR closed OR integrated) NEAR/6 (access OR system$ OR port$ OR catheter* OR device OR cannula* OR picc$ OR pic$ OR pivc$)):ti,ab,kw | 107796 |
| #70 | luer:ti,ab,kw | 681 |
| #71 | #26 OR #27 OR #28 OR #29 OR #30 OR #31 OR #32 OR #33 OR #34 OR #35 OR #36 OR #37 OR #38 OR #39 OR #40 OR #41 OR #42 OR #43 OR #44 OR #45 OR #46 OR #47 OR #48 OR #49 OR #50 OR #51 OR #52 OR #53 OR #54 OR #55 OR #56 OR #57 OR #58 OR #59 OR #60 OR #61 OR #62 OR #63 OR #64 OR #65 OR #66 OR #67 OR #68 OR #69 OR #70 | 3438750 |
| #72 | #18 AND #71 | 9369 |
| #73 | 'double lumen catheter'/exp/mj OR 'single lumen catheter'/exp/mj OR 'triple lumen catheter'/exp/mj | 249 |
| #74 | lumen$:ti,ab,kw | 114687 |
| #75 | ((single OR dual OR double OR triple OR multi*) NEAR/3 (tube$ OR tubing OR cathet* OR cannula* OR line$)):ti,ab,kw | 57069 |
| #76 | 'antibiotic agent'/exp/mj/dd_ad OR 'antibiotic agent'/exp/mj/dd_iv | 47270 |
| #77 | antibiotic*:ti,ab,kw OR antibacterial*:ti,ab,kw OR antimicrobial*:ti,ab,kw OR 'anti biotic*':ti,ab,kw OR 'anti bacterial*':ti,ab,kw OR 'anti microbial*':ti,ab,kw | 907754 |
| #78 | 'antineoplastic agent'/exp/mj/dd_ad OR 'antineoplastic agent'/exp/mj/dd_iv | 107717 |
| #79 | chemotherap*:ti,ab,kw | 837545 |
| #80 | antineoplas*:ti,ab,kw OR anticancer*:ti,ab,kw OR antitumo$r:ti,ab,kw OR 'anti neoplas*':ti,ab,kw OR 'anti cancer*':ti,ab,kw OR 'anti tumo*':ti,ab,kw | 495627 |
| #81 | #73 OR #74 OR #75 OR #76 OR #77 OR #78 OR #79 OR #80 | 2332849 |
| #82 | #5 AND #81 | 2241 |
| #83 | #27 OR #28 OR #29 OR #40 OR #41 OR #42 OR #48 OR #49 OR #50 OR #51 OR #57 OR #58 OR #67 OR #68 | 1018036 |
| #84 | #17 AND #83 | 1720 |
| #85 | 'catheter infection'/exp/mj/dm_pc | 2127 |
| #86 | 'cross infection'/exp/mj/dm_pc | 7531 |
| #87 | 'infection control'/mj | 33333 |
| #88 | ((infection$ OR bsi) NEAR/6 (prevent* OR reduc* OR decreas* OR improv* OR control OR risk$ OR rate$)):ti,kw | 79411 |
| #89 | #85 OR #86 OR #87 OR #88 | 79001 |
| #90 | #19 AND #89 | 814 |
| #91 | #25 OR #72 OR #82 OR #84 OR #90 | 12586 |
| #92 | #91 NOT ('conference abstract'/it OR 'preprint'/it OR 'conference paper'/it OR 'conference review'/it) | 8075 |
| #93 | #92 AND [1980-2023]/py | 7727 |
| #94 | 'systematic review'/de OR 'meta analysis'/exp OR (((systematic OR 'state of the art' OR scoping OR literature OR umbrella) NEXT/1 (review* OR overview* OR assessment*)):ti,ab,kw) OR 'review* of reviews':ti,ab OR 'meta analy*':ti,ab,kw OR metaanaly*:ti,ab,kw OR (((systematic OR evidence) NEAR/1 assess*):ti,ab,kw) OR 'research evidence':ti,ab OR metasynthe*:ti,ab,kw OR 'meta synthe*':ti,ab,kw | 917572 |
| #95 | #92 AND #94 | 343 |
| #96 | #95 AND [2018-2023]/py | 168 |
| #97 | 'randomized controlled trial'/exp OR random*:ti,ab,kw OR placebo*:ti,ab,kw OR 'single blind*':ti,ab,kw OR 'double blind*':ti,ab,kw OR 'triple blind*':ti,ab,kw | 2322118 |
| #98 | #93 AND #97 | 1175 |
| #99 | 'cohort analysis'/exp OR 'controlled study'/exp OR 'evaluation study'/exp OR 'clinical trial'/exp OR 'clinical study'/de OR (control$:ti,ab,kw AND (study:ti,ab,kw OR group*:ti,ab,kw)) OR (time:ti,ab,kw AND factors:ti,ab,kw) OR cohort$:ti,ab,kw OR program:ti,ab,kw OR 'comparative stud*':ti,ab,kw OR 'evaluation stud*':ti,ab,kw OR survey*:ti,ab,kw OR 'follow up*':ti,ab,kw OR ci:ti,ab,kw | 16226157 |
| #100 | 'review'/exp OR 'meta analysis'/exp OR 'editorial'/exp OR 'practice guideline'/exp OR ((review:ti OR 'meta analy*':ti) NOT (study:ti OR trial:ti)) | 5089917 |
| #101 | #99 NOT #100 | 15025313 |
| #102 | #93 AND #101 | 3366 |
| #103 | 'epidemiology'/de | 271014 |
| #104 | 'time series':ti,ab,kw | 55804 |
| #105 | (case NEAR/3 series):ti,ab,kw | 164090 |
| #106 | (before NEAR/6 after):ti,ab,kw | 694443 |
| #107 | #103 OR #104 OR #105 OR #106 | 1170133 |
| #108 | #93 AND #107 NOT #100 | 332 |
| #109 | #95 OR #98 OR #102 OR #108 | 4075 |

**Cochrane Library**

Cochrane Database of Systematic Reviews Issue 4 of 12, April 2024

Cochrane Central Register of Controlled Trials Issue 4 of 12, April 2024

| **ID** | **Search** | **Hits** |
| --- | --- | --- |
| #1 | (([mh ^"catheterization, central venous"]) OR ([mh ^"Central Venous Catheters"])) AND ((peripheral* OR percutaneous*):ti,kw) | 223 |
| #2 | ((peripheral*:ti,ab,kw OR percutaneous*:ti,ab,kw) NEAR/6 "central venous":ti,ab,kw NEAR/3 (catheter*:ti,ab,kw OR line?:ti,ab,kw)) | 347 |
| #3 | ((peripheral*:ti,ab,kw OR percutaneous*:ti,ab,kw) NEAR/3 central:ti,ab,kw NEXT (catheter*:ti,ab,kw OR line?:ti,ab,kw)) | 465 |
| #4 | (PICC or PICCs):ti,ab,kw | 513 |
| #5 | {or #1-#4} | 858 |
| #6 | [mh "catheterization, peripheral"] | 1375 |
| #7 | ((peripheral*:ti,ab,kw OR midline?:ti,ab,kw OR intravenous:ti,ab,kw OR intravascular:ti,ab,kw) NEAR/3 catheter*:ti,ab,kw) | 3198 |
| #8 | (peripheral*:ti,ab,kw NEAR/3 (cannula*:ti,ab,kw OR port?:ti,ab,kw OR intravenous:ti,ab,kw OR IV:ti,ab,kw)) | 685 |
| #9 | (Peripheral NEXT line?):ti,ab,kw | 50 |
| #10 | (PIVC?:ti,ab,kw OR (PIC NEXT line?):ti,ab,kw) | 175 |
| #11 | {or #6-#10} | 3840 |
| #12 | ([mh ^"Vascular Access Devices"]) AND (artery:ti,kw OR arteries:ti,kw OR arterial*:ti,kw) | 120 |
| #13 | (arterial:ti,ab,kw NEAR/3 (catheter*:ti,ab,kw OR line?:ti,ab,kw OR Cannula*:ti,ab,kw)) | 1730 |
| #14 | (art NEXT line?):ti,ab,kw | 0 |
| #15 | {or #12-#14} | 1836 |
| #16 | #5 or #11 | 4089 |
| #17 | #5 or #11 or #15 | 5642 |
| #18 | [mh Catheters/st] | 54 |
| #19 | [mh Catheterization/st] | 168 |
| #20 | [mh ^"Patient Care Bundles"] | 75 |
| #21 | bundle?:ti,ab,kw | 3242 |
| #22 | (multimodal:ti,ab,kw OR multi-modal:ti,ab,kw) | 8852 |
| #23 | ((standard?:ti OR management:ti OR routine?:ti) NEAR/3 (practice?:ti OR care?:ti OR process*:ti OR strateg*:ti)) | 6254 |
| #24 | {or #18-#23} | 18447 |
| #25 | #17 and #24 | 162 |
| #26 | (insertion or inserting or securement or placement or fixation):ti,kw | 14394 |
| #27 | [mh antisepsis] OR [mh sterilization] | 836 |
| #28 | [mh ^"Equipment Contamination"] | 465 |
| #29 | [mh ^Chlorhexidine] | 2847 |
| #30 | [mh ^Povidone-Iodine] | 904 |
| #31 | [mh "Anti-Infective Agents, Local"] | 2696 |
| #32 | ((steril*:ti,ab,kw OR nonsteril*:ti,ab,kw OR asepsis:ti,ab,kw OR aseptic:ti,ab,kw OR antisep*:ti,ab,kw OR disinfect*:ti,ab,kw OR alcohol:ti,ab,kw OR chlorhexidine:ti,ab,kw OR iodine:ti,ab,kw) NEAR/6 (skin?:ti,ab,kw OR insert*:ti,ab,kw OR clean*:ti,ab,kw OR prepar*:ti,ab,kw OR site?:ti,ab,kw)) | 4052 |
| #33 | [mh "Anesthetics, Local"] | 10787 |
| #34 | ((local*:ti,ab,kw OR topical*:ti,ab,kw OR insert*:ti,ab,kw OR site?:ti,ab,kw) NEAR/3 an?esth*:ti,ab,kw) | 25138 |
| #35 | [mh "Ultrasonography, Interventional"] | 3531 |
| #36 | ((ultrasound*:ti,ab,kw OR ultrason*:ti,ab,kw OR sonograph*:ti,ab,kw OR USGVA:ti,ab,kw OR POCUS:ti,ab,kw) NEAR/3 (guid*:ti,ab,kw OR directed:ti,ab,kw OR insert*:ti,ab,kw)) | 15620 |
| #37 | ((upper:ti,ab,kw OR lower:ti,ab,kw) NEAR/3 (limb?:ti,ab,kw OR arm?:ti,ab,kw OR leg?:ti,ab,kw OR extremit*:ti,ab,kw)) | 41138 |
| #38 | ((select*:ti,ab,kw OR choos*:ti,ab,kw OR choice:ti,ab,kw) NEAR/6 (site?:ti,ab,kw OR location?:ti,ab,kw)) | 2485 |
| #39 | (hand?:ti,ab,kw OR forearm?:ti,ab,kw OR "cubital fossa":ti,ab,kw OR chelidon:ti,ab,kw OR grivet:ti,ab,kw OR elbow:ti,ab,kw) | 57610 |
| #40 | [mh Silicones] or [mh ^Polyurethanes] | 1796 |
| #41 | (Silicon*:ti,ab,kw OR Polyurethan*:ti,ab,kw OR polytetrafluoroethylen*:ti,ab,kw) | 6356 |
| #42 | ((catheter*:ti,ab,kw OR Cannula*:ti,ab,kw OR tube?:ti,ab,kw OR tubing:ti,ab,kw OR line?:ti,ab,kw OR PICC?:ti,ab,kw OR PIC?:ti,ab,kw OR PIVC?:ti,ab,kw) NEAR/6 material?:ti,ab,kw) | 852 |
| #43 | [mh ^bandages] OR [mh ^"occlusive dressings"] | 2773 |
| #44 | (dressing?:ti,ab,kw OR gauze:ti,ab,kw OR occlusive:ti,ab,kw OR nonocclusive:ti,ab,kw OR transparent:ti,ab,kw OR semipermeable:ti,ab,kw OR semi-permeable:ti,ab,kw OR bandage?:ti,ab,kw) | 18333 |
| #45 | [mh ^"patient care team"] OR [mh ^"nursing, team"] | 2270 |
| #46 | team?:ti,kw | 5098 |
| #47 | ((catheter*:ti,ab,kw OR Cannula*:ti,ab,kw OR line?:ti,ab,kw OR PICC?:ti,ab,kw OR PIC?:ti,ab,kw OR PIVC?:ti,ab,kw OR insert*:ti,ab,kw OR access:ti,ab,kw OR special*:ti,ab,kw OR multidisciplinary:ti,ab,kw OR multi-disciplinary:ti,ab,kw OR care:ti,ab,kw OR trained:ti,ab,kw) NEAR/3 (team?:ti,ab,kw OR service:ti,ab,kw)) | 11399 |
| #48 | ((specialist?:ti,ab,kw OR nurse?:ti,ab,kw OR surgeon?:ti,ab,kw OR clinician?:ti,ab,kw OR technician?:ti,ab,kw) NEAR/3 led:ti,ab,kw) | 2982 |
| #49 | [mh ^"professional competence"] OR [mh ^"clinical competence"] or [mh Catheterization/ed] or [mh ^education] OR [mh curriculum] | 7790 |
| #50 | (training or education or accreditation or certification or (clinical NEXT competenc*)):ti,kw | 124908 |
| #51 | ((training:ti,ab,kw OR education:ti,ab,kw OR accreditation:ti,ab,kw OR certification:ti,ab,kw OR competenc*:ti,ab,kw) NEAR/6 (insert*:ti,ab,kw OR place*:ti,ab,kw OR secur*:ti,ab,kw OR fixa*:ti,ab,kw OR catheter*:ti,ab,kw OR Cannula*:ti,ab,kw OR PICC?:ti,ab,kw OR PIC?:ti,ab,kw OR PIVC?:ti,ab,kw)) | 3603 |
| #52 | [mh "Gloves, Protective"] or [mh ^Hygiene] OR [mh ^"Hand Hygiene"] | 860 |
| #53 | glove?:ti,ab,kw | 1572 |
| #54 | ((set?:ti,ab,kw OR pack*:ti,ab,kw OR kit?:ti,ab,kw) NEAR/6 (insertion:ti,ab,kw OR infusion:ti,ab,kw OR catheter*:ti,ab,kw OR Cannula*:ti,ab,kw OR PICC?:ti,ab,kw OR PIC?:ti,ab,kw OR PIVC?:ti,ab,kw)) | 761 |
| #55 | (standard*:ti,ab,kw NEAR/6 (set?:ti,ab,kw OR pack*:ti,ab,kw OR kit?:ti,ab,kw OR equipment:ti,ab,kw)) | 2586 |
| #56 | (maintenance or patency):ti,kw | 23496 |
| #57 | [mh ^"Infusions, Intravenous"/is,nu,st] | 120 |
| #58 | ((Continuous*:ti,ab,kw OR intermittent*:ti,ab,kw) NEAR/6 (infus*:ti,ab,kw OR medication?:ti,ab,kw OR administat*:ti,ab,kw OR therap*:ti,ab,kw)) | 21158 |
| #59 | [mh ^"Saline Solution"] OR [mh Heparin] | 6691 |
| #60 | (flush*:ti,kw OR locking:ti,kw OR lock:ti,kw) | 5003 |
| #61 | ((saline:ti,ab,kw OR NaCl:ti,ab,kw OR heparin:ti,ab,kw) NEAR/6 (lock*:ti,ab,kw OR manag*:ti,ab,kw OR maintenance:ti,ab,kw)) | 885 |
| #62 | replacement:ti,kw | 23105 |
| #63 | ((chang*:ti,ab,kw OR replac*:ti,ab,kw) NEAR/6 (tube?:ti,ab,kw OR tubing:ti,ab,kw OR catheter*:ti,ab,kw OR Cannula*:ti,ab,kw OR PICC?:ti,ab,kw OR PIC?:ti,ab,kw OR PIVC?:ti,ab,kw)) | 1552 |
| #64 | [mh ^"Device Removal"] | 571 |
| #65 | (removal or extraction or disconnection):ti,kw | 17078 |
| #66 | ((remov*:ti,ab,kw OR extract*:ti,ab,kw OR disconnect*:ti,ab,kw) NEAR/6 (tube?:ti,ab,kw OR tubing:ti,ab,kw OR catheter*:ti,ab,kw OR cannula*:ti,ab,kw OR PICC?:ti,ab,kw OR PIC?:ti,ab,kw OR PIVC?:ti,ab,kw OR planned:ti,ab,kw OR unplanned:ti,ab,kw OR schedul*:ti,ab,kw)) | 5466 |
| #67 | (access*:ti,ab,kw NEAR/6 (protocol?:ti,ab,kw OR practice?:ti,ab,kw OR clean*:ti,ab,kw OR steril*:ti,ab,kw OR nonsteril*:ti,ab,kw OR asepsis:ti,ab,kw OR aseptic:ti,ab,kw OR antisep*:ti,ab,kw OR disinfect*:ti,ab,kw)) | 1309 |
| #68 | ((device?:ti,ab,kw OR catheter*:ti,ab,kw OR Cannula*:ti,ab,kw OR PICC?:ti,ab,kw OR PIC?:ti,ab,kw OR PIVC?:ti,ab,kw OR port?:ti,ab,kw) NEAR/6 (clean*:ti,ab,kw OR steril*:ti,ab,kw OR nonsteril*:ti,ab,kw OR asepsis:ti,ab,kw OR aseptic:ti,ab,kw OR antisep*:ti,ab,kw OR disinfect*:ti,ab,kw)) | 1500 |
| #69 | ((open:ti,ab,kw OR closed:ti,ab,kw OR integrated:ti,ab,kw) NEAR/6 (access:ti,ab,kw OR system?:ti,ab,kw OR port?:ti,ab,kw OR catheter*:ti,ab,kw OR device:ti,ab,kw OR cannula*:ti,ab,kw OR PICC?:ti,ab,kw OR PIC?:ti,ab,kw OR PIVC?:ti,ab,kw)) | 6259 |
| #70 | luer:ti,ab,kw | 71 |
| #71 | {or #26-#70} | 386190 |
| #72 | #16 and #71 | 2590 |
| #73 | lumen?:ti,ab,kw | 4978 |
| #74 | ((single:ti,ab,kw OR dual:ti,ab,kw OR double:ti,ab,kw OR triple:ti,ab,kw OR multi*:ti,ab,kw) NEAR/3 (tube?:ti,ab,kw OR tubing:ti,ab,kw OR cathet*:ti,ab,kw OR cannula*:ti,ab,kw OR line?:ti,ab,kw)) | 3733 |
| #75 | [mh "Anti-Bacterial Agents"/ad] | 5456 |
| #76 | (antibiotic*:ti,ab,kw OR antibacterial*:ti,ab,kw OR antimicrobial*:ti,ab,kw OR anti-biotic*:ti,ab,kw OR anti-bacterial*:ti,ab,kw OR anti-microbial*:ti,ab,kw) | 51889 |
| #77 | [mh "Antineoplastic Agents"/ad] | 6853 |
| #78 | chemotherap*:ti,ab,kw | 96580 |
| #79 | (antineoplas*:ti,ab,kw OR anticancer*:ti,ab,kw OR antitumo?r:ti,ab,kw OR anti-neoplas*:ti,ab,kw OR anti-cancer*:ti,ab,kw OR anti-tumo?r:ti,ab,kw) | 46787 |
| #80 | {or #74-#79} | 163318 |
| #81 | #5 and #80 | 236 |
| #82 | #27 or #28 or #29 or #30 or #31 #32 or #49 or #50 or #51 or #43 or #44 or #57 or #58 or #67 or #68 | 171374 |
| #83 | #15 and #82 | 289 |
| #84 | [mh ^"Catheter-Related Infections"] OR [mh ^"bacterial infections"] OR [mh bacteremia/pc] OR [mh ^"Cross Infection"] OR [mh ^"Infection Control"] | 6533 |
| #85 | ((infection? or BSI) NEAR/6 (prevent* or reduc* or decreas* or improv* or control or risk? or rate?)):ti,kw | 25765 |
| #86 | #84 or #85 | 29062 |
| #87 | #17 and #86 | 338 |
| #88 | #25 or #72 or #81 or #83 or #87 | 2981 |
| #89 | #88 with Cochrane Library publication date Between Jan 2018 and Mar 2023, in Cochrane Reviews | 7 |
| #90 | (clinicaltrials or trialsearch or ANZCTR or ensaiosclinicos or chictr or cris or ctri or registroclinico or clinicaltrialsregister or DRKS or IRCT or rctportal or JapicCTI or JMACCT or jRCT or JPRN or UMIN or trialregister or PACTR or REPEC or SLCTR or TCTR):so | 509264 |
| #91 | Conference proceeding:pt or abstract:so | 242922 |
| #92 | #88 not (#90 or #91) with Publication Year from 1980 to 2024, in Trials | 1820 |
| #93 | #89 or #92 | 1827 |

**WHO Global Index Medicus (**[**https://pesquisa.bvsalud.org/gim/**](https://pesquisa.bvsalud.org/gim/)**)**

3/16/2023

| **Search** | **Result** |
| --- | --- |
| ((peripheral* OR intravenous OR intravascular OR artery OR arteries OR arterial* OR percutaneous) AND (catheter* OR cannula*)) OR picc OR piccs OR pivc OR pivcs OR "peripheral line" OR "peripheral lines" OR "pic line" OR "pic lines" OR "art line" OR "art lines" | 124 |

5/07/2024

| **Search** | **Result** |
| --- | --- |
| ((peripheral* OR intravenous OR intravascular OR artery OR arteries OR arterial* OR percutaneous) AND (catheter* OR cannula*)) OR picc OR piccs OR pivc OR pivcs OR "peripheral line" OR "peripheral lines" OR "pic line" OR "pic lines" OR "art line" OR "art lines" AND (year_cluster:[2023 TO 2024]) | 204 |

**CINAHL (Ebsco)**

| **#** | **Query** | **Limiters/Expanders** | **Results** |
| --- | --- | --- | --- |
| S1 | (MH "Catheterization, Peripheral Central Venous") | Expanders - Apply equivalent subjects | 761 |
| S2 | (((TI peripheral* OR AB peripheral*) OR (TI percutaneous* OR AB percutaneous*)) N6 (TI "central venous" OR AB "central venous") N3 ((TI catheter* OR AB catheter*) OR (TI line# OR AB line#))) | Search modes - Find all my search terms | 491 |
| S3 | (((TI peripheral* OR AB peripheral*) OR (TI percutaneous* OR AB percutaneous*)) N3 (TI central OR AB central) W1 ((TI catheter* OR AB catheter*) OR (TI line# OR AB line#))) | Search modes - Find all my search terms | 1,710 |
| S4 | (TI (PICC OR PICCs) OR AB (PICC OR PICCs)) | Search modes - Find all my search terms | 1,492 |
| S5 | S1 OR S2 OR S3 OR S4 | Search modes - Find all my search terms | 2,775 |
| S6 | (MH "Catheterization, Peripheral") | Expanders - Apply equivalent subjects | 4,019 |
| S7 | (((TI peripheral* OR AB peripheral*) OR (TI midline# OR AB midline#) OR (TI intravenous OR AB intravenous) OR (TI intravascular OR AB intravascular)) N3 (TI catheter* OR AB catheter*)) | Search modes - Find all my search terms | 4,107 |
| S8 | ((TI peripheral* OR AB peripheral*) N3 ((TI cannula* OR AB cannula*) OR (TI port# OR AB port#) OR (TI intravenous OR AB intravenous) OR (TI IV OR AB IV))) | Search modes - Find all my search terms | 833 |
| S9 | (TI "Peripheral line#" OR AB "Peripheral line#") | Search modes - Find all my search terms | 68 |
| S10 | ((TI PIVC# OR AB PIVC#) OR (TI "PIC line#" OR AB "PIC line#")) | Search modes - Find all my search terms | 217 |
| S11 | S6 OR S7 OR S8 OR S9 OR S10 | Search modes - Find all my search terms | 7,403 |
| S12 | (MH "Arterial Catheters") | Expanders - Apply equivalent subjects | 305 |
| S13 | ((TI arter* OR AB arter*) N3 ((TI catheter* OR AB catheter*) OR (TI line# OR AB line#) OR (TI Cannula* OR AB Cannula*))) | Search modes - Find all my search terms | 4,909 |
| S14 | (TI "art line#" OR AB "art line#") | Search modes - Find all my search terms | 2 |
| S15 | S12 OR S13 OR S14 | Search modes - Find all my search terms | 5,045 |
| S16 | S5 OR S11 | Search modes - Find all my search terms | 8,273 |
| S17 | S5 OR S11 OR S15 | Search modes - Find all my search terms | 12,757 |
| S18 | (MM "Catheters+/ST/NU/OG") OR (MM "Catheterization/ST/OG/NU") | Expanders - Apply equivalent subjects | 487 |
| S19 | (MM "Catheter Care") OR (MM "Catheter Care, Vascular+") | Expanders - Apply equivalent subjects | 1,801 |
| S20 | (TI bundle# OR AB bundle#) | Search modes - Find all my search terms | 13,336 |
| S21 | ((TI multimodal OR AB multimodal) OR (TI multi-modal OR AB multi-modal)) | Search modes - Find all my search terms | 13,299 |
| S22 | (((TI standard#) OR (TI management) OR (TI routine#)) N3 ((TI practice#) OR (TI care#) OR (TI process*) OR (TI strateg*))) | Search modes - Find all my search terms | 25,742 |
| S23 | S18 OR S19 OR S20 OR S21 OR S22 | Search modes - Find all my search terms | 54,139 |
| S24 | S17 AND S23 | Search modes - Find all my search terms | 850 |
| S25 | ((TI insertion) OR (TI inserting) OR (TI securement) OR (TI placement) OR (TI fixation)) | Search modes - Find all my search terms | 33,258 |
| S26 | (MH "Sterilization and Disinfection") OR (MH "Asepsis") OR (MH "Equipment Contamination/PC") OR (MH "Antiinfective Agents, Local+") | Expanders - Apply equivalent subjects | 25,165 |
| S27 | (((TI steril* OR AB steril*) OR (TI nonsteril* OR AB nonsteril*) OR (TI asepsis OR AB asepsis) OR (TI aseptic OR AB aseptic) OR (TI antisep* OR AB antisep*) OR (TI disinfect* OR AB disinfect*) OR (TI alcohol OR AB alcohol) OR (TI chlorhexidine OR AB chlorhexidine) OR (TI iodine OR AB iodine)) N6 ((TI skin# OR AB skin#) OR (TI insert* OR AB insert*) OR (TI clean* OR AB clean*) OR (TI prepar* OR AB prepar*) OR (TI site# OR AB site#))) | Search modes - Find all my search terms | 5,055 |
| S28 | (MH "Anesthetics, Local+") | Expanders - Apply equivalent subjects | 19,496 |
| S29 | (((TI local* OR AB local*) OR (TI topical* OR AB topical*) OR (TI insert* OR AB insert*) OR (TI site# OR AB site#)) N3 (TI an#esth* OR AB an#esth*)) | Search modes - Find all my search terms | 10,780 |
| S30 | (MH "Ultrasonography+") | Expanders - Apply equivalent subjects | 116,626 |
| S31 | (((TI ultrasound* OR AB ultrasound*) OR (TI ultrason* OR AB ultrason*) OR (TI sonograph* OR AB sonograph*) OR (TI USGVA OR AB USGVA) OR (TI POCUS OR AB POCUS)) N3 ((TI guid* OR AB guid*) OR (TI directed OR AB directed) OR (TI insert* OR AB insert*))) | Search modes - Find all my search terms | 14,876 |
| S32 | (MH "Insertion Site Selection") | Expanders - Apply equivalent subjects | 64 |
| S33 | (((TI upper OR AB upper) OR (TI lower OR AB lower)) N3 ((TI limb# OR AB limb#) OR (TI arm# OR AB arm#) OR (TI leg# OR AB leg#) OR (TI extremit* OR AB extremit*))) | Search modes - Find all my search terms | 63,693 |
| S34 | (((TI select* OR AB select*) OR (TI choos* OR AB choos*) OR (TI choice OR AB choice)) N6 ((TI site# OR AB site#) OR (TI location# OR AB location#))) | Search modes - Find all my search terms | 4,715 |
| S35 | ((TI hand# OR AB hand#) OR (TI forearm# OR AB forearm#) OR (TI "cubital fossa" OR AB "cubital fossa") OR (TI chelidon OR AB chelidon) OR (TI grivet OR AB grivet) OR (TI elbow OR AB elbow)) | Search modes - Find all my search terms | 104,522 |
| S36 | (MH "Silicones+") OR (MH "Polyurethanes") | Expanders - Apply equivalent subjects | 3,843 |
| S37 | ((TI Silicon* OR AB Silicon*) OR (TI Polyurethan* OR AB Polyurethan*) OR (TI polytetrafluoroethylen* OR AB polytetrafluoroethylen*)) | Search modes - Find all my search terms | 7,135 |
| S38 | (((TI catheter* OR AB catheter*) OR (TI Cannula* OR AB Cannula*) OR (TI tube# OR AB tube#) OR (TI tubing OR AB tubing) OR (TI line# OR AB line#) OR (TI PICC# OR AB PICC#) OR (TI PIC# OR AB PIC#) OR (TI PIVC# OR AB PIVC#)) N6 (TI material# OR AB material#)) | Search modes - Find all my search terms | 1,487 |
| S39 | (MH "Transparent Dressings") OR (MH "Occlusive Dressings") OR (MH "Bandages and Dressings") | Expanders - Apply equivalent subjects | 11,812 |
| S40 | ((TI dressing# OR AB dressing#) OR (TI gauze OR AB gauze) OR (TI occlusive OR AB occlusive) OR (TI nonocclusive OR AB nonocclusive) OR (TI transparent OR AB transparent) OR (TI semipermeable OR AB semipermeable) OR (TI semi-permeable OR AB semi-permeable) OR (TI bandage# OR AB bandage#)) | Search modes - Find all my search terms | 22,963 |
| S41 | (MH "Multidisciplinary Care Team") OR (MH "Team Nursing") | Expanders - Apply equivalent subjects | 53,122 |
| S42 | (TI team#) | Search modes - Find all my search terms | 26,244 |
| S43 | (((TI catheter* OR AB catheter*) OR (TI Cannula* OR AB Cannula*) OR (TI line# OR AB line#) OR (TI PICC# OR AB PICC#) OR (TI PIC# OR AB PIC#) OR (TI PIVC# OR AB PIVC#) OR (TI insert* OR AB insert*) OR (TI access OR AB access) OR (TI special* OR AB special*) OR (TI multidisciplinary OR AB multidisciplinary) OR (TI multi-disciplinary OR AB multi-disciplinary) OR (TI care OR AB care) OR (TI trained OR AB trained)) N3 ((TI team# OR AB team#) OR (TI service OR AB service))) | Search modes - Find all my search terms | 99,248 |
| S44 | (((TI specialist# OR AB specialist#) OR (TI nurse# OR AB nurse#) OR (TI surgeon# OR AB surgeon#) OR (TI clinician# OR AB clinician#) OR (TI technician# OR AB technician#)) N3 (TI led OR AB led)) | Search modes - Find all my search terms | 7,500 |
| S45 | (MH "Credentialing Examinations") OR (MH "Professional Competence") OR (MH "Clinical Competence") OR (MH "Accreditation+") OR (MH "Certification") OR (MH "Education") OR (MH "Curriculum") OR (MH "Education, Clinical") OR (MH "Catheterization+/ED") | Expanders - Apply equivalent subjects | 149,938 |
| S46 | ((TI training) OR (TI education) OR (TI accreditation) OR (TI certification) OR (TI "clinical competenc*")) | Search modes - Find all my search terms | 186,446 |
| S47 | (((TI training OR AB training) OR (TI education OR AB education) OR (TI accreditation OR AB accreditation) OR (TI certification OR AB certification) OR (TI competenc* OR AB competenc*)) N6 ((TI insert* OR AB insert*) OR (TI place* OR AB place*) OR (TI secur* OR AB secur*) OR (TI fixa* OR AB fixa*) OR (TI catheter* OR AB catheter*) OR (TI Cannula* OR AB Cannula*) OR (TI PICC# OR AB PICC#) OR (TI PIC# OR AB PIC#) OR (TI PIVC# OR AB PIVC#))) | Search modes - Find all my search terms | 7,765 |
| S48 | (MH "Gloves") | Expanders - Apply equivalent subjects | 3,247 |
| S49 | (TI glove# OR AB glove#) | Search modes - Find all my search terms | 3,923 |
| S50 | (((TI set# OR AB set#) OR (TI pack* OR AB pack*) OR (TI kit# OR AB kit#)) N6 ((TI insertion OR AB insertion) OR (TI infusion OR AB infusion) OR (TI catheter* OR AB catheter*) OR (TI Cannula* OR AB Cannula*) OR (TI PICC# OR AB PICC#) OR (TI PIC# OR AB PIC#) OR (TI PIVC# OR AB PIVC#))) | Search modes - Find all my search terms | 805 |
| S51 | ((TI standard* OR AB standard*) N6 ((TI set# OR AB set#) OR (TI pack* OR AB pack*) OR (TI kit# OR AB kit#) OR (TI equipment OR AB equipment))) | Search modes - Find all my search terms | 6,715 |
| S52 | ((TI maintenance) OR (TI patency)) | Search modes - Find all my search terms | 12,333 |
| S53 | (MH "Infusions, Intravenous/ST") OR (MH "Infusion Devices, Intermittent") | Expanders - Apply equivalent subjects | 234 |
| S54 | (((TI Continuous* OR AB Continuous*) OR (TI intermittent* OR AB intermittent*)) N6 ((TI infus* OR AB infus*) OR (TI medication# OR AB medication#) OR (TI administat* OR AB administat*) OR (TI therap* OR AB therap*))) | Search modes - Find all my search terms | 11,902 |
| S55 | (MH "Catheter Irrigation, Vascular") | Expanders - Apply equivalent subjects | 322 |
| S56 | (MH "Saline Solution, Hypertonic") OR (MH "Normal Saline") OR (MH "Heparin+") | Expanders - Apply equivalent subjects | 14,260 |
| S57 | ((TI flush*) OR (TI locking) OR (TI lock)) | Search modes - Find all my search terms | 3,402 |
| S58 | (((TI saline OR AB saline) OR (TI NaCl OR AB NaCl) OR (TI heparin OR AB heparin)) N6 ((TI lock* OR AB lock*) OR (TI manag* OR AB manag*) OR (TI maintenance OR AB maintenance))) | Search modes - Find all my search terms | 738 |
| S59 | (TI replacement) | Search modes - Find all my search terms | 23,799 |
| S60 | (((TI chang* OR AB chang*) OR (TI replac* OR AB replac*)) N6 ((TI tube# OR AB tube#) OR (TI tubing OR AB tubing) OR (TI catheter* OR AB catheter*) OR (TI Cannula* OR AB Cannula*) OR (TI PICC# OR AB PICC#) OR (TI PIC# OR AB PIC#) OR (TI PIVC# OR AB PIVC#))) | Search modes - Find all my search terms | 2,643 |
| S61 | (MH "Device Removal") OR (MH "Catheter Removal") | Expanders - Apply equivalent subjects | 6,036 |
| S62 | ((TI removal) OR (TI extraction) OR (TI disconnection)) | Search modes - Find all my search terms | 15,219 |
| S63 | (((TI remov* OR AB remov*) OR (TI extract* OR AB extract*) OR (TI disconnect* OR AB disconnect*)) N6 ((TI tube# OR AB tube#) OR (TI tubing OR AB tubing) OR (TI catheter* OR AB catheter*) OR (TI cannula* OR AB cannula*) OR (TI PICC# OR AB PICC#) OR (TI PIC# OR AB PIC#) OR (TI PIVC# OR AB PIVC#) OR (TI planned OR AB planned) OR (TI unplanned OR AB unplanned) OR (TI schedul* OR AB schedul*))) | Search modes - Find all my search terms | 5,299 |
| S64 | ((TI access* OR AB access*) N6 ((TI protocol# OR AB protocol#) OR (TI practice# OR AB practice#) OR (TI clean* OR AB clean*) OR (TI steril* OR AB steril*) OR (TI nonsteril* OR AB nonsteril*) OR (TI asepsis OR AB asepsis) OR (TI aseptic OR AB aseptic) OR (TI antisep* OR AB antisep*) OR (TI disinfect* OR AB disinfect*))) | Search modes - Find all my search terms | 5,907 |
| S65 | (((TI device# OR AB device#) OR (TI catheter* OR AB catheter*) OR (TI Cannula* OR AB Cannula*) OR (TI PICC# OR AB PICC#) OR (TI PIC# OR AB PIC#) OR (TI PIVC# OR AB PIVC#) OR (TI port# OR AB port#)) N6 ((TI clean* OR AB clean*) OR (TI steril* OR AB steril*) OR (TI nonsteril* OR AB nonsteril*) OR (TI asepsis OR AB asepsis) OR (TI aseptic OR AB aseptic) OR (TI antisep* OR AB antisep*) OR (TI disinfect* OR AB disinfect*))) | Search modes - Find all my search terms | 1,974 |
| S66 | (((TI open OR AB open) OR (TI closed OR AB closed) OR (TI integrated OR AB integrated)) N6 ((TI access OR AB access) OR (TI system# OR AB system#) OR (TI port# OR AB port#) OR (TI catheter* OR AB catheter*) OR (TI device OR AB device) OR (TI cannula* OR AB cannula*) OR (TI PICC# OR AB PICC#) OR (TI PIC# OR AB PIC#) OR (TI PIVC# OR AB PIVC#))) | Search modes - Find all my search terms | 17,910 |
| S67 | (TI luer OR AB luer) | Search modes - Find all my search terms | 141 |
| S68 | S25 OR S26 OR S27 OR S28 OR S29 OR S30 OR S31 OR S32 OR S33 OR S34 OR S35 OR S36 OR S37 OR S38 OR S39 OR S40 OR S41 OR S42 OR S43 OR S44 OR S45 OR S46 OR S47 OR S48 OR S49 OR S50 OR S51 OR S52 OR S53 OR S54 OR S55 OR S56 OR S57 OR S58 OR S59 OR S60 OR S61 OR S62 OR S63 OR S64 OR S65 OR S66 OR S67 | Search modes - Find all my search terms | 940,045 |
| S69 | S16 AND S68 | Search modes - Find all my search terms | 4,352 |
| S70 | (TI lumen# OR AB lumen#) | Search modes - Find all my search terms | 8,257 |
| S71 | (((TI single OR AB single) OR (TI dual OR AB dual) OR (TI double OR AB double) OR (TI triple OR AB triple) OR (TI multi* OR AB multi*)) N3 ((TI tube# OR AB tube#) OR (TI tubing OR AB tubing) OR (TI cathet* OR AB cathet*) OR (TI cannula* OR AB cannula*) OR (TI line# OR AB line#))) | Search modes - Find all my search terms | 6,093 |
| S72 | (MH "Antibiotics+/AD") | Expanders - Apply equivalent subjects | 24,778 |
| S73 | ((TI antibiotic* OR AB antibiotic*) OR (TI antibacterial* OR AB antibacterial*) OR (TI antimicrobial* OR AB antimicrobial*) OR (TI anti-biotic* OR AB anti-biotic*) OR (TI anti-bacterial* OR AB anti-bacterial*) OR (TI anti-microbial* OR AB anti-microbial*)) | Search modes - Find all my search terms | 83,388 |
| S74 | (MH "Antineoplastic Agents+/AD") | Expanders - Apply equivalent subjects | 36,353 |
| S75 | (TI chemotherap* OR AB chemotherap*) | Search modes - Find all my search terms | 89,082 |
| S76 | ((TI antineoplas* OR AB antineoplas*) OR (TI anticancer* OR AB anticancer*) OR (TI antitumo#r OR AB antitumo#r) OR (TI anti-neoplas* OR AB anti-neoplas*) OR (TI anti-cancer* OR AB anti-cancer*) OR (TI anti-tumo#r OR AB anti-tumo#r)) | Search modes - Find all my search terms | 32,501 |
| S77 | S70 OR S71 OR S72 OR S73 OR S74 OR S75 OR S76 | Search modes - Find all my search terms | 241,770 |
| S78 | S5 AND S77 | Search modes - Find all my search terms | 483 |
| S79 | S26 OR S27 OR S39 OR S40 OR S45 OR S46 OR S47 OR S53 OR S54 OR S64 OR S65 | Search modes - Find all my search terms | 379,754 |
| S80 | S15 AND S79 | Search modes - Find all my search terms | 324 |
| S81 | (MM "Catheter-Related Infections") OR (MM "Catheter-Related Bloodstream Infections/PC") | Expanders - Apply equivalent subjects | 3,710 |
| S82 | (MM "Bacteremia/PC") OR (MM "Bacterial Infections+/PC") OR (MM "Cross Infection/PC") OR (MM "Infection Control") | Expanders - Apply equivalent subjects | 36,767 |
| S83 | (((TI infection#) OR (TI BSI)) N6 ((TI prevent*) OR (TI reduc*) OR (TI decreas*) OR (TI improv*) OR (TI control) OR (TI rate#) OR (TI risk#))) | Search modes - Find all my search terms | 25,810 |
| S84 | S81 OR S82 OR S83 | Search modes - Find all my search terms | 56,811 |
| S85 | S17 AND S84 | Search modes - Find all my search terms | 860 |
| S86 | S24 OR S69 OR S78 OR S80 OR S85 | Search modes - Find all my search terms | 5,372 |
| S87 | (MH animals+ OR MH ("animal studies") OR TI ("animal model*")) NOT MH (human) | Search modes - Find all my search terms | 212,475 |
| S88 | S86 NOT S87 | Search modes - Find all my search terms | 5,296 |
| S89 | (TI (systematic* N3 review*)) OR (AB (systematic* N3 review*)) OR (TI (systematic* N3 bibliographic*)) OR (AB (systematic* N3 bibliographic*)) OR (TI (systematic* N3 literature)) OR (AB (systematic* N3 literature)) OR (TI (comprehensive* N3 literature)) OR (AB (comprehensive* N3 literature)) OR (TI (comprehensive* N3 bibliographic*)) OR (AB (comprehensive* N3 bibliographic*)) OR (TI (integrative N3 review)) OR (AB (integrative N3 review)) OR (JN "Cochrane Database of Systematic Reviews") OR (TI (information N2 synthesis)) OR (TI (data N2 synthesis)) OR (AB (information N2 synthesis)) OR (AB (data N2 synthesis)) OR (TI (data N2 extract*)) OR (AB (data N2 extract*)) OR (TI (medline OR pubmed OR psyclit OR cinahl OR (psycinfo NOT"psycinfo database") OR "web of science" OR scopus OR embase)) OR (AB (medline OR pubmed OR psyclit OR cinahl OR (psycinfo NOT"psycinfo database") OR "web of science" OR scopus OR embase)) OR (MH "Systematic Review") OR (MH "Meta Analysis") OR (TI (meta-analy* OR metaanaly*)) OR (AB (meta-analy* OR metaanaly*)) OR (ZT "systematic review") OR (ZT "meta analysis") | Search modes - Boolean/Phrase | 313,421 |
| S90 | S88 AND S89 | Limiters - Publication Date: 20180101-20240508 | 128 |
| S91 | MH randomized controlled trials OR MH double‐blind studies OR MH single‐blind studies OR MH random assignment OR MH pretest‐posttest design OR MH cluster sample OR TI (randomised OR randomized) OR AB (random*) OR TI (trial) OR MH (sample size) AND AB (assigned OR allocated OR control) OR MH (placebos) OR PT (randomized controlled trial) OR AB (control W5 group) OR MH (crossover design) OR MH (comparative studies) OR AB (cluster W3 RCT) | Search modes - Boolean/Phrase | 1,043,131 |
| S92 | S88 AND S91 | Limiters - Publication Date: 20180101-20240508 | 1,286 |
| S93 | (cohort OR (control AND study) OR (control AND group*) OR program OR comparative stud* OR evaluation studies OR survey* OR follow‐up* OR time factors OR ci OR (MH "Comparative Studies") OR (MH "Clinical Research") OR (MH "Clinical Trials+") OR (MH "Nonrandomized Trials") OR (MH "Nonexperimental Studies+") OR (MH "Quasi-Experimental Studies+")) NOT ((ZT "review") OR (MH "Literature Review+") OR (MH "Case Studies") OR (MH "Meta Analysis") OR (MH "Practice Guidelines") OR (MH "History+")) | Search modes - Boolean/Phrase | 2,558,591 |
| S94 | S88 AND S93 | Limiters - Publication Date: 20180101-20240508 | 2,535 |
| S95 | (MH "Time Series") OR (MH "Multiple Time Series") OR (MH "Quasi-Experimental Studies") OR (MH "Interrupted Time Series Analysis") OR (MH "Controlled Before-After Studies") | Expanders - Apply equivalent subjects | 22,055 |
| S96 | ("time series") OR ((case N3 series)) OR (((TI before OR AB before) N6 (TI after OR AB after))) | Search modes - Find all my search terms | 156,225 |
| S97 | S88 AND (S95 OR S96) | Limiters - Publication Date: 20180101-20240508 | 250 |
| S98 | S90 OR S92 OR S94 OR S97 | Search modes - Find all my search terms | 2,831 |

# Supplement B- Study Eligibility Criteria

**Table B1:** Study eligibility criteria

|  | **Criteria** | |
| --- | --- | --- |
|  | **Inclusion** | **Exclusion** |
| Population | Adults (18 years or older), adolescents, children, and neonates requiring a PICC | Patients of any age requiring other type of catheters |
| Comparisons (intervention vs control) with specification of relevant catheter type and age group | Insertion  Sterile technique vs technique where the need for sterility is not mandated  chlorhexidine-containing antiseptic for skin preparation vs non-chlorhexidine containing antiseptics.  Inserted by clinician who has used soap and water vs inserted by clinician who has used alcohol-based hand rub for hand hygiene  Ultrasound-guided insertion vs non-ultrasound-guided guided  Silicon vs non-silicon (polyurethane)  Non-occlusive (e.g., gauze) vs occlusive dressings  Insertion team vs no specific insertion team  Inserted by individual with formal training accreditation/certification vs no specified formal training accreditation/certification  Gloves vs no gloves  Sterile gloves vs non-sterile gloves  Use of standard insertion set vs no specific requirement for a standard insertion pack/kit  UL (anywhere) vs LL (anywhere)  Lower arm (distal section of upper limb) vs upper arm (proximal section of the upper limb)  LA at insertion site versus no LA at insertion site (adolescents, children, neonates)  Scalp inserted vs anywhere other than the scalp (neonates only)  Maintenance  Formal sterile dressing vs no formal sterile dressing protocol  Continuous-infusion vs no continuous (intermittent) infusion  Systematic sterile flushing (saline or other) after product administration vs no systematic sterile flushing after product administration  Saline flushing/locking vs anticoagulant (i.e.: heparin) flushing/locking  Regular change of tubing vs no (specified) regular change of tubing  Access  Sterile/aseptic protocol vs no specified for-mal sterile/aseptic protocol  Use of closed-access device system (e.g. luer lock) vs open-access system  Removal  Scheduled removal (defined time schedule) vs clinically indicated (removed based on being clinically indicated due to a suspected or confirmed complication)  Removal within 24 hours if inserted under Emergency Conditions vs not removed within 24 hours if inserted under Emergency Conditions | Any other comparison not listed as eligible |
| Outcomes | For all comparisons:  Catheter associated or related bloodstream infections  Local infections  BSI-related mortality  All-cause mortality  Phlebitis/thrombophlebitis  Sepsis  For selected KQ:  Overall adverse events related to IVC insertion (training vs. no training, upper limb vs. lower limb, gloves .vs no gloves, insertion team vs. individual comparisons) | Studies that do not include at least one of the outcomes listed under the inclusion criteria  Unexplained catheter thrombosis  Subcutaneous extravasation |
| Publication dates | 1980 - 2023 | Older than 1980 |
| Geography | No limitations | NA |
| Settings | Any setting | NA |
| Publication language | Any languages | NA |
| Publication type | Full publications | Comments, letters to editor  Publications without an available full text |
| Study design | RCTs  Non-RCTs  Controlled observational studies  Controlled before-after studies  Interrupted time series and repeated measures studies  Before/after studies | Case series  Case reports  Systematic or nonsystematic reviews  Studies without a comparison (or incidence/prevalence studies and surveys)  Pooled data analyses |

*Abbreviations:* IVC: intravascular catheter; KQ: key question; *LA: local* NA: not applicable; PIVC: peripheral intravenous catheter; (non) RCT: randomised controlled trial; vs: versus

# Supplement C- Harmonisation of Rob Assessments

**Table C1:** Risk of bias ratings

| **Tool** | | | **Harmonised category** |
| --- | --- | --- | --- |
| **RoB 2.0** | **ROBINS-I** | **EPHPP** |  |
| High | Critical | Weak | High |
| Some concerns | Serious | Moderate | Some concerns |
|  | Moderate |  |  |
| Low | Low | Strong | Low |

Abbreviations: EPHPP=Effective Public Health Practice Project tool; RoB 2.0= Cochrane Risk of Bias tool version 2; ROBINS-I=Risk Of Bias In Non-randomized Studies of Interventions tool

# Supplement D- List of Excluded Studies and Reasons of Exclusion

## X1 Ineligible study design (n=58)

1. Corcuera Martinez MI, Aldonza Torres M, Diez Revilla AM, Maali Centeno S, Maneru Oria A, Elizari Roncal I, et al. Impact assessment following implementation of a vascular access team. J. 2022;23(1):135-44.

2. Ojo SA. Reflections on setting up a nurse-led paediatric peripherally inserted central catheter service. Br J Nurs. 2020;29(14):S16-S20.

3. Poovelikunnel TT, Duffy F, Puthussery T, Gangadharan S, McCormack F, Carpenter H, et al. Clinically indicated replacement of peripheral vascular catheters: is it safe for patients? Br J Nurs. 2020;29(8):S4-S10.

4. Barton A. Medical adhesive-related skin injuries associated with vascular access: minimising risk with Appeel Sterile. Br J Nurs. 2020;29(8):S20-S7.

5. Buetti N, Ruckly S, Lucet JC, Perozziello A, Mimoz O, Souweine B, et al. The Insertion Site Should Be Considered for the Empirical Therapy of Short-Term Central Venous and Arterial Catheter-Related Infections. Crit Care Med. 2020;48(5):739-44.

6. Sengul T, Guven B, Ocakci AF, Kaya N. Connectors as a risk factor for blood-associated infections (3-way stopcock and needleless connector): A randomized-experimental study. Am J Infect Control. 2020;48(3):275-80.

7. Cooper AS. Clinically Indicated Replacement Versus Routine Replacement of Peripheral Venous Catheters. Crit Care Nurse. 2019;39(4):67-8.

8. Oliveira LB, Fava YR, Rodrigues ARB, Franulovic AC, Ferreira NT, Puschel VAA. Management of peripherally inserted central catheter use in an intensive care unit of a teaching hospital in Brazil: a best practice implementation project. JBI Database System Rev Implement Rep. 2018;16(9):1874-86.

9. Evans NS, Ratchford EV. Catheter-related venous thrombosis. Vasc Med. 2018;23(4):411-3.

10. Stevens C, Milner KA, Trudeau J. Routine Versus Clinically Indicated Short Peripheral Catheter Replacement: An Evidence-based Practice Project. J Infus Nurs. 2018;41(3):198-204.

11. Anonymous. Effectiveness of Intracavitary Electrocardiogram Guidance in Peripherally Inserted Central Catheter Tip Placement in Neonates. J Perinat Neonatal Nurs. 2017;31(4):E2.

12. Tuffaha HW, Rickard CM, Webster J, Marsh N, Gordon L, Wallis M, et al. Cost-effectiveness analysis of clinically indicated versus routine replacement of peripheral intravenous catheters. Appl Health Econ Health Policy. 2014;12(1):51-8.

13. Walker G, Todd A. Nurse-led PICC insertion: is it cost effective? Br J Nurs. 2013;22(19):S9-15.

14. Safdar N, O'Horo JC, Maki DG. Arterial catheter-related bloodstream infection: incidence, pathogenesis, risk factors and prevention. J Hosp Infect. 2013;85(3):189-95.

15. Richardson D, Kaufman L. Reducing blood exposure risks and costs associated with SPIVC insertion. Nurs Manage. 2011;42(12):31-4.

16. Carr PJ, Glynn RW, Dineen B, Kropmans TJ. A pilot intravenous cannulation team: an Irish perspective. Br J Nurs. 2010;19(10):S19-27.

17. McMahon DD. Evaluating new technology to improve patient outcomes: a quality improvement approach. J Infus Nurs. 2002;25(4):250-5.

18. Anonymous. [Preparation, management and monitoring of an infusion]. Rev Infirm. 2002(81):28-30.

19. Funk D, Gray J, Plourde PJ. Two-year trends of peripherally inserted central catheter-line complications at a tertiary-care hospital: role of nursing expertise. Infect Control Hosp Epidemiol. 2001;22(6):377-9.

20. Jackson MM. A practical approach to designing and performing a focused study. Am J Infect Control. 1997;25(6):520-8.

21. Everitt NJ. Randomized comparison of silicone versus Teflon cannulas for peripheral intravenous nutrition. Ann R Coll Surg Engl. 1996;78(2):156.

22. van der Linden J. [Cannulas and gloves reduce the risk of blood contamination]. Lakartidningen. 1991;88(49):4237.

23. Kaieda R. Pulmonary artery catheter sleeves. Anesthesiol Rev. 1990;17(4):55-8.

24. Koller W. [Intravascular catheters and infusion therapy--microbiological and hygienic aspects]. Osterr Krankenpflegez. 1987;40:45-9.

25. Yeow KM, Wang CH, Liao TH, Chen JS, Wu JH, Yang TS, et al. The Groshong peripheral inserted central (PIC) catheter: Initial experience with ultrasound-guided insertion. Chinese Journal of Radiology. 1999;24(2):61-6.

26. Bolton D. Improving peripheral cannulation practice at an NHS Trust. Br J Nurs. 2010;19(21):1346, 8-50.

27. Bolton D. Clinically indicated replacement of peripheral cannulas. Br J Nurs. 2015;24(19):S4-12.

28. Burns DF. Developing a successful radiology nursing peripherally inserted central catheters and midline insertion program. Journal of Radiology Nursing. 2006;25(4):116-8.

29. Crawford M, Soukup SM, Woods SS, Deisch P. Peripherally inserted central catheter program. Nurs Clin North Am. 2000;35(2):349-60.

30. Silva MPC, Bragato AGdC, Ferreira DdO, Zago LB, Toffano SEM, Nicolussi AC, et al. Bundle para manuseio do cateter central de inserção periférica em neonatos. Acta Paulista de Enfermagem. 2019;32(3):261-6.

31. Davis J, Kokotis K. A new perspective for PICC line insertions: cost effectiveness and outcomes associated with an independent PICC service. Journal of the Association for Vascular Access. 2004;9(2):93-8.

32. DelPrete JS, Evans MM. Peripheral IV Site Care: What the Evidence Shows. Med-Surg Matters. 2013;22(5):4-6.

33. Driscoll M, Buckenmyer C, Spirk M, Molchany C. Advanced practice. Inserting and maintaining peripherally inserted central catheters. Medsurg Nurs. 1997;6(6):350-8.

34. East SA. Planning, implementation, and evaluation of a successful hospital-based peripherally inserted central catheter program. J Intraven Nurs. 1994;17(4):189-92.

35. Gupta P, Ruchi R, Basu S, Faridi MMA. International pediatric nursing. Life span of peripheral intravenous cannula in a neonatal intensive care unit of a developing country. J Pediatr Nurs. 2003;18(4):287-92.

36. Johann DA, Danski MTR, Pedrolo E, DeLazzari LSM, Mingorance P. Nursing care evaluation: peripherally inserted central catheter dressings in newborns. Revista Mineira de Enfermagem. 2010;14(4):515-20.

37. Jose T, Shardlow J, Kaviya A, Morley CA, Bulugahapitiya DS. 1350: Nursing intervention on a cardiology ward can reduce peripheral intravenous cannula related complications and infections. Eur J Cardiovasc Nurs. 2016;6(1_suppl):30-.

38. Da Silva MCM. AtuaÇÃo Da Enfermagem No Controle De InfecÇÃo Da Corrente SanguÍnea Relacionada Aos Cateteres Venosos PerifÉricos. Revista de Enfermagem UFPE on line. 2021;15(2):1-9.

39. Mesquita Melo E, Lima Aragão A, de Paula Pessoa CM, Teixeira Lima FE, Barbosa IV, Borges Studar RM, et al. CARE PROVIDED BY NURSING STAFF DURING THE PERIPHERAL VENIPUNCTURE PROCEDURE. Journal of Nursing UFPE / Revista de Enfermagem UFPE. 2015;9(3):1022-30.

40. Moncaio ACS, de Figueiredo RM. Knowledge and practices in the use of intermitent [sic] peripheral catheter by the nursing staff. Revista Eletronica de Enfermagem. 2009;11(3):620-7.

41. Robertson J. Liposomal lidocaine improved intravenous cannulation success rates in children. Evid Based Nurs. 2006;9(1):10.

42. Santos-Costa P, Paiva-Santos F, Sousa LB, Bernardes RA, Ventura F, Salgueiro-Oliveira A, et al. Evidence-Informed Development of a Bundle for Peripheral Intravenous Catheterization in Portugal: A Delphi Consensus Study. Nurs Rep. 2022;12(3):498-509.

43. Woody G, Davis BA. Increasing nurse competence in peripheral intravenous therapy. J Infus Nurs. 2013;36(6):413-9.

44. Millington SJ, Hendin A, Shiloh AL, Koenig S. Better With Ultrasound: Peripheral Intravenous Catheter Insertion. Chest. 2020;157(2):369-75.

45. Bergami C, Monjardim M, Macedo C. Use of peripherally inserted central catheter (PICC) in pediatric oncology. REME Rev Min Enferm. 2012;16(4):538-45.

46. Yang F, Hua R, Wu W, Bi D, Wu Y, Wang J, et al. Establishment of risk predictive nomogram model of upper extremity venous thrombosis associated with peripherally venous inserted central catheter in cancer patients. Cancer Research and Clinic. 2020;32(7):456-61.

47. Gouel-Cheron A, Swihart BJ, Warner S, Mathew L, Strich JR, Mancera A, et al. Epidemiology of ICU-Onset Bloodstream Infection: Prevalence, Pathogens, and Risk Factors Among 150,948 ICU Patients at 85 U.S. Hospitals∗. Crit Care Med. 2022;50(12):1725-36.

48. Chalela JA, Hill M, Snelgrove D, Kapoor N, Andrews C. Peripheral Insertion of Pediatric Central Venous Catheters in Adults with Difficult I.V. Access. J Emerg Med. 2023;65(1):e19-e22.

49. Ligia S, Morano SG, Kaiser F, Micozzi A, Chistolini A, Barberi W, et al. Peripherally inserted central venous catheter for pediatric acute leukemia: A retrospective 11-year single-center experience. J. 2023.

50. Liu R, Xu H, Pu L, Xie X, Chen H, Wu Z, et al. Clinical characteristics of peripherally inserted central catheter-related complications in cancer patients undergoing chemotherapy: a prospective and observational study. BMC Cancer. 2023;23(1):894.

51. Ota K, Takeda Y, Nishioka D, Oka M, Hamada E, Ota K, et al. Risk factors for contaminated blood cultures in the emergency department: A prospective cohort study. Microbial Risk Analysis. 2023;24.

52. Romitti MG, Perez CR, Pezzotti E, Motta M, Risso FM. Long peripheral catheters in neonates: filling the gap between short peripheral catheters and epicutaneous-caval catheters? J. 2023;24(5):920-5.

53. Rosenthal VD, Jin Z, Valderrama-Beltran SL, Gualtero SM, Linares CY, Aguirre-Avalos G, et al. Multinational prospective cohort study of incidence and risk factors for central line-associated bloodstream infections in ICUs of 8 Latin American countries. Am J Infect Control. 2023;51(10):1114-9.

54. Salvático E, Chávez NC, Oliva OdV, Prado S. Colocación y mantenimiento de Catéter Central de Inserción Periférica, en pacientes pediátricos del Hospital de Niños Santísima Trinidad de Córdoba, 1/1/2019 al 31/12/2020. Notas enferm (Córdoba). 2023;24(41):67-74.

55. Bolis D, D'Arrigo S, Bartesaghi A, Panzeri C, Pelegalli P, Steffanoni A, et al. Prospective clinical study on the incidence of catheter-related complications in a neurological intensive care unit: 4 years of experience. J. 2024;25(1):100-6.

56. Elli S, Cannizzo L, Giannini L, Romanato F, Trimarco C, Pessina M, et al. Femorally inserted central catheters with exit site at mid-thigh: A low risk alternative for central venous catheterization. J. 2024;25(3):808-12.

57. Quinn M, Horowitz JK, Krein SL, Gaston A, Ullman A, Chopra V. The role of hospital-based vascular access teams and implications for patient safety: A multi-methods study. J Hosp Med. 2024;19(1):13-23.

58. Takematsu Y, Shibasaki S, Tanaka T, Hiro J, Takahara T, Matsuoka H, et al. The safe implementation of peripherally inserted central catheters by nurse practitioners for patients with gastroenterological diseases in Japan: a single-center retrospective study. Surg. 2024;54(5):487-95.

## X2 Ineligible document type (n=51)

1. Rosenthal K. Do needleless connectors increase bloodstream infection risk? Nurs Manage. 2006;37(4):78-80.

2. Zeng Y, Li W, Li X, Ma L. Time to First Dressing Change after Peripherally Inserted Central Venous Catheter (PICC) Insertion in Breast Cancer Patients. Comput. 2022;2022:9380796.

3. Park JY, Jinkyu LEE, Bora H. Keyword Network Analysis of Infusion Nursing from Posts on the Q&A Board in the Intravenous Nurses Café. Healthcare Informatics Research. 2023:75-83.

4. KEEPING IVs WORKING. Qld Nurse. 2013;32(2):31-.

5. Chittick P, Sherertz RJ. Evaluation of the flush characteristics of 2 peripheral vascular catheters. Infect Control Hosp Epidemiol. 2010;31(12):1311-3.

6. Barberi-Heyob M, Merlin JL, Vigneron M, Conroy T. Addition of heparin in 5-fluorouracil solution for portal vein infusion has no influence on its stability under clinically relevant conditions. Anticancer Drugs. 1995;6(1):163-4.

7. Anonymous. To flush or not to flush with heparin. Rn. 1993;56(4):22.

8. Garate-Echenique L, Armenteros-Yeguas V, Tomás-López MA, Moraza-Dulanto I, Miranda-Serrano E, Peña-Tejera CM. Abstracts from the 3rd World Congress on Vascular Access, WoCoVA 2014, 18-20 June 2014, Berlin, Germany. J. 2014;15(3):193-239.

9. Tomford J, Hershey C, editors. THE EFFECT OF AN INTRAVENOUS THERAPY TEAM ON PERIPHERAL VENOUS CATHETER ASSOCIATED PHLEBITIS-A CONTROLLED TRIAL. Clinical Research; 1982: SLACK INC 6900 GROVE RD, THOROFARE, NJ 08086.

10. Kovacevich D, Faubion W, Braunschweig C, Smith C, Wesley J. Prevalence of catheter sepsis in parenteral nutrition patients with triple lumen vs single lumen catheters. JPEN. 1988;12:23S.

11. Wolfe J. Needle-free powder lidocaine for analgesia for venipuncture or cannulation in children. Journal of Pain & Palliative Care Pharmacotherapy. 2009;23(1):63-4.

12. Theorell C. Interventional infection control reduces the rate of catheter related bloodstream infections in neonates: University of Illinois at Chicago, Health Sciences Center; 2006.

13. Smith B, Royer TI. Doing it better: putting research into practice. New standards for improving peripheral I.V. catheter securement. Nursing. 2007;37(3):72-4.

14. Rickard C, Webster J, Wallis M, Marsh N, McGrail M, French V, et al. Peripheral intravenous catheters can be left in situ until clinically indicated for removal: Randomised controlled trial. Aust Crit Care. 2012;25(2):125-.

15. O'Leary C. Improving peripheral intravenous cannulation in nurses: computer based education versus intensive practice. Oncol Nurs Forum. 2008;35(3):500-.

16. O'Brien EE, Rosenberg S, Bollinger E, Lenhart L, Sramek S, Mikolajczak A, et al. Implementation of a Comprehensive, Unit-Based Protocol for Prevention of Neonatal Catheter Associated Blood Stream Infections. Journal of Obstetric, Gynecologic & Neonatal Nursing. 2014;43(Supp 1):S63-S4.

17. Legriel S, Mongardon N, Troche G, Bruneel F, Bedos JP. Catheter-related colonization or infection in critically ill patients: Is the number of simultaneous catheters a risk factor? Am J Infect Control. 2011;39(1):83-5.

18. Krau SD. Intravascular catheter dressings with chlorhexidine-impregnated sponges reduced infections in the ICU. Evid Based Nurs. 2009;12(4):115.

19. Huang DT, Angus DC. Therapy guided by pulmonary artery catheter for high-risk surgical patients was not better than standard care. ACP J Club. 2003;139(3):66-.

20. DeGennaro D. Dedicated Team for Central Line Placement: Walden University; 2014.

21. Corley A, Ullman A, Marsh N, Genzel J, Larsen E, Monteagle E, et al. Securement bundles to prevent peripheral intravenous catheter failure – the SECURE-PIVC trial: A pilot randomised controlled trial. Infect Dis Health. 2021;26:S8-S9.

22. Cobett S, LeBlanc A. IV site infection: a prospective, randomized clinical trial comparing the efficacy of three methods of skin antisepsis: CINA conference '99. CINA: Official Journal of the Canadian Intravenous Nurses Association. 1999;15:48-9.

23. Chopra V, Saint S. Vascular catheter infections: time to get technical. Lancet. 2015;386(10008):2034-6.

24. Brimage-Williams TA. Does a Practice Change to Chlorhexidine Gluconate Compared to Betadine for Skin Prep in PICC Insertion Decrease Central Line Infections? Does A Practice Change To Chlorhexidine Gluconate Compared To Betadine For Skin Prep In PICC Insertion Decrease Central Line Infections? 2018:1-.

25. Benbow M. Clinically indicated and routine replacement of peripheral intravenous catheters did not differ for catheter failure. Evid Based Nurs. 2009;12(1):19.

26. Auron M. PICC vs. midline catheter use for short-term indications was linked to major complications at </=30 d. Ann Intern Med. 2022;175(4):JC47.

27. Ahc M. Infectious Disease Alert Updates. Infectious Disease Alert. 2019;39(2):N.PAG-N.PAG.

28. Payne-James JJ, Rogers J, Bray MJ, Rana SK, McSwiggan D, Silk DB. Comparison of polyurethane and polytetrafluoroethylene peripheral venous cannulas in the development of thrombophlebitis: a randomised double-blind study. Clinical nutrition (Edinburgh, Scotland). 1990;9(Spec Suppl):65.

29. Martinsen C, Findlay CP, Smithies MN. A randomised study to assess dressing performance of two peripheral intravenous cannula dressings. European society of intensive care medicine; 1998, 6-9 september; stockholm, sweden. 1998.

30. Pappas N. Ultrasound guidance as a rescue technique for peripheral intravenous cannulation. Aana J. 2006;74:464.

31. Boyd AT, McMahon MJ. A controlled trial of silicone elastomer versus polyurethane catheters for intravenous feeding. Clin-nutr. 1988;7 Spec Suppl:99.

32. Palmer D. Fewer patients dislodged peripheral intravenous catheters with transparent dressings than with gauze dressings. Evidence based nursing. 1998;1(3):81‐.

33. Galang H. Pilot of a Randomized Trial Comparing Outcomes of Three Types of Peripheral Intravenous Catheters (PIVC): utilizing the Plan, Do, Study, Act Cycle. Dissertation/ thesis. 2017:1‐.

34. Ballantyne M, McNair C, Stevens B, Gibbins S, Newman C, Ung E. Evaluation of the safety and efficacy of amethocaine gel for reducing pain from peripherally inserted central catheter insertion. Pediatr Res. 2002;51(4):363A.

35. Danek GD. Effect of local anesthetic on stress response of infants undergoing peripherally inserted central catheter placement. Dissertation/ thesis. 1998:101 p.

36. Avelar AFM, Peterlini MAS, Pedreira MLG. Ultrassonografia Vascular na Utilização de CateteresIntravenosos Periféricos em Crianças: estudo clinico, randômico e controlado. Rev paul enferm. 2009;28(4):[55]-[].

37. Oliveira GLRd. Avaliação de tecnologia em saúde: cateter de segurança completo. 2015. p. 96-.

38. Maki DG. ACP Journal Club. Clinically indicated and routine replacement of peripheral IV catheters did not differ for phlebitis. Ann Intern Med. 2013;158(2):JC8.

39. Jane Shaw S. Use of closed cannulae in peripheral intravenous cannulation. Nurs Stand. 2017;31(36):54-63.

40. Armstrong P, Young C, McKeown D. Ethyl chloride and venepuncture pain: a comparison with intradermal lidocaine. Can J Anaesth. 1990;37(6):656-8.

41. Ring WH. Recent comparison of catheters makes unsubstantiated conclusions. J Intraven Nurs. 1991;14(5):288.

42. Hadaway LC. I.v. risk reducers. Nurs Manage. 2001;32(12):57-8.

43. Rosenthal K. When your patient develops phlebitis. Nursing. 2006;36(2):14.

44. Marcatto Jde O, Vasconcelos PC, Araujo CM, Tavares EC, Pereira e Silva Y. EMLA versus glucose for PICC insertion: a randomised triple-masked controlled study. Arch Dis Child Fetal Neonatal Ed. 2011;96(6):F467-8.

45. Goudet V, Timsit JF, Lucet JC, Lepape A, Balayn D, Seguin S, et al. Comparison of four skin preparation strategies to prevent catheter-related infection in intensive care unit (CLEAN trial): a study protocol for a randomized controlled trial. Trials. 2013;14:114.

46. Ullman A, Keogh S, Marsh N, Rickard C. Routine versus clinically indicated replacement of peripheral catheters. Br J Nurs. 2015;24(2):S14.

47. Rangarajan S, Morgenstern J, Milne WK, Heitz C. Hot Off the Press: Peripheral Intravenous Cannula Insertion and Use in the Emergency Department. Acad Emerg Med. 2018;25(6):668-71.

48. Ray-Barruel G, Cooke M, Mitchell M, Chopra V, Rickard CM. Implementing the I-DECIDED clinical decision-making tool for peripheral intravenous catheter assessment and safe removal: protocol for an interrupted time-series study. BMJ Open. 2018;8(6):e021290.

49. Blanco-Mavillard I, Bennasar-Veny M, De Pedro-Gomez JE, Moya-Suarez AB, Parra-Garcia G, Rodriguez-Calero MA, et al. Implementation of a knowledge mobilization model to prevent peripheral venous catheter-related adverse events: PREBACP study-a multicenter cluster-randomized trial protocol. Implement Sci. 2018;13(1):100.

50. Guenezan J, Drugeon B, O'Neill R, Caillaud D, Senamaud C, Pouzet C, et al. Skin antisepsis with chlorhexidine-alcohol versus povidone iodine-alcohol, combined or not with use of a bundle of new devices, for prevention of short-term peripheral venous catheter-related infectious complications and catheter failure: an open-label, single-centre, randomised, four-parallel group, two-by-two factorial trial: CLEAN 3 protocol study. BMJ Open. 2019;9(4):e028549.

51. Scholten HJ, Hoever Y, Kanters E, Hoveling T, de Wild M, Korsten EHM, et al. Ultrasound transducer with dynamic visual aid improves out-of-plane vascular access: a feasibility study. Br J Anaesth. 2022;129(3):e69-e71.

## X3_Ineligible study population (n=97)

1. Allen GB, Miller V, Nicholas C, Hess S, Cordes MK, Fortune JB, et al. A multitiered strategy of simulation training, kit consolidation, and electronic documentation is associated with a reduction in central line-associated bloodstream infections. Am J Infect Control. 2014;42(6):643-8.

2. Aly R, Bayles C, Maibach H. Restriction of bacterial growth under commercial catheter dressings. Am J Infect Control. 1988;16(3):95-100.

3. Anderson S, Cockrell J, Beller P, Murphy E, Nelson P, Hawkins M, et al. Administration of local anesthetic agents to decrease pain associated with peripheral vascular access. J Infus Nurs. 2010;33(6):353-61.

4. Aygun H, Armagan E, Ozdemir F, Kose A, Selimoglu K, Koksal O, et al. Comparison of Ice and Lidocaine- Prilocaine Cream Mixture in the Reduction of Pain During Peripheral Intravenous Cannulation in Emergency Department Patients. Journal of Academic Emergency Medicine. 2013;12(1):27-9.

5. Bahl A, Karabon P, Chu D. Comparison of Venous Thrombosis Complications in Midlines Versus Peripherally Inserted Central Catheters: Are Midlines the Safer Option? Clin Appl Thromb Hemost. 2019;25:1076029619839150.

6. Bahl A, Mielke N, Gibson SM, George J. The use of procedural kits may reduce unscheduled central line dressing changes: A matched pre-post intervention study. J. 2024;25(3):73-81.

7. Balanyuk I, Ledonne G, Provenzano M, Bianco R, Meroni C, Ferri P, et al. Distraction Technique for pain reduction in Peripheral Venous Catheterization: randomized, controlled trial. Acta Biomed. 2018;89(4-S):55-63.

8. Barria RM, Lorca P, Munoz S. Randomized controlled trial of vascular access in newborns in the neonatal intensive care unit. J Obstet Gynecol Neonatal Nurs. 2007;36(5):450-6.

9. Bjornestam B, Hedborg K, Ransjo U, Finkel Y. The effect of a 1-hour training program on the incidence of bacteremia in pediatric patients receiving parenteral nutrition. J Intraven Nurs. 2000;23(3):154-7.

10. Bomberg H, Kubulus C, List F, Albert N, Schmitt K, Graber S, et al. Diabetes: a risk factor for catheter-associated infections. Reg Anesth Pain Med. 2015;40(1):16-21.

11. Bouza E, Munoz P, Lopez-Rodriguez J, Jesus Perez M, Rincon C, Martin Rabadan P, et al. A needleless closed system device (CLAVE) protects from intravascular catheter tip and hub colonization: a prospective randomized study. J Hosp Infect. 2003;54(4):279-87.

12. Brors G, Gjeilo KH, Lund T, Skevik K, Aa E, Hovik LH, et al. Amiodarone-induced phlebitis: incidence and adherence to a clinical practice guideline. Eur J Cardiovasc Nurs. 2023;22(8):824-31.

13. Brown J, Larson M. Pain during insertion of peripheral intravenous catheters with and without intradermal lidocaine. Clin Nurse Spec. 1999;13(6):283-5; quiz 6-8.

14. Cates M. Evaluation of Peripheral Intravenous Practices. Vascular Access. 2016;10(1):8-13.

15. Chen W, Deng H, Shen L, Qin M, He L. A comprehensive intervention program on the long-term placement of peripherally inserted central venous catheters. J Cancer Res Ther. 2014;10(2):359-62.

16. Clifton GD, Branson P, Kelly HJ, Dotson LR, Record KE, Phillips BA, et al. Comparison of normal saline and heparin solutions for maintenance of arterial catheter patency. Heart Lung. 1991;20(2):115-8.

17. Connolly S, Korzemba H, Harb G, Lebel F, Syltevik C. Techniques for hyaluronidase-facilitated subcutaneous fluid administration with recombinant human hyaluronidase: the increased flow utilizing subcutaneously enabled administration technique (INFUSE AT) study. J Infus Nurs. 2011;34(5):300-7.

18. Corley A, Cantara M, Gardner J, Trexler P, Rock C, Maragakis LL. Central line-associated bloodstream infection rate elevation: Attributable to National Healthcare Safety Network surveillance definition changes, ongoing opportunities for infection prevention, or both? Am J Infect Control. 2017;45(9):1030-2.

19. Crecelius C, Rouhfar L, Beirne OR. Venous cannulation and topical ethyl chloride in patients receiving nitrous oxide. Anesth Prog. 1999;46(3):100-3.

20. de Neef M, Heijboer H, van Woensel JB, de Haan RJ. The efficacy of heparinization in prolonging patency of arterial and central venous catheters in children: a randomized double-blind trial. Pediatr Hematol Oncol. 2002;19(8):553-60.

21. Deguzman ZC, O'Mara SK, Sulo S, Haines T, Blackburn L, Corazza J. Bacteriostatic normal saline compared with buffered 1% lidocaine when injected intradermally as a local anesthetic to reduce pain during intravenous catheter insertion. J Perianesth Nurs. 2012;27(6):399-407.

22. Del Cotillo M, Grane N, Llavore M, Quintana S. Heparinized solution vs. saline solution in the maintenance of arterial catheters: a double blind randomized clinical trial. Intensive Care Med. 2008;34(2):339-43.

23. Deshpande P, Jain A, Shah PS. Outcomes associated with early removal versus retention of peripherally inserted central catheters after diagnosis of catheter-associated infections in neonates. J Matern Fetal Neonatal Med. 2016;29(24):4082-7.

24. Dickey J. Effectiveness of intradermally injected lidocaine hydrochloride as a local anesthetic for intravenous catheter insertion. J Emerg Nurs. 1988;14(3):160-3.

25. Ecevit A, Ince DA, Hanta D, Kurt A, Harman A, Özkiraz S, et al. Comparing the complications of ultrasoundguided versus percutaneously inserted central venous catheters in newborn infants in the neonatal intensive care unit. Cocuk Sagligi ve Hastaliklari Dergisi. 2013;56(1):12-9.

26. Eggimann P, Pagani JL, Dupuis-Lozeron E, Ms BE, Thevenin MJ, Joseph C, et al. Sustained reduction of catheter-associated bloodstream infections with enhancement of catheter bundle by chlorhexidine dressings over 11 years. Intensive Care Med. 2019;45(6):823-33.

27. Esteve F, Pujol M, Ariza J, Gudiol F, Verdaguer R, Cisnal M, et al. [Impact of a prevention program for catheter-related bloodstream infection in the intensive care unit of a tertiary hospital]. Enferm Infecc Microbiol Clin. 2009;27(10):561-5.

28. Fein JA, Callahan JM, Boardman CR. Intravenous catheterization in the ED: is there a role for topical anesthesia? Am J Emerg Med. 1999;17(6):624-5.

29. Forestier F, Rossi H, Calderon J, Soubiron L, Bourdarias B, Janvier G. [Training for adult subclavian venous catheterization: use of real-time echography]. Ann Fr Anesth Reanim. 2002;21(9):698-702.

30. Fry C, Aholt D. Local anesthesia prior to the insertion of peripherally inserted central catheters. J Infus Nurs. 2001;24(6):404-8.

31. Ganter-Ritz V, Speroni KG, Atherton M. A randomized double-blind study comparing intradermal anesthetic tolerability, efficacy, and cost-effectiveness of lidocaine, buffered lidocaine, and bacteriostatic normal saline for peripheral intravenous insertion. J Infus Nurs. 2012;35(2):93-9.

32. Garcia-Teresa MA, Casado-Flores J, Delgado Dominguez MA, Roqueta-Mas J, Cambra-Lasaosa F, Concha-Torre A, et al. Infectious complications of percutaneous central venous catheterization in pediatric patients: a Spanish multicenter study. Intensive Care Med. 2007;33(3):466-76.

33. Griffin MP, Siadaty MS. Papaverine prolongs patency of peripheral arterial catheters in neonates. J Pediatr. 2005;146(1):62-5.

34. Gross I, Block C, Benenson S, Cohen MJ, Brezis M. [The effect of an intervention on rates of central vascular catheter-related bloodstream infection in intensive care units at the Hadassah Medical Center]. Harefuah. 2013;152(1):16-20, 60.

35. Hendry F, Checketts MR, McLeod GA. Effect of intradermal anaesthesia on success rate and pain of intravenous cannulation: a randomized non-blind crossover study. Scott Med J. 2011;56(4):210-3.

36. Holdgate A, Wong G. Does local anaesthetic affect the success rate of intravenous cannulation? Anaesth Intensive Care. 1999;27(3):257-9.

37. Hosseini MB, Jodeiri B, Mahallei M, Abdoli-Oskooi S, Safari A, Salimi Z. Early outcome of peripherally inserted central catheter versus peripheral IV line in very low birth weight neonates. Feyz Journal of Kashan University of Medical Sciences. 2014;17(6):561-7.

38. Hudson DA, Engelbrecht G, Duminy FJ. Another method to prevent venous thrombosis in microsurgery: an in situ venous catheter. Plast Reconstr Surg. 2000;105(3):999-1003.

39. Jeon Y, Choi S, Kim H. Evaluation of a simplified augmented reality device for ultrasound-guided vascular access in a vascular phantom. J Clin Anesth. 2014;26(6):485-9.

40. Johnson BH, Rypins EB. Single-lumen vs double-lumen catheters for total parenteral nutrition. A randomized, prospective trial. Arch Surg. 1990;125(8):990-2.

41. Koeppen M, Weinert F, Oehlschlaeger S, Koerner A, Rosenberger P, Haeberle HA. Needle-free connectors catheter-related bloodstream infections: a prospective randomized controlled trial. Intensive Care Med Exp. 2019;7(1):63.

42. Kondo M, Kim SJ, Fujiwara Y, Iinuma A, Koji K, Irie Y, et al. [Evaluation of ten intravenous catheters for operability and safety in the infusion room]. Gan To Kagaku Ryoho. 2004;31(12):2005-8.

43. Koren O, Abu Rajab Saaida R, Rozner E, Turgeman Y. Outcomes and safety of concurrent coronary and peripheral catheterization (REVascularization in concomitant PERIpheral artery disease and coronary artery disease REV-PERICAD Study). Catheter Cardiovasc Interv. 2020;96(3):E317-E23.

44. Ks D, Balachander B, Rao Pn S. Feeding Practices, Lines, and Hospital-Acquired Infection during the Sustenance Phase of Infection Control Quality Improvement. Journal of Pediatric Infectious Diseases. 2020;16(01):026-30.

45. Kulkarni M, Elsner C, Ouellet D, Zeldin R. Heparinized saline versus normal saline in maintaining patency of the radial artery catheter. Can J Surg. 1994;37(1):37-42.

46. Larsen EN, Corley A, Mitchell M, Lye I, Powell M, Tom S, et al. A pilot randomised controlled trial of dressing and securement methods to prevent arterial catheter failure in intensive care. Aust Crit Care. 2021;34(1):38-46.

47. Larson J, Hanson J. In profile. Developing a peripherally inserted central catheter service with registered nurses. Can Oncol Nurs J. 1999;9(3):145-6.

48. Levy I, Katz J, Solter E, Samra Z, Vidne B, Birk E, et al. Chlorhexidine-impregnated dressing for prevention of colonization of central venous catheters in infants and children: a randomized controlled study. Pediatr Infect Dis J. 2005;24(8):676-9.

49. Looi JL, Cave A, El-Jack S. Learning curve in transradial coronary angiography. Am J Cardiol. 2011;108(8):1092-5.

50. Lysakowski C, Dumont L, Tramer MR, Tassonyi E. A needle-free jet-injection system with lidocaine for peripheral intravenous cannula insertion: a randomized controlled trial with cost-effectiveness analysis. Anesth Analg. 2003;96(1):215-9, table of contents.

51. Maki, Dg, Ringer, M, Alvarado, Cj. Prospective randomized trial of povidone-iodine, alcohol, and chlorhexidine for prevention of infection associated with central venous and arterial catheters. CINA: official journal of the canadian intravenous nurses association. 1993;9(1):10‐5.

52. Maki DG, Ringer M, Alvarado CJ. Prospective randomised trial of povidone-iodine, alcohol, and chlorhexidine for prevention of infection associated with central venous and arterial catheters. Lancet. 1991;338(8763):339-43.

53. Masaki H, Watanabe H, Degawa S, Yoshimine H, Asoh N, Rikitomi N, et al. Significant reduction of methicillin-resistant Staphylococcus aureus bacteremia in geriatric wards after introduction of infection control measures against nosocomial infections. Intern Med. 2001;40(3):214-20.

54. McDonagh DL, Macy GE. Skin antisepsis with chlorhexidine- alcohol versus povidoneiodine alcohol, with and without skin scrubbing, for prevention of intravascular catheter-related infection (CLEAN): an open-label, multicentre, randomised, controlled, two-by-two factorial trial. J Neurosurg Anesthesiol. 2016;28(2):165-6.

55. Mimoz O, Lucet JC, Kerforne T, Pascal J, Souweine B, Goudet V, et al. Skin antisepsis with chlorhexidine-alcohol versus povidone iodine-alcohol, with and without skin scrubbing, for prevention of intravascular-catheter-related infection (CLEAN): an open-label, multicentre, randomised, controlled, two-by-two factorial trial. Lancet. 2015;386(10008):2069-77.

56. Muller WD, Trittenwein G, Stein J, Schober P. [Percutaneous-peripheral vena cava catheterization in intensive care of premature and newborn infants. Comparison of Shaw's silastic catheter with the customary polyvinyl catheter]. Anaesthesist. 1983;32(11):545-7.

57. Nasr B, Carret M, Pluchon K, Yven C, Bezon E, Goueffic Y. Perioperative adverse events in percutaneous versus open brachial access. J Vasc Surg. 2023;77(3):864-9.

58. Olson C, Heilman JM. Clinical Performance of a New Transparent Chlorhexidine Gluconate Central Venous Catheter Dressing. Journal of the Association for Vascular Access. 2008;13(1):13-9.

59. Ozyazicioglu N, Arikan D. The effect of nurse training on the improvement of intravenous applications. Nurse Educ Today. 2008;28(2):179-85.

60. Palomar M, Alvarez-Lerma F, Riera A, Diaz MT, Torres F, Agra Y, et al. Impact of a national multimodal intervention to prevent catheter-related bloodstream infection in the ICU: the Spanish experience. Crit Care Med. 2013;41(10):2364-72.

61. Patterson P, Hussa AA, Fedele KA, Vegh GL, Hackman CM. Comparison of 4 analgesic agents for venipuncture. Aana J. 2000;68(1):43-51.

62. Phumyeesoon S, Thanomsingh P. Early Elective Replacement of Umbilical Venous Catheter with Peripherally Inserted Central Catheter to Reduce Central Line-Associated Blood Stream Infections in Premature Infants: A Randomized Trial. J Med Assoc Thai. 2023;106(1):63-9.

63. Picardi M, Giordano C, Della Pepa R, Pugliese N, Esposito M, Abagnale DP, et al. Intravascular Complications of Central Venous Catheterization by Insertion Site in Acute Leukemia during Remission Induction Chemotherapy Phase: Lower Risk with Peripherally Inserted Catheters in a Single-Center Retrospective Study. Cancers. 2023;15(7):04.

64. Pitman JS, Buscemi M, Funk EM, Weaver S, Thompson JA, Falyar C. Incorporating Evidence-Based Ultrasound-Guided Vascular Access (USGVA) Standards Into the Nurse Anesthetist Armamentarium: A Quality Improvement Project. J Perianesth Nurs. 2023;38(4):564-71.

65. Ribada Fraga D, Estévez Hermida FM, Ferreiro Martínez MdM. Aplicación de Cloruro de Etilo en la canalización de vías venosas periféricas a pacientes oncológicos. Enfermeria Oncologica. 2022;24(1):39-49.

66. Robinson JL, Casey LM, Huynh HQ, Spady DW. Prospective cohort study of the outcome of and risk factors for intravascular catheter-related bloodstream infections in children with intestinal failure. JPEN Journal of Parenteral & Enteral Nutrition. 2014;38(5):625-30.

67. Rohm KD, Schollhorn TA, Gwosdek MJ, Piper SN, Maleck WH, Boldt J. Do we necessarily need local anaesthetics for venous cannulation? A comparison of different cannula sizes. Eur J Anaesthesiol. 2004;21(3):214-6.

68. Rubio DRM, Soto AG, Oliveira F, Lopez F, Sola A. EMLA cream for percutaneous venous central line placement in preterm infants: a randomized, controlled trial. Pediatr Res. 2000;47(4):415A.

69. Rusch D, Koch T, Spies M, Hj Eberhart L. Pain During Venous Cannulation. Dtsch Arztebl Int. 2017;114(37):605-11.

70. Rzhevskiy A, Popov A, Pavlov C, Anissimov Y, Zvyagin A, Levin Y, et al. Intradermal injection of lidocaine with a microneedle device to provide rapid local anaesthesia for peripheral intravenous cannulation: A randomised open-label placebo-controlled clinical trial. PLoS ONE. 2022;17(1):e0261641.

71. Sabak M, Al-Hadidi A, Demashkieh L, Zengin S, Hakmeh W. Homemade phantoms improve ultrasound-guided vein cannulation confidence and procedural performance on patients. Ulus Travma Acil Cerrahi Derg. 2022;28(9):1312-6.

72. Saliba P, Hornero A, Cuervo G, Grau I, Jimenez E, Garcia D, et al. Mortality risk factors among non-ICU patients with nosocomial vascular catheter-related bloodstream infections: a prospective cohort study. J Hosp Infect. 2018;99(1):48-54.

73. Selby IR, Bowles BJ. Analgesia for venous cannulation: a comparison of EMLA (5 minutes application), lignocaine, ethyl chloride, and nothing. J R Soc Med. 1995;88(5):264-7.

74. Shepherd EG, Kelly TJ, Vinsel JA, Cunningham DJ, Keels E, Beauseau W, et al. Significant reduction of central-line associated bloodstream infections in a network of diverse neonatal nurseries. J Pediatr. 2015;167(1):41-6.e3.

75. Silveira E, Slaviero JV, Azmus A, Moraes CVd, Teixeira JV, Leite RS, et al. Experiência inicial com a extensão de cateter-guia ExpressmanTM em um centro terciário de cardiologia de alto volume. J Transcatheter Interv. 2023;31:eA20230009.-eA.

76. Smirk C, Soosay Raj T, Smith AL, Morris S. Neonatal percutaneous central venous lines: fit to burst. Arch Dis Child Fetal Neonatal Ed. 2009;94(4):F298-300.

77. Stolker JM, Hadid M, Hussain ZM, Rough SJ, Ibrahim M, Kennedy KF, et al. Training the next generation of invasive cardiologists: Feasibility of implementing a trans-radial access program at an academic hospital. Cardiovasc Revasc Med. 2016;17(7):431-7.

78. Stotter AT, Ward H, Waterfield AH, Hilton J, Sim AJ. Junctional care: the key to prevention of catheter sepsis in intravenous feeding. JPEN J Parenter Enteral Nutr. 1987;11(2):159-62.

79. Tamura T, Kobayashi E, Kawaguchi M, Matsuoka Y, Fujii A, Ando M, et al. Comparison between the effects of normal saline with and without heparin for the prevention and management of arterial catheter occlusion: a triple-blinded randomized trial. J Anesth. 2021;35(4):536-42.

80. Thind D, Roberts SJ, van der Griend BF. Coolsense(R) versus EMLA(R) for peripheral venous cannulation in adult volunteers: A randomised crossover trial. Anaesth Intensive Care. 2021;49(6):468-76.

81. Timsit JF, Schwebel C, Bouadma L, Geffroy A, Garrouste-Orgeas M, Pease S, et al. Chlorhexidine-impregnated sponges and less frequent dressing changes for prevention of catheter-related infections in critically ill adults: a randomized controlled trial. Jama. 2009;301(12):1231-41.

82. Trautmann M, Zauser B, Wiedeck H, Buttenschon K, Marre R. Bacterial colonization and endotoxin contamination of intravenous infusion fluids. J Hosp Infect. 1997;37(3):225-36.

83. Tu Z, Tan Y, Liu L, Xie J, Xu Y, Liu W. Ultrasound-Guided Cannulation of the Great Saphenous Vein in Neonates: A Randomized Study. Am J Perinatol. 2023;40(11):1217-22.

84. Tully RP, McGrath BA, Moore JA, Rigg J, Alexander P. Observational Study of the Effect of Heparin-Containing Flush Solutions on the Incidence of Arterial Catheter Occlusion. Journal of the Intensive Care Society. 2014;15(3):213-5.

85. Tuncali BE, Kuvaki B, Tuncali B, Capar E. A comparison of the efficacy of heparinized and nonheparinized solutions for maintenance of perioperative radial arterial catheter patency and subsequent occlusion. Anesth Analg. 2005;100(4):1117-11121.

86. Ullman AJ, Cooke M, Kleidon T, Rickard CM. Road map for improvement: Point prevalence audit and survey of central venous access devices in paediatric acute care. J Paediatr Child Health. 2017;53(2):123-30.

87. Valles J, Fernandez I, Alcaraz D, Chacon E, Cazorla A, Canals M, et al. Prospective randomized trial of 3 antiseptic solutions for prevention of catheter colonization in an intensive care unit for adult patients. Infect Control Hosp Epidemiol. 2008;29(9):847-53.

88. Vitto MJ, Myers M, Vitto CM, Evans DP. Perceived Difficulty and Success Rate of Standard Versus Ultrasound-Guided Peripheral Intravenous Cannulation in a Novice Study Group: A Randomized Crossover Trial. J Ultrasound Med. 2016;35(5):895-8.

89. Volkow P, Vazquez C, Tellez O, Aguilar C, Barrera L, Rodrgiuez E, et al. Polyurethane II catheter as long-indwelling intravenous catheter in patients with cancer. Am J Infect Control. 2003;31(7):392-6.

90. Walz JM, Ellison RT, 3rd, Mack DA, Flaherty HM, McIlwaine JK, Whyte KG, et al. The bundle "plus": the effect of a multidisciplinary team approach to eradicate central line-associated bloodstream infections. Anesth Analg. 2015;120(4):868-76.

91. Wang F, Wang Y, Liu J. Risk factors for peripherally inserted central venous catheter-related complications in children: A retrospective cohort study. Medicine (United States). 2023;102(39):E34924.

92. Wellard S, Palaster L. An evaluation of two methods of pre-cannulation skin disinfection. Aust J Adv Nurs. 1996;14(1):3-7.

93. Whitta RK, Hall KF, Bennetts TM, Welman L, Rawlins P. Comparison of normal or heparinised saline flushing on function of arterial lines. Crit Care Resusc. 2006;8(3):205-8.

94. Xiong J, Pan T, Jin H, Xie X, Wang Y, Wang D. A comparison of heparinised and non-heparinised normal saline solutions for maintaining the patency of arterial pressure measurement cannulae after heart surgery. J Cardiothorac Surg. 2019;14(1):39.

95. Yasuda H, Sanui M, Abe T, Shime N, Komuro T, Hatakeyama J, et al. Comparison of the efficacy of three topical antiseptic solutions for the prevention of catheter colonization: a multicenter randomized controlled study. Crit Care. 2017;21(1):320.

96. Yıldız Y, Ulukan MÖ, Cantürk E, Gül YG, Erkanli K, Demiraran Y, et al. The comparison of anatomical description and ultrasound-guided percutaneous central

venous catheter insertion methods single center experience. Journal of Cardio-Vascular-Thoracic Anaesthesia and Intensive Care Society. 2019;25(3):160-6.

97. Zevola DR, Dioso J, Moggio R. Comparison of heparinized and nonheparinized solutions for maintaining patency of arterial and pulmonary artery catheters. Am J Crit Care. 1997;6(1):52-5.

## X4_Ineligible intervention (n=157)

1. Aguda C, Sloan C, Klimpel K, Lewis C, Holt DM. The Effects of Focused Nursing Education on 3F Groshong™ PICC Occlusion Rates: The Experience of One Tertiary Pediatric Care Facility. Journal of the Association for Vascular Access. 2011;15(4):213-21.

2. Akers AS, Chelluri L. Peripherally inserted central catheter use in the hospitalized patient: is there a role for the hospitalist? J Hosp Med. 2009;4(6):E1-4.

3. Alaiev D, Krouss M, Israilov S, Musser L, Talledo J, Mestari N, et al. Nudging to select single-lumen over multiple-lumen peripherally inserted central catheters (PICCs) in a large safety net system. Infection Control and Hospital Epidemiology. 2023;24.

4. Aly H, Herson V, Duncan A, Herr J, Bender J, Patel K, et al. Is bloodstream infection preventable among premature infants? A tale of two cities. Pediatrics. 2005;115(6):1513-8.

5. Anantasit N, Cheeptinnakorntaworn P, Khositseth A, Lertbunrian R, Chantra M. Ultrasound Versus Traditional Palpation to Guide Radial Artery Cannulation in Critically Ill Children: A Randomized Trial. J Ultrasound Med. 2017;36(12):2495-501.

6. Anderson D. Parent application of eutectic mixture of local anaesthetics seemed to be as effective as clinician application for reducing children's pain and distress during intravenous insertion. Evidence based nursing. 2000:11‐.

7. Anonymous. Efficacy of Vein Visualization Devices for Peripheral Intravenous Catheter Placement in Preterm Infants: A Randomized Clinical Trial. J Perinat Neonatal Nurs. 2019;33(1):E2.

8. Arjun R, Niyas VKM, Sasidharan A, Jomes J, Yadav MK, Kesavan S. Peripherally Inserted Central Catheters-associated blood stream infections-occurrence, risk factors, and pathogens, a single center study. J. 2023;24(4):187-92.

9. Arnts IJ, Schrijvers NM, van der Flier M, Groenewoud JM, Antonius T, Liem KD. Central line bloodstream infections can be reduced in newborn infants using the modified Seldinger technique and care bundles of preventative measures. Acta Paediatr. 2015;104(4):e152-7.

10. Avsar G, Ciftci B, Ozlu ZK, Uslu H, Coskun MV, Kasikci M, et al. Effect of Peripheral Venous Catheter Care on Microbiological Colonization: a Randomized Controlled Trial. International journal of caring sciences. 2021;14(2):937‐45.

11. Baxi SM, Shuman EK, Scipione CA, Chen B, Sharma A, Rasanathan JJ, et al. Impact of postplacement adjustment of peripherally inserted central catheters on the risk of bloodstream infection and venous thrombus formation. Infect Control Hosp Epidemiol. 2013;34(8):785-92.

12. Beards SC, Doedens L, Jackson A, Lipman J. A comparison of arterial lines and insertion techniques in critically ill patients. Anaesthesia. 1994;49(11):968-73.

13. Bell DN, O'Connor A, Leslie K. The influence of flushing epidural catheters before use on detection of intravenous placement: an in vitro and in vivo study. Anaesth Intensive Care. 2007;35(6):932-8.

14. Bilagi AP, Sharma J, Rorke J, Keszler M. Trimming of percutaneous central venous catheters prior to insertion and risk of catheter related sepsis in the NICU. Pediatr Res. 2004;55:16.

15. Bjorkman L, Ohlin A. Scrubbing the hub of intravenous catheters with an alcohol wipe for 15 sec reduced neonatal sepsis. Acta Paediatr. 2015;104(3):232-6.

16. Burek AG, Davis MB, Pechous B, Shaughnessy EE, Meier KA, Mooney S, et al. Inappropriate Use of Peripherally Inserted Central Catheters in Pediatrics: A Multisite Study. Hosp. 2024;14(3):180-8.

17. Buyukyilmaz F, Sahiner NC, Caglar S, Eren H. Effectiveness of an Intravenous Protection Device in Pediatric Patients on Catheter Dwell Time and Phlebitis Score. Asian Nurs Res (Korean Soc Nurs Sci). 2019;13(4):236-41.

18. Campbell SG, Trojanowski J, Ackroyd-Stolarz SA. How often should peripheral intravenous catheters in ambulatory patients be flushed? J Infus Nurs. 2005;28(6):399-404.

19. Catho G, Rosa Mangeret F, Sauvan V, Chraïti MN, Pfister R, Baud O, et al. Risk of catheter-associated bloodstream infection by catheter type in a neonatal intensive care unit: a large cohort study of more than 1100 intravascular catheters. J Hosp Infect. 2023;139:6-10.

20. Chabni N, Regagba D, Meguenni K, Ghomari SM, Smahi MC. Risk factors for hospital-acquired infections in the neonatal polyvalent unit of specialized mother-child hospital Tlemcen in western Algeria, "case-control study". Journal de Pediatrie et de Puericulture. 2015;28(2):71-9.

21. Chasseigne V, Buisson M, Serrand C, Leguelinel-Blache G, Kinowski JM, Goupil J, et al. Pharmaceutical Analysis of Peripherally Inserted Central Catheter Requests Increases the Use of Single-Lumen Catheters: A Prospective Pilot Study. J Patient Saf. 2022;18(8):e1238-e42.

22. Chathas MK, Paton JB, Fisher DE. Percutaneous central venous catheterization. Three years' experience in a neonatal intensive care unit. Am J Dis Child. 1990;144(11):1246-50.

23. Cho CH, Schlattmann P, Nagel S, Schmittbuttner N, Hartung F, Teichgraber UK. Cephalad dislocation of PICCs under different upper limb positions: influence of age, gender, BMI, number of lumens. J. 2018;19(2):141-5.

24. Chopra V, Kaatz S, Grant P, Swaminathan L, Boldenow T, Conlon A, et al. Risk of Venous Thromboembolism Following Peripherally Inserted Central Catheter Exchange: An Analysis of 23,000 Hospitalized Patients. Am J Med. 2018;131(6):651-60.

25. Chopra V, Montoya A, Joshi D, Becker C, Brant A, McGuirk H, et al. Peripherally Inserted Central Catheter Use in Skilled Nursing Facilities: A Pilot Study. J Am Geriatr Soc. 2015;63(9):1894-9.

26. Cimala I, Grosicki S, Barchnicka A, Krupa-Kotara K. Evaluation on inflammatory states of peripheral veins connected with cannulation. Przegl Epidemiol. 2018;72(2):205-13.

27. Coyer B, Carlucci M. Reducing Central Line Utilization by Peripherally Infusing Vasopressors. Dimensions of Critical Care Nursing. 2023;42(3):131-6.

28. Crowell J, O'Neil K, Drager L. Project HANDS: A Bundled Approach to Increase Short Peripheral Catheter Dwell Time. J Infus Nurs. 2017;40(5):274-80.

29. Crowley JJ, Pereira JK, Harris LS, Becker CJ. Peripherally inserted central catheters: experience in 523 children. Radiology. 1997;204(3):617-21.

30. Decker K, Ireland S, O'Sullivan L, Boucher S, Kite L, Rhodes D, et al. Peripheral intravenous catheter insertion in the Emergency Department. Australas Emerg Nurs J. 2016;19(3):138-42.

31. DeVries M, Sleweon T. Bridging the Gap: Introduction of an Antimicrobial Peripherally Inserted Central Catheter (PICC) in Response to High PICC Central Line-Associated Bloodstream Infection Incidence. JAVA - Journal of the Association for Vascular Access. 2021;26(2):7-30.

32. Diwakar K, Kumar S, Srivastava P, Uddin MW, Mishra S. Reduction in the incidence of infusion-related phlebitis in a pediatric critical care unit of Eastern India: A quality improvement initiative. Med J Armed Forces India. 2024;80(1):46-51.

33. Dreimanis D, Beckingham W, Collignon P, Roberts J. Staphylococcus aureus bacteraemia surveillance: a relatively easy to collect but accurate clinical indicator on serious health-care associated infections and antibiotic resistance. Australian Infection Control. 2005;10(4):127-30.

34. Eren H, Caliskan N. Effect of a Vein Imaging Device and of Fist Clenching on Determination of an Appropriate Vein and on Catheter Placement Time in Patients Receiving Chemotherapy: A Randomized Controlled Trial. Cancer Nurs. 2022;45(2):105-12.

35. Erhard DM, Nguyen S, Guy KJ, Casalaz DM, Konig K. Dwell times and risk of non-elective removal of 1-French peripherally inserted central catheters according to catheter tip position in very preterm infants. Eur J Pediatr. 2017;176(3):407-11.

36. Farkas JC, Liu N, Bleriot JP, Chevret S, Goldstein FW, Carlet J. Single- versus triple-lumen central catheter-related sepsis: a prospective randomized study in a critically ill population. Am J Med. 1992;93(3):277-82.

37. Fillman KM, Ryder JH, Brailita DM, Rupp ME, Cavalieri RJ, Fey PD, et al. Disinfection of vascular catheter connectors that are protected by antiseptic caps is unnecessary. Infect Control Hosp Epidemiol. 2024;45(1):35-9.

38. Fohlen A, Briant AR, Dutheil JJ, Le Pennec V, Pelage JP, Parienti JJ. Complications of peripherally inserted central catheters in adult hospitalized patients and outpatients in the KTFIXPICC study: A randomized controlled trial evaluating a fixation device KT FIX Plussystem. Am J Infect Control. 2022;50(8):916-21.

39. Fong NI, Holtzman SR, Bettmann MA, Bettis SJ. Peripherally inserted central catheters: outcome as a function of the operator. J Vasc Interv Radiol. 2001;12(6):723-9.

40. Geffers C, Baerwolff S, Schwab F, Gastmeier P. Incidence of healthcare-associated infections in high-risk neonates: results from the German surveillance system for very-low-birthweight infants. J Hosp Infect. 2008;68(3):214-21.

41. Gellert GA, Ewert DP, Bendana N, Smith E, Beck-Sague C, Chin A, et al. A cluster of coagulase-negative staphylococcal bacteremias associated with peripheral vascular catheter colonization in a neonatal intensive care unit. Am J Infect Control. 1993;21(1):16-20.

42. Goes-Silva E, Abreu TF, Frota AC, Pessoa-Silva CL, Cunha AJ, Hofer CB. Use of peripherally inserted central catheters to prevent catheter-associated bloodstream infection in children. Infect Control Hosp Epidemiol. 2009;30(10):1024-6.

43. Goodfriend L, Kennedy S, Hein A, Baker R. Implementation of a Vascular Access Experience Program to Train Unit-Based Vascular Access Champions. J Infus Nurs. 2020;43(4):193-9.

44. Grasso F, Capasso A, Pacella D, Borgia F, Salome S, Capasso L, et al. Ultrasound Guided Catheter Tip Location in Neonates: A Prospective Cohort Study. J Pediatr. 2022;244:86-91 e2.

45. Grove JR, Pevec WC. Venous thrombosis related to peripherally inserted central catheters. J Vasc Interv Radiol. 2000;11(7):837-40.

46. Hamaba H, Miyata Y, Wada T, Hayasaka T, Hayashi Y. An analysis of prior experience influencing quality of pulmonary artery catheter placement in residents. Ann Card Anaesth. 2020;23(2):161-4.

47. Harlan MD, Kennell JS, Lucas W, Ren D, Tuite PK. A Clinical Nurse Specialist–Led Quality Improvement Initiative to Identify Barriers to Adherence to a Bundle for Central Line Maintenance. Clin Nurse Spec. 2022;36(2):99-108.

48. Hassan MS, Fauzi MH, Mdnoh AY, Abdullah AA, Md Nor J, Yaacob N, et al. Effectiveness of Ice Compression to Reduce Pain among Primary School Children Venipuncture and Peripheral Intravenous Cannulation in Emergency Department North-Eastern Malaysia. International Medical Journal. 2022;29(1):34-7.

49. Hawkins T, Greenslade JH, Suna J, Williams J, Rickard CM, Jensen M, et al. Peripheral Intravenous Cannula Insertion and Use in the Emergency Department: An Intervention Study. Acad Emerg Med. 2018;25(1):26-32.

50. He S, Wang J, Zhang X, Xie J, Wan Q, He R, et al. A Comparison of In Vitro Measurement and Ultrasound for Peripherally Inserted Central Catheter Placement in Premature Infants: A Before-and-After Self-Controlled Prospective Study. Cureus. 2024;16(3):e56335.

51. Helton J, Hines A, Best J. Peripheral IV Site Rotation Based on Clinical Assessment vs. Length of Time Since Insertion. Medsurg Nurs. 2016;25(1):44-9.

52. Hendy R. Auditing PICC line management. Nurs Times. 2001;97(38):32-3.

53. Hill ML, Baldwin L, Slaughter JC, Walsh WF, Weitkamp JH. A silver-alginate-coated dressing to reduce peripherally inserted central catheter (PICC) infections in NICU patients: a pilot randomized controlled trial. J Perinatol. 2010;30(7):469-73.

54. Hoffer EK, Borsa J, Santulli P, Bloch R, Fontaine AB. Prospective randomized comparison of valved versus nonvalved peripherally inserted central vein catheters. AJR Am J Roentgenol. 1999;173(5):1393-8.

55. Holder MR, Stutzman SE, Olson DM. Impact of Ultrasound on Short Peripheral Intravenous Catheter Placement on Vein Thrombosis Risk. J Infus Nurs. 2017;40(3):176-82.

56. Hosseini SJ, Eidy F, Kianmehr M, Firouzian AA, Hajiabadi F, Marhamati M, et al. Comparing the Effects of Pulsatile and Continuous Flushing on Time and Type of Peripheral Intravenous Catheters Patency: A Randomized Clinical Trial. J Caring Sci. 2021;10(2):84-8.

57. Huang V, Ruhe JJ, Lerner P, Fedorenko M. Risk factors for readmission in patients discharged with outpatient parenteral antimicrobial therapy: a retrospective cohort study. BMC Pharmacol Toxicol. 2018;19(1):50.

58. Hynes JP, Murray AS, Murray OM, Eustace SK, Gilchrist S, Dolan A, et al. Use of Lean Six Sigma methodology shows reduction of inpatient waiting time for peripherally inserted central catheter placement. Clin Radiol. 2019;74(9):733 e5- e9.

59. Janes M, Kalyn A, Pinelli J, Paes B. A randomized trial comparing peripherally inserted central venous catheters and peripheral intravenous catheters in infants with very low birth weight. J Pediatr Surg. 2000;35(7):1040-4.

60. Jimenez N, Bradford H, Seidel KD, Sousa M, Lynn AM. A comparison of a needle-free injection system for local anesthesia versus EMLA for intravenous catheter insertion in the pediatric patient. Anesth Analg. 2006;102(2):411-4.

61. Kagan E, Salgado CD, Banks AL, Marculescu CE, Cantey JR. Peripherally inserted central catheter-associated bloodstream infection: Risk factors and the role of antibiotic-impregnated catheters for prevention. Am J Infect Control. 2019;47(2):191-5.

62. Kamala F, Boo NY, Cheah FC, Birinder K. Randomized controlled trial of heparin for prevention of blockage of peripherally inserted central catheters in neonates. Acta Paediatr. 2002;91(12):1350-6.

63. Kaplan AV. Infusion sleeve catheter. Semin Interv Cardiol. 1996;1(1):36-8.

64. Kearney L, Craswell A, Massey D, Marsh N, Nugent R, Alexander C, et al. Peripheral intravenous catheter management in childbirth (PICMIC): A multi-centre, prospective cohort study. J Adv Nurs. 2021;77(11):4451-8.

65. Kemp L, Burge J, Choban P, Harden J, Mirtallo J, Flancbaum L. The effect of catheter type and site on infection rates in total parenteral nutrition patients. JPEN J Parenter Enteral Nutr. 1994;18(1):71-4.

66. Kerwat K, Eberhart L, Kerwat M, Horth D, Wulf H, Steinfeldt T, et al. Chlorhexidine gluconate dressings reduce bacterial colonization rates in epidural and peripheral regional catheters. Biomed Res Int. 2015;2015:149785.

67. Kim-Saechao SJ, Almario E, Rubin ZA. A novel infection prevention approach: Leveraging a mandatory electronic communication tool to decrease peripherally inserted central catheter infections, complications, and cost. Am J Infect Control. 2016;44(11):1335-45.

68. Klenner AF, Fusch C, Rakow A, Kadow I, Beyersdorff E, Eichler P, et al. Benefit and risk of heparin for maintaining peripheral venous catheters in neonates: a placebo-controlled trial. J Pediatr. 2003;143(6):741-5.

69. Konjević S, Djukić D, Stanimirović B, Blagojević A, Bobić V, Banja B. Peripherally inserted central catheter complications in neonates – our experiences. Signa Vitae. 2015;10(S1):16-9.

70. Kurata Y, Ohira G, Hayano K, Imanishi S, Tochigi T, Takahashi Y, et al. Peripherally inserted central catheter securement with cyanoacrylate glue and bloodstream infection: A retrospective cohort study. JPEN J Parenter Enteral Nutr. 2024;48(2):215-23.

71. Lago P, Tiozzo C, Boccuzzo G, Allegro A, Zacchello F. Remifentanil for percutaneous intravenous central catheter placement in preterm infant: a randomized controlled trial. Paediatr Anaesth. 2008;18(8):736-44.

72. Leroy O, Billiau V, Beuscart C, Santre C, Chidiac C, Ramage C, et al. Nosocomial infections associated with long-term radial artery cannulation. Intensive Care Med. 1989;15(4):241-6.

73. Li MY, Yu CW, Yang YC, Chang CC. [Reducing the pain of intravenous injections in preschool children]. Hu Li Za Zhi. 2014;61(2 Suppl):S68-75.

74. Li S, Lu H. Functions of Heparin Sodium Injection in the Prevention of Peripherally Inserted Central Catheter-Related Venous Thrombosis in NSCLC Patients during Postoperative Chemotherapy. Comput Math Methods Med. 2022;2022:1239058.

75. Lilly M, Meyer T, Braun R, Kurz E, Nelson R, Lilly C, et al. The effect of operator experience on peripherally inserted central catheter (PICC) placement fluoroscopy time in a single residency program over a 4-year period. Journal of Vascular and Interventional Radiology. 2017;28(2):S22-S.

76. Lim ZJ, Nagle D, McAllan F, Ramanan R, Dendle C, Stuart RL, et al. Evaluating the sustained effectiveness of a multimodal intervention aimed at influencing PIVC insertion practices in the emergency department. Emerg Med J. 2020;37(7):444-9.

77. Lisova K, Pavelkova K, Matejckova T, Simkova P, Hloch O, Charvat J. The difficult PICC insertion is associated with the significant increase of complications in 1 month follow-up. J. 2024:11297298241229868.

78. Liu X, Tao S, Ji H, Chen S, Gu Y, Jin X. Risk factors for peripherally inserted central catheter (PICC)-associated infections in patients receiving chemotherapy and the preventive effect of a self-efficacy intervention program: a randomized controlled trial. Ann Palliat Med. 2021;10(9):9398-405.

79. Liu YC, Seydou T, Sadio Y, Liang TZ, Ge J. [Comparative study of complications related to the use of peripheral venous catheter with and without closed system with heparin cap]. Pan Afr Med J. 2015;21:302.

80. Lopez V, Molassiotis A, Chan WK, Ng F, Wong E. An intervention study to evaluate nursing management of peripheral intravascular devices. J Infus Nurs. 2004;27(5):322-31.

81. Lucet JC, Bouadma L, Zahar JR, Schwebel C, Geffroy A, Pease S, et al. Infectious risk associated with arterial catheters compared with central venous catheters. Crit Care Med. 2010;38(4):1030-5.

82. Maezawa T, Sakuraya M, Yoshida K. The safety of peripherally inserted central venous catheters in critically ill patients: A retrospective observational study. J. 2023.

83. Maughan NM, Kim H, Hao Y, Unangst S, Roach MC, Jr., Garcia-Ramirez JL, et al. Initial experience and lessons learned with implementing Lutetium-177-dotatate radiopharmaceutical therapy in a radiation oncology-based program. Brachytherapy. 2021;20(1):237-47.

84. Maynard EC, Oh W. Topical nitroglycerin ointment as an aid to insertion of peripheral venous catheters in neonates. J Pediatr. 1989;114(3):474-6.

85. McHugh SM, Corrigan MA, Dimitrov BD, Cowman S, Tierney S, Hill AD, et al. Preventing infection in general surgery: improvements through education of surgeons by surgeons. J Hosp Infect. 2011;78(4):312-6.

86. McIntyre C, August D, Cobbald L, Lack G, Takashima M, Foxcroft K, et al. Neonatal Vascular Access Practice and Complications: An Observational Study of 1,375 Catheter Days. J Perinat Neonatal Nurs. 2023;37(4):332-9.

87. Michaud C, Champagne J, Laporte L. [Choosing a vascular catheter in oncology: listening to the patient]. Perspective Infirmiere. 2011;8(2):24-8.

88. Mjahed K, Sadraoui A, Benslama A, Idali B, Benaguida M. [Combination of Emla cream and nitrous oxide for venous cannulation in children]. Ann Fr Anesth Reanim. 1997;16(5):488-91.

89. Moayedi S, Witting M, Hirshon JM, George N, Burke A, Schenkel S. Prospective, randomized controlled comparison of a flash-tip catheter and a traditional intravenous catheter in an urban emergency department. J. 2018;19(4):387-91.

90. Montano JD. [Implantation of the passive safety catheter in the Jerez Hospital]. Rev Enferm. 2007;30(10):37-9.

91. Moppett IK, Szypula K, Yeoman PM. Comparison of EMLA and lidocaine iontophoresis for cannulation analgesia. Eur J Anaesthesiol. 2004;21(3):210-3.

92. Nieto-Rodriguez JA, Garcia-Martin MA, Barreda-Hernandez MD, Hervas MJ, Cano-Real O. Heparin and infusion phlebitis: a prospective study. Ann Pharmacother. 1992;26(10):1211-4.

93. Ntelezos K, Katsoulas T, Mariolis-Sapsakos T, Galanis P, Alexandrou E, Konstantinou E. Comparing Safety and Colonization Rates Between Octyl-Isocyanacrylate Glue and Standard Gauze Sponge Dressings for Patients with PICC-PORTs: A Pilot Randomized Controlled Trial. International Journal of Caring Sciences. 2022;15(2):1275-82.

94. Nyika ML, Mukona D, Zvinavashe M. Factors Contributing to Phlebitis Among Adult Patients Admitted in the Medical-Surgical Units of a Central Hospital in Harare, Zimbabwe. J Infus Nurs. 2018;41(2):96-102.

95. Oliveira TGP, Marcatto JO, Corrêa ADR, Santos LMD, Rocha PK, Simão DADS, et al. Compliance with central venous catheter infection prevention practices after intervention with simulation. Rev Bras Enferm. 2023;76(4):e20220574.

96. Oto J, Nishimura M, Morimatsu H, Katayama H, Onodera M, Takahashi H, et al. Comparison of contamination between conventional three-way stopcock and needleless injection device: a randomized controlled trial. Med Sci Monit. 2007;13(10):CR417-21.

97. Ouriel K, Kandarpa K, Schuerr DM, Hultquist M, Hodkinson G, Wallin B. Prourokinase versus urokinase for recanalization of peripheral occlusions, safety and efficacy: the PURPOSE trial. J Vasc Interv Radiol. 1999;10(8):1083-91.

98. Park J, Chang SJ. Effect of Skin Disinfection according to the Disinfection Frequency in Peripheral Intravenous Therapy. Journal of Korean Academy of Fundamentals of Nursing. 2020;27(4):366-74.

99. Pichler J, Soothill J, Hill S. Reduction of blood stream infections in children following a change to chlorhexidine disinfection of parenteral nutrition catheter connectors. Clin Nutr. 2014;33(1):85-9.

100. Piersigilli F, Iacona G, Yazami S, Carkeek K, Hocq C, Auriti C, et al. Cyanoacrylate glue as part of a new bundle to decrease neonatal PICC-related complications. Eur J Pediatr. 2023;182(12):5607-13.

101. Pittiruti M, Scoppettuolo G, Emoli A, Dolcetti L, Migliorini I, Lagreca A, et al. Parenteral nutrition through ultrasound-placed PICCs and midline catheters is associated with a low rate of complications: an observational study. Nutritional Therapy & Metabolism. 2009;27(3):142-8.

102. Prates Vigna C, de Carvalho Jericó M. WELFARE INDICATOR: A COMPARATIVE STUDY BETWEEN PUNCTURE AND FIXATION TECHNOLOGIES FOR PERIPHERAL INTRAVENOUS CATHETER. Journal of Nursing UFPE / Revista de Enfermagem UFPE. 2016;10(9):3384-92.

103. Prince K, Summers L, Knight MA. Needleless i.v. therapy: comparing three systems for safety. Nurs Manage. 1994;25(3):80N, P.

104. Puri P. Total parenteral nutrition in the newborn using peripheral veins: role of IV nursing team. Zeitschrift für Kinderchirurgie. 1982;37(10):50-2.

105. Reynolds J. Comparison of percutaneous venous catheters and teflon catheters for intravenous therapy in neonates. Neonatal Netw. 1993;12(5):33-9.

106. Ribeiro RCH, de Oliveira GAS, Ribeiro DF, Cesarino CB, Martins MI, Oliveira SAC. Survey about infection at the site of a double-lumen catheter insertion...World Congress of Nephrology Nursing, São Paulo, April 22 to April 25, 2007. Acta Paulista de Enfermagem. 2008;21:212-5.

107. Rickard CM, Marsh NM, Larsen EN, McGrail MR, Graves N, Runnegar N, et al. Effect of infusion set replacement intervals on catheter-related bloodstream infections (RSVP): a randomised, controlled, equivalence (central venous access device)-non-inferiority (peripheral arterial catheter) trial. Lancet. 2021;397(10283):1447-58.

108. Rijnders BJ, Van Wijngaerden E, Wilmer A, Peetermans WE. Use of full sterile barrier precautions during insertion of arterial catheters: a randomized trial. Clin Infect Dis. 2003;36(6):743-8.

109. Riveros-Perez E, Albo C, Guzzo E, Sanchez MG, Yang N, Rocuts A. Utility of color flow Doppler ultrasound to identify peripheral intravenous catheter position in adult surgical patients. SAGE Open Med. 2020;8:2050312120912123.

110. Robazzi ML, Bechelli MH, Levy CE, Moriya TM. [Intravenous catheters--a study of bacteriological conditions and evaluation of nursing care]. Rev Bras Enferm. 1984;37(1):18-25.

111. Roberts GW, Holmes MD, Staugas RE, Day RA, Finlay CF, Pitcher A. Peripheral intravenous line survival and phlebitis prevention in patients receiving intravenous antibiotics: heparin/hydrocortisone versus in-line filters. Ann Pharmacother. 1994;28(1):11-6.

112. Ross VM, Guenter P, Corrigan ML, Kovacevich D, Winkler MF, Resnick HE, et al. Central venous catheter infections in home parenteral nutrition patients: Outcomes from Sustain: American Society for Parenteral and Enteral Nutrition's National Patient Registry for Nutrition Care. Am J Infect Control. 2016;44(12):1462-8.

113. Rowe MS, Arnold K, Spencer TR. Catheter securement impact on PICC-related CLABSI: A university hospital perspective. Am J Infect Control. 2020;48(12):1497-500.

114. Ryu J, Yu M. Virtual Reality Simulation for Advanced Infection Control Education in Neonatal Intensive Care Units: Focusing on the Prevention of Central Line-Associated Bloodstream Infections and Ventilator-Associated Infections. Healthcare (Basel). 2023;11(16):14.

115. Saju AS, Prasad L, Reghuraman M, Karl Sampath I. Use of vein-viewing device to assist intravenous cannulation decreases the time and number of attempts for successful cannulation in pediatric patients. Paediatr Neonatal Pain. 2019;1(2):39-44.

116. Sargent J, Nixon E. I.v. access options for AIDS patients with cytomegalovirus disease. Br J Nurs. 1997;6(10):543-6, 8, 50-3.

117. Sargent J, Nixon E. I.v. access options for AIDS patients with cytomegalovirus disease. Br J Nurs. 1997;6(10):543-6, 8, 50-3.

118. Schelonka RL, Scruggs S, Nichols K, Dimmitt RA, Carlo WA. Sustained reductions in neonatal nosocomial infection rates following a comprehensive infection control intervention. J Perinatol. 2006;26(3):176-9.

119. Schettini F, Ferrario L, Foglia E, Garagiola E, Parodi L, Cavagnaro P, et al. The implementation of a standardized optimal procedure for peripheral venous catheters' management: Results from a multi-dimensional assessment. PLoS ONE. 2022;17(1):e0263227.

120. Schweikert W, Herlitz J, Pohlman AS, Gehlbach BK, Hall JB, Kress JP. A randomized controlled trial evaluating the utility of ultrasound confirmation of peripherally inserted central catheter placement. Proceedings of the American Thoracic Society. 2006:A295 [Poster 703].

121. Sengupta A, Lehmann C, Diener-West M, Perl TM, Milstone AM. Catheter duration and risk of CLA-BSI in neonates with PICCs. Pediatrics. 2010;125(4):648-53.

122. Sertic AJ, Connolly BL, Temple MJ, Parra DA, Amaral JG, Lee KS. Perforations associated with peripherally inserted central catheters in a neonatal population. Pediatr Radiol. 2018;48(1):109-19.

123. Sheppard K, LeDesma M, Morris NL, O'Connor K. A prospective study of two intravenous catheter securement techniques in a skilled nursing facility. J Intraven Nurs. 1999;22(3):151-6.

124. Sherrod J, Warner B, Altimier L. Designing and monitoring an RN-based PICC team. Neonat Intensive Care. 2004;17(2):19-21.

125. Shu-Juan LI, Ke Z, Huan-Huan W, Li-Ling LI, Yun CAO, Wen-Hao Z, et al. Peripherally inserted central venous catheter-related thrombosis in a neonate. Chinese Journal of Contemporary Pediatrics. 2023(12):658-62.

126. Strumpfer AL. Lower incidence of peripheral catheter complications by the use of elastomeric hydrogel catheters in home intravenous therapy patients. J Intraven Nurs. 1991;14(4):261-7.

127. Surov AV, Zyrianov SK. [Comparison of clinical and economic performance of peripheral venous catheters]. Khirurgiia (Mosk). 2012(9):82-6.

128. Swaminathan L, Flanders S, Rogers M, Calleja Y, Snyder A, Thyagarajan R, et al. Improving PICC use and outcomes in hospitalised patients: an interrupted time series study using MAGIC criteria. BMJ Qual Saf. 2018;27(4):271-8.

129. Tagliari AP, Kochi AN, Mastella B, Saadi RP, di Leoni Ferrari A, Saadi EK, et al. Axillary vein puncture guided by ultrasound vs cephalic vein dissection in pacemaker and defibrillator implant: A multicenter randomized clinical trial. Heart Rhythm. 2020;17(9):1554-60.

130. Tan PC, Mackeen A, Khong SY, Omar SZ, Noor Azmi MA. Peripheral Intravenous Catheterisation in Obstetric Patients in the Hand or Forearm Vein: A Randomised Trial. Sci Rep. 2016;6:23223.

131. Taylor T, Massaro A, Williams L, Doering J, McCarter R, He J, et al. Effect of a dedicated percutaneously inserted central catheter team on neonatal catheter-related bloodstream infection. Adv Neonatal Care. 2011;11(2):122-8.

132. Thibodeau S, Riley J, Rouse KB. Effectiveness of a new flushing and maintenance policy using peripherally inserted central catheters for adults: best practice. J Infus Nurs. 2007;30(5):287-92.

133. Thibodeau S, Riley J, Rouse KB. Effectiveness of a new flushing and maintenance policy using peripherally inserted central catheters for adults: best practice. J Infus Nurs. 2007;30(5):287-92.

134. Thorpe M, Berry W, Soper J. Duration of peripheral intravenous catheter patency in children. Paediatr child health. 2021;26(1):32-4.

135. Tighe MJ, Wong C, Martin IG, McMahon MJ. Do heparin, hydrocortisone, and glyceryl trinitrate influence thrombophlebitis during full intravenous nutrition via a peripheral vein? JPEN J Parenter Enteral Nutr. 1995;19(6):507-9.

136. Tomazoni A, Rocha PK, Pedreira M, Rodrigues EDC, Manzo BF, Santos LMD. Methods for measuring venous peripherally inserted central catheters in newborns. Rev Bras Enferm. 2021;75(2):e20210045.

137. Tran M, Shein SL, Ji X, Ahuja SP. Identification of a "VTE-rich" population in pediatrics - Critically ill children with central venous catheters. Thromb Res. 2018;161:73-7.

138. Traore O, Allaert FA, Fournet-Fayard S, Verriere JL, Laveran H. Comparison of in-vivo antibacterial activity of two skin disinfection procedures for insertion of peripheral catheters: povidone iodine versus chlorhexidine. J Hosp Infect. 2000;44(2):147-50.

139. Treas LS, Latinis-Bridges B. Efficacy of heparin in peripheral venous infusion in neonates. J Obstet Gynecol Neonatal Nurs. 1992;21(3):214-9.

140. Ullman AJ, August D, Kleidon T, Walker R, Marsh NM, Bulmer A, et al. Peripherally Inserted Central catheter iNnovation to reduce Infections and Clots (the PICNIC trial): a randomised controlled trial protocol. BMJ Open. 2021;11(4):e042475.

141. Ullman AJ, Takashima M, Kleidon T, Ray-Barruel G, Alexandrou E, Rickard CM. Global Pediatric Peripheral Intravenous Catheter Practice and Performance: A Secondary Analysis of 4206 Catheters. J Pediatr Nurs. 2020;50:e18-e25.

142. Vachharajani AJ, Vachharajani NA, Morris H, Niesen A, Elward A, Linck DA, et al. Reducing peripherally inserted central catheters in the neonatal intensive care unit. J Perinatol. 2017;37(4):409-13.

143. Valbousquet Schneider L, Jr., Duron S, Arnaud FX, Bousquet A, Kervella Y, Bouzad C, et al. Evaluation of PICC complications in orthopedic inpatients with bone infection for long-term intravenous antibiotics therapy. J. 2015;16(4):299-308.

144. van Rens MF, Hugill K, Mahmah MA, Francia AL, van Loon FH. Effect of peripheral intravenous catheter type and material on therapy failure in a neonatal population. J. 2023;24(6):1284-92.

145. Wang W, Zhao C, Ji Q, Liu Y, Shen G, Wei L. Prevention of peripherally inserted central line-associated blood stream infections in very low-birth-weight infants by using a central line bundle guideline with a standard checklist: a case control study. BMC Pediatr. 2015;15:69.

146. Wang XX, He Y, Chu J, Xu JS. Risk factors analysis and the establishment of nomogram prediction model for PICC-related venous thrombosis in patients with lymphoma: a double-center cohort-based case-control study. Front. 2024;14:1347297.

147. Webster J, Larsen E, Marsh N, Choudhury A, Harris P, Rickard CM. Chlorhexidine gluconate or polyhexamethylene biguanide disc dressing to reduce the incidence of central-line-associated bloodstream infection: a feasibility randomized controlled trial (the CLABSI trial). J Hosp Infect. 2017;96(3):223-8.

148. Yamamoto LG, Boychuk RB. A blinded, randomized, paired, placebo-controlled trial of 20-minute EMLA cream to reduce the pain of peripheral i.v. cannulation in the ED. Am J Emerg Med. 1998;16(7):634-6.

149. Yuan C, Zhao Q, Song X, Meng F. WITHDRAWN: Prevention of peripherally inserted central catheter-related infections in very low-birth-weight infants by using a central line bundle guideline with a standard checklist. Int J Nurs Sci. 2016;3(1):50-3.

150. Zeidman A, Ram E, Bank Y, Stein GY, Horowitz A, Dresnik Z. The efficacy and safety of MICROMAT as an intravenous site infection protector. Microbial Ecology in Health and Disease. 2009;16(4):211-3.

151. Zempsky WT, Anand KJ, Sullivan KM, Fraser D, Cucina K. Lidocaine iontophoresis for topical anesthesia before intravenous line placement in children. J Pediatr. 1998;132(6):1061-3.

152. Zerla PA, Canelli A, Cerne L, Caravella G, Gilardini A, De Luca G, et al. Evaluating safety, efficacy, and cost-effectiveness of PICC securement by subcutaneously anchored stabilization device. J. 2017;18(3):238-42.

153. Zhao J, Ruan Z, Zhao J, Yang Y, Xiao S, Ji H. Study on the timing of first dressing change with alginate dressing application in PICC placement among tumor patients. J Cancer Res Ther. 2022;18(7):2013-20.

154. Zheng Y, Zhou HJ, Tao N, Tian Y, Qin SW, Qin BY, et al. Prevention of catheter tip malposition with an ultrasound-guided finger-pressure method to block the internal jugular vein during PICC placement: a meta-analysis. Wideochir Inne Tech Maloinwazyjne. 2022;17(2):289-98.

155. Zitek T, Busby E, Hudson H, McCourt JD, Baydoun J, Slattery DE. Ultrasound-guided Placement of Single-lumen Peripheral Intravenous Catheters in the Internal Jugular Vein. West J Emerg Med. 2018;19(5):808-12.

156. 周维华, 张建美, 陈丽萍, 张秀平, 沈世银, 黄芳. 葡萄糖酸氯已定抗菌透明敷贴治疗PICC置管穿刺部位感染的效果. Nursing of Integrated Traditional Chinese & Western Medicine. 2018;4(12):129-31.

157. 马丽娟, 林丽, 朱娓, 陈玲. 风险防范管理体系在宫颈癌PICC 置管 化疗病人中的应用. Chinese nursing research. 2020;34(14):2515‐9.

## X5_Ineligible comparison (n=162)

1. Trembath HE, Caruso DM, McLean SE, Akinkuotu AC, Hayes Dixon AA, Phillips MR. Central Line-Associated Bloodstream Infection Risk Factors in a Pediatric Population. Am Surg. 2024;90(1):69-74.

2. Rosich-Soteras A, Bonilla-Serrano C, Llaurado-Gonzalez MA, Fernandez-Bombin A, Trivino-Lopez JA, Barcelo-Querol L, et al. Implementation of a vascular access team and an intravenous therapy programme: A first-year activity analysis. J. 2024:11297298231220537.

3. Estrella Y, Panzlau N, Vinokur K, Ayala S, Lin M, Gaeta T, et al. Comparing contamination rates of sterile-covered and uncovered transducers for ultrasound-guided peripheral intravenous lines. Ultrasound J. 2024;16(1):6.

4. Chen L, Lu Y, Wang L, Pan Y, Zhou X. Construction of a nomogram risk prediction model for PICC-related venous thrombosis and its application. Asian Journal of Surgery. 2024;47(1):107-11.

5. Yang Y, Liu H, He M, Yang Y, Hu M, He R, et al. Multivariate analysis of medical adhesive-related skin injury at the site of peripherally inserted central catheter insertion in cancer patients: A prospective cohort study. J. 2023.

6. Venturini S, Reffo I, Avolio M, Basaglia G, Del Fabro G, Callegari A, et al. Dalbavancin in catheter-related bloodstream infections: a pilot study. Infez Med. 2023;31(2):250-6.

7. Tan KK, Hino G, Jr., Zhou AY, Al-Fayiz H, Rodriguez S, Abdul-Mutakabbir JC. Identifying the potential impact of a multidisciplinary outpatient antimicrobial therapy program in an area of high social vulnerability. Ther. 2023;10:20499361231194257.

8. Shan J, Lv S, Li H, Wang D, Zhang X, Liu W. A comparative study between two methods of delivery of chemotherapeutic agent in patients with bone and soft tissue sarcoma of lower extremity. BMC Musculoskeletal Disorders. 2023;24(1).

9. Salonen S, Tammela O, Koivisto AM, Korhonen P. Umbilically and Peripherally Inserted Thin Central Venous Catheters Have Similar Risks of Complications in Very Low-Birth-Weight Infants. Clin Pediatr (Phila). 2023;62(11):1361-8.

10. Rodríguez García A, Pérez Fernández Y, Pulido Pérez R, Lugo Castro Y, Rodríguez Hernández AJ, Valdivielso Mendi O. DESPLAZAMIENTO DE CATÉTERES VENOSOS DE PUNCIÓN ECODIRIGIDA. ESTUDIO TRANSVERSAL SOBRE LA INFLUENCIA DE LA ESTRATEGIA EFIX PARA LA SELECCIÓN DEL DISPOSITIVO DE FIJACIÓN. ENE Revista de Enfermeria. 2023;17(1):1-18.

11. Rieger MJ, Schenkel X, Dedic I, Brunn T, Gnannt R, Hofmann M, et al. Complication rates of peripherally inserted central catheters vs implanted ports in patients receiving systemic anticancer therapy: A retrospective cohort study. International Journal of Cancer. 2023;153(7):1397-405.

12. Pitiriga V, Bakalis J, Theodoridou K, Dimitroulia E, Saroglou G, Tsakris A. Comparison of microbial colonization rates between central venous catheters and peripherally inserted central catheters. Antimicrobial Resistance and Infection Control. 2023;12(1).

13. Merchaoui Z, Laudouar Q, Marais C, Morin L, Ghali N, Charbel R, et al. Ultrasound guided percutaneous catheterization of the brachiocephalic vein by small caliber catheter: An alternative to epicutaneo-caval catheter in newborn and premature infants. J. 2023;24(3):487-91.

14. Marsh N, Larsen EN, O'Brien C, Groom P, Kleidon TM, Alexandrou E, et al. Comparing the use of midline catheters versus peripherally inserted central catheters for patients requiring peripherally compatible therapies: A pilot randomised controlled trial (the compact trial). Infect Dis Health. 2023;28(4):259-64.

15. Lee HN, Lee S, Park SJ, Cho Y, Chung HH. Development and validation of an updated PICC length prediction formula based on anteroposterior chest radiographs for the ultrasound-guided bedside placement. PLoS ONE. 2023;18(11 November).

16. Larcher R, Barrigah-Benissan K, Ory J, Simon C, Beregi JP, Lavigne JP, et al. Peripherally Inserted Central Venous Catheter (PICC) Related Bloodstream Infection in Cancer Patients Treated with Chemotherapy Compared with Noncancer Patients: A Propensity-Score-Matched Analysis. Cancers. 2023;15(12):20.

17. Jang E, Son SM, Moon KY, Lee S, Han HS, Park SC, et al. Analysis of tip malposition and correction of peripherally inserted central catheters under ultrasound-guidance: 5-year outcomes from a single center. J. 2023.

18. Jaffray J, Mosha M, Branchford B, Goldenberg NA, Silvey M, Croteau SE, et al. Evaluation of venous thromboembolism risk factors reveals subtype heterogenicity in children with central venous catheters: a multicenter study from the Children's Hospital Acquired Thrombosis consortium. Journal of Thrombosis and Haemostasis. 2023;21(9):2441-50.

19. İŞCan B. İleri Derecede Preterm Bebeklerde Standart ve Antibiyotik Emdirilmiş Periferik Venöz Kateterlerin Kullanımı Üzerine Retrospektif Gözlemsel Bir Çalışma. Forbes Journal of Medicine. 2023;4(3):320-6.

20. Hess S, Poryo M, Ruckes C, Papan C, Ehrlich A, Ebrahimi-Fakhari D, et al. Assessment of an umbilical venous catheter dwell-time of 8–14 days versus 1–7 days in very low birth weight infacts (UVC – You Will See): a pilot single-center, randomized controlled trial. Early Hum Dev. 2023;179.

21. Gifford AH, Hinton AC, Jia S, Nasr SZ, Mermis JD, Lahiri T, et al. Complications and Practice Variation in the Use of Peripherally Inserted Central Venous Catheters in People With Cystic Fibrosis: The Prospective Study of Peripherally Inserted Venous Catheters in People With Cystic Fibrosis Study. Chest. 2023;164(3):614-24.

22. Frondizi F, Dolcetti L, Pittiruti M, Calabrese M, Fantoni M, Biasucci DG, et al. Complications associated with the use of peripherally inserted central catheters and midline catheters in COVID-19 patients: An observational prospective study. Am J Infect Control. 2023;51(11):1208-12.

23. Bunch J, Hanley B, Donahue D. A retrospective, comparative, clinical study of occlusion rate of peripherally inserted central catheters fabricated of poly(vinyl alcohol)-based hydrogel composite. Journal of Materials Science: Materials in Medicine. 2023;34(7).

24. Bredenberg E, Atwater R, Grimm E, Chopra V, Dale Shamburger C, Anstett TJ. Promoting appropriate midline catheter and PICC placement through implementation of an EHR-based clinical decision support tool: An interrupted time-series analysis. Journal of Hospital Medicine. 2023;18(6):483-90.

25. Menezes RP, Melo SGO, Oliveira MB, Silva FF, Alves PGV, Bessa MAS, et al. Healthcare-associated infections in high-risk neonates: Temporal trends in a national surveillance system. Early Hum Dev. 2021;158.

26. Zhang M, Kang L, Li Q. A comparative study on the use of different connectors in tube sealing in elderly tumor patients with PICC. Int J Clin Exp Med. 2017;10(6):9488‐94.

27. Chittick P, Azhar S, Movva K, Keller P, Boura JA, Band J. Early onset versus late onset peripherally inserted central venous catheter infections: an analysis of risk factors and microbiology. Infect Control Hosp Epidemiol. 2013;34(9):980-3.

28. Hentschel J, Brüngger B, Stüdi K, Mühlemann K. Prospective surveillance of nosocomial infections in a Swiss NICU: Low risk of pneumonia on nasal continuous positive airway pressure? Infection. 2005;33(5-6):350-5.

29. Tokars JI, Cookson ST, McArthur MA, Boyer CL, McGeer AJ, Jarvis WR. Prospective evaluation of risk factors for bloodstream infection in patients receiving home infusion therapy. Ann Intern Med. 1999;131(5):340-7.

30. Choudhary VS. Impact of Intermittent Saline Flushing in the Patency of Peripheral Venouscatheter among Clients in Selected Hospitals of District Muktsar, Punjab. International Journal of Nursing Education. 2013;5(2):89-96.

31. Mali PM, Chendake MB, Mohite VR. Efficacy Of Intermittent Normal Saline Flushing Of IV Cannula To Reduce The Phlebitis. Journal of Pharmaceutical Negative Results. 2022;13:1597-606.

32. Shah PS, Kalyn A, Satodia P, Dunn MS, Parvez B, Daneman A, et al. A randomized, controlled trial of heparin versus placebo infusion to prolong the usability of peripherally placed percutaneous central venous catheters (PCVCs) in neonates: the HIP (Heparin Infusion for PCVC) study. Pediatrics. 2007;119(1):e284-91.

33. Petroulias PL. Use of Electronic Tablets for Patient Education on Flushing Peripherally Inserted Central Catheters. J Infus Nurs. 2017;40(5):298-304.

34. Forni C, Loro L, Tremosini M, Trofa C, D'Alessandro F, Sabbatini T, et al. [Cohort study of peripheral catheter related complications and identification of predictive factors in a population of orthopedic patients]. Assist Inferm Ric. 2010;29(4):166-73.

35. Didagelos M, Pagiantza A, Zegkos T, Zarra K, Angelopoulos V, Kouparanis A, et al. Low Molecular Weight Heparin in Improving RAO After Transradial Coronary Catheterization: The LOW-RAO Randomized Study. JACC Cardiovasc Interv. 2022;15(16):1686-8.

36. Boyle MF, Kuntz B. Saline locks in prehospital care. Prehosp Disaster Med. 1994;9(3):190-2.

37. Baye ND, Teshome AA, Ayenew AA, Amare TJ, Mulu AT, Abebe EC, et al. Incidence, time to occurrence and predictors of peripheral intravenous cannula-related complications among neonates and infants in Northwest Ethiopia: an institutional-based prospective study. BMC Nurs. 2023;22(1):11.

38. Alizadeh A, Yazdi AH, Kafi M, Rad MA, Moradi M, Emkanjoo Z. Predictors of local venous complications resulting from electrophysiological procedures. Cardiol J. 2012;19(1):15-9.

39. Colacchio K, Deng Y, Northrup V, Bizzarro MJ. Complications associated with central and non-central venous catheters in a neonatal intensive care unit. J Perinatol. 2012;32(12):941-6.

40. Schwengel DA, McGready J, Berenholtz SM, Kozlowski LJ, Nichols DG, Yaster M. Peripherally inserted central catheters: a randomized, controlled, prospective trial in pediatric surgical patients. Anesth Analg. 2004;99(4):1038-43.

41. Tao F, Wang X, Liu J, Li J, Sui F. Perioperative application of midline catheter and PICC in Patients with gastrointestinal tumors. J BUON. 2019;24(6):2546-52.

42. Liu Z, Song X, Kong L, Liu S, Luo Z, Yu Y. Clinical application effect of ultrasound-guided implant modified mid-length catheter of peripheral venous with modified Seldinger technique. Chinese and Foreign Medical Research. 2019;17 (35):173-5.

43. Lund F, Schultz JH, Maatouk I, Krautter M, Moltner A, Werner A, et al. Effectiveness of IV cannulation skills laboratory training and its transfer into clinical practice: a randomized, controlled trial. PLoS ONE. 2012;7(3):e32831.

44. Caglar S, Buyukyilmaz F, Bakoglu I, Inal S, Salihoglu O. Efficacy of Vein Visualization Devices for Peripheral Intravenous Catheter Placement in Preterm Infants: A Randomized Clinical Trial. J Perinat Neonatal Nurs. 2019;33(1):61-7.

45. 肖宁. 复方利多卡因乳膏在儿童PICC置管中的镇痛效果观察. Nursing of Integrated Traditional Chinese & Western Medicine. 2019;5(5):121-3.

46. Waterhouse J, Bandisode V, Brandon D, Olson M, Docherty SL. Evaluation of the use of a stabilization device to improve the quality of care in patients with peripherally inserted central catheters. AACN Adv Crit Care. 2014;25(3):213-20.

47. Sumitani S, Watanabe Y. Survey of intravenous placement skills with the peripheral-short catheter: comparison of the practice by new nurses, mid-career nurses, and experienced nurses. Journal of Japan Academy of Nursing Science. 2010;30(3):61-9.

48. Fernández Menéndez AY, García Bertolo A, Orviz Rivera J, Fernández López A, Del Valle Fernandez M, González García E. Device Selection for Vascular Access Guideline implementation at a

regional hospital intensive care unit (ICU). International Journal of Integrated Care. 2019;19(4):1-2.

49. Mendoza I, Ryan PL, Villareal C, Latido M, Anicoche M, Bulacan P, et al. Trans-illumination Devices: Improving IV Insertion Accuracy and Success Rates. J Perianesth Nurs. 2022;37(4):e6-e.

50. Lorente L, Jimenez A, Martin MM, Jimenez JJ, Iribarren JL, Mora ML. Lower arterial catheter-related infection in brachial than in femoral access. Am J Infect Control. 2010;38(9):e40-2.

51. Kavakli Ö, Uzun Ş, HatİPoĞLu S. The Investigation of the Efficiency of Fixation Set in the Performing Peripheral Intravenous Catheter. Turkiye Klinikleri Journal of Nursing Sciences. 2013;5(2):79-84.

52. Ferrete-Morales C, Vazquez-Perez MA, Sanchez-Berna M, Gilabert-Cerro I, Corzo-Delgado JE, Pineda-Vergara JA, et al. [Incidence of phlebitis due to peripherally inserted venous catheters: impact of a catheter management protocol]. Enferm Clin. 2010;20(1):3-9.

53. Diaz Benito M, Sopena Vazquez MJ. Estudio sobre la posición de la cabeza: EN LA CANALIZACIÓN DE CAJÉTER VENOSO CENTRAL DE ACCESO PERIFÉRICO. Revista ROL de Enfermería. 2019;42(2):8-12.

54. Chan-Dinevski I, Annamalai G. Peripherally Inserted Central Catheters (PICCs) at the Bedside by X-ray Technologists: A Review of Our Experience. J Med Imaging Radiat Sci. 2020;51(3):373-8.

55. 杨翠, 唐梦琳, 罗玉兰, 胡琳, 宋锦平. 腔内心电图联合体外测量法定位患儿下肢 静脉PICC 尖端位置准确性和安全性的 随机对照研究. Chinese nursing research. 2021;35(24):4358‐65.

56. Kavakli Ö, Uzun Ş, HatİPoĞLu S. Periferik İntravenöz Kateter Uygulamasında Tespit Seti Etkinliğinin İncelenmesi. Turkiye klinikleri hemsirelik bilimleri. 2013;5(2):79‐84.

57. Simcock L. No Going Back: Advantages of Ultrasound-Guided Upper Arm PICC Placement. Journal of the Association for Vascular Access. 2008;13(4):191-7.

58. Fang F, Zhang HY, Wang F, Yang W, Zhang JL. Comparison of three different fixiation methods for peripherally inserted central catheter. Chinese Journal of Clinical Nutrition. 2011;19(2):119-23.

59. Ricard JD, Salomon L, Boyer A, Thiery G, Meybeck A, Roy C, et al. Central or peripheral catheters for initial venous access of ICU patients: a randomized controlled trial. Crit Care Med. 2013;41(9):2108-15.

60. Thandaveshwara D, Krishnamurthy V, Prajwala HV. Comparison of Continuous Flush with Pulse Flush Technique in Clearing Blood Contamination of Small Bore Intra Vascular Catheter: A Randomised Control Trial. Journal of Clinical and Diagnostic Research. 2018;12(8):SC09-SC11.

61. Elsobkey FA, Abd El Salam AA, Amer HMM, Elfeshawy R. Educational training using transmitted light device on nurses' performance and pain among children undergoing peripheral intravenous cannulation. Systematic Reviews in Pharmacy. 2020;11(12):2237-48.

62. Federica B, Nizar Yahya B, Hevan Al-Atroushy A, Wahida Ibraheem A, Bayar Saleem H, Bijeen Fareq J, et al. It is possible to create a vascular access team in a middle resource country? Experience of Hevi Paediatric Teaching Hospital at DUHOK - IRAQ. J. 2023;24(5):994-9.

63. Huang C, Wu Z, Huang W, Zhang X, Lin X, Luo J, et al. Identifying the impact of the Zone Insertion Method(TM) (ZIM(TM)): A randomized controlled trial. J. 2023;24(4):729-38.

64. Usman Ghani F, Mohsina S, Areej Shamim A, Mehreen Aman ALI, Shafique Ul R, Fazul UR. Incidence, Risk Factors and Prevention Related to Infusion Phlebitis. Journal of Pharmaceutical Negative Results. 2022;13:2822-5.

65. Annetta MG, Celentano D, Zumstein L, Attina G, Ruggiero A, Conti G, et al. Catheter-related complications in onco-hematologic children: A retrospective clinical study on 227 central venous access devices. J. 2024;25(2):512-8.

66. Wilson GM, Winsett RP, Modi B, Jia R, Patton T, Silberberg D. Comparative intervention assessing a catheter stabilization device on peripheral intravenous line loss. J. 2024;25(1):82-8.

67. Gabel KS, Geelhoed GW, Zalkind DL. A comparative study of a new skin preparation method for peripheral intravenous lines. Am Surg. 1988;54(5):307-10.

68. Covey M, McLane C, Smith N, Matasic J, Holm K. Infection related to intravascular pressure monitoring: effects of flush and tubing changes. Am J Infect Control. 1988;16(5):206-13.

69. Lombardi TP, Gundersen B, Zammett LO, Walters JK, Morris BA. Efficacy of 0.9% sodium chloride injection with or without heparin sodium for maintaining patency of intravenous catheters in children. Clin Pharm. 1988;7(11):832-6.

70. Rutherford C. A study of single lumen peripherally inserted central line catheter dwelling time and complications. J Intraven Nurs. 1988;11(3):169-73.

71. Franceschi D, Gerding RL, Phillips G, Fratianne RB. Risk factors associated with intravascular catheter infections in burned patients: a prospective, randomized study. J Trauma. 1989;29(6):811-6.

72. Thompson D. Povidone-iodine and thrombophlebitis. Nurs Times. 1989;85(30):63.

73. Andrews JC, Marx MV, Williams DM, Sproat I, Walker-Andrews SC. The upper arm approach for placement of peripherally inserted central catheters for protracted venous access. AJR Am J Roentgenol. 1992;158(2):427-9.

74. Hanrahan KS, Kleiber C, Fagan CL. Evaluation of saline for i.v. locks in children. Pediatr Nurs. 1994;20(6):549-52.

75. BeVier PA, Rice CE. Initiating a pediatric peripherally inserted central catheter and midline catheter program. J Intraven Nurs. 1994;17(4):201-5.

76. Fry DE, Fry RV, Borzotta AP. Nosocomial blood-borne infection secondary to intravascular devices. Am J Surg. 1994;167(2):268-72.

77. Lawson RA, Smart NG, Gudgeon AC, Morton NS. Evaluation of an amethocaine gel preparation for percutaneous analgesia before venous cannulation in children. Br J Anaesth. 1995;75(3):282-5.

78. Cardella JF, Cardella K, Bacci N, Fox PS, Post JH. Cumulative experience with 1,273 peripherally inserted central catheters at a single institution. J Vasc Interv Radiol. 1996;7(1):5-13.

79. Sacchetti AD, Carraccio C. Subcutaneous lidocaine does not affect the success rate of intravenous access in children less than 24 months of age. Acad Emerg Med. 1996;3(11):1016-9.

80. Homer LD, Holmes KR. Risks associated with 72- and 96-hour peripheral intravenous catheter dwell times. J Intraven Nurs. 1998;21(5):301-5.

81. LaRue GD. Efficacy of ultrasonography in peripheral venous cannulation. J Intraven Nurs. 2000;23(1):29-34.

82. Heath J, Jones S. Utilization of an elastomeric continuous infusion device to maintain catheter patency. J Intraven Nurs. 2001;24(2):102-6.

83. Amo Priego MD, Carmona Monge FJ, Gomez Nieves I, Bonilla Zafra G, Gordo Vidal F. [Assessment of the efficacy of the implementation of an arterial cannulation protocol as quality assurance method]. Enferm Intensiva. 2004;15(4):159-64.

84. Smith B. Peripheral intravenous catheter dwell times: a comparison of 3 securement methods for implementation of a 96-hour scheduled change protocol. J Infus Nurs. 2006;29(1):14-7.

85. Graf JM, Newman CD, McPherson ML. Sutured securement of peripherally inserted central catheters yields fewer complications in pediatric patients. JPEN J Parenter Enteral Nutr. 2006;30(6):532-5.

86. Linck DA, Donze A, Hamvas A. Neonatal peripherally inserted central catheter team. Evolution and outcomes of a bedside-nurse-designed program. Adv Neonatal Care. 2007;7(1):22-9.

87. Salles FT, Santos VL, Secoli SR, Aron S, Debbio CB, Baptista CC, et al. A comparison and cost-effectiveness analysis of peripheral catheter dressings. Ostomy Wound Manage. 2007;53(9):26-33.

88. Katsogridakis YL, Seshadri R, Sullivan C, Waltzman ML. Veinlite transillumination in the pediatric emergency department: a therapeutic interventional trial. Pediatr Emerg Care. 2008;24(2):83-8.

89. Lobo BL, Vaidean G, Broyles J, Reaves AB, Shorr RI. Risk of venous thromboembolism in hospitalized patients with peripherally inserted central catheters. J Hosp Med. 2009;4(7):417-22.

90. Liu H, Han T, Zheng Y, Tong X, Piao M, Zhang H. Analysis of complication rates and reasons for nonelective removal of PICCs in neonatal intensive care unit preterm infants. J Infus Nurs. 2009;32(6):336-40.

91. Bausone-Gazda D, Lefaiver CA, Walters SA. A randomized controlled trial to compare the complications of 2 peripheral intravenous catheter-stabilization systems. J Infus Nurs. 2010;33(6):371-84.

92. De Paula DH, Tura BR, Lamas Cda C. Adverse events related to intravenous antibiotic therapy: a prospective observational study in the treatment of infective endocarditis. BMJ Open. 2012;2(5).

93. Maiocco G, Coole C. Use of ultrasound guidance for peripheral intravenous placement in difficult-to-access patients: advancing practice with evidence. J Nurs Care Qual. 2012;27(1):51-5.

94. Tsai MH, Hsu JF, Lien R, Huang HR, Chiang CC, Chu SM, et al. Catheter management in neonates with bloodstream infection and a percutaneously inserted central venous catheter in situ: removal or not? Am J Infect Control. 2012;40(1):59-64.

95. Barr DA, Semple L, Seaton RA. Self-administration of outpatient parenteral antibiotic therapy and risk of catheter-related adverse events: a retrospective cohort study. Eur J Clin Microbiol Infect Dis. 2012;31(10):2611-9.

96. Timsit JF, Mimoz O, Mourvillier B, Souweine B, Garrouste-Orgeas M, Alfandari S, et al. Randomized controlled trial of chlorhexidine dressing and highly adhesive dressing for preventing catheter-related infections in critically ill adults. Am J Respir Crit Care Med. 2012;186(12):1272-8.

97. Webster J, McGrail M, Marsh N, Wallis MC, Ray-Barruel G, Rickard CM. Postinfusion Phlebitis: Incidence and Risk Factors. Nurs Res Pract. 2015;2015:691934.

98. Yang Q, Lei S. Alginate Dressing Application in Hemostasis After Using Seldinger Peripherally Inserted Central Venous Catheter in Tumor Patients. Indian J Hematol Blood Transfus. 2015;31(4):434-8.

99. Ozkiraz S, Gokmen Z, Anuk Ince D, Akcan AB, Kilicdag H, Ozel D, et al. Peripherally inserted central venous catheters in critically ill premature neonates. J. 2013;14(4):320-4.

100. Irie T. Useful techniques for ultrasonography-guided free-hand puncture of a deep peripheral vein and catheter placement to obtain CT angiography. Jpn J Radiol. 2013;31(11):770-4.

101. Bai XH, Zang S, Yu L. A comparison of two intravenous infusion devices in lung carcinoma patients receiving combined radiotherapy and chemotherapy. J Cancer Res Ther. 2013;9(4):664-7.

102. Sharp R, Esterman A, McCutcheon H, Hearse N, Cummings M. The safety and efficacy of midlines compared to peripherally inserted central catheters for adult cystic fibrosis patients: a retrospective, observational study. Int J Nurs Stud. 2014;51(5):694-702.

103. Giangregorio M, Mott S, Tong E, Handa S, Gauvreau K, Connor JA. Management of peripherally inserted central catheters (PICC) in pediatric heart failure patients receiving continuous inotropic support. J Pediatr Nurs. 2014;29(4):e3-9.

104. Laudenbach N, Braun CA, Klaverkamp L, Hedman-Dennis S. Peripheral i.v. stabilization and the rate of complications in children: an exploratory study. J Pediatr Nurs. 2014;29(4):348-53.

105. Caparas JV, Hu JP. Safe administration of vancomycin through a novel midline catheter: a randomized, prospective clinical trial. J. 2014;15(4):251-6.

106. Kawamura H, Takahashi N, Takahashi M, Taketomi A. Differences in microorganism growth on various dressings used to cover injection sites: inspection of the risk of catheter-related bloodstream infections caused by Gram-negative bacilli. Surg Today. 2014;44(12):2339-44.

107. Pittiruti M, Emoli A, Porta P, Marche B, DeAngelis R, Scoppettuolo G. A prospective, randomized comparison of three different types of valved and non-valved peripherally inserted central catheters. J. 2014;15(6):519-23.

108. Marsh N, Webster J, Flynn J, Mihala G, Hewer B, Fraser J, et al. Securement methods for peripheral venous catheters to prevent failure: a randomised controlled pilot trial. J. 2015;16(3):237-44.

109. Costa P, Kimura AF, Brandon DH, Paiva ED, de Camargo PP. The development of a risk score for unplanned removal of peripherally inserted central catheter in newborns. Rev Lat Am Enfermagem. 2015;23(3):475-82.

110. Nolan ME, Yadav H, Cawcutt KA, Cartin-Ceba R. Complication rates among peripherally inserted central venous catheters and centrally inserted central catheters in the medical intensive care unit. J Crit Care. 2016;31(1):238-42.

111. Zhu A, Wang T, Wen S. Peripheral intravenous catheters in situ for more than 96 h in adults: What factors affect removal? Int J Nurs Pract. 2016;22(6):529-37.

112. Gunther SC, Schwebel C, Hamidfar-Roy R, Bonadona A, Lugosi M, Ara-Somohano C, et al. Complications of intravascular catheters in ICU: definitions, incidence and severity. A randomized controlled trial comparing usual transparent dressings versus new-generation dressings (the ADVANCED study). Intensive Care Med. 2016;42(11):1753-65.

113. Rodrigues J, Dias A, Oliveira G, Farela Neves J. [Multidimensional Strategy Regarding the Reduction of Central-Line Associated Infection in Pediatric Intensive Care]. Acta Med Port. 2016;29(6):373-80.

114. Xu T, Kingsley L, DiNucci S, Messer G, Jeong JH, Morgan B, et al. Safety and utilization of peripherally inserted central catheters versus midline catheters at a large academic medical center. Am J Infect Control. 2016;44(12):1458-61.

115. Sou V, McManus C, Mifflin N, Frost SA, Ale J, Alexandrou E. A clinical pathway for the management of difficult venous access. BMC Nurs. 2017;16:64.

116. Santolim TQ, Baptista AM, Giovani AMM, Zumarraga JP, Camargo OP. Peripherally Inserted Central Catheters in Orthopedic Patients: Experience from 1023 Procedures. Acta Ortop Bras. 2018;26(3):206-10.

117. Xu L, Hu Y, Huang X, Fu J, Zhang J. Heparinized saline versus normal saline for maintaining peripheral venous catheter patency in China: An open-label, randomized controlled study. J Int Med Res. 2017;45(2):471-80.

118. Kleidon TM, Ullman AJ, Gibson V, Chaseling B, Schoutrop J, Mihala G, et al. A Pilot Randomized Controlled Trial of Novel Dressing and Securement Techniques in 101 Pediatric Patients. J Vasc Interv Radiol. 2017;28(11):1548-56 e1.

119. Chan RJ, Northfield S, Larsen E, Mihala G, Ullman A, Hancock P, et al. Central venous Access device SeCurement And Dressing Effectiveness for peripherally inserted central catheters in adult acute hospital patients (CASCADE): a pilot randomised controlled trial. Trials. 2017;18(1):458.

120. Vinograd AM, Zorc JJ, Dean AJ, Abbadessa MKF, Chen AE. First-Attempt Success, Longevity, and Complication Rates of Ultrasound-Guided Peripheral Intravenous Catheters in Children. Pediatr Emerg Care. 2018;34(6):376-80.

121. Cortese B, Rigattieri S, Aranzulla TC, Russo F, Latib A, Burzotta F, et al. Transradial versus transfemoral ancillary approach in complex structural, coronary, and peripheral interventions. Results from the multicenter ancillary registry: A study of the Italian Radial Club. Catheter Cardiovasc Interv. 2018;91(1):97-102.

122. Meng L, Nguyen CM, Patel S, Mlynash M, Caulfield AF. Association between continuous peripheral i.v. infusion of 3% sodium chloride injection and phlebitis in adults. Am J Health Syst Pharm. 2018;75(5):284-91.

123. Lam PW, Volling C, Chan T, Wiggers JB, Castellani L, Wright J, et al. Impact of Defaulting to Single-Lumen Peripherally Inserted Central Catheters on Patient Outcomes: An Interrupted Time Series Study. Clin Infect Dis. 2018;67(6):954-7.

124. Kleidon T, Ullman AJ, Zhang L, Mihala G, Chaseling B, Schoutrop J, et al. How Does Your PICCOMPARE? A Pilot Randomized Controlled Trial Comparing Various PICC Materials in Pediatrics. J Hosp Med. 2018;13(8):517-25.

125. Roszell SS, Rabinovich HB, Smith-Miller CA. Maintaining Short Peripheral Catheter Patency: A Comparison of Saline Lock Versus Continuous Infusion in the Acute Care Setting. J Infus Nurs. 2018;41(3):165-9.

126. Assis DB, Madalosso G, Padoveze MC, Lobo RD, Oliveira MS, Boszczowski I, et al. Implementation of tailored interventions in a statewide programme to reduce central line-associated bloodstream infections. J Hosp Infect. 2018;100(3):e163-e8.

127. Yu X, Yue S, Wang M, Cao C, Liao Z, Ding Y, et al. Risk Factors Related to Peripherally Inserted Central Venous Catheter Nonselective Removal in Neonates. Biomed Res Int. 2018;2018:3769376.

128. Ruegg L, Faucett M, Choong K. Emergency inserted peripheral intravenous catheters: a quality improvement project. Br J Nurs. 2018;27(14):S28-S30.

129. Marsh N, Larsen E, Genzel J, Mihala G, Ullman AJ, Kleidon T, et al. A novel integrated dressing to secure peripheral intravenous catheters in an adult acute hospital: a pilot randomised controlled trial. Trials. 2018;19(1):596.

130. Potts DA, Davis KF, Elci OU, Fein JA. A Vibrating Cold Device to Reduce Pain in the Pediatric Emergency Department: A Randomized Clinical Trial. Pediatr Emerg Care. 2019;35(6):419-25.

131. O'Malley C, Sriram S, White M, Polinski C, Seng C, Schreiber MD. Feasibility and Outcomes Associated With the Use of 2.6-Fr Double-Lumen PICCs in Neonates. Adv Neonatal Care. 2019;19(2):E3-E8.

132. Kostner R, Mairvongrasspeinten H, De Martin Polo A, Vittadello F, Ausserhofer D, Mantovan F. [Types and frequencies of complications associated with midline catheters and PICCs in a South Tyrolean district hospital: a retrospective cohort study]. Pflege. 2019;32(2):1-8.

133. Margatho AS, Ciol MA, Hoffman JM, Dos Reis PED, Furuya RK, Lima D, et al. Chlorhexidine-impregnated gel dressing compared with transparent polyurethane dressing in the prevention of catheter-related infections in critically ill adult patients: A pilot randomised controlled trial. Aust Crit Care. 2019;32(6):471-8.

134. Shokoohi H, Boniface KS, Kulie P, Long A, McCarthy M. The Utility and Survivorship of Peripheral Intravenous Catheters Inserted in the Emergency Department. Ann Emerg Med. 2019;74(3):381-90.

135. Roberts JS, Niu J, Alexander C, Pastor-Cervantes JA. Real-Time Ultrasound-Guided Venous Access of the Arm for Right Heart Catheterization. J Invasive Cardiol. 2019;31(7):E170-E6.

136. Dickson HG, Flynn O, West D, Alexandrou E, Mifflin N, Malone M. A Cluster of Failures of Midline Catheters in a Hospital in the Home Program: A Retrospective Analysis. J Infus Nurs. 2019;42(4):203-8.

137. Price J, Xiao J, Tausch K, Hang B, Bahl A. Single Versus Double Tourniquet Technique for Ultrasound-Guided Venous Catheter Placement. West J Emerg Med. 2019;20(5):719-25.

138. Stuckey C, Curtis MP. Development of a nurse-led ultrasound-guided peripheral intravenous program. J Vasc Nurs. 2019;37(4):246-9.

139. Seo H, Altshuler D, Dubrovskaya Y, Nunnally ME, Nunn C, Ello N, et al. The Safety of Midline Catheters for Intravenous Therapy at a Large Academic Medical Center. Ann Pharmacother. 2020;54(3):232-8.

140. Saliba P, Cuervo G, Hornero A, De Carli G, Marani A, Puro V, et al. The impact of flushing with pre-filled saline syringes on the incidence of peripheral venous catheter failure: A quasi-experimental study. J. 2020;21(4):490-6.

141. Perez-Granda MJ, Bouza E, Pinilla B, Cruces R, Gonzalez A, Millan J, et al. Randomized clinical trial analyzing maintenance of peripheral venous catheters in an internal medicine unit: Heparin vs. saline. PLoS ONE. 2020;15(1):e0226251.

142. Fabiani A, Eletto V, Dreas L, Beltrame D, Sanson G. Midline or long peripheral catheters in difficult venous access conditions? A comparative study in patients with acute cardiovascular diseases. Am J Infect Control. 2020;48(10):1158-65.

143. Oh EJ, Lee JH, Kwon EJ, Min JJ. Simulation-based training using a vessel phantom effectively improved first attempt success and dynamic needle-tip positioning ability for ultrasound-guided radial artery cannulation in real patients: An assessor-blinded randomized controlled study. PLoS ONE. 2020;15(6):e0234567.

144. Chen X, Zhou L, Tan Y, Tao Z. Selection of PICC catheter location in neonates via evidence-based ACE Star model. Zhong Nan Da Xue Xue Bao Yi Xue Ban. 2020;45(9):1082-8.

145. Corley A, Ullman AJ, Marsh N, Emily NL, Mihala G, Harris PNA, et al. SECUREment bundles to prevent peripheral intravenous catheter failure-the SECURE-PIVC trial: study protocol for a pilot randomized controlled trial. Br J Nurs. 2020;29(19):S40-S6.

146. Kleidon TM, Horowitz J, Rickard CM, Ullman AJ, Marsh N, Schults J, et al. Peripherally Inserted Central Catheter Thrombosis After Placement via Electrocardiography vs Traditional Methods. Am J Med. 2021;134(2):e79-e88.

147. Gilardi E, Piano A, Chellini P, Fiori B, Dolcetti L, Pittiruti M, et al. Reduction of bacterial colonization at the exit site of peripherally inserted central catheters: A comparison between chlorhexidine-releasing sponge dressings and cyano-acrylate. J. 2021;22(4):597-601.

148. Kleinman Sween J, Lowrie A, Kirmse JM, Laughlin RK, Wodziak B, Sampathkumar P. A quality improvement project to decrease utilization of multilumen peripherally inserted central catheters. Infect Control Hosp Epidemiol. 2021;42(2):222-4.

149. Park EJ, Park K, Kim JJ, Oh SB, Jung KS, Oh SY, et al. Safety, Efficacy, and Patient Satisfaction with Initial Peripherally Inserted Central Catheters Compared with Usual Intravenous Access in Terminally Ill Cancer Patients: A Randomized Phase II Study. Cancer Res Treat. 2021;53(3):881-8.

150. Filipovich SJ, Dilgard JW, Conrad SP, Moore CB, Hefley JB. Training Program for Ultrasound-Guided Intravenous Catheter Insertion. Mil Med. 2021;186(9-10):e879-e83.

151. Kleidon TM, Schults JA, Wainwright C, Mihala G, Gibson V, Saiyed M, et al. Comparison of midline catheters and peripherally inserted central catheters to reduce the need for general anesthesia in children with respiratory disease: A feasibility randomized controlled trial. Paediatr Anaesth. 2021;31(9):985-95.

152. Takahashi T, Murayama R, Abe-Doi M, Miyahara M, Kanno C, Nakagami G, et al. Catheter failure in the administration of hyperosmotic drugs through a peripheral vein and vascular selection: A retrospective cohort study. Drug Discov Ther. 2021;15(5):236-40.

153. Li P, Sun X, Tang J, Wei CS, Zhao J. Efficacy analysis and hemodynamic changes of hematological system diseases after PICC chemotherapy. Eur Rev Med Pharmacol Sci. 2021;25(21):6566-72.

154. Anderson AP, Taroc AM, Wang X, Beardsley E, Solari P, Klein EJ. Ultrasound guided peripheral IV placement: An observational study of the learning curve in pediatric patients. J. 2022;23(2):250-6.

155. Bing S, Smotherman C, Rodriguez RG, Skarupa DJ, Ra JH, Crandall ML. PICC versus midlines: Comparison of peripherally inserted central catheters and midline catheters with respect to incidence of thromboembolic and infectious complications. Am J Surg. 2022;223(5):983-7.

156. Choi K, Keum MA, Kim MS, Kim Y, Choi S, Kyoung KH, et al. Feasibility of the Ultrasound-Guided Insertion of the Peripherally Inserted Central Catheter (PICC) by the Vascular Surgeon at the Bedside in the Trauma Intensive Care Unit. Ann Vasc Surg. 2022;80:143-51.

157. Kim SH, Hur S, Lee M, Kim HC, Jae HJ, Chung JW, et al. Outcomes of Venoplasty-Assisted, Peripherally Inserted Central Catheter Placement in Patients with Upper-Arm Venous Stenosis: Comparison with Midlines and Contralateral Placement. J Vasc Interv Radiol. 2022;33(2):189-96.

158. Swaminathan L, Flanders S, Horowitz J, Zhang Q, O'Malley M, Chopra V. Safety and Outcomes of Midline Catheters vs Peripherally Inserted Central Catheters for Patients With Short-term Indications: A Multicenter Study. JAMA Intern Med. 2022;182(1):50-8.

159. Xu YP, Shang ZR, Dorazio RM, Shi LP. Risk factors for peripherally inserted central catheterization-associated bloodstream infection in neonates. Zhongguo Dang Dai Er Ke Za Zhi. 2022;24(2):141-6.

160. Buetti N, Abbas M, Pittet D, Chraiti MN, Sauvan V, De Kraker MEA, et al. Lower risk of peripheral venous catheter-related bloodstream infection by hand insertion. Antimicrob Resist Infect Control. 2022;11(1):80.

161. Kwon S, Son SM, Lee SH, Kim JH, Kim H, Kim JY, et al. Outcomes of bedside peripherally inserted central catheter placement: a retrospective study at a single institution. Acute Crit Care. 2020;35(1):31-7.

162. Varabyeva A, Lo CP, Brancaccio A, Perissinotti AJ, Patel T, Sandison K, et al. Impact of number of lumens in central-venous catheters on central-line bloodstream infection (CLABSI) and venous thromboembolism (VTE) risk in patients with acute leukemia. Infect Control Hosp Epidemiol. 2023;44(1):125-7.

## X6_Ineligible outcome (n=150)

1. Wang P, He L, Yuan Q, Lu J, Ji Q, Peng A, et al. Risk factors for peripherally inserted central catheter-related venous thrombosis in adult patients with cancer. Thromb J. 2024;22(1):6.

2. Qi M, Qin Y, Meng S, Feng N, Meng Y. Risk factors for medical adhesive-related skin injury at the site of peripherally inserted central venous catheter placement in patients with cancer: a single-centre prospective study from China. BMJ Open. 2024;14(3):e080816.

3. Nie S, Wang L, Ma S, Sun H. Trends in the prevalence and risk factors for peripherally inserted central catheter-related complications in cancer patients from 2016 to 2022: a multicenter study. Support Care Cancer. 2024;32(4):239.

4. Gao X, Mi X, Hou S, Kang C. Analysis of factors related to thrombosis in patients with PICC placements. Medicine (Baltimore). 2024;103(5):e37168.

5. Zhu Y, Li D, Li Y, Cai W. Predictive Model for PICC Occlusion Risk for Patients in Intensive Care Units: A Retrospective Clinical Study. Alternative therapies in health and medicine. 2023;29(8):278-85.

6. Zhang J, Ma G, Peng S, Hou J, Xu R, Luo L, et al. Risk Factors and Predictive Models for Peripherally Inserted Central Catheter Unplanned Extubation in Patients With Cancer: Prospective, Machine Learning Study. J Med Internet Res. 2023;25:e49016.

7. Sun M, Zhu MMY, Zhang X, Yin X. Comparative Study of Catheter Structure Types on Venous Thrombosis in Cancer Patients with Peripherally Inserted Central Catheters. Iranian Red Crescent Medical Journal. 2023;25(9).

8. Li J, Hao N, Han J, Zhang M, Li X. Incidence and Predictive Model of Medical Adhesive-Related Skin Injury in Cancer Patients Managed With Central Venous Access Devices: A Retrospective Study. J Wound Ostomy Continence Nurs. 2023;50(3):209-13.

9. Hu L, Li Y, Li H, Ling W, Zheng C, Ran M, et al. Nurses' practice of peripherally inserted central catheter maintenance and its influencing factors in Guizhou province, China: a cross-sectional study. BMJ Open. 2023;13(3):e068656.

10. Thamlikitkul V, Indranoi A. Switching from heparinized saline flush to normal saline flush for maintaining peripheral venous catheter patency. Int J Qual Health Care. 2006;18(3):183-5.

11. Smith SN, Moureau N, Vaughn VM, Boldenow T, Kaatz S, Grant PJ, et al. Patterns and Predictors of Peripherally Inserted Central Catheter Occlusion: The 3P-O Study. J Vasc Interv Radiol. 2017;28(5):749-56 e2.

12. Shenep LE, Shenep MA, Cheatham W, Hoffman JM, Hale A, Williams BF, et al. Efficacy of intravascular catheter lock solutions containing preservatives in the prevention of microbial colonization. J Hosp Infect. 2011;79(4):317-22.

13. Paisley MK, Stamper M, Brown J, Brown N, Ganong LH. The use of heparin and normal saline flushes in neonatal intravenous catheters. Pediatr Nurs. 1997;23(5):521-4, 7.

14. Lyons MG, Phalen AG. A randomized controlled comparison of flushing protocols in home care patients with peripherally inserted central catheters. J Infus Nurs. 2014;37(4):270-81.

15. Lee HJ, Yang PS, Lee SB, Yi JS, Ryu SY, Kim TW, et al. The Influence of Flush Methods on Transfemoral Catheter Cerebral Angiography: Continuous Flush versus Intermittent Flush. J Vasc Interv Radiol. 2016;27(5):651-7.

16. Jonker MA, Osterby KR, Vermeulen LC, Kleppin SM, Kudsk KA. Does low-dose heparin maintain central venous access device patency?: a comparison of heparin versus saline during a period of heparin shortage. JPEN J Parenter Enteral Nutr. 2010;34(4):444-9.

17. Danek GD, Noris EM. Pediatric i.v. catheters: efficacy of saline flush. Pediatr Nurs. 1992;18(2):111-3.

18. Bowers L, Speroni KG, Jones L, Atherton M. Comparison of occlusion rates by flushing solutions for peripherally inserted central catheters with positive pressure Luer-activated devices. J Infus Nurs. 2008;31(1):22-7.

19. Myaneh ZT, Ali Alizadeh S, Shahrokhi A, Rashvand F. Comparing the Effects of Chlorhexidine 2% and Iodopovidone-alcohol on Peripheral Venous Catheter Bacterial Colonization in Preterm Neonates. Iranian Journal of Neonatology. 2019;10(3).

20. Bahl A, Pandurangadu AV, Tucker J, Bagan M. A randomized controlled trial assessing the use of ultrasound for nurse-performed IV placement in difficult access ED patients. Am J Emerg Med. 2016;34(10):1950-4.

21. Mohammed A. The effectiveness of the light source device on the insertion of cannulae into the peripheral veins in children in Khartoum state hospitals 2012. Int J Sci Res (IJSR) 2016;5(3):2020–47.

22. Bair AE, Rose JS, Vance CW, Andrada-Brown E, Kuppermann N. Ultrasound-assisted peripheral venous access in young children: a randomized controlled trial and pilot feasibility study. West J Emerg Med. 2008;9(4):219-24.

23. Steege SL, Stout-Aguilar J, Rider C. Using Ultrasound-guided PIV Insertion in the Pediatric Population to Decrease Insertion Attempts. Journal of Radiology Nursing. 2021;40(2):157-60.

24. Safaee F, Kazemian M, Borimnejad L, Rasouli M. Effects of a Care Package on the Shelf Life and Incidence of the Infection of Central Peripheral Venous Catheter in the Premature Infants Admitted to the NICU. Journal of Client-centered Nursing Care. 2020;6(4):231-8.

25. Reguindin J, Wang A, Capoccitti K, Lai S, Saby C, Sakin N, et al. Supporting clinical competency in managing peripherally inserted central catheters during the COVID-19 pandemic: An education evaluation. Vascular Access. 2022;16(2):30-40.

26. Neshat H, Aslani K, Jamshidi M, Aslanabadi S, Ghorbani F. Comparison of the Effect of Massage and EMLA Cream on Children's Physiological Indices During Venipuncture: A Factorial Clinical Trial. J Perianesth Nurs. 2020;35(6):619-24.

27. Morata L, Ogilvie C, Yon J, Johnson A. Decreasing Peripherally Inserted Central Catheter Use With Ultrasound-Guided Peripheral Intravenous Lines: A Quality Improvement Project in the Acute Care Setting. J Nurs Adm. 2017;47(6):338-44.

28. Meyer BM. Managing Peripherally Inserted Central Catheter Thrombosis Risk: A Guide for Clinical Best Practice. Journal of the Association for Vascular Access. 2011;16(3):144-7.

29. Jayalaxmi N, Devi MB, Monika N. Effectiveness of Structured Teaching Programme on Knowledge and Practice Regarding Intravenous Cannulation among the Staff Nurses. International Journal of Nursing Education. 2016;8(2):46-9.

30. Emamgholi S, Khanjari S, Haghani H. Impact of an Educational Program on Nurses' Performance in Providing Peripherally Inserted Central Catheter Care for Neonates. J Infus Nurs. 2020;43(5):275-82.

31. Oliveira AM, Danski MT, Pedrolo E. Technological innovation for peripheral venipuncture: ultrasound training. Rev Bras Enferm. 2016;69(6):1052-8.

32. Barreras J, Chang TP. Using a Near Infrared Device to Improve Successful Venous Access in Children with Special Health Care Needs. Journal of the Association for Vascular Access. 2017;22(2):75-80.

33. Hess HA. A biomedical device to improve pediatric vascular access success. Pediatr Nurs. 2010;36(5):259-63.

34. Bian Y, Huang Y, Bai J, Zheng J, Huang Y. A randomized controlled trial of ultrasound-assisted technique versus conventional puncture method for saphenous venous cannulations in children with congenital heart disease. BMC anesthesiol. 2021;21(1):131.

35. Gras S, Roy-Gash F, Bruneau B, Salvi N, Colas AE, Skhiri A, et al. Reducing the time to successful intravenous cannulation in anaesthetised children with poor vein visibility using a near-infrared device: A randomised multicentre trial. Eur J Anaesthesiol. 2021;38(8):888-94.

36. Hosokawa K, Kato H, Kishi C, Kato Y, Shime N. Transillumination by light-emitting diode facilitates peripheral venous cannulations in infants and small children. Acta Anaesthesiol Scand. 2010;54(8):957-61.

37. Ruiz-Sternberg A, Velez-Van-Meerbeke A, Ruiz-Sternberg J. Clinical acceptability and ease of use of a safety IV catheter system. Curr Med Res Opin. 2012;28(8):1381-7.

38. Fan YY, Qin HY, Li J, Xin MZ. Randomized comparison of cancer chemotherapy patient's degree of comfort of two methods of peripherally inserted central venous catheters placement. Chinese Journal of Cancer Prevention and Treatment. 2013;20(21):1679-85.

39. de Graaff JC, Cuper NJ, van Dijk AT, Timmers-Raaijmaakers BC, van der Werff DB, Kalkman CJ. Evaluating NIR vascular imaging to support intravenous cannulation in awake children difficult to cannulate; a randomized clinical trial. Paediatr Anaesth. 2014;24(11):1174-9.

40. Costa P, Dorea EP, Kimura AF, Yamamoto LY, Damiani LP. Incidence of Nonelective Removal of Single-Lumen Silicone and Dual-Lumen Polyurethane Percutaneously Inserted Central Catheters in Neonates. Journal of the Association for Vascular Access. 2014;19(1):35-41.

41. McNeely HL, Ream TL, Thrasher JM, Dziadkowiec O, Callahan TJ. Utilization of a biomedical device (VeinViewer((R)) ) to assist with peripheral intravenous catheter (PIV) insertion for pediatric nurses. J Spec Pediatr Nurs. 2018;23(2):e12208.

42. Chen IL, Ou-Yang MC, Chen FS, Chung MY, Chen CC, Liu YC, et al. The equations of the inserted length of percutaneous central venous catheters on neonates in NICU. Pediatr neonatol. 2019;60(3):305-10.

43. Song Y, Liu S, Lou T, Ma Y, Wang N, Yong Q, et al. Risk factors associated with peripherally inserted central catheter-related venous thrombosis in hospitalized patients of advanced age. J Int Med Res. 2020;48(1):300060518820744.

44. Hu Q, Su Y, Yan L. Effects of Peripherally Inserted Central Catheter (PICC) Catheterization Nursing on Bloodstream Infection in Peripheral Central Venous Catheters in Lung Cancer: A Single-Center, Retrospective Study. Comput Math Methods Med. 2022;2022:2791464.

45. Usclade A, Blanc N, Kohlmuller M, Torres A, Siret S, Tachet C, et al. Infrared augmented reality device versus standard procedure for peripheral venous catheterisation in children less than 3 years old: A quasi-experimental cluster randomised controlled trial. J Clin Nurs. 2022;31(11-12):1628-35.

46. Verma S, Bangarwa N, Ahlawat G, Kamal K. Light-emitting Diode Vein Finding Device in Facilitating Peripheral Intravenous Cannulation in Children: A Randomised Clinical Study. Journal of Clinical and Diagnostic Research. 2022;16(10):9-12.

47. Luister A, Khostwal N, Deindl P, Herrmann J, Singer D, Ebenebe CU. Recommendations for Peripherally Inserted Central Catheter Insertion Depths in Neonates. Neonatology. 2023;120(2):263-7.

48. Manner T, Kanto J, Iisalo E, Lindberg R, Viinamaki O, Scheinin M. Reduction of pain at venous cannulation in children with a eutectic mixture of lidocaine and prilocaine (EMLA cream): comparison with placebo cream and no local premedication. Acta Anaesthesiol Scand. 1987;31(8):735-9.

49. Wig J, Johl KS. Our experience with EMLA Cream (for painless venous cannulation in children). Indian J Physiol Pharmacol. 1990;34(2):130-2.

50. Arts SE, Abu-Saad HH, Champion GD, Crawford MR, Fisher RJ, Juniper KH, et al. Age-related response to lidocaine-prilocaine (EMLA) emulsion and effect of music distraction on the pain of intravenous cannulation. Pediatrics. 1994;93(5):797-801.

51. Soong WJ, Jeng MJ, Hwang B. The evaluation of percutaneous central venous catheters--a convenient technique in pediatric patients. Intensive Care Med. 1995;21(9):759-65.

52. Smith AJ, Eggers KA, Stacey MR. Topical ibuprofen for skin analgesia prior to venepuncture. Anaesthesia. 1996;51(5):495-7.

53. Lander J, Hodgins M, Nazarali S, McTavish J, Ouellette J, Friesen E. Determinants of success and failure of EMLA. Pain. 1996;64(1):89-97.

54. Garcia OC, Reichberg S, Brion LP, Schulman M. Topical anesthesia for line insertion in very low birth weight infants. J Perinatol. 1997;17(6):477-80.

55. MacRae K. Hand-held Dopplers in central catheter insertion. Prof Nurse. 1998;14(2):99-102.

56. Moore J. No more tears: a randomized controlled double-blind trial of Amethocaine gel vs. placebo in the management of procedural pain in neonates. J Adv Nurs. 2001;34(4):475-82.

57. Cordoni A, Cordoni LE. Eutectic mixture of local anesthetics reduces pain during intravenous catheter insertion in the pediatric patient. Clin J Pain. 2001;17(2):115-8.

58. Wolf AR, Stoddart PA, Murphy PJ, Sasada M. Rapid skin anaesthesia using high velocity lignocaine particles: a prospective placebo controlled trial. Arch Dis Child. 2002;86(4):309-12.

59. Ballantyne M, McNair C, Ung E, Gibbins S, Stevens B. A randomized controlled trial evaluating the efficacy of tetracaine gel for pain relief from peripherally inserted central catheters in infants. Adv Neonatal Care. 2003;3(6):297-307.

60. Coyle D, Bloomgarden D, Beres R, Patel S, Sane S, Hurst E. Power injection of contrast media via peripherally inserted central catheters for CT. J Vasc Interv Radiol. 2004;15(8):809-14.

61. Machado AF, Pedreira ML, Chaud MN. [Prospective, randomized and controlled trial on the dwell time of peripheral intravenous catheters in children, according to three dressing regimens]. Rev Lat Am Enfermagem. 2005;13(3):291-8.

62. Costantino TG, Parikh AK, Satz WA, Fojtik JP. Ultrasonography-guided peripheral intravenous access versus traditional approaches in patients with difficult intravenous access. Ann Emerg Med. 2005;46(5):456-61.

63. Singer AJ, Weeks R, Regev R. Laser-assisted anesthesia reduces the pain of venous cannulation in children and adults: a randomized controlled trial. Acad Emerg Med. 2006;13(6):623-8.

64. Lemyre B, Sherlock R, Hogan D, Gaboury I, Blanchard C, Moher D. How effective is tetracaine 4% gel, before a peripherally inserted central catheter, in reducing procedural pain in infants: a randomized double-blind placebo controlled trial [ISRCTN75884221]. BMC Med. 2006;4:11.

65. Skarbek-Borowska S, Becker BM, Lovgren K, Bates A, Minugh PA. Brief focal ultrasound with topical anesthetic decreases the pain of intravenous placement in children. Pediatr Emerg Care. 2006;22(5):339-45.

66. Windle PE, Kwan ML, Warwick H, Sibayan A, Espiritu C, Vergara J. Comparison of bacteriostatic normal saline and lidocaine used as intradermal anesthesia for the placement of intravenous lines. J Perianesth Nurs. 2006;21(4):251-8.

67. Yilmaz G, Caylan R, Aydin K, Topbas M, Koksal I. Effect of education on the rate of and the understanding of risk factors for intravascular catheter-related infections. Infect Control Hosp Epidemiol. 2007;28(6):689-94.

68. Aponte H, Acosta S, Rigamonti D, Sylvia B, Austin P, Samolitis T. The use of ultrasound for placement of intravenous catheters. Aana J. 2007;75(3):212-6.

69. Simhi E, Kachko L, Bruckheimer E, Katz J. A vein entry indicator device for facilitating peripheral intravenous cannulation in children: a prospective, randomized, controlled trial. Anesth Analg. 2008;107(5):1531-5.

70. Lolom I, Deblangy C, Capelle A, Guerinot W, Bouvet E, Barry B, et al. [Effect of a long-term quality improvement program on the risk of infection related to peripheral venous catheters]. Presse Med. 2009;38(1):34-42.

71. Stein J, George B, River G, Hebig A, McDermott D. Ultrasonographically guided peripheral intravenous cannulation in emergency department patients with difficult intravenous access: a randomized trial. Ann Emerg Med. 2009;54(1):33-40.

72. Doniger SJ, Ishimine P, Fox JC, Kanegaye JT. Randomized controlled trial of ultrasound-guided peripheral intravenous catheter placement versus traditional techniques in difficult-access pediatric patients. Pediatr Emerg Care. 2009;25(3):154-9.

73. Stokowski G, Steele D, Wilson D. The use of ultrasound to improve practice and reduce complication rates in peripherally inserted central catheter insertions: final report of investigation. J Infus Nurs. 2009;32(3):145-55.

74. Visscher M, deCastro MV, Combs L, Perkins L, Winer J, Schwegman N, et al. Effect of chlorhexidine gluconate on the skin integrity at PICC line sites. J Perinatol. 2009;29(12):802-7.

75. Oakley E, Wong AM. Ultrasound-assisted peripheral vascular access in a paediatric ED. Emerg Med Australas. 2010;22(2):166-70.

76. Aouad MT, Kanazi GE, Abdallah FW, Moukaddem FH, Turbay MJ, Obeid MY, et al. Femoral vein cannulation performed by residents: a comparison between ultrasound-guided and landmark technique in infants and children undergoing cardiac surgery. Anesth Analg. 2010;111(3):724-8.

77. Boyd S, Aggarwal I, Davey P, Logan M, Nathwani D. Peripheral intravenous catheters: the road to quality improvement and safer patient care. J Hosp Infect. 2011;77(1):37-41.

78. Elgin K, Cozzi K, Fowler MA, Perry SA, Davis MS, Conaway MR, et al. Maintaining patency with packed red blood cell infusions: comparison of IV normal saline infusion vs. normal saline syringe method. Medsurg Nurs. 2011;20(3):134-8.

79. Chapman LL, Sullivan B, Pacheco AL, Draleau CP, Becker BM. VeinViewer-assisted Intravenous catheter placement in a pediatric emergency department. Acad Emerg Med. 2011;18(9):966-71.

80. Phipps K, Modic A, O'Riordan MA, Walsh M. A randomized trial of the Vein Viewer versus standard technique for placement of peripherally inserted central catheters (PICCs) in neonates. J Perinatol. 2012;32(7):498-501.

81. Tofani BF, Rineair SA, Gosdin CH, Pilcher PM, McGee S, Varadarajan KR, et al. Quality improvement project to reduce infiltration and extravasation events in a pediatric hospital. J Pediatr Nurs. 2012;27(6):682-9.

82. Kim DK, Choi SW, Kwak YH. The effect of SonoPrep(R) on EMLA(R) cream application for pain relief prior to intravenous cannulation. Eur J Pediatr. 2012;171(6):985-8.

83. Benkhadra M, Collignon M, Fournel I, Oeuvrard C, Rollin P, Perrin M, et al. Ultrasound guidance allows faster peripheral IV cannulation in children under 3 years of age with difficult venous access: a prospective randomized study. Paediatr Anaesth. 2012;22(5):449-54.

84. de Carvalho Onofre PS, da Luz Goncalves Pedreira M, Peterlini MA. Placement of peripherally inserted central catheters in children guided by ultrasound: a prospective randomized, and controlled trial. Pediatr Crit Care Med. 2012;13(5):e282-7.

85. Kaddoum RN, Anghelescu DL, Parish ME, Wright BB, Trujillo L, Wu J, et al. A randomized controlled trial comparing the AccuVein AV300 device to standard insertion technique for intravenous cannulation of anesthetized children. Paediatr Anaesth. 2012;22(9):884-9.

86. Kampf G, Reise G, James C, Gittelbauer K, Gosch J, Alpers B. Improving patient safety during insertion of peripheral venous catheters: an observational intervention study. GMS Hyg Infect Control. 2013;8(2):Doc18.

87. Abdeyazdan Z, Sheikhan-Sudani E, Sadeghnia A, Talakoub S. Effect of using static ultrasound technique on peripherally inserted central catheters' insertion success rate in neonates in a neonatal intensive care unit. Iran J Nurs Midwifery Res. 2014;19(6):643-6.

88. Evans RS, Sharp JH, Linford LH, Lloyd JF, Woller SC, Stevens SM, et al. Reduction of peripherally inserted central catheter-associated DVT. Chest. 2013;143(3):627-33.

89. Mestre G, Berbel C, Tortajada P, Alarcia M, Coca R, Fernandez MM, et al. Successful multifaceted intervention aimed to reduce short peripheral venous catheter-related adverse events: a quasiexperimental cohort study. Am J Infect Control. 2013;41(6):520-6.

90. Weiner SG, Sarff AR, Esener DE, Shroff SD, Budhram GR, Switkowski KM, et al. Single-operator ultrasound-guided intravenous line placement by emergency nurses reduces the need for physician intervention in patients with difficult-to-establish intravenous access. J Emerg Med. 2013;44(3):653-60.

91. Waterhouse MR, Liu DR, Wang VJ. Cryotherapeutic topical analgesics for pediatric intravenous catheter placement: ice versus vapocoolant spray. Pediatr Emerg Care. 2013;29(1):8-12.

92. Srinivasan HB, Tjin ATA, Galang R, Hecht A, Srinivasan G. Migration patterns of peripherally inserted central venous catheters at 24 hours postinsertion in neonates. Am J Perinatol. 2013;30(10):871-4.

93. Szmuk P, Steiner J, Pop RB, Farrow-Gillespie A, Mascha EJ, Sessler DI. The VeinViewer vascular imaging system worsens first-attempt cannulation rate for experienced nurses in infants and children with anticipated difficult intravenous access. Anesth Analg. 2013;116(5):1087-92.

94. Katheria AC, Fleming SE, Kim JH. A randomized controlled trial of ultrasound-guided peripherally inserted central catheters compared with standard radiograph in neonates. J Perinatol. 2013;33(10):791-4.

95. Avelar AF, Peterlini MA, da Pedreira ML. [Assertiveness and peripheral intravenous catheters dwell time with ultrasonography-guided insertion in children and adolescents]. Rev Esc Enferm USP. 2013;47(3):539-46.

96. Singh A, Bajpai M, Panda SS, Jana M. Complications of peripherally inserted central venous catheters in neonates: Lesson learned over 2 years in a tertiary care centre in India. Afr J Paediatr Surg. 2014;11(3):242-7.

97. Aulagnier J, Hoc C, Mathieu E, Dreyfus JF, Fischler M, Le Guen M. Efficacy of AccuVein to facilitate peripheral intravenous placement in adults presenting to an emergency department: a randomized clinical trial. Acad Emerg Med. 2014;21(8):858-63.

98. Ismailoglu EG, Zaybak A, Akarca FK, Kiyan S. The effect of the use of ultrasound in the success of peripheral venous catheterisation. Int Emerg Nurs. 2015;23(2):89-93.

99. Takeshita J, Nakayama Y, Nakajima Y, Sessler DI, Ogawa S, Sawa T, et al. Optimal site for ultrasound-guided venous catheterisation in paediatric patients: an observational study to investigate predictors for catheterisation success and a randomised controlled study to determine the most successful site. Crit Care. 2015;19(1):15.

100. Smitherman AB, Alexander T, Connelly M, Snavely AC, Weston BW, Liles EA, et al. The incidence of catheter-associated venous thrombosis in noncritically ill children. Hosp Pediatr. 2015;5(2):59-66.

101. Curtis SJ, Craig WR, Logue E, Vandermeer B, Hanson A, Klassen T. Ultrasound or near-infrared vascular imaging to guide peripheral intravenous catheterization in children: a pragmatic randomized controlled trial. Cmaj. 2015;187(8):563-70.

102. Curtis K, Ockerby C, Bennett P, Heywood E, Marshall L. Peripherally inserted central catheter cushioning:  a pilot study comparing gauze with silicone foam. Clin J Oncol Nurs. 2015;19(3):253-6.

103. Schmitz ML, Zempsky WT, Meyer JM. Safety and Efficacy of a Needle-free Powder Lidocaine Delivery System in Pediatric Patients Undergoing Venipuncture or Peripheral Venous Cannulation: Randomized Double-blind COMFORT-004 Trial. Clin Ther. 2015;37(8):1761-72.

104. Ramer L, Hunt P, Ortega E, Knowlton J, Briggs R, Hirokawa S. Effect of Intravenous (IV) Assistive Device (VeinViewer) on IV Access Attempts, Procedural Time, and Patient and Nurse Satisfaction. J Pediatr Oncol Nurs. 2016;33(4):273-81.

105. Jeon EY, Cho YK, Yoon DY, Hwang JH. Which arm and vein are more appropriate for single-step, non-fluoroscopic, peripherally inserted central catheter insertion? J. 2016;17(3):249-55.

106. Rupp JD, Ferre RM, Boyd JS, Dearing E, McNaughton CD, Liu D, et al. Extravasation Risk Using Ultrasound-guided Peripheral Intravenous Catheters for Computed Tomography Contrast Administration. Acad Emerg Med. 2016;23(8):918-21.

107. Partovi-Deilami K, Nielsen JK, Moller AM, Nesheim SS, Jorgensen VL. Effect of Ultrasound-Guided Placement of Difficult-to-Place Peripheral Venous Catheters: A Prospective Study of a Training Program for Nurse Anesthetists. Aana J. 2016;84(2):86-92.

108. Keleekai NL, Schuster CA, Murray CL, King MA, Stahl BR, Labrozzi LJ, et al. Improving Nurses' Peripheral Intravenous Catheter Insertion Knowledge, Confidence, and Skills Using a Simulation-Based Blended Learning Program: A Randomized Trial. Simul Healthc. 2016;11(6):376-84.

109. McDiarmid S, Scrivens N, Carrier M, Sabri E, Toye B, Huebsch L, et al. Outcomes in a nurse-led peripherally inserted central catheter program: a retrospective cohort study. CMAJ Open. 2017;5(3):E535-E9.

110. Watterson K, Hauck MJ, Auker A, Burns R, Greider J, Marlin M, et al. S.T.I.C.K.: A Quality Improvement Pediatric IV Infiltration Prevention Bundle. J Pediatr Nurs. 2018;41:38-41.

111. Jeong IS, Jeon GR, Lee MS, Shin BJ, Kim YJ, Park SM, et al. Intravenous Infiltration Risk by Catheter Dwell Time Among Hospitalized Children. J Pediatr Nurs. 2017;32:47-51.

112. Zhang X, Lu Z, Hu Y, Xue M, Dai H. Evidence-Based Implementation of Peripherally Inserted Central Catheters (PICCs) Insertion at a Vascular Access Care Outpatient Clinic. Worldviews Evid Based Nurs. 2017;14(2):163-7.

113. Gopalasingam N, Obad DS, Kristensen BS, Lundgaard P, Veien M, Gjedsted J, et al. Ultrasound-guidance outperforms the palpation technique for peripheral venous catheterisation in anaesthetised toddlers: a randomised study. Acta Anaesthesiol Scand. 2017;61(6):601-8.

114. Feinsmith S, Huebinger R, Pitts M, Baran E, Haas S. Outcomes of a Simplified Ultrasound-Guided Intravenous Training Course for Emergency Nurses. J Emerg Nurs. 2018;44(2):169-75 e2.

115. Xu B, Zhang J, Tang S, Hou J, Ma M. Comparison of two types of catheters through femoral vein catheterization in patients with lung cancer undergoing chemotherapy: A retrospective study. J. 2018;19(6):651-7.

116. Whalen M, Maliszewski B, Sheinfeld R, Gardner H, Baptiste D. Outcomes of an Innovative Evidence-Based Practice Project: Building a Difficult-Access Team in the Emergency Department. J Emerg Nurs. 2018;44(5):478-82.

117. Saliba P, Hornero A, Cuervo G, Grau I, Jimenez E, Berbel D, et al. Interventions to decrease short-term peripheral venous catheter-related bloodstream infections: impact on incidence and mortality. J Hosp Infect. 2018;100(3):e178-e86.

118. Otani T, Morikawa Y, Hayakawa I, Atsumi Y, Tomari K, Tomobe Y, et al. Ultrasound-guided peripheral intravenous access placement for children in the emergency department. Eur J Pediatr. 2018;177(10):1443-9.

119. Carr PJ, Rippey JCR, Cooke ML, Higgins NS, Trevenen M, Foale A, et al. From insertion to removal: A multicenter survival analysis of an admitted cohort with peripheral intravenous catheters inserted in the emergency department. Infect Control Hosp Epidemiol. 2018;39(10):1216-21.

120. Platt V, Osenkarski S. Improving Vascular Access Outcomes and Enhancing Practice. J Infus Nurs. 2018;41(6):375-82.

121. Demir D, Inal S. Does the Use of a Vein Visualization Device for Peripheral Venous Catheter Placement Increase Success Rate in Pediatric Patients? Pediatr Emerg Care. 2019;35(7):474-9.

122. Egerton-Warburton D, McAllan F, Ramanan R, Lim ZJ, Nagle D, Dendle C, et al. Human factor-designed multimodal intervention reduces the rate of unused peripheral intravenous cannula insertion. Emerg Med Australas. 2019;31(3):372-7.

123. Keller EJ, Aragona E, Molina H, Lee J, Salem R, Resnick SA, et al. Cost-Effectiveness of a Guided Peripherally Inserted Central Catheter Placement System: A Single-Center Cohort Study. J Vasc Interv Radiol. 2019;30(5):709-14.

124. Carr PJ, Rippey JCR, Cooke ML, Trevenen ML, Higgins NS, Foale AS, et al. Factors associated with peripheral intravenous cannulation first-time insertion success in the emergency department. A multicentre prospective cohort analysis of patient, clinician and product characteristics. BMJ Open. 2019;9(4):e022278.

125. Vinograd AM, Chen AE, Woodford AL, Fesnak S, Gaines S, Elci OU, et al. Ultrasonographic Guidance to Improve First-Attempt Success in Children With Predicted Difficult Intravenous Access in the Emergency Department: A Randomized Controlled Trial. Ann Emerg Med. 2019;74(1):19-27.

126. Hartman JH, Bena JF, Morrison SL, Albert NM. Effect of Adding a Pediatric Vascular Access Team Component to a Pediatric Peripheral Vascular Access Algorithm. J Pediatr Health Care. 2020;34(1):4-9.

127. Galen B, Baron S, Young S, Hall A, Berger-Spivack L, Southern W. Reducing peripherally inserted central catheters and midline catheters by training nurses in ultrasound-guided peripheral intravenous catheter placement. BMJ Qual Saf. 2020;29(3):245-9.

128. Skulec R, Callerova J, Vojtisek P, Cerny V. Two different techniques of ultrasound-guided peripheral venous catheter placement versus the traditional approach in the pre-hospital emergency setting: a randomized study. Intern Emerg Med. 2020;15(2):303-10.

129. Fujioka G, Newcomb P, Hunchusky C, Myers H, Behan D. Pain Perception of a Structured Vascular Access Team Approach to Short Peripheral Catheter (SPC) Placement Compared to SPC Placement by Bedside Nurses. J Infus Nurs. 2020;43(1):33-8.

130. Archer-Jones A, Sweeny A, Schults JA, Rickard CM, Johnson L, Gunter A, et al. Evaluating an ultrasound-guided peripheral intravenous cannulation training program for emergency clinicians: An Australian perspective. Australas Emerg Care. 2020;23(3):151-6.

131. Kanno C, Murayama R, Abe-Doi M, Takahashi T, Shintani Y, Nogami J, et al. Development of an algorithm using ultrasonography-assisted peripheral intravenous catheter placement for reducing catheter failure. Drug Discov Ther. 2020;14(1):27-34.

132. Barth D, Nemec RM, Cho DD, Slomer A, Cojocari E, Kim K, et al. The practical integration of a hybrid model of ultrasound-guided peripheral venous access in a large apheresis center. J Clin Apher. 2020;35(4):328-34.

133. Inal S, Demir D. Impact of Peripheral Venous Catheter Placement With Vein Visualization Device Support on Success Rate and Pain Levels in Pediatric Patients Aged 0 to 3 Years. Pediatr Emerg Care. 2021;37(3):138-44.

134. Cottrell JT, Chang T, Baird J, Barreras J, Elkhunovich MA. Ultrasound-guided placement of peripherally inserted intravenous catheters increase catheter dwell time in children. J. 2021;22(2):189-93.

135. Mulemba T, Bank R, Sabantini M, Chopi V, Chirwa G, Mumba S, et al. Improving Peripheral Intravenous Catheter Care for Children with Cancer Receiving Chemotherapy in Malawi. J Pediatr Nurs. 2021;56:13-7.

136. Resnick O, Abu Ahmad W, Bancovsky D, Rogachev S, Ashash A, Ohana Sarna Cahan L, et al. Predicting factors for complications in peripheral intravenous catheters in the pediatric population. Acta Paediatr. 2021;110(5):1639-44.

137. Boyar V, Galiczewski C. Reducing Peripheral Intravenous Catheter Extravasation in Neonates: A Quality Improvement Project. J Wound Ostomy Continence Nurs. 2021;48(1):31-8.

138. Hackett A, Wells C, Zhang Z, Kero J, Soriano J, Rivera J, et al. Development of a Peripheral Intravenous Access Training Program for Nurses in the Pediatric Intensive Care Units. J Pediatr Nurs. 2021;61:394-403.

139. Li F, Shen H, Wang M, Wang Y. Peripheral insertion of reverse-tapered and non-tapered central catheters (PICC) in patients receiving tumor chemotherapy. J Cancer Res Ther. 2021;17(7):1651-5.

140. Gavelli V, Wackernagel D. Peripherally inserted central catheters in extremely preterm infants: Placement success rates and complications. Acta Paediatr. 2022;111(3):554-6.

141. Yalcinli S, Karbek Akarca F, Can O, Uz I, Konakci G. Comparison of Standard Technique, Ultrasonography, and Near-Infrared Light in Difficult Peripheral Vascular Access: A Randomized Controlled Trial. Prehosp Disaster Med. 2022;37(1):65-70.

142. Zhang Z, Wang X, Zhang L, Lou X, Su X, Wang X, et al. Infrared Vein Imaging for Insertion of Peripheral Intravenous Catheter for Patients Requiring Isolation for Severe Acute Respiratory Syndrome Coronavirus 2 Infection: A Nonrandomized Clinical Trial. J Emerg Nurs. 2022;48(2):159-66.

143. Bhargava V, Su E, Haileselassie B, Davis D, Steffen KM. Ultrasound education improves safety for peripheral intravenous catheter insertion in critically ill children. Pediatr Res. 2022;91(5):1057-63.

144. Bhargava M, Broccard S, Bai Y, Wu B, Dincer EH, Broccard A. Risk factors for peripherally inserted central catheter line-related deep venous thrombosis in critically ill intensive care unit patients. SAGE Open Med. 2020;8:2050312120929238.

145. D'Arrigo S, Sandroni C, Cacciola S, Dell'Anna AM, Pittiruti M, Annetta MG, et al. Are single-lumen 5Fr and triple-lumen 6Fr PICCs suitable for hemodynamic assessment by trans-pulmonary thermodilution? A pilot study. Ann Intensive Care. 2020;10(1):165.

146. Qian H, Liu J, Xu C, Zhu W, Chen L. Predisposing factors and effect of bundle nursing in PICC-related upper extremity deep venous thrombosis in patients with non-Hodgkin's lymphoma undergoing chemotherapy. Am J Transl Res. 2021;13(8):9679-86.

147. Bayoumi MAA, Elmalik EE, Ali H, D'Souza S, Furigay J, Romo A, et al. Neonatal Simulation Program: A 5 Years Educational Journey From Qatar. Front. 2022;10:843147.

148. Shivani S, Joseph HB. Effectiveness of reinforcement program on adherence toward short peripheral catheter (SPC) care guidelines among registered nurses working in pediatric wards of a tertiary care hospital. J Educ Health Promot. 2022;11:359.

149. Feinsmith SE, Amick AE, Feinglass JM, Sell J, Davis EM, Spencer TR, et al. Performance of peripheral catheters inserted with ultrasound guidance versus landmark technique after a simulation-based mastery learning intervention. J. 2023;24(4):630-8.

150. Mousavi H, Ghanbari A, Karkhah S, Alizadeh J, Kazemnejad Leyli E, Jafaraghaee F. The effect of clinical guideline education on the knowledge and practice of nurses for peripheral intravenous catheter placement based on short message service: A quasi-experimental study. J. 2024;25(1):132-9.

## X7_Full text not retrievable (n=41)

1. Wang Y, Miao M, Xu M, Wan G. Event characteristics and risk factors of unplanned removal of peripherally inserted central catheters in patients with chest tumor. Chinese Journal of Clinical Nutrition. 2023;31(6):354-61.

2. Li L, Liu P, Yang X, Li X, Cui H, Song J, et al. Whole-course standardized peripherally inserted central catheter implantation improves the safety of parenteral nutrition infusion: a case-control study. Chinese Journal of Clinical Nutrition. 2023;31(4):204-7.

3. Lacasaña Bellmunt P, Garcia Ortega MJ, Garcia Ruiz C, Palomino Gutiérrez B, Toro Padilla R, Vila Sánchez A, et al. Permeabilisation of peripheral venous catheters of intermittent use: with and without heparin. Metas de Enfermería. 2006;9(7):10-6.

4. Julio César de la Torre M, María Montealegre S. Heparinization versus salinization in short peripheral catheters for blood draws in clinical trials. Metas de Enfermería. 2012;15(7):15-8.

5. Golberg M, Sankaran R, Givelichian L, Sankaran K. Maintaining patency of peripheral intermittent infusion devices with heparinized saline and saline: a randomized double blind controlled trial in neonatal intensive care and a review of literature. Neonat Intensive Care. 1999;12(1):18-22.

6. Xie LL. Effects of low-concentration heparin sodium on preventing thrombophlebitis in patients with peripherally inserted central catheter. Chinese Journal of Clinical Nutrition. 2010;18(1):56-9.

7. Uitterhoeve R, de Rond M, Polman L, Rijsemus T. [Process and product of research utilization--flushing of intravenous catheters with NaCl 0.9 percent instead of heparin 150 E/ml]. Oncologica. 1997;14(4):5-9.

8. Liu J, Zhang J, Tan L. Comparison on the application of modified midline catheters and PICC applied in patients with gastrointestinal tumors in perioperative period. Nursing Practice and Research. 2018;15 (7):143-5.

9. Nishizawa T, Matsumoto T, Todaka T, Sasano M, Kitagawa H, Shimabuku A. Nurse-Performed Ultrasound-Guided Technique for Difficult Peripheral Intravenous Access in Critically Ill Patients: A Randomized Controlled Trial. The Journal of the Association for Vascular Access. 2020;25:34-9.

10. 胡惠芳, 沈宇宏, 蒋勤慧, 王洁, 周艺. AIDET沟通模式在PICC专科护理门诊中的应用. Nursing of Integrated Traditional Chinese & Western Medicine. 2018;4(10):7-10.

11. 肖月君, 谢伟琴. 预消毒法预防早产儿PICC 所致静脉炎的效果. Nursing of Integrated Traditional Chinese & Western Medicine. 2018;4(4):112-4.

12. 杨丽娟, 刘丽华, 伏蕤, 李文熙, 何爽, 江丹灵. PICC 置入病人并发机械性静脉炎列线图 预测模型的构建. Chinese Nursing Research. 2021;35(10):1746-50.

13. 孙韫, 王楠楠. 集束化管理预防新生儿导管相关性血流感染的效果观察. Nursing of Integrated Traditional Chinese & Western Medicine. 2018;4(5):141-3.

14. 吴娇, 章红, 薛凯凯, 刘玉平. 集束化护理在胃恶性肿瘤患者术后 PICC 置管维护中的应用. Nursing of Integrated Traditional Chinese & Western Medicine. 2018;4(10):142-4.

15. 关晨阳, 廖海涛, 髙文, 韦义萍. 肿瘤病人 PICC 导管相关性血栓的危险因素分析. Chinese Nursing Research. 2017;31(10):1211-5.

16. Yin L, Wang Q. Comparison and analysis of application effect of VPA and PICC in chemotherapy of young patients with cervical cancer. Chinese Nursing Research. 2015;29(12C):4567-9.

17. Ralph Webber JL, Maningo-Salinas MJ. “Sticking It to Them”—Reducing Migration of Peripherally Inserted Central Catheters. Journal of the Association for Vascular Access. 2020;25(1):10-5.

18. Rodrigo Pedrosa O, Muñoz Blanco MJ, García Morón S, García García J. Complications of peripheral venous cannula insertion depending on localisation in children. Metas de Enfermería. 2003;6(52):24-30.

19. Raynak A, Wood B. The Clinical Nurse Specialist Role and its Relevance to Vascular Access: A Canadian Perspective. Journal of the Association for Vascular Access. 2021;26(3):25-30.

20. Ong MEH, Chan YH, Yap S, Ang PHY. 'Intravenous access by paramedics in out-of-hospital cardiac arrest'. Singapore Nursing Journal. 2003;30(2):38-41.

21. Molina Chueca R, Cueto Quintana PF, Valls Artajona S, Gallego Aguirre L, Garcia Trujillo S, Rabadà Fernandez G. Ventajas de la utilización de PICCs y Midline insertados en UCI. Agora de Enfermeria. 2018;22(2):55-7.

22. Lawson TT. Influence of insertion site on short peripheral catheter performance and frequency of infiltration in neonatal patients. Neonat Intensive Care. 1996;9(3):53-7.

23. García López C, Acosta Comas A, Serra Barril MA, Fernández Ortega P. Complicaciones derivadas de la inserción periférica de catéter central con punción ciega o ecoguiada. Metas de Enfermería. 2018;21(5):64-9.

24. Ault MJ, Ng PK, Artal R. The changing use of peripherally inserted central catheters: a review of 5000 insertions at a single hospital. Journal of Clinical Outcomes Management. 2001;8(9):17-23.

25. Ashktorab T, Solimanian T, Borzabadi Z, Majd HA, Samini M. Effects of skin disinfection by alcohol and chlorhexidine on catheter-related phlebitis: a comparative study. Advances in Nursing & Midwifery. 2006;16(53):42-.

26. Arredondo N, Humston M, McCraven SS, Shields J, Palacios L, Pavelka J, et al. A WIN for Safety and Efficacy: Evaluation of Developmental WINguide and Hybrid Guide for Wire-in-Needle Peripherally Inserted Central Line Catheter (PICC) Cannulation. Journal of the Association for Vascular Access. 2021;26(1):24-31.

27. The well-dressed peripheral IV site. Emergency Medicine (00136654). 1988;20(7):63-7.

28. Goggin M, Joyce WP, Prendergast C, Gibney E. The use of fine bore silicone catheters for peripheral intravenous nutrition (IVN). A randomised study. Ir J Med Sci. 1992;161(3):83.

29. Wei-Na W, Chia-Chi K. The Comparative Efficacy of 96- and 72-Hour Replacement Cycles for Peripheral Intravenous Catheters: a Randomized Controlled Trial. Journal of nursing & healthcare research. 2014;10(3):190‐8.

30. Gabriel Botella F, Labios Gomez M, Herrera Medina I, Balaguer Martinez JV, Amer Llueca G, Ochovo Marquez S, et al. Infections associated with peripheral intravenous catheters. Comparative study of two techniques of insertion. Revista Espanola de Microbiologia Clinica. 1992;7(7):326-32.

31. Chen HC, Tzeng CM, Liu WS, Huang YF, Chen YY. Topical Xylocaine spray for reducing the pain of venipuncture in neonates. Clinical Neonatology. 2006;13(2):38-41.

32. Xia C, Lu A, Sun J. Role of modified Seldinger technique combined with vascular ultrasonography in the placement of peripherally inserted central catheters in patients with breast cancer undergoing postoperative chemotherapy. Chinese Journal of Clinical Nutrition. 2014;22(3):187-90.

33. Seckold T, Walker S, Dwyer T, Signal T. Peripherally Inserted Central Catheter Postinsertion Complications: A Retrospective Study. Journal of the Association for Vascular Access. 2019;24(1):10-20.

34. Sharifi R, Montaseri S, Edraki M, Razavi SM. The Comparison of Bacterial Colonization in the Centralized Venous Catheters by Peripheral Vessel in the Upper Limbs with the Lower Limbers of the Hospitalized Premature Newborns in the Neonatal Intensive Care Unit. International Journal of Pharmaceutical Research. 2020;12(01):27-33.

35. Anonymous. To flush or not to flush with heparin. Rn. 1993;56(4):22.

36. Garcia Lavandera MV, Suarez Perez S, Coalla Gonzalez C, Bernardo Rodriguez Y. [Pharmacy economic repercussions and other expenses in changing vascular equipment with no change in the rate of infection]. Enferm Intensiva. 1997;8(3):111-20.

37. Larson J, Hanson J. Developing a peripherally inserted central catheter service with registered nurses. Can Oncol Nurs J. 1999;9(3):145-6.

38. Del Prato F, Di Matteo A, Messina F, Napolitano M. [PICC: central venous access by the peripheral route. Medical-nursing aspects]. Minerva Pediatr. 2010;62(3 Suppl 1):161-3.

39. Campbell-Jones V. A comparison of lidocaine versus normal saline for local anesthesia before intravenous cannula insertion. J Natl Black Nurses Assoc. 2010;21(2):27-33.

40. Jackowska T, Pawlik K. [Prevention of nosocomial infections in the pediatric ward - own experiences]. Dev Period Med. 2015;19(2):225-34.

41. Gunasundram S, Tan M, Lim KZH, Loh VMP. Reducing the incidence of phlebitis in medical adult inpatients with peripheral venous catheter care bundle: a best practice implementation project. JBI Evid Implement. 2021;19(1):68-83.

# Supplement E- Outcome Tables

## Table E1: Chlorhexidine-containing antiseptic for skin preparation vs non-chlorhexidine containing antiseptics

| **Authors, Year**  **Registration Number/Trial Name**  **Country**  **Funding**  **RoB** | **Study Design**  **Study Period or Duration**  **Study Arms** | **Number of Participants (Number of Catheters)**  **Population**  **Age****  **Female (%)** | **BSI** | **BSI-related Mortality** | **Sepsis** | **Local Infection** | **All-cause Mortality** | **Phlebitis/**  **Thrombophlebitis** |
| --- | --- | --- | --- | --- | --- | --- | --- | --- |
| Garland et al. 2009 [4]  NR/NR  U.S.  Industry: Enturia (formerly Medi-Flex)  Some concerns | Open-label RCT  24 months (2005 to 2007)  G1: chlorhexidine  G2: povidone-iodine | 48 (NR)  Neonates  Weeks  Overall: NR  G1: 32.4 (2.9)  G2: 33.0 (4.1)  Overall: 33%*  G1: 33%*  G2: 33%* | CABSI:  G1: 1/24 (4%)  G2: 1/24 (4%)  p= 0.99  Incidence of BSI:  G1: 2.8 per 1000 catheter days  G2: 3.0 per 1000 catheter days  p= 0.96 | NR | NR | NR | NR | NR |
| Kinoshita et al. 2019 [5]  NR/NR  Japan  NR  Some concerns | Prospective controlled cohort study  30 months (October 2014 to March 2017)  G1: chlorhexidin  G2: non-chlorhexidine | 2383 (NR)  Neonates  Weeks gestational age  Overall: 28.5 (3.3)  G1: 28.2 (3.2)  G2: 28.6 (3.3)  Overall: 47%  G1: 45%  G2: 48% | CLABSI:  More than 1% CHG/ethanol vs 10% povidone iodine: aHR (95% CI) 1.96 (0.77 to 4.94)  Less than 1% CHG/ethanol vs 10% povidone iodine: aHR (95% CI) 4.95 (1.33 to 18.40) | NR | NR | NR | NR | NR |

Abbreviations: aHR=adjusted hazard ratio; aRR=adjusted relative risk; BSI=bloodstream infection; CABSI=catheter-associated bloodstream infection; CHD=chlorhexidine; CI=confidence interval; CLABSI=central line-associated bloodstream infection; CRBSI= catheter-related bloodstream infection; G=group; KQ=key question; NR=not reported; p=p-value; PICC=peripherally inserted central catheter; PIVC=peripheral intravenous catheter; RCT=randomised controlled trial; RoB=risk of bias; SD=standard deviation; vs=versus

*self-calculated **Age in mean (SD) unless otherwise specified

## Table E2: Training vs no specified formal training

| **Authors, Year**  **Registration Number/Trial Name**  **Country**  **Funding**  **RoB** | **Study Design**  **Study Period or Duration**  **Study Arms** | **Number of Participants (Number of Catheters)**  **Population**  **Age****  **Female (%)** | **BSI** | **BSI-related Mortality** | **Sepsis** | **Local Infection** | **All-cause Mortality** | **Phlebitis/**  **Thrombophlebitis** | **Overall Adverse Events** |
| --- | --- | --- | --- | --- | --- | --- | --- | --- | --- |
| Balachander et al. 2021 [6]  NR/NR  India  None  High | Before-after study  14 months (June 2017 to July 2018)  G1: training  G2: no training | 1631 (NR)  Neonates  Mean gestation age:  G1: 36.6 ± 2.8 (24–41)  G2: 36.3 ± 3.13 (23–42)  NR | BSI per 1000 patient days:  G1: 1.65 ± 2.16  G2: 5.5 ± 4.13  p= 0.054  PLABSI /1000 peripheral line days:  G1: 2.37 ± 3.33  G2: 10.8 ± 8.4  p=0.03  CLABSI/1000 central line days:  G1: 18.34 ± 27.31  G2: 9.11 ± 8.9  p= 0.412 | NR | NR | NR | NR | NR | NR |
| Bozaan et al. 2019 [7]  NR/NR  U.S.  NR  High | Before-after study  NR  G1: training  G2: no training | 226 (NR)  Adults  Age mean (SD)  G1: 60.3 (1.8)  G2: 60.9 (1.5)  Overall: 48%*  G1: 41%  G2: 54% | CLABSI:  G1: 2/93 (2%)  G2: 7/133 (5%)  p= 0.239 | NR | NR | NR | NR | NR | G1: 14/93 (15%)  G2: 19/133 (14%)  p= 0.872 |
| Kun et al. 2017 [8]  NR/NR  China  NR  High | Before-after study  28 days (NR)  G1: training  G2: no training | 160 (NR)  Adults  Overall: NR  G1: 49 (4.55)  G2: 48 (4.45)  Overall: 29%*  G1: 30%*  G2: 28%* | NR | NR | NR | NR | NR | NR | G1: 12/80 (15%)  G2: 29/80 (36%) |
| Purran et al. 2016 [9]  NR/NR  UK  NR  High | Before-after study  43 months (April 2011 to June 2012 and July 2012 to December 2014)  G1: training  G2: no training | 355 (NR)  NR  NR  NR | NR | NR | NR | NR | NR | NR | G1: 11/228 (5%*)  G2: 14/67 (21%*) |
| Sakai et al., 2023 [10]  NR/NR  Japan  Academic: Fujita Health University  Some concerns | Retrospective cohort study  72 months (April 2014 to March 31  2020)  G1: training  G2: no training | 1649 (2230)  Adults  Overall: 66.2  G1: 66.7  G2: 65.2  Overall: 40%  G1: 40%  G2: 40% | CLABSI- per catheters:  G1: 86/1452 (5.9%*)  G2: 50/693 (7.2%*)  aHR (95% CI): 0.96 (0.53 to 1.75)  Sensitivity analysis (matched cases):  G1: 15/357 (4.2%)  G2: 28/357 (7.8%) aHR (95% CI): 0.82 (0.44 to 1.54) | NR | NR | NR | NR | NR | Per catheters:  G1: 22/1505 (1.5%)  G2: 37/725 (5.1%) |
| Walters et al. 2019 [11]  NR/NR  U.S.  NR  High | Before-after study  41 months (August 2014 to 2017)  PICC  G1: training  G2: no training | 1276* (NR)  Adults  NR  NR | CLABSI:  G1: 0/1082* (0%)  G2: 1/194 (0%) | NR | NR | NR | NR | NR | NR |
| Zhang et al. 2014 [12]  NR/NR  China  NR  Some concerns | Retrospective cohort study  12 months (August 2010 to August 2011)  PICC  G1: training  G2: no training | 610 (NR)  Adults  Overall: NR  G1: 54.83 (12.94)  G2: 54.78 (13.34)  Overall: 36.4%*  G1: 39%*  G2: 33%* | Catheter-related infections (bacteremia or fungemia):  G1: 0/310 (0%)  G2: 8/300 (3%)  p< 0.01 | NR | NR | NR | NR | Phlebitis:  G1: 5/310 (2%)  G2: 20/300 (7%)  p< 0.05 | NR |

Abbreviations: BSI=bloodstream infection; G=group; (a)HR= (adjusted) hazard ratio; KQ=key question; IVC=intravenous catheter; NR=not reported; p=p-value; PICC=peripherally inserted central catheter; PIVC=peripheral intravenous catheter; RCT=randomised controlled trial; RoB=risk of bias; SD=standard deviation; vs=versus

*self-calculated

**Age in mean (SD) unless otherwise specified

## Table E3: Gloves vs no gloves

| **Authors, Year**  **Registration Number/Trial Name**  **Country**  **Funding**  **RoB** | **Study Design**  **Study Period or Duration**  **Study Arms** | **Number of Participants (Number of Catheters)**  **Population**  **Age****  **Female (%)** | **BSI** | **BSI-related Mortality** | **Sepsis** | **Local Infection** | **All-cause Mortality** | **Phlebitis/**  **Thrombophlebitis** | **Overall Adverse Events** |
| --- | --- | --- | --- | --- | --- | --- | --- | --- | --- |
| Kaufman et al. 2014 [13]  NCT01729000/NR  U.S.  Academic, Foundation/Non-profit professional organization: University of Virginia Institute of Quality and Patient Safety, Cardinal Health Foundation, and University of Virginia Children’s Hospital Grant’s Program  Low | Open-label RCT  30 months (December 2008 to June 2011)  G1: gloves  G2: hand hygiene alone | 124 (NR)  Neonates  Days  Overall: NR  G1: 3.3 (1.7)  G2: 3.6 (1.9)  Weeks gestational age:  Overall: NR  G1: 25.7 (1.8)  G2: 25.9 (1.7)  Overall: 52%*  G1: 52%  G2: 52% | CLABSI:  G1: 4/60 (7%*)  G2: 4/60 (7%*)  Any BSI:  G1: 19/60 (32%)  G2: 27/60 (45%) | NR | NR | NR | G1: 6/60 (10%)  G2: 2/60 (3%) | NR | NR |

Abbreviations: BSI=bloodstream infection; CDC=Centers for Disease Control and Prevention; CI=confidence interval; CLABSI=central line-associated bloodstream infection; G=group; IVC=intravenous catheter; KQ=key question; NR=not reported; p=p-value; PICC=peripherally inserted central catheter; PIVC=peripheral intravenous catheter; RCT=randomised controlled trial; RoB=risk of bias; RR=relative risk; SD=standard deviation; vs=versus

*self-calculated

**Age in mean (SD) unless otherwise specified

## Table E4: Ultrasound-guided insertion vs non-ultrasound-guided guided

| **Authors, Year**  **Registration Number/Trial Name**  **Country**  **Funding**  **RoB** | **Study Design**  **Study Period or Duration**  **Study Arms** | **Number of Participants (Number of Catheters)**  **Population**  **Age****  **Female (%)** | **BSI** | **BSI-related Mortality** | **Sepsis** | **Local Infection** | **All-cause Mortality** | **Phlebitis/**  **Thrombophlebitis** |
| --- | --- | --- | --- | --- | --- | --- | --- | --- |
| Barber et al. 2002 [14]  NR/NR  UK  NR  Some concerns | Prospective controlled cohort study  36 months  G1: US  G2: non-US | 144 (NR)  NR  NR  NR | Systemic infection- per catheter:  G1: 3/37 (8%*)  G2: 4/107 (4%*) | NR | NR | NR | Per catheter:  G1: 1/37 (3%*)  G2: 7/107 (7%*) | Thormbophlebitis- per catheter:  G1: 0/37 (0%*)  G2: 0/107 (0%*) |
| Chen et al. 2017 [15]  NR/NR  Taiwan  NR  High | Retrospective cohort study  120 months (January 2004 to December 2014)  G1:US  G2: non-US | 43 (NR)  Adults  Years  G1: 46.3 (14.1)  G2: 48.7 (16.9)  Overall: 52%*  G1: 47%  G2: 57% | Bacteraemia  G1: 8/36 (22%)  G2: 1/7 (14%)  p= 1.00 | NR | NR | NR | NR | NR |
| Gong et al. 2012 [16]  NR/NR  China  Government, Academic/Grant from Jiangsu Provincial Administration of Chinese Medicine (LZ11091) and a special research fund of Organization Department of Jiangsu Provincial Party Committee, Talent Work Leading Group of Jiangsu Provincial Party Committee,Talent Work Leading Group of Jiangsu Province (333 High-level Talents Training Project)  Some concerns | Prospective controlled cohort study  11 months (February to December 2010)  G1: US  G2: non-US | 180 (NR)  Adults  Years as range  Overall: NR  G1: 22-79  G2: 26-74  Overall: 43%*  G1: 40%*  G2: 36%* | NR | NR | NR | Catheter-related local infection on day six to removal of catheter:  G1: 1/85 (1%)  G2: 3/90 (4%)  p= 0.57 | NR | Phlebitis on fifth day of catheterization:  G1: 1/90 (1%)  G2: 10/90 (11%)  p= 0.001 |
| Li et al. 2014 [17]  ChiCTR-TRC-12002749/NR  China  Academic: Guangdong Science and Technology Project of China  Some concerns | Open-label RCT  8 months (July 2011 to February 2012)  G1: US  G2: non-US | 100 (NR)  Adults  Overall: NR  >=60  G1: 7/50(14.0%)  G2: 5/48 (10.4%)  50-59  G1: 8/50 (16.0%)  G2: 16/48 (33.3%)  40-49  G1: 20/50 (40.0%)  G2: 16/48 (33.3%)  30-39  G1: 11/50 (22.0%)  G2: 9/48 (18.8%)  <=29  G1: 4/50 (8.0%)  G2: 2/48 (4.2%)  Overall: 27%*  G1: 30%  G2: 23% | CLABSI:  G1: 0/50 (0%)  G2: 1/48 (2%) | NR | NR | NR | G1: 0/50 (0%)  G2: 1/48 (2%) | Phlebitis, mechanical:  G1: 0/50 (0%)  G2: 11/48 (23%)  p=0.001 |
| Qi et al. 2012 [18]  NR/NR  China  NR  High | Open-label RCT  NR  G1: US  G2: non-US | 938 (NR)  Adults  NR  NR | NR | NR | NR | NR | NR | Phlebitis:  G1: 2/566 (0%)  G2: 22/372 (%)  p= 0.000 |
| Tan et al. 2016 [19]  ChiCTR-TRC-14004993/NR  China  NR  High | Single-blind RCT  24 months (May 2009 to April 2011)  G1: US  G2: non-US | 360 (NR)  Adults  Years  Overall: NR  G1: 57.45 (12.94)  G2: 58.09 (11.98)  Overall: 58%*  G1: 59%*  G2: 57%* | NR | NR | NR | Infection (local infection/CRBSI)  G1: 2/144 (1%*)  G2: 13/175 (7%*)  p= 0.015 | NR | Phlebitis:  G1: 2/144 (1%)  G2: 14/175 (8%)  p= 0.005 |
| Tang et al. 2012 [20]  NR/NR  China  NR  Some concerns | Prospective controlled cohort study  3 months (June to August 2011)  G1: US  G2: non-US | 216 (NR)  NR  NR  NR | NR | NR | NR | NR | NR | Phlebitis:  G1: 3/108 (3%)  G2: 18/108 (17%)  p= 0.001 |
| Wang et al. 2016 [21]  NR/NR  China  None: Grant Support & Financial Disclosures: None  Some concerns | Open-label RCT  36 months (January 2013 to December 2015)  G1: US  G2: non-US | 200 (NR)  Adults  Years  Overall: NR  G1: 52.1 (3.4)  G2: 53.1 (3.7)  Overall: 39%*  G1: 40%*  G2: 37%* | NR | NR | NR | NR | NR | Phlebitis:  G1: 2/100 (2%)  G2: 8/100 (8%)  p< 0.05 |
| Yin et al. 2022 [22]  NR/NR  China  NR  Some concerns | Open-label RCT  18 months (March 2020 to August 2021)  G1: US  G2: non-US | 94 (NR)  Neonates  Days  Overall: NR  G1: 14.64 (6.22)  G2: 15.10 (5.97)  Overall: 43%*  G1: 45%*  G2: 40%* | NR | NR | NR | NR | NR | Phlebitis:  G1: 0/47 (0%)  G2: 2/47 (4%) |
| Yuan et al. 2013 [23]  NR/NR  China  NR  Some concerns | Prospective controlled cohort study  6 months (January 2011 and June 2011)  G1: US  G2: non-US (modified Seldinger Technique) G3: non-US (blind) | 597 (NR)  Adults  Years  Overall: 59.61 (12.25)  G1: 60.9 (17.17)  G2: 57.9 (13.21)  G3: 56.7 (12.15)  Overall: 41%*  G1: 46%*  G2: 25%*  G3: 31%* | NR | NR | NR | Catheter-related infections  G1: 5/391 (1%)  G2: 2/24 (8%)  G3: 9/182 (5%)  p= 0.009 | NR | Phlebitis:  G1: 0/391 (0%)  G2: 0/24 (0%)  G3: 0/182 (0%)  p= 1.00 |
| Zhang et al. 2019 [24]  NR/NR  China  Government: Liangshan Prefecture Technology Development and Application Project of Sichuan Province  High | Open-label RCT  18 months (December 2016 to May 2018)  G1: US  G2: non-US | 95 (NR)  Adults  Years  Overall:NR  G1: 52.51  G2: 52.12  % female  Overall:45%  G1: 47%  G2: 44% | NR | NR | NR | NR | NR | Phlebitis:  G1: 0/48 (0%)  G2: 2/47 (4%) |

Abbreviations: BSI=bloodstream infection; CI=confidence interval; CLABSI=central line-associated bloodstream infection; CRVT=catheter-related venous thrombosis; G=group; IQR=interquartile range; KQ=key question; ml=milliliter; NR=not reported; OP=operation; p=p-value; PICC=peripherally inserted central catheter; PIVC=peripheral intravenous catheter; RCT=randomised controlled trial; RoB=risk of bias; RR=relative risk; SD=standard deviation; US=ultrasound; vs=versus

*self-calculated

**Age in mean (SD) unless otherwise specified

## Table E5: Lower vs upper arm

| **Authors, Year**  **Registration Number/Trial Name**  **Country**  **Funding**  **RoB** | **Study Design**  **Study Period or Duration**  **Study Arms** | **Number of Participants (Number of Catheters)**  **Population**  **Age****  **Female (%)** | **BSI** | **BSI-related Mortality** | **Sepsis** | **Local Infection** | **All-cause Morta-lity** | **Phlebitis/**  **Thrombophlebitis** |
| --- | --- | --- | --- | --- | --- | --- | --- | --- |
| Pongruangporn et al. 2013 [25]  NR  U.S.  Government: Centers for Disease Control and Prevention Epicenter Program  Some concerns | Case-control study  32 months (January 2006 to July 2008)  G1: lower arm (anticubital/forearm)  G2: upper arm | 647 (NR)  Adults  Median (IQR)  Overall cases: 58 (46–71)  Overall controls: 58 (47–71)  Overall cases: 49%  Overall controls: 50% | Hospital-acquired PICC BSIs:  G1: n= 113): 24 BSI, 89 no BSI  G2 (n= 534): 138 BSI, 396 no BSI;  Upper arm vs Anticubital/forearm: OR (95% CI) 1.29 (0.79 to 2.11)  p = 0.31  p <0.001 | NR | NR | NR | NR | NR |
| Razavinejad et al. 2023[26]  NR/NR  Iran  Academic: Shiraz University of Medical Sciences,  Shiraz, Iran  Some concerns | Restrospective controlled cohort study  37 months (August 2015 to August 2018)  G1: lower arm (median cubital vein)  G2: upper arm (cephalic vein) | 2500 (2500)  Neonates  Mean gestational age  Overall: 32  G1, G2: NR  NR | G1: NR  G2: NR  OR (95%CI): 1.36 (0.78 to 2.35) | NR | NR | NR | NR | NR |

Abbreviations: IQR=interquartile range; Abbreviations: BSI=bloodstream infection; CI=confidence interval; G=group; HR=hazard ratio; IRR=incidence rate ratio; IV=intravenous; KQ=key question; No=number; NR=not reported; OR=odds ratio; p=p-value; PICC=peripherally inserted central catheter; PIVC=peripheral intravenous catheter; RCT=randomised controlled trial; RoB=risk of bias; SD=standard deviation; vs=versus *self-calculated

**Age in mean (SD) unless otherwise specified

## Table E6: Upper vs lower limb

| **Authors, Year**  **Registration Number/Trial Name**  **Country**  **Funding**  **RoB** | **Study Design**  **Study Period or Duration**  **Study Arms** | **Number of Participants (Number of Catheters)**  **Population**  **Age****  **Female (%)** | **BSI** | **BSI-related Mortality** | **Sepsis** | **Local Infection** | **All-cause Mortality** | **Phlebitis/**  **Thrombophlebitis** | **Overall Adverse Events** |
| --- | --- | --- | --- | --- | --- | --- | --- | --- | --- |
| Aggarwal et al. 2001 [27]  NR  India  None  Some concerns | Retrospective cohort study  10 months  G1: upper limb  G2: lower limb | 38 (NR)  Neonates  Weeks gestational age  Overall: 29.7 (4)  G1, G2: NR  NR | NR | NR | NR | Infection (local/systemic):  G1: 1/18 (6%)  G2: 3/7 (43%) | NR | NR | Overall complications- per catheter:  G1: 4/18 (22%*)  G2: 4/7 (57%*)  Complication rate per 1000 CVC days  G1: 13/1000  G2: 44/1000 |
| Bashir et al. 2016 [28]  NR/NR  Canada  Foundation/Non-profit professional organization: Alberta Children’s Hospital Foundation  Some concerns | Retrospective cohort study  60 months (January 2006 to January 2011)  G1: upper limb  G2: lower limb | 827 (NR)  Neonates  Weeks gestational age  Overall: NR  G1: 28.8 (3.2)  G2: 28.9 (3.2)  Overall: 45%*  G1: 46%  G2: 44% | CLABSI  G1: 35/593 (6%)  G2: 10/234 (4%)  p= 0.35 | NR | NR | NR | NR | Phlebitis:  G1: 21/593 (4%)  G2: 9/234 (4%)  p= 0.83 | G1: 186/593 (31%)  G2: 61/234 (26%)  p= 0.13 |
| Bulbul et al. 2010 [29]  NR/NR  Turkey  NR  Some concerns | Prospective controlled cohort study  24 months (January 2005 to January 2007)  G1: upper limb  G2: lower limb | 139 (NR)  Neonates  Weeks  Overall: 31.3 (4.1)  G1, G2: NR  Overall: 41%  G1, G2: NR | NR | NR | NR | NR | NR | NR | All complications– per catheter  G1: 44/99 (44%)  G2: 12/21 (57%) |
| Callejas et al. 2016 [30]  NR/NR  Canada  NR  Some concerns | Retrospective controlled cohort study  42 months (January 2010 and June 2013)  G1: upper limb  G2: lower limb | NR (689)  Neonates, children  Days (range):  Overall: NR  G1: 29 (1-367)  G2: 16 (1 - 239)  G3: 17 (1–138)  NR | No of infectious complications (overall) per number of PICCs  G1: 56/471 (12%)  G2: 8/149 (5%) | NR | NR | NR | Death or transfer to other NICUs:  G1: 45/471 (10%)  G2: 18/149 (12%) | Phlebitis- per catheters  G1: 4 /471 (1%*)  G2: 1/149 (1%*) | Overall number of non-infectious complications:  G1: 59/471 (13%)  G2: 13/149 (9%)  p= 0.462 for UL  p= 0.126 for LL |
| Ekaputri et al. 2022 [31]  NR/NR  Indonesia  None  Some concerns | Prospective controlled-cohort study  27 months (November 2018 to December 2020)  G1: upper limb  G2: lower limb | 114  Neonates  Gestational age (weeks, %)-  Overall:  Term (≥37): 17%  Moderate preterm (32-36): 37%  Very preterm (28-31): 26%  Extremely preterm (<28): 20%  G1, G2: NR  Overall: 43%  G1, G2: NR | CLBSI:  G1: NR  G2: NR  RR (95% CI): 0.855 (0.34 to 2.15) | NR | NR | NR | NR | NR | NR |
| Elmekkawia et al. 2019 [32]  NR/NR  Canada  None  Some concerns | Retrospective cohort study  67 months (January 2005 to August 2010)  G1: upper limb  G2: lower limb | 365 (NR)  Neonates  Weeks gestational age in median (IQR)  Overall: NR  G1: 30 (26 - 35)  G2: 32 (27 - 37)  Overall: 42%*  G1: 40%  G2: 43% | NR | NR | Sepsis  G1: 9/138 (7%)  G2: 10/227 (4%) | NR | Mortality:  G1: 7/138 (5%)  G2: 14/227 (6%)  P=0.818 | Phlebitis:  G1: 1/138 (1%)  G2: 10/227 (4%) | NR |
| Gai et al. 2022 [33]  NR/NR  China  NR  Some concerns | Retrospective cohort study  10 months (March 2020 to December 2020)  G1: upper limb  G2: lower limb | 40 (NR)  Neonates  Age in groups  <= 15 days;  G1: 3/20  G2: 2/20  15-24 days  G1: 8/20  G2: 10/20  >= 25 days  G1: 9/20  G2: 8/20  Overall: 35%*  G1: 30%*  G2: 40%* | CRBSI:  G1: 1/20 (5%)  G2: 0/20 (0%)  p= 0.059 | NR | NR | NR | NR | Phlebitis:  G1: 2/20 (10%)  G2: 0/20 (0%)  p= 0.05 | NR |
| Hoang et al. 2008 [34]  NR/NR  U.S.  NR  Some concerns | Retrospective cohort study  48 months interval (June 2002 to June 2006)  G1: upper limb  G2: lower limb | 396 (NR)  Neonates  Days in median (range)  Overall: NR  G1: 6 (3–12)  G2: 8 (3–20)  Overall: 45%*  G1: 45%  G2: 44% | CRBSI:  per catheters  G1: 43/370 (12%)  G2: 10/107 (9%) | NR | NR | NR | NR | Phlebitis - per catheters  G1: 21/370 (6%)  G2: 6/107 (6%)  p=NS | NR |
| Hu et al. 2021 [35]  NR/NR  China  None  High | Retrospective cohort study  12 months (January 2020 to January 2021)  G1: upper limb  G2: lower limb | 366*  Neonates  NR  NR | CRBSI:  G1: 33/346 ( 10%*)  G2: 5/20 (25%)* | NR | NR | NR | NR | NR | NR |
| Kinoshita et al. 2019 [5]  NR/NR  Japan  NR  Some concerns | Prospective controlled cohort study  30 months (October 2014 to March 2017)  G1: upper limb  G2: lower limb | 2383 (NR)  Neonates  Weeks gestational age:  Overall: 28.5 (3.3)  Overall: 47%  G1, G2: NR | Central line−associated bloodstream infection:  lower limb vs upper limb: AHR (95% CI) 0.73 (0.40 to 1.32) | NR | NR | NR | NR | NR | NR |
| Kisa et al. 2015 [36]  NR/NR  Canada  NR  Some concerns | Retrospective cohort study  42 months (January 2010 to June 2013)  G1: upper limb  G2: lower limb | 692 (NR)  Neonates  Weeks gestational age in median (IQR)  Overall: NR  G1: 28 (26 - 32)  G2: 31 (27 - 35)  Overall: 44%*  G1: 40%  G2: 48% | NR | NR | NR | NR | NR | Infected thrombophlebitis:  G1: 0/485 (0%)  G2: 2/142 (1%) | NR |
| López Sastre et al. 2000 [37]  NR/NR  Spain  NR  Some concerns | Prospective controlled cohort study  18 months (July 1997 to December 1998)  G1: upper limb  G2: lower limb | 787 (939)  Neonates  Weeks gestational age:  Overall: 33.5 (4.8)  G1: 33.4 (4.8)  G2: 34 (5)  Overall: 47%  G1: 47*%  G2: 48*% | NR |  | Sepsis- per catheter  G1: 38/826(5%*)  G2: 2/82(2%*) | NR | NR  NR | Phlebitis-per catheter:  G1: 68/826 (8%*)  G2: 17/82 (21%*) | NR |
| Ma et al. 2015 [38]  NR/NR  U.S.  NR  High | Retrospective cohort study,  108 months (2004 to 2013)  G1: upper limb  G2: lower limb | 129 (NR)  Neonates  Weeks gestational age (range):  Overall: NR  G1: 36 (35 - 37)  G2: 37 (35 - 37)  NR | NR | NR | NR | Infections- per catheter:  G1: 0/89 (0%) *  G2: 0/40 (0%) * | NR | During silo reduction or within 5 days after abdominal closure:  Phlebitis- catheters (for Silo-reduction + Primary closure ) :  G1: 0/89 (0%*)  G2: 4/40 (10%*)  Phlebitis -catheters (Silo-reduction):  G1: 0/72 (0%)  G2: 3/26 (12%)  p < 0.01  Phlebitis -catheters (Primary closure)  G1: 0/17 (0%)  G2: 1/14 (7%)  p= NS  More than 5 days after abdominal closure:  Phlebitis- catheters (for Silo-reduction + Primary closure ) :  G1: 0/89 (0%*)  G2: 1/40 (3%*)  Phlebitis- catheters ( Primary closure ) :  G1: 0/17 (0%)  G2: 1/14 (7%)  p = NS  Thrombosis- catheters (for Silo-reduction + Primary closure ) :  G1: 1/89 (1%*)  G2: 0/40 (0%*)  Thrombosis- catheters ( Silo-reduction)  G1: 1/72 (1%)  G2: 0/26 (0%)  p= NS | Complications during silo reduction or within 5 days after abdominal closure:  G1: 3/89 (3%*)  G2: 8/40 (20%*)  Complications more than 5 days after abdominal closure:  G1: 11/89 (12%*)  G2: 7/40 (18%*) |
| Malinoski et al. 2013 [39]  NR/NR  U.S.  NR  Some concerns | Prospective controlled cohort study  36 months (2009 to 2012)  G1: upper limb (arm)  G2: lower limb (femoral) | 124* (NR)  Adults  Years  Overall: 56 (18)  G1, G2: NR  Overall: 32%  G1, G2: NR | NR | NR | NR | NR | NR | Phlebitis:  G1: 18/89 (20%)  G2: 5/35 (14%)  p< 0.001 | NR |
| Padilla-Sanchez et al. 2019 [40]  NR/NR  Spain  None  Some concerns | Retrospective cohort study  12 months (October 2014 to September 2015)  G1: upper limb  G2: lower limb | 116 (140)  Neonates  Weeks gestational age  Overall: 31.8 (5.3)  G1, G2: NR  Overall: 46%  G1, G2: NR | Catheter associated Bacteriemia:  G1: 8/94 (9%)  G2: 0/28 (0%) | NR | NR | NR | NR | Phlebitis- per catheters:  G1: 0/94 (0%)  G2: 1/28 (4%) | NR |
| Pet et al. 2020 [41]  NR/NR  U.S.  Academic: University of Washington Division of Neonatology for funding for statistical analysis  Some concerns | Retrospective cohort study  42 months (January 2012 to June 2015)  G1: upper limb  G2: lower limb | 1234 (NR)  Neonates  Days (IQR)  Overall: 7.1 (2.3 - 21.6)  G1, G2: NR  Overall: 465%  G1, G2: NR | NR | NR | NR | Infection- per catheter (without any details):  G1: 11/524 (2%)  G2: 7/710 (1%)  OR (95%CI): 2.15 (0.8 to 5.79)  p= NS | NR | Phlebitis/Edema/ Erythema/Perfusion changes- per catheter:  G1: 18/524 (3%)  G2: 44/710 (6%)  OR (95% CI): 0.54 (0.31 to 0.93)  p = 0.03  Associated with PICC clot -catheter:  G1: 18/524 (3%)  G2: 40/710 (5%)  OR (95%CI): 0.6 (0.33 to 1.06)  p= NS | Any complications- per catheter:  G1: 210/524 (40%)  G2: 215/710 (30%)  G1 vs G2: OR (95%CI): 1.54 (1.22 to 1.94) p< 0.001 |
| Tsai et al. 2009 [42]  NR/NR  Taiwan  NR  Some concerns | Retrospective cohort study  30 months (January 2004 to June 2006)  G1: upper limb  G2: lower limb | 518 (NR)  Neonates  Weeks gestational age:  Overall: NR  G1: 28.1 (2.5)  G2: 28.0 (2.6)  Overall: 44%*  G1: 43%*  G2: 45%* | NR | NR | Sepsis - per catheters:  G1: 34/278 (12%)  G2: 54/240 (23%)  p=0.002 | N | All-cause Mortality:  G1: 17*/278 (6%*)  G2: 11*/240 (4%*) | Phlebitis - per catheters:  G1: 29/278 (10%*)  G2: 0/240 (0%)  p < 0.001 | NR |
| Wrightson et al. 2013 [43]  NR/NR  U.S.  NR  High | Retrospective cohort study  72 months (2004 to 2009)  G1: upper limb  G2: lower limb | NR (626)  Neonates  NR  Overall: NR  G1: 41%  G2: 45% | NR | NR | Presumed sepsis:  G1: 31/374 (8%)  G2: 18/252 (7%)  p=0.6006 | NR | G1: 18/374 (5%)  G2: 3/252 (1%) | Phlebitis- per catheters  G1: 4/374 (1%)  G2: 5/252 (2%)  p=0.5 | NR |

Abbreviations: AHR=adjusted hazard ratio; BSI=bloodstream infection; CI=confidence interval; CLABSI=central line-associated bloodstream infection; G=group; HR=hazards ratio; IQR=interquartile range; IVC=intravenous catheter; KQ=key question; NICU=neonatal intensive care unit; No=number; NR=not reported; NS=non significant; p=p-value; OR=odds ratio; PICC=peripherally inserted central catheter; PIVC=peripheral intravenous catheter; RCT=randomised controlled trial; RoB=risk of bias; SD=standard deviation; vs=versus

*self-calculated

**Age in mean (SD) unless otherwise specified

## Table E7: Silicone vs nonsilicone

| **Authors, Year**  **Registration Number/Trial Name**  **Country**  **Funding**  **RoB** | **Study Design**  **Study Period or Duration**  **Study Arms** | **Number of Participants (Number of Catheters)**  **Population**  **Age****  **Female (%)** | **BSI** | **BSI-related Mortality** | **Sepsis** | **Local Infection** | **All-cause Mortality** | **Phlebitis/**  **Thrombophlebitis** |
| --- | --- | --- | --- | --- | --- | --- | --- | --- |
| Linder et al. 1984 [44]  NR/NR  Sweden  NR  High | Prospective controlled cohort study  NR  G1: silicone elastomer catheters  G2: polyethylene elastomere catheters | 61 (NR)  Adolescents, adults  Years in range  Overall: 17-89  G1, G2: NR  Overall: 38%*  G1, G2: NR | NR | NR | NR | NR | NR | Thrombophleibitiss- per catheter:  G1: 14/39 (36%*)  G2: 4/22 (18%*)  p= NS |
| Gomes de Souza et al. 2021 [45]  NR/NR  Brazil  NR  Some concerns | Retrospective cohort study  12 months (October 2018 to September 2019)  G1: Silicone  G2: Polyurethane | 449 (NR)  Neonates  Gestational age:  Overall: NR  G1: 30.99 (4.34)  G2: 30.84 (4.1)  Overall: 48%*  G1: 51%  G2: 45% | NR | NR | NR | NR | NR | Phlebitis:  G1: 1/203 (0%)  G2: 6/246 (2%)  p= 0.098 |
| Ong et al. 2010 [46]  NR/NR  Singapore, New Zealand, Australia  NR  Some concerns/Low | Open-label RCT  20 months (August 2005 to April 2007)  G1: distal valve silicone  G2: proximal valve polyurethane | 393 (NR)  Adults  Years  Overall: 50.4  G1: 51.9 (17.9)  G2: 49.0 (15.8)  Overall: 37%*  G1: 34%  G2: 40% | Definite CRBSI:  G1: 5/194 (3%)  G2: 2/198 (1.%)  Probable CRBSI  G1: 7/194 (4%)  G2: 2/198 (1.0%) | NR | NR | Exit site infections- per catheter:  G1: 4/194 (2%)  G2: 2/198 (1%) | NR | Phlebitis- per catheter:  G1: 45/194 (23%)  G2: 23/198 (12%)  p= 0.003 |

Abbreviations: BSI=bloodstream infection; G=group; KQ=key question; NR=not reported; NS=not significant; p=p-value; PICC=peripherally inserted central catheter; PIVC=peripheral intravenous catheter; RCT=randomised controlled trial; RoB=risk of bias; SD=standard deviation; vs=versus

*self-calculated

**Age in mean (SD) unless otherwise specified

## Table E8: Occlusive vs non-occlusive dressings

| **Authors, Year**  **Registration Number/Trial Name**  **Country**  **Funding**  **RoB** | **Study Design**  **Study Period or Duration**  **Study Arms** | **Number of Participants (Number of Catheters)**  **Population**  **Age****  **Female (%)** | **BSI** | **BSI-related Mortality** | **Sepsis** | **Local Infection** | **All-cause Mortality** | **Phlebitis/**  **Thrombo-phlebitis** |
| --- | --- | --- | --- | --- | --- | --- | --- | --- |
| Chico-Padron et al. 2011 [47]  NR/NR  Spain  NR  Some concerns | Open-label RCT  9 months (July 2007 to March 2008)  G1: occlusive dressing (transparent)  G2: non-occlusive dressing (gauze) | 75 (NR)  Adults  Years  Overall: NR  G1: 58 (21)  G2: 62 (13.1)  NR | NR | NR | NR | NR | NR | Phlebitis- per catheter:  G1: 2/12 (17%*)  G2: 1/13 (8%*) |

Abbreviations: BSI=bloodstream infection; G=group; IV= intravenous; IVC=intravenous catheter; NR=not reported; NS=not significant; p=p-value; PICC=peripherally inserted central catheter; PIVC=peripheral intravenous catheter; RCT=randomised controlled trial; KQ=PICO (Population, Intervention, Comparison, Outcome) question; RoB=risk of bias; SD=standard deviation; UK=United Kingdom; U.S.=United States of America; vs=versus

*self-calculated

**Age in mean (SD) unless otherwise specified

## Table E9: Insertion team vs no specific insertion team

| **Authors, Year**  **Registration Number/Trial Name**  **Country**  **Funding**  **RoB** | **Study Design**  **Study Period or Duration**  **Study Arms** | **Number of Participants (Number of Catheters)**  **Population**  **Age****  **Female (%)** | **BSI** | **BSI-related Mortality** | **Sepsis** | **Local Infection** | **All-cause Mortality** | **Phlebitis/**  **Thrombophlebitis** | **Overall Adverse Events** |
| --- | --- | --- | --- | --- | --- | --- | --- | --- | --- |
| Levit et al. 2020 [48]  NR/NR  U.S.  None  High | Before-after study  32 months (January 2009 to July 2011)  G1: insertion team/did insertion (specialized PICC-team nurses)  G2: no insertion team (nurses before specialized training) | 731 (1255)  Neonates  Weeks gestational age  Overall: NR  G1: 30.5 (5.3)  G1: 29.9 (5.2)  Overall: 51%*  G1: 45%  G2: 56% | CLABSI (rate (95% CI)):  G1: 0.3 (0.8 to 3.1)  G2: 1.6 (0.01 to 1.2)  RR (95% CI): 0.43 (0.08 to 2.34) | NR | NR | NR | NR | Phlebitis (rate (95% CI))-NR  G1: 0.5 (0.15 to 1.4)  G2: 4.9 (3.3 to 7.1)  RR (95% CI) 0.10 (0.03 to 0.31) | Any complication:  Rate (95% CI)  G1: 5.5 (4.0 to 7.7)  G2: 12.8 (10.1 to 16.1)  RR (95% CI) 0.43 (0.29 to 0.65) |
| Pitts et al. 2013 [49]  NR/NR  U.S.  NR  High | Before-after study  2 years (2008 to 2010)  G1: insertion team/did insertion (specialized nurse-led pediatric vascular access team)  G2: no insertion team (no specialized training; physicians, surgeons, and radiologists) | 669 (NR)  Children, adolescents  Years  Overall: 9  G1, G2: NR  NR | CLABSI:  G1: 2.0 / 1000 catheter line days (second quarter of 2010)  G2: 9.12 / 1000 catheter line days (before 2009) | NR | NR | NR | NR | NR | NR |
| Yongshu et al. 2019 [50]  NR/NR  China  NR  High | Before-after study  16 months (September 2017 to December 2018)  G1: insertion team / did insertion (specialized insertion and maintenance team of nurses)  G2: no insertion team (nurses without specialized training) | 140 (NR)  Neonates  Gestational age: weeks: mean (SD)  Overall: NR  G1: 29.85 (1.30)  G2: 30.26 (1.42)  Overall: 48%*  G1: 48%  G2: 47% | NR | NR | NR | NR | NR | Phlebitis:  G1: 4/70 (6%)  G2: 12/70 (17%)  p= 0.034 | NR |

Abbreviations: BSI=bloodstream infection; CI=confidence interval; G=group; IRR=incidence rate ratio; IV=intravenous; IVC=intravenous catheter; NR=not reported; OR=odds ratio; p=p-value; KQ=PICO (Population, Intervention, Comparison, Outcome) question; RCT=randomised controlled trial; RoB=risk of bias; RR=relative risk; SD=standard deviation; U.S.=United States; vs=versus

*self-calculated

**Age in mean (SD) unless otherwise specified

## Table E10: Scalp inserted vs anywhere other than the scalp

| **Authors, Year**  **RoB** | **Study Design**  **Study Duration (Period)**  **Catheter Type**  **Intervention Arms** | **Population**  **Age****  **Female (%)** | **BSI** | **BSI-related Mortality** | **Sepsis** | **Local Infection** | **All-cause Mortality** | **Phlebitis/**  **Thrombophlebitis** |
| --- | --- | --- | --- | --- | --- | --- | --- | --- |
| Aggarwal et al. 2001 [27]  NR  India  None  Some concerns | Retrospective cohort study  10 months (NR)  G1: catheters inserted in scalp  G2: catheters inserted in the upper limb  G3: catheters inserted in the lower limb | 38 (NR)  Neonates  Weeks gestational age  Overall: 29.7 (4.3)  G1, G2, G3: NR  NR | NR | NR | NR | Infection (local/systemic)- per catheter:  G1: 2/19 (11%)  G2: 1/18 (6%)  G3: 3/7 (43%) | NR | NR |
| Callejas et al. 2016 [30]  NR/NR  Canada  NR  Some concerns | Retrospective cohort study  42 months (January 2010 to June 2013)  G1: catheters inserted in scalp  G2: catheters inserted in the upper limb  G3: catheters inserted in the lower limb | NR (689)  Neonates, children  Days (range):  Overall: NR  G1: 29 (1-367)  G2: 16 (1-239)  G3: 17 (1–138)  NR | No. of infectious complications (overall)- per catheter  G1: 5/69 (7%)  G2: 56/471 (12%)  G3: 8/149 (5%)  p= 0.335 for G1 vs G2 (reported per 1000 catheter days only)  p= 0.535 for G1 vs G3 (reported per 1000 catheter days only) | NR | NR | NR | Death or transfer to other NICUs- per catheter:  G1: 9/69 (13%)  G2: 45/471 (10%)  G3: 18/149 (12%) | Phlebitis- per catheter:  G1: 0/69 (0%)  G2: 4 /471 (0.8%*)  G3: 1/149 (0.7%*)  p= 0.462 for G1 vs G2  G1 vs G3: p= 0.126 (reported for overall non-infectious complications) |
| Kisa et al. 2015 [36]  NR/NR  Canada  NR  Some concerns | Retrospective cohort study  42 months (January 2010 to June 2013)  G1: catheters inserted in scalp  G2: catheters inserted in the upper limb  G3: catheters inserted in the lower limb | 692 (NR)  Neonates  Weeks gestational age in median (IQR)  Overall: NR  G1: 37 (34- 38)  G2: 28 (26- 32)  G3: 31 (27- 35)  Overall: 45%*  G1: 47%  G2: 40%  G3: 48% | NR | NR | NR | NR | NR | Infected thrombophlebitis:  G1: 0/65 (0%)  G2: 0/485 (0%)  G3: 2/142 (1%) |
| López Sastre et al. 2000 [37]  NR/NR  Spain  NR  Some concerns | Prospective controlled cohort study  18 months (July 1997 to December 1998)  G1: catheters inserted in scalp  G2: catheters inserted in the upper limb  G3: catheters inserted in the lower limb | 787 (939)  Neonates  Weeks gestation age:  Overall: 33.5 (4.8)  G1: 33.9 (4.8)  G2: 33.4 (4.8)  G3: 34 (5)  Overall: 47%  G1: 42*%  G2: 47*%  G3: 48*% | NR | NR | Sepsis- catheters- out of total sepsis-related- per catheter:  G1: 1/41 (2%)  G2: 38/41 (93%)  G3: 2/41 (5%)  p= NS | NR | NR | Phlebitis-per catheter:  G1: 5/31 (16%*)  G2: 68/826 (8%*)  G3: 17/82 (21%*) |
| Padilla-Sanchez et al. 2019 [40]  NR/NR  Spain  None: This work has not received any scholarship or grant  Some concerns | Retrospective cohort study  12 months (October 2014 to September 2015)  G1: catheters inserted in scalp  G2: catheters inserted in the upper limb  G3: catheters inserted in the lower limb | 116 (140)  Neonates  Weeks gestational age:  Overall: 31.8 (5.3)  G1, G2, G3: NR  Overall: 46%  G1, G2, G3: NR | CRBSI:  G1: 0/18 (0%)  G2: 8/94 (9%)  G3: 0/28 (0%) | NR | NR | NR | NR | Phlebitis- per catheters:  G1: 2/18 (11%)  G2: 0/94 (0%)  G3: 1/28 (4%) |

Abbreviations: BSI=bloodstream infection; CRBSI=catheter-related bloodstream infection; G=group; IQR: interquartile range; IV=intravenous; KQ: key question; NICU: neonatal intensive care unit; NR: not reported; NS=non-significant; PICC: peripherally inserted central catheter; PIVC: peripheral intravenous catheter; RCT: randomised controlled trial; RoB: risk of bias; SD: standard deviation; vs: versus

*self-calculated

**Age in mean (SD) unless otherwise specified

## Table E11: Saline flushing/locking after product administration vs anticoagulant flushing

| **Authors Year**  **Registration Number/Trial Name**  **Country**  **Funding**  **RoB** | **Study Design**  **Study Duration (Period)**  **Study Arms** | **Number of Participants (Number of Catheters)**  **Population**  **Age****  **Female (%)** | **BSI** | **BSI-mortality** | **Sepsis** | **Local Infection** | **All-cause Mortality** | **Phlebitis/**  **Thrombophlebitis** |
| --- | --- | --- | --- | --- | --- | --- | --- | --- |
| Araujo et al. 2011 [51]  NR/NR    Brazil    NR  High | Open-label RCT    15 months    G1: saline flushing  G2: heparin flushing 10 U/m | 133 (NR)  Neonates    Days  Overall: NR  G1:3.57 (18.7)  G2: 3.63 (15.7)  Overall: 49%*  G1: 48%*  G2: 50%* | CRBSI  G1: 1/69 (1%*)  G2: 0/64 (0%*) | NR | G1: 17/69 (25%*)  G2: 11/64 (17%*) | NR | NR | NR |

Abbreviations: BSI=bloodstream infection; CI=confidence interval; CRBSI=catheter-related bloodstream infection; CVC=central venous catheters; G=group; IQR=interquartile range; IVC=intravenous catheter; KQ=key question; NR=not reported; OR=odds ratio; p=p-value; PICC=peripherally inserted central catheter; PIVC=peripheral intravenous catheter; RCT=randomised controlled trial; RoB=risk of bias; SD=standard deviation; U/ml=units/milliliter; vs=versus

*self-calculated

**Age in mean (SD) unless otherwise specified

## Table E12: Use of closed-access device system (e.g., luer lock) vs open-access system

| **Authors, Year**  **Registration Number/Trial Name**  **Country**  **Funding**  **RoB** | **Study Design**  **Study Period or Duration**  **Study Arms** | **Number of Participants (Number of Catheters)**  **Population**  **Age****  **Female (%)** | **BSI** | **BSI-related mortality** | **Sepsis** | **Local Infection** | **All-cause Mortality** | **Phlebitis/**  **Thrombophlebitis** |
| --- | --- | --- | --- | --- | --- | --- | --- | --- |
| Morano et al. 2015 [52]  NR/NR  Italy  NR  Some concerns | Retrospective controlled cohort study  48 months (January 2009 to December 2012)  G1: closed system  G2: open system | 483 (612)  Adults  Median  Overall: 54.7  G1, G2: NR  Overall: 52%  G1, G2: NR | CRBSI:  G1, G2: NR  HR (95% CI): 0.71 (0.53 to 1.71) | NR | NR | NR | NR | NR |
| Reiter et al. 2006 [53]  NR/NR  U.S.  Government, Academic: University of Colorado Hospital Board of Directors Research Grant and General Clinical Research Centers Program National Center for Research Resources NIH  Some concerns | Prospective controlled cohort study  60 months (1999 to 2003)  G1: closed system  G2: open system | 300 (NR)  Neonates  Weeks gestational age  Overall: NR  G1: 29.7(4.3)  G2: 32( 5.3)  NR | CRBSI:  G1: 37*/150 (25%*)  G2: 29*/150 (20%*) | NR | Sepsis, mean (%)  G1: 1.3/150 (0.7%)  G2: 1.2/150 (0.8%)  p= 0.55 | NR | NR | NR |
| Rundjan et al. 2015 [54]  NR/NR  Indonesia  Hospital/Hospital research grant  Some concerns | Open-label RCT  4 months (June to September 2013)  G1: closed system  G2: open system | 60 (NR)  Neonates  Weeks gestational ageOverall: NR  G1: 32.4 (1.81)  G2: 32.6 (2.1)  Overall: NR  G1: 53%  G2: 57% | NR | NR | Confirmed sepsis:  G1: 1/30 (3%)  G2: 8/30 (27%)  p = 0.026  Relative risk (95% CI): 0.095 (0.011 to 0.85)  Presumed sepsis  G1: 13/30 (43%)  G2: 23/30 (77%)  Probable sepsis  G1: 7/30 (23%)  G2: 18/30 (60%) | NR | NR | NR |
| Yu et al. 2024 [55]  NR/NR  China  Foundation: National Natural Science Foundation  of China and National High Level Hospital Clinical  Research Funding  Some concerns | Prospective controlled cohort study  19 months (December 2020  to June 2022)  G1: closed system  G2: open system | 3166 (3166)  Adults  Overall: 58.28  G1: 57.76  G2: 60.03  Overall: 54%*  G1: 59%*  G2: 36%* | Infections (CRBSI, local infections and local inflammation):  G1: 382*/2436 (15.7%)  G2: 196*/730 (26.9%) | NR | NR | NR | NR | NR |
| Zerla et al. 2015 [56]  NR/NR  Italy  NR  High | Retrospective cohort study  46 months (March 2010 to December 2013)  G1: closed system  G2: open system | NR (793)  Adults  NR  NR | CRBSI- per catheter: G1: 2/620 (0.3%)  G2: 5/173 (3%) | NR | NR | NR | NR | NR |

Abbreviations: BSI=bloodstream infection; CI=confidence interval; CRBSI=catheter-related bloodstream infection; G=group; KQ=key question; N=number; NR=not reported; p=p-value; PICC=peripherally inserted central catheter; PIVC=peripheral intravenous catheter; RCT=randomised controlled trial; RoB=risk of bias; SD=standard deviation; vs=versus

*self-calculated

**Age in mean (SD) unless otherwise specified

## Table E13: Single lumen vs. multi-lumen

| **Authors Year**  **Registration Number/Trial Name**  **Country**  **Funding**  **RoB** | **Study Design**  **Study Period or Duration**  **Study Arms** | **Number of Participants (Number of Catheters)**  **Population**  **Age****  **Female (%)** | **BSI** | **BSI-related Mortality** | **Sepsis** | **Local Infection** | **All-cause Mortality** | **Phlebitis/**  **Thrombophlebitis** |
| --- | --- | --- | --- | --- | --- | --- | --- | --- |
| Al Raiy et al. 2010 [57]  NR/NR  U.S.  NR  Some concerns | Prospective controlled cohort study  17 months (May 2006 to September 2007)  G1: single lumen catheters  G2: multi-lumen catheters | 622 (NR)  NR  NR  NR | CLABSI  G1: 0/108 (0%)  G2: 13/514 (25*%) | NR | NR | NR | NR | NR |
| Bae et al., 2023 [58]  NR/NR  South Korea  Academic: Biomedical Research Institute, Jeonbuk National University Hospital  Some concerns | Retrospective controlled cohort study  3 months (September 2019 to November 2019)  G1: single lumen catheters  G2: multi-lumen catheters | 126 (126)  Adults  Overall: 71.0  G1, G2: NR  Overall: 50%  G1, G2: NR | CLBSI:  G1: 3/73 (4.1%)  G2: 2*/53* (3.8%*) | NR | NR | NR | NR | NR |
| Barrigah‑Benissan et al. 2023 [59]  NR/NR  France  None  Some concerns | Retrospective controlled cohort study  12 months (April, 2018 to April, 2019  G1: single lumen catheters  G2: double lumen catheters | 783 (901)  Adults  Median (IQR)  Overall: 70.9 (59.4–79.8)  G1, G2: NR  Overall: 44.5%  G1, G2: NR | G1: 43/712 (6.0%*)  G2: 30/189 (15.9%) | NR | NR | NR | NR | NR |
| Chopra et al. 2014 [60]  NR/NR  U.S.  Academic: ocally Initiated Project (LIP)  Award #41-127 from the Center for Clinical Management Research, VA  Ann Arbor Healthcare System.  Some concerns | Retrospective controlled cohort study  36 months (June 2009 to  July 2012)  G1: single lumen catheters  G2: multi lumen catheters | 747 (966)  Adults  NR  NR | CLABSI:  G1: 8/459 (1.8%*)  G2: 50/507 (9.9%*)  Dual vs single lumen: OR (95% CI) 3.99 (1.46 to 10.94)  Triple vs single lumen: OR (95% CI) 6.34 (1.85 to 21.71) | NR | NR | NR | NR | NR |
| Khalidi et al. 2009 [61]  NR/NR  U.S.  NR  High | Prospective cohort study  NR  G1: single lumen+ standard cap  G2: dual lumen + standard cap | 80 (NR)  Adults  NR  NR | CRBSI:  G1: 0/9 (0%)  G2: 0/43 (0%) | NR | NR | NR | NR | NR |
| Kinoshita et al. 2019 [5]  NR/NR  Japan  NR  Some concerns | Prospective controlled cohort study  30 months (October 2014 to March 2017)  G1: single lumen catheters  G2: multi-lumen catheters | 2383 (NR)  Neonates  Gestational age in weeks:  Overall: 28.5 (3.3)  G1, G2: NR  Overall: 47%  G1, G2: NR | CLABSI:  G1: NR  G2: NR  G1 vs G2:  aHR (95% CI) 0.39 (0.13 to 1.16) | NR | NR | NR | NR | NR |
| Liscynesky et al. 2017 [62]  NR/NR  U.S.  NR  Some concerns | Prospective controlled cohort study  9 months (November through July 2011)  G1: single lumen catheters  G2: multi-lumen catheters | 187 (NR)  Adults  Overall: 50 (14)*  G1, G2: NR  *based on 187 total population, not 182 single + dual  Overall: 48%  G1, G2: NR  *based on 187 total population, not 182 single + dual | NR | NR | NR | G1: 6*/112 (5%*)  G2: 11/75 (15%*)  p= 0.04 | NR | NR |
| Pongruangporn et al. 2013 [25]  NR  U.S.  Government: Centers for Disease Control and Prevention Epicenter Program  Some concerns | Case-control study  30 months (January 2006 to July 2008)  G1: single lumen  G2: dual lumen  G3: triple lumen | 647 (NR)  Adults  Years in median (IQR)  Overall cases: 58 (46–71)  Overall controls: 58 (47–71)  G1, G2: NR  Overall cases: 49%  Overall controls: 50%  G1, G2: NR | Hospital-acquired PICC BSIs (cases, controls):  G1 (n=237): 33 BSI, 204  no BSI  G2 (n=311): 87 BSI, 224 no BSI  G3 (n=98): 41 BSI, 57 no BSI  G2 vs G1: OR (95% CI) 2.40 (1.54 to 3.74)  p < 0.001  G3 vs G1 OR (95% CI) 4.45 (2.58 to 7.66)  p< 0.001 | NR | NR | NR | NR | NR |
| Rabelo-Silva et al. 2022 [63]  NR/NR  Brazil  Academic: Hospital de Clínicas de Porto Alegre Research and Events Incentive Fund (number 2018-0267)  Some concerns | Prospective controlled cohort study  23 months (October 2018 to August 2020)  G1: single lumen catheters  G2: multi-lumen catheters | 11135 (12725)  Adults  Years  Overall: 66.4 (19)  G1, G2: NR  Overall: 51%  G1, G2: NR | Confirmed/suspected CLABSI- per catheter:  G1: 165/4989 (3%)  G2: 511/7736* (7%)  OR (95%CI) 2.08 (1.62 to 2.67)  p< 0.001  DVT+CLABSI-per catheters:  G1: 199 (4%)  G2: 602 (8%)  OR (95% CI) 2.13 (1.67 to 2.72)  p< 0.001 | NR | NR | NR |  | NR |

Abbreviations: AHR=adjusted hazard ratio; BSI=bloodstream infection; CI=confidence interval; CLABSI=central line-associated bloodstream infection; DVT=deep vein thrombosis; G=group; IQR=interquartile range; KQ=key question; NR=not reported; OR=odds ratio; p=p-value; PICC=peripherally inserted central catheter; PIVC=peripheral intravenous catheter; RCT=randomised controlled trial; RoB=risk of bias; SD=standard deviation; vs=versus

*self-calculated

**Age in mean (SD) unless otherwise specified

## Table E14: Bundle studies

| **Authors, Year**  **Registration Number/Trial Name**  **Country**  **Funding**  **RoB** | **Study Design**  **Study Period or Duration**  **Study Arms** | **Number of Participants (Number of Catheters)**  **Population**  **Age****  **Female (%)** | **BSI** | **BSI-related Mortality** | **Sepsis** | **Local Infection** | **All-cause Mortality** | **Phlebitis/**  **Thrombophlebitis** | **Overall adverse events** |
| --- | --- | --- | --- | --- | --- | --- | --- | --- | --- |
| Bayoumi, 2021[64]  NR/NR  Qatar  Fundation: Medical Research Center  High | Before after study  3 years (2016 to 2018)  G1: insertion kit, occlusive dressing, closed system  G2: no intervention | 1336 (NR)  Neonates  Gestational age:  22–28 weeks  G1: 38%  G2: 46  G3: 54%  >28–32 weeks  G1: 39%  G2: 42  G3: 28%  >32–36 weeks  G1: 8%  G2: 7%  G3: 8%  >36weeks  G1: 16%  G2: 5%  G3: 10%  NR | G1 (2017): 16/508 (3.14%)*  G1 (2018) 14/450 (3.11%)*  G2 (2016): 10/378 (2.6%)*  p= 0.895    CLABSI Rate:  G1 (2017): 2.46/ 1000 catheter-days  G1 (2018): 2.34/ 1000 catheter-days  G2: 2.27/ 1000 catheter-days  p = 0.978 | NR | NR | NR | G1 (2017): 17/508 (3.34%)*  G1 (2018): 13/450 (2.88%)*  G2: 9/378 (2.38%)*  p= 0.699 | NR | NR |
| Costa, 2016[65]  NR/NR  Brasil  NR  High | Prospective cohort study  25 months (August, 2010 to August, 2012)  G1: 1,9 Fr silicone single lumen catheters  G2: 2,0 Fr polyurethane double-lumen catheters | 383 (401)  Neonates  Postnatal age- days  Overall: 7.6  G1, G2: NR  Overall: 55%  G1, G2: NR | G1: 13*/185* (7.0%*)  G2: 59/216 (27.3%)  RR (95% CI) G2 vs. G1: 3.7 (2.1 to 6.5)  p < 0.001 | NR | NR | NR | NR | NR | NR |
| Eturajulu, 2022[66]  NR/NR  Malaysia  Axademic: University of Malaya Medical Centre  High | Before after study  18 months (October 2019 to March 2021)  G1: insertion trainning, insertion team  G2: no intervention | 102 (102)  Adults  Years  Overall: 59.91 (16.32)  G1, G2: NR  Overall: 43%*  G1, G2: NR | G1: 4*/68 (5.9%)  G2: 3*/34 (8.8%)  p= NR  CLABSI per 1000 catheter days:  G1: 2.7 per 1000 catheter days  G2: 3.4 per 1000 catheter days  p= NR | NR | NR | NR | NR | NR | NR |
| Golombek, 2002[67]  NR/NR  US  NR  Some concerns | Before after study  39 months (1993 to 1995 and February 1998 to May 1999)  G1: sterile insertion, occlusive dressing, insertion team, clinically indicated removal  G2: no intervention | 136 (189)  Neonates  Gestational age (weeks):  Overall: NR  G1: 25.6  G2: 26  Age at insertion (days):  Overall: NR  G1: 11.4  G2: 12.5  NR | G1 (1998-1999): 7.1%- 5.1 infections per 1000 catheter days (1 infection/194 days)  G2 (1993-1995): 25%- 15.8 infections per 1000 catheter days (1 infection/63 days) | NR | G1: 1/27 (4%)  G2: 23/57 (40%) | NR | G1: 3/27 (11%)  G2: 0/57 (0%)  p< 0.05 | NR | NR |
| Harnage, 2007[68]  NR/NR  US  NR  High | Before after study  15 months (January 2006 to March 2007)  G1: sterile insertion, CHD, US guided insertion, occlusive dressing, flushing, closed system  G2: no intervention | NR (2850)  Neonates  NR  NR | G1: 0/2083 (0%*)  G2: 11/767 (1.43%*) | NR | NR | NR | NR | NR | NR |
| He, 2022[69]  NR/NR  China  Foundation: ChenzhouFirst People’s Hospital in Hunan  High | RCT  19 months (July 2020 to January 2022)  G1: US guided insertion, occlussive dressing  G2: routine care | 100 (NR)  Adults  Years  Overall: NR  G1: 44.2  G2: 55.3  Overall: NR  G1: 44%*  G2: 40%* | NR | NR | NR | G1: 1/50 (2%)  G2: 3/50 (6%)  p< 0.05 | NR | G1: 0/50 (0%)  G2: 1/50 (2%)  p< 0.05 | G1: 2*/50 (4.5%)  G2: 9/50 (18%)  p< 0.05 |
| Kaplan, 2011[70]  NR/NR  Canada  Government: Center for Medicare and Medicaid Services a  High | ITS  15 months (September 2008 to December 2009)  G1: sterile insertion, sterile gloves, flushing, closed system  G2: no intervention | 1916 (NR)  Neonates  Gestational age (weeks)  Overall: 22-29  G1, G2: NR  NR | Bacterial bloodstream infection- average infection rate:  G1: 14.3%  G2: 18.2% | NR | NR | NR | NR | NR | NR |
| Liu, 2013[71]  NR/NR  China  NR  High | RCT  24 months (January 2011 to January 2013)  G1: sterile insertion, sterile gloves, US guided, lower arm, silicone catheter, occlussive dressing  G2: routine care | 1490 (NR)  Adults  NR  NR | NR | NR | NR | G1: 29/732 (3.96%)  G2: 64/758 (8.44%)  p= 0.001 | NR | G1: 23/732 (3.14%)  G2: 68/758 (8.97%)  p= 0.023 | NR |
| Paiva, 2013 [72]  NR/NR  Brasil  Government: The National Council for Scientific and Technological Development  Some concerns | Prospective cohort study  12 months (July 2010 to June 2011)  G1: 1,9 Fr Silicone single-lumen catheters  G2: 2Fr Polyurethane double lumen catheters | 270 (270)  Neonates  Chronological age (average days)  Overall: NR  G1: 11.3  G2: 8.5  Overall: 41%  G1: 36%  G2: 46% | NR | NR | NR | NR | NR | NR | G1: 67*/189 (35.4%)  G2: 37*/81 (45.6%)  p= 0.11 |
| Ren, 2022[73]  NR/NR  China  NR  High | Retrospective controlled cohort study  36 months (January 2019 and December 2021)  G1: training, saline flushing or heparin flushing during the night, occlusive dressing  G2: routine care | 102 (NR)  Neonates  Mean days of age  Overall: NR  G1: 6.43  G2: 6.16  Overall: NR  G1: 51%  G2: 53% | Intravascular  catheter  Infection  G1: 1/51 (1.96%)  G2: 3/51 (5.88%) | NR | NR | G1: 1/51 (1.96%)  G2: 2/51 (3.92%) | NR | G1: 0/51 (0%)  G2: 2/51 (3.92%) | G1: 4/51 (7.84%)  G2: 13/51 (25.49%) |
| Royer, 2010[74]  NR/NR  US  NR  High | Prospective cohort study  79 months (January 2003 to June 2009)  G1: sterile insertion, CHD, insertion team, flushing  G2: no intervention | NR (NR)  Neonates  NR  NR | G1: 0 per 1000 central line days  G2: 1.73 per 1000 central line days | NR | NR | NR | NR | NR | NR |
| Steiner, 2015[75]  NR/NR  Austria  None  High | Before after study  36 months (2010 to 2012)  G1: sterile insertion, insertion trainning, sterile gloves, insertion kit  G2: no intervention | 526 (NR)  Neonates  Patient days  Overall: NR  G1 (2011): 5863  G2 (2012): 7096  G3 (2010): 5620  Overall: 50%*  G1 (2011): 55%*  G1 (2012): 45%*  G2 (2010): 50%* | G1: 34/161 (21.1%*)  G1 (2012): 18/197 (9.1%*)  G2 (2010): 50/168 (29.8%*)  G1 (2011): 9.5 per 1000 central line days  G1 (2012): 4.7 per 1000 central line days  G2 (2010): 13.9 per 1000 central line days  p < 0.0001 | NR | NR | NR | NR | NR | NR |
| Tian, 2010[76]  NR/NR  China  Foundation: Science and Technology Grants for Medicine and Health Research  Some concerns | Before after study  50 months (August 2004 to November 2006 and January 2007 to October 2008)  G1: sterile insertion, CHD, insertion training, sterile gloves, sterile access protocol  G2: no intervention | 232 (232)  Adults  Years:  Overall: NR  ≤40- G1: 24%;  G2: 29%  41–50- G1: 42%;  G2: 27%  51–60- G1: 18%;  G2: 22%  61–70- G1: 9%;  G2: 14%  >70- G1: 8%;  G2: 8%  Overall: 44%*  G1: 39%  G2: 48% | G1: 3/165 (1.81%)  G2: 4/69 (5.8%)  p=NR | NR | NR | G1: 2/165 (1.21%)  G2: 3/69 (4.35%)  p=NR | NR | G1: 5/165 (3.03%)  G2: 5/69 (7.25%)  p=NR | G1: 19/165 (11.51%)  G2: 21/69 (30.43%)  p= 0.0004 |
| Tong, 2011[77]  NR/NR  China  NR  High | Before after study  38 months (July 2004 to October 2009)  G1: sterile insertion, sterile gloves, insertion training  G2: no intervention | 3020 (NR)  Adults, children  Years- avarage (range)  Overall: NR  G1: 58 (3-91)  G2: 62 (1-101)  Overall: 54%*  G1: 52%*  G2: 55%* | G1: 2.9 ‰ (Cases/catheter days: 29/99643)  G2: 6.0 ‰ (Cases/catheter days: 48/79793)  p=0.000 | NR | NR | NR | NR | NR | NR |

Abbreviations: aHR=adjusted hazard ratio; aRR=adjusted relative risk; BSI=bloodstream infection; CABSI=catheter-associated bloodstream infection; CHD=chlorhexidine; CI=confidence interval; CLABSI=central line-associated bloodstream infection; CRBSI= catheter-related bloodstream infection; G=group; KQ=key question; NR=not reported; p=p-value; PICC=peripherally inserted central catheter; PIVC=peripheral intravenous catheter; RCT=randomised controlled trial; RoB=risk of bias; SD=standard deviation; vs=versus

*self-calculated

**Age in mean (SD) unless otherwise specified

# Supplement F- Risk of Bias Ratings of Included Studies

Figure F1
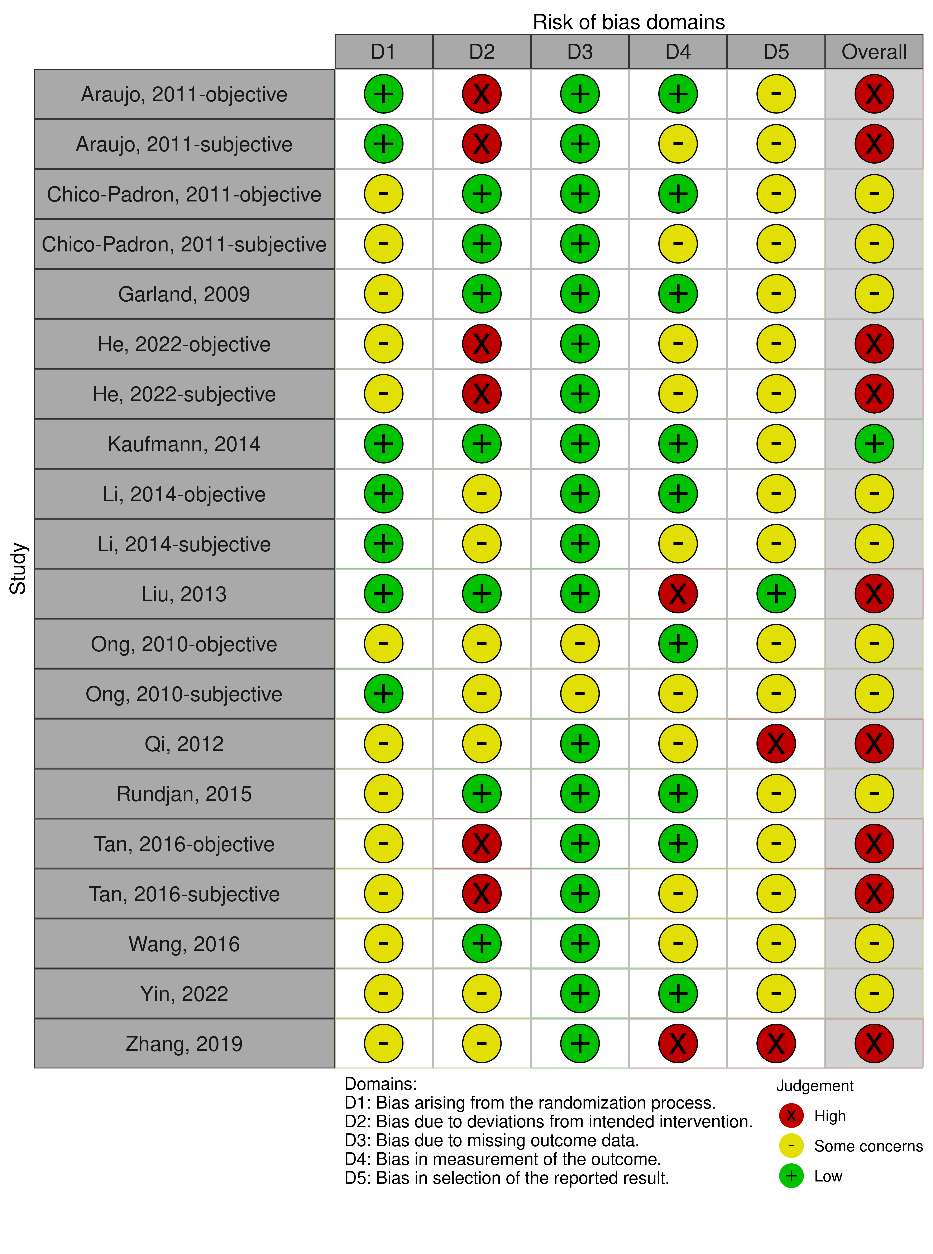
. Risk of Bias Ratings for RCTs (RoB2 tool)


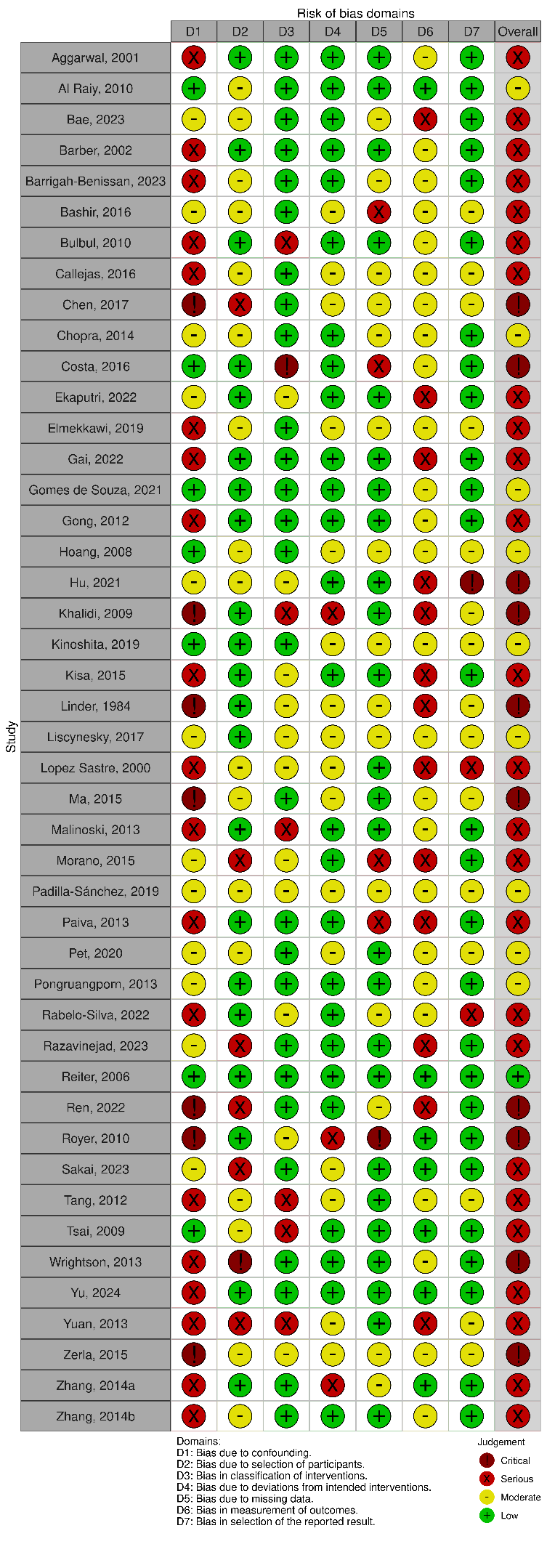


Figure F2. Risk of Bias Ratings for observational studies (Robins I tool

Figure F3. Risk of Bias Ratings for before-after studies


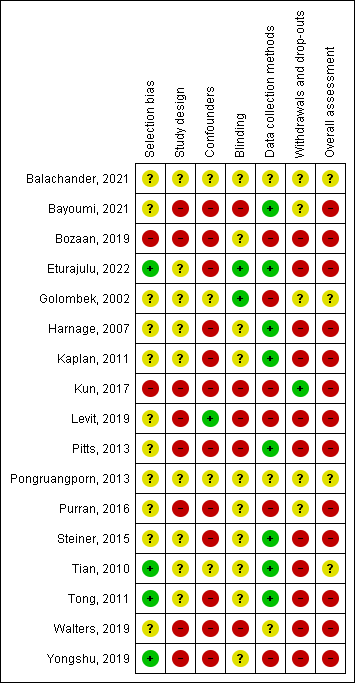


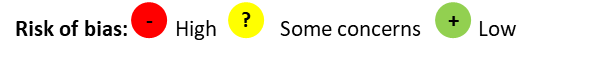


# Supplement G- Complete list of references for Table 2

Garland JS, Alex CP, Uhing MR, Peterside IE, Rentz A, Harris MC. Pilot trial to compare tolerance of chlorhexidine gluconate to povidone-iodine antisepsis for central venous catheter placement in neonates. J Perinatol 2009; 29(12): 808-13.

Sakai H, Hirosue M, Iwata M, Terasawa T. The effect of introducing a nurse-practitioner-led peripherally inserted central venous catheter placement program on the utilization of central venous access device: A retrospective study in Japan. J 2023.

Zhang J, Tang S, He L, et al. [Effect of standardized PICC training and management on the clinical effect and complication of catheterization]. Zhong Nan Da Xue Xue Bao Yi Xue Ban 2014; 39(6): 638-43.

Walters B, Price C. Quality Improvement Initiative Reduces the Occurrence of Complications in Peripherally Inserted Central Catheters. J Infus Nurs 2019; 42(1): 29-36.

Kaufman DA, Blackman A, Conaway MR, Sinkin RA. Nonsterile glove use in addition to hand hygiene to prevent late-onset infection in preterm infants: randomized clinical trial. JAMA Pediatr 2014; 168(10): 909-16.

Li J, Fan YY, Xin MZ, et al. A randomised, controlled trial comparing the long-term effects of peripherally inserted central catheter placement in chemotherapy patients using B-mode ultrasound with modified Seldinger technique versus blind puncture. Eur J Oncol Nurs 2014; 18(1): 94-103.

Tan J, Liu L, Xie J, Hu L, Yang Q, Wang H. Cost-effectiveness analysis of ultrasound-guided Seldinger peripherally inserted central catheters (PICC). Springerplus 2016; 5(1): 2051.

Wang Q, Wang N, Sun Y. Clinical effect of peripherally inserted central catheters based on modified seldinger technique under guidance of vascular ultrasound. Pak J Med Sci 2016; 32(5): 1179-83.

Zhang L, Zhang X, Kang Y, Teng Y, Yang Y. Application of PICC Catheterization with Modified Seldinger Technique Under the Guidance of B-mode Ultrasound in Chemotherapy Patients. Anti-Tumor Pharmacy 2019; 9(5): 779-82.

Qi YZ, Guo Y, Xu XX, Zhang H, Li L. Comparison of placement of peripherally inserted central catheters using vascular ultrasound guidance system and traditional method in 938 tumor patients. Chinese Journal of Clinical Nutrition 2012; 20(4): 253-5.

Yin T, Huo Y, Zhao Y, Li W, Gao H. Retrospective Study of the Application Value Analysis of Ultrasound-Guided Technology in Peripheral Deep Venous Catheterization of Neonates. Dis Markers 2022; 2022: 1726906.

Pongruangporn M, Ajenjo MC, Russo AJ, et al. Patient- and device-specific risk factors for peripherally inserted central venous catheter-related bloodstream infections. Infect Control Hosp Epidemiol 2013; 34(2): 184-9.

Razavinejad SM, Saeed N, Pourarian S, et al. Complications and Related Risk Factors of Peripherally Inserted Central Catheters in Neonates: A Historical Cohort Study. Archives of Iranian Medicine 2023; 26(4): 218-25.

Malinoski D, Ewing T, Bhakta A, et al. Which central venous catheters have the highest rate of catheter-associated deep venous thrombosis: a prospective analysis of 2,128 catheter days in the surgical intensive care unit. J Trauma Acute Care Surg 2013; 74(2): 454-60; discussion 61-2.

Callejas A, Osiovich H, Ting JY. Use of peripherally inserted central catheters (PICC) via scalp veins in neonates. J Matern Fetal Neonatal Med 2016; 29(21): 3434-8.

Gai M, Wang Y, Chen J, et al. Effect of femoral PICC line insertion in neonates with digestive tract disease. Am J Transl Res 2022; 14(10): 7487-93.

Kinoshita D, Hada S, Fujita R, Matsunaga N, Sakaki H, Ohki Y. Maximal sterile barrier precautions independently contribute to decreased central line-associated bloodstream infection in very low birth weight infants: A prospective multicenter observational study. Am J Infect Control 2019; 47(11): 1365-9.

Padilla-Sanchez C, Montejano-Lozoya R, Benavent-Taengua L, et al. Risk factors associated with adverse events in neonates with peripherally inserted central catheter. Enferm Intensiva (Engl Ed) 2019; 30(4): 170-80.

Bashir RA, Swarnam K, Vayalthrikkovil S, Yee W, Soraisham AS. Association between Peripherally Inserted Central Venous Catheter Insertion Site and Complication Rates in Preterm Infants. Am J Perinatol 2016; 33(10): 945-50.

Hu Y, Ling Y, Ye Y, et al. Analysis of risk factors of PICC-related bloodstream infection in newborns: implications for nursing care. Eur J Med Res 2021; 26(1): 80.

Ekaputri DS, Sukmawati M, Sidiartha IGL, Nilawati GAP, Utama IMGDL, Gustawan IW. Peripherally Inserted Central Catheter Dwell Time as a Risk Factor of Central Line-Associated Bloodstream Infection in Neonates. Iranian Journal of Neonatology 2022; 13(2): 39-45.

Elmekkawi A, Maulidi H, Mak W, Aziz A, Lee KS. Outcomes of upper extremity versus lower extremity placed peripherally inserted central catheters in a medical-surgical neonatal intensive care unit1. J Neonatal Perinatal Med 2019; 12(1): 57-63.

Wrightson DD. Peripherally inserted central catheter complications in neonates with upper versus lower extremity insertion sites. Adv Neonatal Care 2013; 13(3): 198-204.

Tsai MH, Lien R, Wang JW, et al. Complication rates with central venous catheters inserted at femoral and non-femoral sites in very low birth weight infants. Pediatr Infect Dis J 2009; 28(11): 966-70.

López Sastre JB, Fernández Colomer B, Coto Cotallo GD, Ramos Aparicio A. Prospective evolution of percutaneous central venous silastic catherters in newborn infants. "castrillo" hospital group. An Esp Pediatr 2000; 53(2): 138-47.

Pet GC, Eickhoff JC, McNevin KE, Do J, McAdams RM. Risk factors for peripherally inserted central catheter complications in neonates. J Perinatol 2020; 40(4): 581-8.

Ma M, Garingo A, Jensen AR, Bliss D, Friedlich P. Complication risks associated with lower versus upper extremity peripherally inserted central venous catheters in neonates with gastroschisis. J Pediatr Surg 2015; 50(4): 556-8.

Aggarwal R, Downe L. Use of percutaneous silastic central venous catheters in the management of newborn infants. Indian Pediatr 2001; 38(8): 889-92.

Kisa P, Ting J, Callejas A, Osiovich H, Butterworth SA. Major thrombotic complications with lower limb PICCs in surgical neonates. J Pediatr Surg 2015; 50(5): 786-9.

Hoang V, Sills J, Chandler M, Busalani E, Clifton-Koeppel R, Modanlou HD. Percutaneously inserted central catheter for total parenteral nutrition in neonates: complications rates related to upper versus lower extremity insertion. Pediatrics 2008; 121(5): e1152-9.

Bulbul A, Okan F, Nuhoglu A. Percutaneously inserted central catheters in the newborns: a center's experience in Turkey. J Matern Fetal Neonatal Med 2010; 23(6): 529-35.

Ong CK, Venkatesh SK, Lau GB, Wang SC. Prospective randomized comparative evaluation of proximal valve polyurethane and distal valve silicone peripherally inserted central catheters. J Vasc Interv Radiol 2010; 21(8): 1191-6.

Linder LE, Curelaru I, Gustavsson B, Hansson HA, Stenqvist O, Wojciechowski J. Material thrombogenicity in central venous catheterization: a comparison between soft, antebrachial catheters of silicone elastomer and polyurethane. JPEN J Parenter Enteral Nutr 1984; 8(4): 399-406.

Gomes de Souza NM, Silveira Rocha R, Pinheiro Ferreira R, Bastos da Silveira Reis C, Souza Bandeira RS, Facanha Melo AP. Comparing the use of silicone and polyurethane Peripherally Inserted Central Catheters in newborns: A retrospective study. J Clin Nurs 2021; 30(23-24): 3439-47.

Chico-Padron RM, Carrion-Garcia L, Delle-Vedove-Rosales L, et al. Comparative safety and costs of transparent versus gauze wound dressings in intravenous catheterization. J Nurs Care Qual 2011; 26(4): 371-6.

Pitts S. Retrospective Analysis of a Pediatric Vascular Access Program and Clinical Outcomes. Journal of the Association for Vascular Access 2013; 18(2): 114-20.

Levit O, Shabanova V, Bizzarro M. Impact of a dedicated nursing team on central line-related complications in neonatal intensive care unit. J Matern Fetal Neonatal Med 2020; 33(15): 2618-22.

Yongshu LIU, Ting S, Lan S, et al. Evaluation of PICC team in prevention of peripherally inserted central catheter-related mechanical phlebitis among very-low-birth-weight premature infants. Nursing of Integrated Traditional Chinese & Western Medicine 2019; 5(6): 5-8.

Araujo OR, Araujo MC, Silva JS, Barros MM. Intermittent heparin is not effective at preventing the occlusion of peripherally inserted central venous catheters in preterm and term neonates. Rev 2011; 23(3): 335-40.

Zerla PA, Canelli A, Caravella G, et al. Open- vs Closed-Tip Valved Peripherally Inserted Central Catheters and Midlines: Findings from a Vascular Access Database. Journal of the Association for Vascular Access 2015; 20(3): 169-76.

Reiter PD, Novak K, Valuck RJ, Rosenberg AA, Fish D. Effect of a closed drug-delivery system on the incidence of nosocomial and catheter-related bloodstream infections in infants. Epidemiol Infect 2006; 134(2): 285-91.

Rundjan L, Rohsiswatmo R, Paramita TN, Oeswadi CA. Closed catheter access system implementation in reducing the bloodstream infection rate in low birth weight preterm infants. Front 2015; 3: 20.

Rejane Rabelo-Silva E, Lourenco SA, Maestri RN, et al. Patterns, appropriateness and outcomes of peripherally inserted central catheter use in Brazil: a multicentre study of 12 725 catheters. BMJ Qual Saf 2022; 31(9): 652-61.

Chopra V, Ratz D, Kuhn L, Lopus T, Chenoweth C, Krein S. PICC-associated bloodstream infections: prevalence, patterns, and predictors. Am J Med 2014; 127(4): 319-28.

Bae HS, Kim KY, Han YM. Comparison of complications between reverse-tapered and nontapered peripherally inserted central catheters. PLoS ONE 2023; 18(5): e0285445.

Barrigah-Benissan K, Ory J, Simon C, et al. Clinical factors associated with peripherally inserted central catheters (PICC) related bloodstream infections: a single centre retrospective cohort. Antimicrob 2023; 12(1): 5.

Liscynesky C, Johnston J, Haydocy KE, Stevenson KB. Prospective evaluation of peripherally inserted central catheter complications in both inpatient and outpatient settings. Am J Infect Control 2017; 45(9): 1046-9.

Tian G, Zhu Y, Qi L, Guo F, Xu H. Efficacy of multifaceted interventions in reducing complications of peripherally inserted central catheter in adult oncology patients. Support Care Cancer 2010; 18(10): 1293-8.

Liu XY, Shen YY, Xu XH, Tang XH. Application of evidence-based nursing in the prevention of postoperative complications with PICC insertion. Chinese Journal of Clinical Nutrition 2013; 21(5): 309-12.

He B, Zhang A, He S. Therapeutic Effect of Ultrasound-Guided Peripherally Inserted Central Catheter Combined with Predictive Nursing in Patients with Large-Area Severe Burns. Comput Math Methods Med 2022; 2022: 1019829.

Royer T. Implementing a better bundle to achieve and sustain a zero central line-associated bloodstream infection rate. J Infus Nurs 2010; 33(6): 398-406.

Golombek SG, Rohan AJ, Parvez B, Salice AL, LaGamma EF. "Proactive" management of percutaneously inserted central catheters results in decreased incidence of infection in the ELBW population. J Perinatol 2002; 22(3): 209-13.

Ren C, Dong F, Du Y, Wang J. Implications of PDCA management for central venous line placement in neonates. Revista de Psiquiatria Clinica 2022; 49(6): 124-7.

Bayoumi MAA, Van Rens MFP, Chandra P, et al. Effect of implementing an Epicutaneo-Caval Catheter team in Neonatal Intensive Care Unit. J 2021; 22(2): 243-53.

Costa P, Paiva ED, Kimura AF, Castro TEd. Fatores de risco para infecção de corrente sanguínea associada ao cateter central de inserção periférica em neonatos. Acta Paulista de Enfermagem 2016; 29(2): 161-8.

Paiva ED, Kimura AF, Costa P, Magalhães TEdC, Toma E, Alves AMA. Complications related to the type of epicutaneous catheter in a cohort of neonates. Online Brazilian Journal of Nursing 2013; 12(4): 942-52.

# Supplement H- Summary of findings tables

## Table H1: A catheter inserted only by an individual with catheter insertion training/certification compared to insertion by an individual with no requirement for formal training/certification ("routine practice") in participants requiring a PICC

| Outcome № of participants (studies) | Relative effect (95% CI) | **Anticipated absolute effects (95% CI)** | | | Certainty | What happens |
| --- | --- | --- | --- | --- | --- | --- |
|  |  | **Effect with catheter inserted by an individual with no requirement for formal training/certification** | **Effect with catheter inserted by an individual with catheter insertion training/certification** | **Difference** |  |  |
| **CABSI/CRBSI** in adults № of participants: 1324 (2 cohort studies)[10, 12] | **aHR 0.82** (0.44 to 1.54)^a^ | 7.8% | **6.5%** (3.5 to 11.8) | **1.4% fewer** (4.3 fewer to 4 more)^a^ | ⨁◯◯◯ VERY LOW^b,c^ | Formal catheter insertion training may reduce the risk of CABSI/CRBSI in adults compared to no formal training; but the evidence is very uncertain. |
| **CABSI/CRBSI** in children and adolescents № of participants: (0 studies) | No evidence | | | | - |  |
| **CABSI/CRBSI** in neonates № of participants: 1631 (1 before–after study)[11] | CABSI/CRBSI/1000 central line days: Before training: 9.11 ± 8.9; after training: 18.34 ± 27.31 p= 0.412 (overall sample size 1631, unclear how many neonates had PICC). | | | | ⨁◯◯◯ VERY LOW^b,d^ | The evidence is very uncertain about the effect of formal catheter insertion training compared to no formal training on CABSI/CRBSI in neonates. |
| **Bloodstream infection–related mortality in** adults, children and adolescents, neonates № of participants: (0 studies) | No evidence | | | | - |  |
| **Sepsis** in adults, children and adolescents, neonates № of participants: (0 studies) | No evidence | | | | - |  |
| **Local infection** in adults, children and adolescents, neonates № of participants: (0 studies) | No evidence | | | | - |  |
| **All-cause mortality** in adults, children and adolescents, neonates № of participants: (0 studies) | No evidence | | | | - |  |
| **Phlebitis/thrombophlebitis** in adults № of participants: 610 (1 cohort study)[12] | **RR 0.24** (0.09 to 0.64)^e,f^ | 6.7% | **1.6%** (0.6 to 4.3) | **5.1% fewer** (6.1 fewer to 2.4 fewer)^e,f^ | ⨁◯◯◯ VERY LOW^b,c^ | Formal catheter insertion training may reduce phlebitis in adults compared to no formal training, but the evidence is very uncertain. |
| **Phlebitis/thrombophlebitis** in children and adolescents, neonates № of participants: (0 studies) | No evidence | | | | - |  |
| **Complications related to intravascular catheter insertion** in adults № of participants: 2230  (1 cohort study)[10] | **RR 0.28** (0.17 to 0.48)^e^ | 5.1% | **1.4%** (0.9 to 2.4) | **3.7% fewer** (4.2 fewer to 2.7 fewer)^e^ | ⨁◯◯◯ VERY LOW^b,c,g^ | The evidence is very uncertain about the effect of formal catheter insertion training compared to no formal training on complications related to insertion in adults. |
| **Complications related to intravascular catheter insertion** in children and adolescents, neonates № of participants: (0 studies) | No evidence | | | | - |  |
| ***The risk in the intervention group** (and its 95% CI) is based on the assumed risk in the comparison group and the **relative effect** of the intervention (and its 95% CI). **CABSI:** catheter-associated bloodstream infection; **CI:** confidence interval; **CRBSI:** catheter-related bloodstream infection; **№:** number; **PICC:** peripherally inserted central catheter; **RR:** risk ratio. | | | | | | |
| **GRADE Working Group grades of evidence** **High certainty:** we are very confident that the true effect lies close to that of the estimate of the effect. **Moderate certainty:** we are moderately confident in the effect estimate: the true effect is likely to be close to the estimate of the effect, but there is a possibility that it is substantially different. **Low certainty:** our confidence in the effect estimate is limited: the true effect may be substantially different from the estimate of the effect. **Very low certainty:** we have very little confidence in the effect estimate: the true effect is likely to be substantially different from the estimate of effect. | | | | | | |

**Explanations**

a. Results based on a cohort study by Sakai et al. 2023; another cohort study by Zhang et al. 2014 reported fewer events with intervention 0/310 (0%) vs. 8/300 (3%). Two additional before–after studies rated as high risk of bias reported similar results between groups: Bozaan et al. 2019: 2/93 (2%) vs. 7/133 (5%); Walters et al. 2019 (numbers reflect catheters): 0/1082 (0%) vs. 1/194 (0.5%).

b. We used the Risk of Bias In Non-randomized Studies – Of Interventions (ROBINS-I) to rate the risk of bias of the non-randomized studies. All studies have bias due to possible residual confounding and bias due to the participant selection. We downgraded 2 steps. No further downgrading was considered necessary.

c. Very few events; downgraded 1 step for imprecision.

d. Unclear if outcome is in PICC only or also in central venous catheters; downgraded 1 step for indirectness.

e. Self-calculated

f. Results based on cohort study by Zhang et al. 2014; an additional before-after study with high risk of bias reported lower proportions of phlebitis with training: 61/1916 (3%) vs. 96/1974 (5%)

g. Two additional before-after studies rated as high risk of bias reported similar results: Kun et al. 2017 12/80 (15%) vs. 29/80 (36%) and Purran et al. 2016 11/228 (5%) vs. 14/67 (21%); another before-after study by Bozaan et al. 2019 reported no statistically significant difference between study groups (14/93 (15%) vs. 19/133 (14%))

## Table H2: A catheter inserted by an individual wearing gloves (either sterile or non-sterile) compared to insertion by an individual not specifically required to wear gloves in participants requiring a PICC

| Outcome № of participants (studies) | Relative effect (95% CI) | **Anticipated absolute effects (95% CI)** | | | Certainty | What happens |
| --- | --- | --- | --- | --- | --- | --- |
|  |  | **Effect with catheter inserted by an individual not specifically required to wear gloves** | **Effect with catheter inserted by an individual wearing gloves** | **Difference** |  |  |
| **CABSI/CRBSI** in adults № of participants: (0 observational studies) | No evidence | | | | - |  |
| **CABSI/CRBSI** in children and adolescents № of participants: (0 studies) | No evidence | | | | - |  |
| **CABSI/CRBSI** in neonates № of participants: 120 (1 RCT)[13] | **RR 1.00** (0.26 to 3.81)^a^ | 6.7% | **6.7%** (1.7 to 25.4) | **0.0% fewer** (4.9 fewer to 18.7 more)^a^ | ⨁◯◯◯ VERY LOW^b^ | The evidence is very uncertain about the effect of wearing gloves compared to not wearing gloves on CABSI/CRBSI in neonates. |
| **Bloodstream infection–related mortality** in adults, children and adolescents, neonates № of participants: (0 studies) | No evidence | | | | - |  |
| **Sepsis** in adults, children and adolescents, neonates № of participants: (0 studies) | No evidence | | | | - |  |
| **Local infection** in adults, children and adolescents, neonates № of participants: (0 studies) | No evidence | | | | - |  |
| **All-cause mortality** in adults № of participants: (0 studies) | No evidence | | | | - |  |
| **All-cause mortality** in children and adolescents № of participants: (0 studies) | No evidence | | | | - |  |
| **All-cause mortality** in neonates № of participants: 120 (1 RCT)[13] | **RR 3.00** (0.63 to 14.27)^a^ | 3.3% | **10.0%** (2.1 to 47.6) | **6.7% more** (1.2 fewer to 44.2 more)^a^ | ⨁◯◯◯ VERY LOW^b^ | The evidence is very uncertain about the effect of wearing gloves compared to not wearing gloves on all-cause mortality in neonates. |
| **Phlebitis/thrombophlebitis** in adults, children and adolescents, neonates № of participants: (0 studies) | No evidence | | | | - |  |
| **Complications related to intravascular catheter insertion** in adults, children and adolescents, neonates № of participants: (0 studies) | No evidence | | | | - |  |
| ***The risk in the intervention group** (and its 95% CI) is based on the assumed risk in the comparison group and the **relative effect** of the intervention (and its 95% CI). **CABSI:** catheter-associated bloodstream infection; **CI:** confidence interval; **CRBSI:** catheter-related bloodstream infection; **№:** number; **PICC:** peripherally inserted central catheter; **RCT:** randomized controlled trial; **RR:** risk ratio. | | | | | | |
| **GRADE Working Group grades of evidence** **High certainty:** we are very confident that the true effect lies close to that of the estimate of the effect. **Moderate certainty:** we are moderately confident in the effect estimate: the true effect is likely to be close to the estimate of the effect, but there is a possibility that it is substantially different. **Low certainty:** our confidence in the effect estimate is limited: the true effect may be substantially different from the estimate of the effect. **Very low certainty:** we have very little confidence in the effect estimate: the true effect is likely to be substantially different from the estimate of effect. | | | | | | |

**Explanations**

a. Self-calculated.

b. Extremely few events; downgraded 3 steps for imprecision.

## Table H3: Catheter insertion with ultrasound-guided assistance compared to insertion without ultrasound-guided assistance in participants requiring a PICC

| Outcome № of participants (studies) | Relative effect (95% CI) | **Anticipated absolute effects (95% CI)** | | | Certainty | What happens |
| --- | --- | --- | --- | --- | --- | --- |
|  |  | **Effect without ultrasound-guided insertion** | **Effect with ultrasound-guided insertion** | **Difference** |  |  |
| **CABSI/CRBSI** in adults № of participants: 98 (1 RCT)[17] | **RR 0.32** (0.01 to 7.67)^a,b^ | 2.1% | **0.0%** (0 to 0) | **2.1% fewer** (6 fewer to 2 more)^a,b^ | ⨁◯◯◯ VERY LOW^c^ | The evidence is very uncertain about the effect of US-guided insertion compared to non-US-guided insertion on CABSI/CRBSI in adults. |
| **CABSI/CRBSI** in children and adolescents, neonates № of participants: (0 studies) | No evidence | | | | - |  |
| **Bloodstream infection–related mortality** in adults, children and adolescents, neonates № of participants: (0 studies) | No evidence | | | | - |  |
| **Sepsis** in adults, children and adolescents, neonates № of participants: (0 studies) | No evidence | | | | - |  |
| **Local infection** in adults № of participants: 319 (1 RCT)[19] | **RR 0.17** (0.04 to 0.73)^a,d^ | 7.4% | **1.3%** (0.3 to 5.4) | **6.2% fewer** (7.1 fewer to 2 fewer)^a,d^ | ⨁◯◯◯ VERY LOW^e,f^ | US-guided insertion compared to non-US-guided insertion on may reduce local infection in adults but the evidence is very uncertain. |
| **Local infection** in children and adolescents, neonates № of participants: (0 studies) | No evidence | | | | - |  |
| **All-cause mortality** in adults № of participants: 98 (1 RCT)[17] | **RR 0.32** (0.01 to 7.67)^a,g^ | 2.1% | **0.0%** (0 to 0) | **2.1% fewer** (6 fewer to 2 more)^a,g^ | ⨁◯◯◯ VERY LOW^c^ | The evidence is very uncertain about the effect of US-guided insertion compared to non-US-guided insertion on all-cause mortality in adults. |
| **All-cause mortality** in children and adolescents, neonates № of participants: (0 studies) | No evidence | | | | - |  |
| **Phlebitis/thrombophlebitis** in adults № of participants: 1744 (5 RCTs) [17-19, 21, 24] | **RR 0.17** (0.08 to 0.50)^h^ | 5.7% | **1.1%** (0.5 to 2.9) | **4.6% fewer** (5.2 fewer to 2.9 fewer)^f^ | ⨁⨁◯◯  LOW^i,j^ | US-guided insertion may reduce phlebitis/thrombophlebitis in adults compared to non-US-guided insertion. |
| **Phlebitis/thrombophlebitis** in children and adolescents № of participants: (0 studies) | No evidence | | | | - |  |
| **Phlebitis/thrombophlebitis** in neonates № of participants: 94 (1 RCT)[22] | **RR 0.20** (0.01 to 4.05)^a^ | 4.3% | **0.9%** (0 to 17.2) | **3.4% fewer** (4.2 fewer to 13 more)^a^ | ⨁◯◯◯ VERY LOW^c^ | The evidence is very uncertain about the effect of US-guided insertion compared to non-US-guided insertion on phlebitis/thrombophlebitis in neonates. |
| ***The risk in the intervention group** (and its 95% CI) is based on the assumed risk in the comparison group and the **relative effect** of the intervention (and its 95% CI). **CABSI:** catheter-associated bloodstream infection; **CI:** confidence interval; **CRBSI:** catheter-related bloodstream infection; **№:** number; **PICC:** periferally inserted central catheter; **RCT:** randomized controlled trial; **RR:** risk ratio; **US:** ultrasound. | | | | | | |
| **GRADE Working Group grades of evidence** **High certainty:** we are very confident that the true effect lies close to that of the estimate of the effect. **Moderate certainty:** we are moderately confident in the effect estimate: the true effect is likely to be close to the estimate of the effect, but there is a possibility that it is substantially different. **Low certainty:** our confidence in the effect estimate is limited: the true effect may be substantially different from the estimate of the effect. **Very low certainty:** we have very little confidence in the effect estimate: the true effect is likely to be substantially different from the estimate of effect. | | | | | | |

**Explanations**

a. Self-calculated.

b. A retrospective cohort study by Chen et al. 2017 rated as high risk of bias reported bacteriemia in 8/36 (22%) of the US-guided and 1/7 (14%) of the non-US-guided insertion group (p=1.00). c. Extremely few events; downgraded 3 steps for imprecision. d. A cohort study by Gong et al. with some concerns for risk of bias reported 1/85 (1%) of local infection in the US-guided insertion group and 3/90 (3%) in the non US-guided insertion group. Another cohort study by Yuan et al. with some concerns for risk of bias reported 5/391 (1%) of local infection in the US-guided insertion group and 11/ 206 (5%) in the non-US-guided insertion group. e. High risk of bias; downgraded 1 step for risk of bias.

f. very few events; downgraded 2 steps for imprecision.

g. A prospective cohort study by Barber et al. 2002 rated as some concerns reported all-cause mortality in 7/107 (7%) US-guided inserted catheters and 1/7 (3%) catheters inserted without US guidance.

h. Meta-analysis. i. Study contributing most to Meta-analysis has a high risk of bias; downgraded 1 step for risk of bias.

j. Very few events but CI indicating risk reduction; downgraded 1 step for imprecision.

## Table H4: Catheter insertion in distal section of the upper limb compared to insertion in the proximal section of the upper limb in participants requiring a PICC

| Outcome № of participants (studies) | Relative effect (95% CI) | **Anticipated absolute effects (95% CI)** | | | Certainty | What happens |
| --- | --- | --- | --- | --- | --- | --- |
|  |  | **Effect with catheter inserted in the proximal section of the upper limb** | **Effect with catheter inserted in the distal section of the upper limb** | **Difference** |  |  |
| **CABSI/CRBSI** in adults № of participants: 162 cases 485 controls (1 case-control study)[25] | Catheter insertion in distal section of the upper limb decreased the risk of CABSI/CRBSI with 23% but the CI includes both an increase and a decrease of effect (OR (95%CI): 0.77 (0.47 to 1.20)). | | | | ⨁◯◯◯ VERY LOW ^a,b,c^ | The evidence is very uncertain about the effect of catheter insertion in the distal section of the upper limb compared to the proximal section of the upper limb on CABSI/CRBSI in adults. |
| **CABSI/CRBSI** in neonates № of participants: 2500 (1 cohort study)[26] | Catheter insertion in distal section of the upper limb increased the risk of CABSI/CRBSI with 36% but the CI includes both an increase and a decrease of effect (OR (95%CI): 1.36 (0.78 to 2.35)). | | | | ⨁◯◯◯ VERY LOW ^a,e^ | Catheter insertion in distal section of the upper limb may increase CABSI/CRBSI in neonates compared to the proximal section of the upper limb, but the evidence is very uncertain. |
| **CABSI/CRBSI** in children and adolescents, neonates № of participants: (0 studies) | No evidence | | | | - |  |
| **Bloodstream infection–related mortality** in adults, children and adolescents, neonates № of participants: (0 studies) | No evidence | | | | - |  |
| **Sepsis** in adults, children and adolescents, neonates № of participants: (0 studies) | No evidence | | | | - |  |
| **Local infection** in adults, children and adolescents, neonates № of participants: (0 studies) | No evidence | | | | - |  |
| **All-cause mortality** in adults, children and adolescents, neonates № of participants: (0 studies) | No evidence | | | | - |  |
| **Phlebitis/thrombophlebitis** in adults, children and adolescents, neonates № of participants: (0 studies) | No evidence | | | | - |  |
| ***The risk in the intervention group** (and its 95% CI) is based on the assumed risk in the comparison group and the **relative effect** of the intervention (and its 95% CI). **CABSI:** catheter-associated bloodstream infection; **CI:** confidence interval; **CRBSI:** catheter-related bloodstream infection; **№:** number; **OR:** odds ratio; **PICC:** peripherally inserted central catheter. | | | | | | |
| **GRADE Working Group grades of evidence** **High certainty:** we are very confident that the true effect lies close to that of the estimate of the effect. **Moderate certainty:** we are moderately confident in the effect estimate: the true effect is likely to be close to the estimate of the effect, but there is a possibility that it is substantially different. **Low certainty:** our confidence in the effect estimate is limited: the true effect may be substantially different from the estimate of the effect. **Very low certainty:** we have very little confidence in the effect estimate: the true effect is likely to be substantially different from the estimate of effect. | | | | | | |

**Explanations**

a. We used the Risk Of Bias In Non-randomized Studies – Of Interventions (ROBINS-I) to rate the risk of bias of the non-randomized studies. All studies have bias due to possible residual confounding and bias due to the participant. We downgraded 2 steps. No further downgrading was considered necessary.

b. We defined *upper arm* as cubital fossa and above. However, in this study, the cubital fossa was combined with the forearm as the lower arm.

c. Wide CI including increase and reduction; downgraded 1 step for imprecision.

d. Self calculated

e. Number of events per group not reported; downgraded 1 step for imprecision.

## Table H5: Catheter insertion in the upper limb compared to insertion in the lower limb in participants requiring a PICC

| Outcome № of participants (studies) | Relative effect (95% CI) | **Anticipated absolute effects (95% CI)** | | | Certainty | What happens |
| --- | --- | --- | --- | --- | --- | --- |
|  |  | **Effect with catheter inserted in the lower limb** | **Effect with catheter inserted in the upper limb** | **Difference** |  |  |
| **CABSI/CRBSI** in adults № of participants: (0 studies) | No evidence | | | | - |  |
| **CABSI/CRBSI** in children and adolescents № of participants: 620 (1 cohort study)[30] | **RR 2.21** (1.08 to 4.54 )^a,b^ | 5.4% | **11.9%** (5.8 to 24.4) | **6.5% more** (0.4 more to 19 more) ^a,b^ | ⨁◯◯◯ VERY LOW^c,d,e^ | Catheter inserted in the upper limb may increase CABSI/CRBSI compared to catheter inserted in the lower limb in children and adolescents, but the evidence is very uncertain. |
| **CABSI/CRBSI** in neonates № of participants: 1355 (6 cohort studies)[5, 28, 31, 33, 35, 40] | CABSI ranged from 5%–10% in the upper limb group and from 0%–25% in the lower limb group.^b^ | | | | ⨁◯◯◯ VERY LOW^c,f^ | The evidence is very uncertain about the effect of catheter inserted in the upper limb compared to catheter inserted in the lower limb on CABSI/CRBSI in neonates. |
| **Bloodstream infection–related mortality** in adults, children and adolescents, neonates № of participants: (0 studies) | No evidence | | | | - |  |
| **Sepsis** in adults, children and adolescents № of participants: (0 studies) | No evidence | | | | - |  |
| **Sepsis** in neonates № of participants: 2417 (4 cohort studies)[32, 37, 42, 43] | Sepsis ranged from 5%–12% in the upper limb group and from 2%–23% in the lower limb group.^b^ | | | | ⨁◯◯◯ VERY LOW^c,g,h^ | The evidence is very uncertain about the effect of catheter inserted in the upper limb compared to catheter inserted in the lower limb on sepsis in neonates. |
| **Local infection** in adults, children and adolescents № of participants: (0 studies) | No evidence | | | | - |  |
| **Local infection** in neonates № of participants: 1388 (3 cohort studies)[27, 38, 41] | Local infection in neonates ranged from 0%–5% in the upper limb group and from 0%–43% in the lower limb group.^b^ | | | | ⨁◯◯◯ VERY LOW^c,e^ | The evidence is very uncertain about the effect of catheter inserted in the upper limb compared to catheter inserted in the lower limb on local infection in neonates. |
| **All-cause mortality** in adults № of participants: (0 studies) | No evidence | | | | - |  |
| **All-cause mortality** in children and adolescents № of participants: 620 (1 cohort study)[30] | **RR 0.79** (0.47 to 1.32)^a,b^ | 12.1% | **9.5%** (5.7 to 15.9) | **2.5% fewer** (6.4 fewer to 3.9 more)^a,b^ | ⨁◯◯◯ VERY LOW^c,e,i^ | The evidence is very uncertain about the effect of catheter inserted in the upper limb compared to catheter inserted in the lower limb on all-cause mortality in children. |
| **All-cause mortality** in neonates № of participants: 1172 (3 cohort studies)[32, 42, 43] | All-cause mortality ranged from 5%–6% in the upper limb group and from 1%–6% in the lower limb group.^b^ | | | | ⨁◯◯◯ VERY LOW^c,e^ | The evidence is very uncertain about the effect of catheter inserted in the upper limb compared to catheter inserted in the lower limb on all-cause mortality in neonates. |
| **Phlebitis/thrombophlebitis** in adults № of participants: 124 (1 cohort study)[39] | **RR 1.42** (0.57 to 3.52)^a^ | 14.3% | **20.3%** (8.1 to 50.3) | **6.0% more** (6.1 fewer to 36 more)^a^ | ⨁◯◯◯ VERY LOW^c,e^ | The evidence is very uncertain about the effect of catheter inserted in the upper limb compared to catheter inserted in the lower limb on phlebitis in adults. |
| **Phlebitis/thrombophlebitis** in children and adolescents № of participants: 620 (1 cohort study)[30] | **RR 1.27** (0.14 to 11.23)^a,b^ | 0.7% | **0.9%** (0.1 to 7.5) | **0.2% more** (0.6 fewer to 6.9 more)^a,b^ | ⨁◯◯◯ VERY LOW^c,d,j^ | The evidence is very uncertain about the effect of catheter inserted in the upper limb compared to catheter inserted in the lower limb on phlebitis/thrombophlebitis in children and adolescents. |
| **Phlebitis/thrombophlebitis** in neonates № of participants: 5837 (11 cohort studies)[28, 32-34, 36-38, 40-43] | Phlebitis/thrombophlebitis ranged from 0%–10% in the upper limb group and from 0%–21% in the lower limb group.^b^ | | | | ⨁◯◯◯ VERY LOW^c,k^ | The evidence is very uncertain about the effect of catheter inserted in the upper limb compared to catheter inserted in the lower limb on phlebitis/thrombophlebitis in neonates. |
| **Complications related to insertion** in adults № of participants: (0 studies) | No evidence | | | | - |  |
| **Complications related to insertion** in children and adolescents № of participants: 620 (1 cohort study)[30] | **RR 1.44** (0.81 to 2.54 )^a,b^ | 8.7% | **12.6%** (7.1 to 22.2) | **3.8% more** (1.7 fewer to 13.4 more)^a,b^ | ⨁◯◯◯ VERY LOW^c,d,e^ | The evidence is very uncertain about the effect of catheter inserted in the upper limb compared to catheter inserted in the lower limb on complications related to insertion in children and adolescents. |
| **Complications related to insertion** in neonates № of participants: 2325 (5 cohort studies)[27-29, 38, 41] | Overall complications ranged from 3%–40% in the upper limb group and from 20%–30% in the lower limb group.^b^ | | | | ⨁◯◯◯ VERY LOW^c,l^ | The evidence is very uncertain about the effect of catheter inserted in the upper limb compared to catheter inserted in the lower limb on complications related to insertion in neonates. |
| ***The risk in the intervention group** (and its 95% CI) is based on the assumed risk in the comparison group and the **relative effect** of the intervention (and its 95% CI). **CABSI:** catheter-associated bloodstream infection; **CI:** confidence interval; **CRBSI:** catheter-related bloodstream infection; **№:** number; **PICC:** peripherally inserted central catheter; **RR:** risk ratio | | | | | | |
| **GRADE Working Group grades of evidence** **High certainty:** we are very confident that the true effect lies close to that of the estimate of the effect. **Moderate certainty:** we are moderately confident in the effect estimate: the true effect is likely to be close to the estimate of the effect, but there is a possibility that it is substantially different. **Low certainty:** our confidence in the effect estimate is limited: the true effect may be substantially different from the estimate of the effect. **Very low certainty:** we have very little confidence in the effect estimate: the true effect is likely to be substantially different from the estimate of effect. | | | | | | |

**Explanations**

a. Self-calculated.

b. Numbers reflect catheters. One study by Ekaputri et al rated as some concerns reported only the effect estimate (no raw data reported).

c. We used the Risk of Bias In Non-randomized Studies – of Interventions (ROBINS-I) to rate the risk of bias of the non-randomized studies. All studies have bias due to possible residual confounding and bias due to the participant selection. We downgraded 2 steps. No further downgrading was considered necessary.

d. Mixed population of neonates and children; downgraded 1 step for indirectness.

e. Very few events; downgraded 2 steps for imprecision.

f. One study showed a decreased risk of CABSI with insertion in the lower limb, while others showed similar results in the upper and lower limb groups; downgraded 1 step for inconsistency.

g. High risk of bias; downgraded 1 step for risk of bias.

h. One study showed a statistically significant increased risk of sepsis with insertion in the lower limb, while others showed similar proportions in the upper and lower limb groups; downgraded 1 step for inconsistency.

i. Outcome defined as "death or transferred to other NICUs"; mixed population of neonates and children; downgraded 2 steps for indirectness.

j. Extremely few events, very wide CI; downgraded 3 steps for imprecision.

k. Some studies showed increased phlebitis/thrombophlebitis in the upper limb group, others in the lower limb group, and other studies showed similar results in both groups; downgraded 1 step for inconsistency.

l. One study showed an increase of complications in the upper limb group and one study in the lower limb group, and two showed similar results in both groups; downgraded 1 step for inconsistency.

## Table H6: Catheter made of silicone material compared to catheter made of non-silicone material in participants requiring a PICC

| Outcome № of participants (studies) | Relative effect (95% CI) | **Anticipated absolute effects (95% CI)** | | | Certainty | What happens |
| --- | --- | --- | --- | --- | --- | --- |
|  |  | **Effect with catheter made of non-silicone material** | **Effect with catheter made of silicone material** | **Difference** |  |  |
| **CABSI/CRBSI** in adults № of participants: 392 (1 RCT)[46] | **RR 2.55** (0.50 to 12.99)^a,b^ | 1.0% | **2.6%** (0.5 to 13.1) | **1.6% more** (0.5 fewer to 12.1 more)^a,b^ | ⨁◯◯◯ VERY LOW^c^ | The evidence is very uncertain about the effect of catheters made of silicone material compared to catheters made of non-silicone material on CABSI/CRBSI in adults. |
| **CABSI/CRBSI** in children and adolescents, neonates № of participants: (0 studies) | No evidence | | | | - |  |
| **Bloodstream infection–related mortality** in adults, children and adolescents, neonates № of participants: (0 studies) | No evidence | | | | - |  |
| **Sepsis** in adults, children and adolescents, neonates № of participants: (0 studies) | No evidence | | | | - |  |
| **Local infection** in adults № of participants: 392 (1 RCT)[46] | **RR 2.04** (0.38 to 11.02)^a,b^ | 1.0% | **2.1%** (0.4 to 11.1) | **1.1% more** (0.6 fewer to 10.1 more)^a,b^ | ⨁◯◯◯ VERY LOW^c^ | The evidence is very uncertain about the effect of catheters made of silicone material compared to catheters made of non-silicone material on local infection in adults. |
| **Local infection** in children and adolescents, neonates № of participants: (0 studies) | No evidence | | | | - |  |
| **All-cause mortality** in adults, children and adolescents, neonates № of participants: (0 studies) | No evidence | | | | - |  |
| **Phlebitis/thrombophlebitis** in adults № of participants: 392 (1 RCT)[46] | **RR 2.00** (1.26 to 3.17)^a,b,d^ | 11.6% | **23.2%** (14.6 to 36.8) | **11.6% more** (3 more to 25.2 more)^a,b,d^ | ⨁⨁◯◯ LOW^e^ | Catheters made of silicone material may slightly increase phlebitis/thrombophlebitis in adults compared to polyurethane. |
| **Phlebitis/thrombophlebitis** in children and adolescents № of participants: 61 (1 cohort study)[44] | **RR 1.97** (0.74 to 5.26)^a^ | 18.2% | **35.8%** (13.5 to 95.6) | **17.6% more** (4.7 fewer to 77.5 more)^a^ | ⨁◯◯◯ VERY LOW^e,f,g^ | The evidence is very uncertain about the effect of catheters made of silicone material compared to catheters made of non-silicone material on phlebitis/thrombophlebitis in children and adolescents. |
| **Phlebitis/thrombophlebitis** in neonates № of participants: 450 (1 cohort study)[45] | **RR 0.20** (0.02 to 1.67)^a^ | 2.4% | **0.5%** (0 to 4.1) | **1.9% fewer** (2.4 fewer to 1.6 more)^a^ | ⨁◯◯◯ VERY LOW^c,h^ | The evidence is very uncertain about the effect of catheters made of silicone material compared to catheters made of non-silicone material on phlebitis/thrombophlebitis in neonates. |
| ***The risk in the intervention group** (and its 95% CI) is based on the assumed risk in the comparison group and the **relative effect** of the intervention (and its 95% CI). **CABSI:** catheter-associated bloodstream infection; **CI:** confidence interval; **CRBSI:** catheter-related bloodstream infection; **№:** number; **PICC:** peripherally inserted central catheter; **RCT:** randomized controlled trial; **RR:** risk ratio | | | | | | |
| **GRADE Working Group grades of evidence** **High certainty:** we are very confident that the true effect lies close to that of the estimate of the effect. **Moderate certainty:** we are moderately confident in the effect estimate: the true effect is likely to be close to the estimate of the effect, but there is a possibility that it is substantially different. **Low certainty:** our confidence in the effect estimate is limited: the true effect may be substantially different from the estimate of the effect. **Very low certainty:** we have very little confidence in the effect estimate: the true effect is likely to be substantially different from the estimate of effect. | | | | | | |

**Explanations**

a. Self-calculated.

b. Numbers reflect catheters.

c. Extremely few events, very wide CI; downgraded 3 steps for imprecision.

d. A cohort study by Curelaru et al. with high risk of bias reported 14/39 (36%) phlebitis/thrombophlebitis with silicone catheters and 4/22 (18%) with non-silicone catheters (study population adults and adolescents).

e. Very few events; downgraded 2 steps for imprecision.

f. We used the Risk Of Bias In Non-randomized Studies – Of Interventions (ROBINS-I) to rate the risk of bias of the non-randomized studies. All studies have bias due to possible residual confounding and bias due to the participant selection. We downgraded 2 steps. We further downgraded 1 step because of additional risk of bias.

g. Results are not presented separately for adults and adolescents; downgraded 1 step for indirectness.

h. We used ROBINS-I to rate the risk of bias of the non-randomized studies. All studies have bias due to possible residual confounding and bias due to the participant selection. We downgraded 2 steps. No further downgrading was considered necessary.

.

## Table H7: Catheter secured with an occlusive dressing compared to catheter secured with non-occlusive dressing in participants requiring a PICC

| Outcome № of participants (studies) | Relative effect (95% CI) | **Anticipated absolute effects (95% CI)** | | | Certainty | What happens |
| --- | --- | --- | --- | --- | --- | --- |
|  |  | **Effect with non-occlusive dressing** | **Effect with occlusive dressing** | **Difference** |  |  |
| **CABSI/CRBSI** in adults, children and adolescents, neonates № of participants: (0 studies) | No evidence | | | | - |  |
| **Bloodstream infection–related mortality** in adults, children and adolescents, neonates № of participants: (0 studies) | No evidence | | | | - |  |
| **Sepsis** in adults, children and adolescents, neonates № of participants: (0 studies) | No evidence | | | | - |  |
| **Local infection** in adults № of participants: (0 studies) | No evidence | | | | - |  |
| **Local infection** in children and adolescents, neonates № of participants: (0 studies) | No evidence | | | | - |  |
| **All-cause mortality** in adults, children and adolescents, neonates № of participants: (0 studies) | No evidence | | | | - |  |
| **Phlebitis/thrombophlebitis** in adults № of participants: 25 (1 RCT)[47] | **RR 2.17** (0.22 to 20.94)^a^ | 7.7% | **16.7%** (1.7 to 100) | **9.0% fewer** (6 fewer to 153.4 more)^a^ | ⨁◯◯◯ VERY LOW^b^ | The evidence is very uncertain about the effect of occlusive compared to non-occlusive dressing on phlebitis/thrombophlebitis in adults. |
| **Phlebitis/thrombophlebitis** in children and adolescents, neonates № of participants: (0 studies) | No evidence | | | | - |  |
| ***The risk in the intervention group** (and its 95% CI) is based on the assumed risk in the comparison group and the **relative effect** of the intervention (and its 95% CI). **CABSI:** catheter-associated bloodstream infection; **CI:** confidence interval; **CRBSI:** catheter-related bloodstream infection; **№:** number; **PICC:** peripherally inserted central catheter; **RCT:** randomized controlled trial; **RR:** risk ratio | | | | | | |
| **GRADE Working Group grades of evidence** **High certainty:** we are very confident that the true effect lies close to that of the estimate of the effect. **Moderate certainty:** we are moderately confident in the effect estimate: the true effect is likely to be close to the estimate of the effect, but there is a possibility that it is substantially different. **Low certainty:** our confidence in the effect estimate is limited: the true effect may be substantially different from the estimate of the effect. **Very low certainty:** we have very little confidence in the effect estimate: the true effect is likely to be substantially different from the estimate of effect. | | | | | | |

**Explanations**

a. Self-calculated.

b. Extremely few events, very wide CI; downgraded 3 steps for imprecision.

## Table H8: Catheter inserted by an insertion team compared to catheter inserted by an individual not part of a specific insertion team in participants requiring a PICC

| Outcome № of participants (studies) | Relative effect (95% CI) | **Anticipated absolute effects (95% CI)** | | | Certainty | What happens |
| --- | --- | --- | --- | --- | --- | --- |
|  |  | **Effect with catheter inserted by an individual not part of a specific insertion team** | **Effect with catheter insertion by insertion teams** | **Difference** |  |  |
| **CABSI/CRBSI** in adults № of participants: (0 studies) | No evidence | | | | - |  |
| **CABSI/CRBSI** in children and adolescents № of participants: 669 (1 before–after study)[49] | Insertion team: 2.0/1000 catheter line days, no insertion team: 9.12/1000 catheter line days | | | | ⨁◯◯◯ VERY LOW^a^ | Catheter insertion by insertion teams may reduce the risk for CABSI/CRBSI in children and adolescents compared to no insertion teams, but the evidence is very uncertain. |
| **CABSI/CRBSI** in neonates № of participants: 731 (1 before–after study)[48] | Rate per 1000 central line days (95% CI):  Insertion team: 0.3 (0.8 to 3.1),  No insertion team: 1.6 (0.01 to 1.2)  Rate ratio (95% CI): 0.43 (0.08 to 2.34) | | | | ⨁◯◯◯ VERY LOW^a,b^ | The evidence is very uncertain about the effect of insertion teams compared to no insertion teams on CABSI/CRBSI in neonates. |
| **Bloodstream infection–related mortality** in adults, children and adolescents, neonates № of participants: (0 studies) | No evidence | | | | - |  |
| **Sepsis** in adults, children and adolescents, neonates № of participants: (0 studies) | No evidence | | | | - |  |
| **Local infection** in adults, children and adolescents, neonates № of participants: (0 studies) | No evidence | | | | - |  |
| **All-cause mortality** in adults, children and adolescents, neonates № of participants: (0 studies) | No evidence | | | | - |  |
| **Phlebitis/thrombophlebitis** in adults, children and adolescents № of participants: (0 studies) | No evidence | | | | - |  |
| **Phlebitis/thrombophlebitis** in neonates № of participants: 871 (2 before–after studies)[48, 50] | The insertion team had a lower rate per 1000 central line days: (0.5, 95% CI 0.15 to 1.4) and proportion (4/70 [6%]) of phlebitis/thrombophlebitis compared to no insertion team: rate: 4.9, 95% CI 3.3 to 7.1 and proportion (12/70 [17%]) | | | | ⨁◯◯◯ VERY LOW^a^ | Catheter insertion by insertion teams may reduce the risk of phlebitis/thrombophlebitis in neonates compared to no insertion teams, but the evidence is very uncertain. |
| **Complications related to catheter insertion** in adults, children and adolescents № of participants: (0 studies) | No evidence | | | | - |  |
| **Complications related to catheter insertion** in neonates № of participants: 731 (1 before–after study)[48] | Rate per 1000 central line days (95% CI):  Insertion team: 5.5 (4.0 to 7.7); no insertion team: 12.8 (10.1 to 16.1); rate ratio (95% CI): 0.43 (0.29 0.65) | | | | ⨁◯◯◯ VERY LOW^a^ | Catheter insertion by insertion teams may reduce the risk of complications in neonates compared to no insertion teams, but the evidence is very uncertain. |
| ***The risk in the intervention group** (and its 95% CI) is based on the assumed risk in the comparison group and the **relative effect** of the intervention (and its 95% CI). **CABSI:** catheter-associated bloodstream infections; **CI:** confidence interval; **CRBSI:** catheter-related bloodstream infection; **№:** number; **PICC:** peripherally inserted central catheter. | | | | | | |
| **GRADE Working Group grades of evidence** **High certainty:** we are very confident that the true effect lies close to that of the estimate of the effect. **Moderate certainty:** we are moderately confident in the effect estimate: the true effect is likely to be close to the estimate of the effect, but there is a possibility that it is substantially different. **Low certainty:** our confidence in the effect estimate is limited: the true effect may be substantially different from the estimate of the effect. **Very low certainty:** we have very little confidence in the effect estimate: the true effect is likely to be substantially different from the estimate of effect. | | | | | | |

**Explanations** a. We used the Effective Public Health Practice Project (EPHPP) tool to assess risk of bias in the before–after-studies. GRADE automatically started with a low certainty of evidence. We further downgraded 1 step for high risk of bias. b. Very wide CI; downgraded 2 steps for imprecision.

## Table H9: Catheter inserted in the scalp compared to catheter inserted anywhere other than the scalp in participants requiring a PICC

| Outcome № of participants (studies) | Relative effect (95% CI) | **Anticipated absolute effects (95% CI)** | | | Certainty | What happens |
| --- | --- | --- | --- | --- | --- | --- |
|  |  | **Effect with catheter inserted anywhere other than the scalp** | **Effect with catheter inserted in the scalp** | **Difference** |  |  |
| **CABSI/CRBSI** in neonates № of participants: 140 (1 cohort study)[40] | **RR 0.38** (0.02 to 6.32)^a,b,c^ | 6.6% | **0.0%** (0.0 to 0.0) | **6.6% fewer** (5.0 fewer to 10.0 more)^a,b,c^ | ⨁◯◯◯ VERY LOW^d,e^ | The evidence is very uncertain about the effect of catheter inserted in the scalp compared to catheter inserted anywhere other than the scalp on CABSI/CRBSI in neonates. |
| **Bloodstream infection–related mortality** in neonates № of participants: (0 studies) | No evidence | | | | - |  |
| **Sepsis** in neonates № of participants: 123 (1 cohort study)[37] | **RR 0.05** (0.01 to 0.35)^b,f^ | 48.8% | **2.4%** (0.5 to 17.1) | **46.3% fewer** (48.3 fewer to 31.7 fewer)^b,f^ | ⨁◯◯◯ VERY LOW^d,g^ | Catheter inserted in the scalp may reduce the risk of sepsis in neonates compared to catheter inserted anywhere other than the scalp, but the evidence is very uncertain. |
| **Local infection** in neonates № of participants: 44  (1 cohort study)[27] | **RR 0.66**  (0.13 to 3.22)^f^ | 16.0% | **10.6%**  (2.1 to 51.5) | **5.4% fewer**  13.9 fewer to 35.5 more)^f^ | ⨁◯◯◯ VERY LOW^d,e,h^ | The evidence is very uncertain about the effect of catheter inserted in the scalp compared to catheter inserted anywhere other than scalp on local infection in neonates. |
| **All-cause mortality** in neonates № of participants: 689 (1 cohort study)[30] | **RR 1.28** (0.67 to 2.47)^f^ | 10.2% | **13.0%** (6.8 to 25.1) | **2.8% more** (3.4 fewer to 14.9 more)^f^ | ⨁◯◯◯ VERY LOW^d,g,i^ | The evidence is very uncertain about the effect of catheter inserted in the scalp compared to catheter inserted anywhere other than scalp on all-cause mortality in neonates. |
| **Phlebitis/thrombophlebitis** in neonates № of participants: 2460 (4 cohort studies)[30, 36, 37, 40] | Incidence ranged from 0% to 16.1% for catheters inserted in the scalp and from 0.3% to 9.4% for catheters inserted anywhere other than the scalp.^i^ | | | | ⨁◯◯◯ VERY LOW^d,g,k^ | The evidence is very uncertain about the effect of catheter inserted in the scalp compared to catheter inserted anywhere other than the scalp phlebitis/thrombophlebitis in neonates. |
| ***The risk in the intervention group** (and its 95% CI) is based on the assumed risk in the comparison group and the **relative effect** of the intervention (and its 95% CI). **CABSI:** catheter-associated bloodstream infections; **CI:** confidence interval; **CRBSI:** catheter-related bloodstream infection; **№:** number; **PICC:** peripherally inserted central catheter; **RR:** risk ratio | | | | | | |
| **GRADE Working Group grades of evidence** **High certainty:** we are very confident that the true effect lies close to that of the estimate of the effect. **Moderate certainty:** we are moderately confident in the effect estimate: the true effect is likely to be close to the estimate of the effect, but there is a possibility that it is substantially different. **Low certainty:** our confidence in the effect estimate is limited: the true effect may be substantially different from the estimate of the effect. **Very low certainty:** we have very little confidence in the effect estimate: the true effect is likely to be substantially different from the estimate of effect. | | | | | | |

Explanations

a. Self-calculated (zero-cell correction).

b. Numbers reflect catheters.

c. Another cohort study by Callejas et al., rated as some risk of bias concerns, reported similar incidence of infectious complications (without clearly mentioning the outcome) between the groups (5/69 (7%) vs 64/620 (10%))

d. We used the Risk Of Bias In Non-randomized Studies – Of Interventions (ROBINS-I) to rate the risk of bias of the non-randomized studies. All studies have bias due to possible residual confounding and bias due to the participant selection. We downgraded 2 steps. No further downgrading was considered necessary.

e. Extremely few events; downgraded 3 steps for imprecision.

f. Self-calculated.

g Very few events; downgraded 2 steps for imprecision.

h. Composite outcome (local and systemic infections); downgraded 1 step for indirectness

i. Composite outcome (all-cause mortality or transfer to other hospital section) and mixed population (neonates and children); downgraded 1 step for indirectness

j. Numbers reflect both, participants and catheters

k. Inconsistent results across studies; downgraded 1 step for inconsistency

## Table H10: Saline flushing compared to anticoagulant flushing in participants requiring a PICC

| Outcome № of participants (studies) | Relative effect (95% CI) | **Anticipated absolute effects (95% CI)** | | | Certainty | What happens |
| --- | --- | --- | --- | --- | --- | --- |
|  |  | **Effect with heparin flushing** | **Effect with saline flushing** | **Difference** |  |  |
| **CABSI/CRBSI** in adults, children and adolescents № of participants: (0 studies) | No evidence | | | | - |  |
| **CABSI/CRBSI** in neonates № of participants: 133 (1 RCT)[51] | **RR 2.75** (0.11 to 66.21) | 0.0% | **0.0%** (0 to 0) | **0.0% fewer** (0 fewer to 0 fewer) | ⨁◯◯◯ VERY LOW^a,b^ | The evidence is very uncertain about the effect of catheter flushed with saline solution compared to catheter flushed with heparin solution on CABSI/CRBSI in neonates. |
| **Bloodstream infection**-related mortality in adults, children and adolescents, neonates № of participants: (0 studies) | No evidence | | | | - |  |
| **Sepsis** in adults, children and adolescents № of participants: (0 studies) | No evidence | | | | - |  |
| **Sepsis** in neonates № of participants: 133 (1 RCT)[51] | **RR 1.43** (0.73 to 2.82) | 17.2% | **24.6%** (12.5 to 48.5) | **7.4% more** (4.6 fewer to 31.3 more) | ⨁◯◯◯ VERY LOW^a,c^ | The evidence is very uncertain about the effect of catheter flushed with saline solution compared to catheter flushed with heparin solution on sepsis in neonates. |
| **Local infection** in adults, children and adolescents, neonates № of participants: (0 studies) | No evidence | | | | - |  |
| **All-cause mortality** in adults, children and adolescents, neonates № of participants: (0 studies) | No evidence | | | | - |  |
| **Phlebitis/thrombophlebitis** in adults № of participants: (0 studies) | No evidence | | | | - |  |
| **Phlebitis/thrombophlebitis** in children and adolescents, neonates № of participants: (0 studies) | No evidence | | | | - |  |
| ***The risk in the intervention group** (and its 95% CI) is based on the assumed risk in the comparison group and the **relative effect** of the intervention (and its 95% CI). **CABSI:** catheter-associated bloodstream infection; **CI:** confidence interval; **CRBSI:** catheter-related bloodstream infection; **№:** number; **PICC:** peripherally inserted central catheter; **RCT:** randomised controlled trial; **RR:** (adjusted) risk ratio. | | | | | | |
| **GRADE Working Group grades of evidence** **High certainty:** we are very confident that the true effect lies close to that of the estimate of the effect. **Moderate certainty:** we are moderately confident in the effect estimate: the true effect is likely to be close to the estimate of the effect, but there is a possibility that it is substantially different. **Low certainty:** our confidence in the effect estimate is limited: the true effect may be substantially different from the estimate of the effect. **Very low certainty:** we have very little confidence in the effect estimate: the true effect is likely to be substantially different from the estimate of effect. | | | | | | |

Explanations

a. Study rated as high risk of bias; downgraded 1 step for risk of bias

b. Extremely few events; downgraded 3 steps for imprecision

c. Very few events; downgraded 2 steps for imprecision

## Table H11: Catheter access using a closed-access device system compared to catheter access using an open-access device system in participants requiring a PICC

| Outcome № of participants (studies) | Relative effect (95% CI) | **Anticipated absolute effects (95% CI)** | | | Certainty | What happens |
| --- | --- | --- | --- | --- | --- | --- |
|  |  | **Effect with open-access device system** | **Effect with closed-access device system** | **Difference** |  |  |
| **CABSI/CRBSI** in adults № of participants: 793 (1 cohort study)[56] | **RR 0.11** (0.02 to 0.57)^a,b,c^ | 2.9% | **0.3%** (0.1 to 1.6) | **2.6% fewer** (2.8 fewer to 1.2 fewer)^a,b,c^ | ⨁◯◯◯ VERY LOW^d,e^ | Closed-access device systems may reduce CABSI/CRBSI in adults compared to open-access device systems, but the evidence is very uncertain. |
| **CABSI/CRBSI** in children and adolescents № of participants: (0 studies) | No evidence | | | | - |  |
| **CABSI/CRBSI** in neonates № of participants: 300 (1 cohort study)[53] | **RR 1.21** (0.83 to 1.96)^a^ | 19.3% | **23.4%** (16 to 37.9) | **4.1% more** (3.3 fewer to 18.6 more)^a^ | ⨁◯◯◯ VERY LOW^f,g^ | The evidence is very uncertain about the effect of using closed- compared to open-access device systems on CABSI/CRBSI in neonates. |
| **Bloodstream infection–related mortality** in adults, children and adolescents, neonates № of participants: (0 studies) | No evidence | | | | - |  |
| **Sepsis** in adults, children and adolescents № of participants: (0 studies) | No evidence | | | | - |  |
| **Sepsis** in neonates № of participants: 60 (1 RCT)[54] | **RR 0.13** (0.02 to 0.94) ^a,h^ | 26.7% | **3.5%** (0.5 to 25.1) | **23.2% fewer** (26.1 fewer to 1.6 fewer)^a,h^ | ⨁◯◯◯ VERY LOW^e^ | Closed-access device systems may reduce sepsis in neonates compared to open-access device systems, but the evidence is very uncertain. |
| **Local infection** in adults, children and adolescents, and neonates № of participants: (0 studies) | No evidence | | | | - |  |
| **All-cause mortality** in adults, children and adolescents, and neonates № of participants: (0 studies) | No evidence | | | | - |  |
| **Phlebitis/thrombophlebitis** in adults, children and adolescents, and neonates № of participants: (0 studies) | No evidence | | | | - |  |
| ***The risk in the intervention group** (and its 95% CI) is based on the assumed risk in the comparison group and the **relative effect** of the intervention (and its 95% CI). **CABSI:** catheter-associated bloodstream infection; **CI:** confidence interval; **CRBSI:** catheter-related bloodstream infection; **№:** number; **PICC:** peripherally inserted central catheter; **RCT:** randomized controlled trial; **RR:** risk ratio. | | | | | | |
| **GRADE Working Group grades of evidence** **High certainty:** we are very confident that the true effect lies close to that of the estimate of the effect. **Moderate certainty:** we are moderately confident in the effect estimate: the true effect is likely to be close to the estimate of the effect, but there is a possibility that it is substantially different. **Low certainty:** our confidence in the effect estimate is limited: the true effect may be substantially different from the estimate of the effect. **Very low certainty:** we have very little confidence in the effect estimate: the true effect is likely to be substantially different from the estimate of effect. | | | | | | |

**Explanations**

a. Self-calculated.

b. Numbers reflect catheters.

c. Another cohort study by Morano et al. 2015 rated as some concerns reported similar results but raw data was not reported (HR (95% CI): 0.71 (0.53 to 1.71)). The study by Yu et al. 2024 rated as some concerns reported also lower incidence of a mixed outcome (CRBSI, local infections and local inflammation) with closed systems (382*/2436 (15.7%) vs. 196*/730 (26.9%)).

d. We used the Risk Of Bias In Non-randomized Studies – Of Interventions (ROBINS-I) to rate the risk of bias of the non-randomized studies. All studies have bias due to possible residual confounding and bias due to the participant selection. We downgraded 2 steps. We further downgraded 1 step because of additional risk of bias.

e. Extremely few events; downgraded 3 steps for imprecision.

f. We used ROBINS-I to rate the risk of bias of the non-randomized studies. All studies have bias due to possible residual confounding and bias due to the participant selection. We downgraded 2 steps. No further downgrading was considered necessary.

g. Very few events; downgraded 2 steps for imprecision.

h. One low risk of bias prospective controlled trial by Reiter et al. reported a similar incidence between groups (mean 1.3/150 [0.7%] vs. 1.2/150 [0.8%]).

## Table H12: Single-lumen catheter compared to multi-lumen catheter in participants requiring a PICC

| Outcome № of participants (studies) | Relative effect (95% CI) | **Anticipated absolute effects (95% CI)** | | | Certainty | What happens |
| --- | --- | --- | --- | --- | --- | --- |
|  |  | **Effect with multi-lumen catheter** | **Effect with single-lumen catheter** | **Difference** |  |  |
| **CABSI/CRBSI** in adults № of participants: 12725 (4 cohort study) [58-60, 63] | CABSI ranged from 0%–6% in the single-lumen group and from 0%–9.9% in the multi-lumen group.^a,b,c^ | | | | ⨁◯◯◯ VERY LOW^d,e^ | Single-lumen catheters may reduce CABSI/CRBSI in adults compared to multi-lumen catheters, but the evidence is very uncertain. |
| **CABSI/CRBSI** in children and adolescents № of participants: (0 studies) | No evidence | | | | - |  |
| **CABSI/CRBSI** in neonates № of participants: 2383 (1 cohort study)[5] | Single-lumen catheters decreased the risk of CABSI/CRBSI by 61% compared to multi-lumen catheters, but the CI includes both an increase and a decrease of catheter-associated/ -related bloodstream infections (aHR [95% CI] 0.39 [0.13 to 1.16]). | | | | ⨁◯◯◯ VERY LOW^d,f^ | Single-lumen catheters may reduce CABSI/CRBSI in neonates compared to multi-lumen catheters, but the evidence is very uncertain. |
| **Bloodstream infection–related mortality** in adults, children and adolescents, neonates № of participants: (0 studies) | No evidence | | | | - |  |
| **Sepsis** in adults, children and adolescents, neonates № of participants: (0 studies) | No evidence | | | | - |  |
| **Local infection** in adults № of participants: 187 (1 cohort study)[62] | **RR 0.34** (0.14 to 0.95)^g^ | 14.7% | **5.0%** (2.1 to 13.9) | **9.7% fewer** (12.6 fewer to 0.7 fewer)^g^ | ⨁◯◯◯ VERY LOW^d,h^ | Single-lumen catheters may reduce local infections in adults compared to multi-lumen catheters, but the evidence is very uncertain. |
| **Local infection** in children and adolescents, neonates № of participants: (0 studies) | No evidence | | | | - |  |
| **All-cause mortality** in adults, children and adolescents, neonates № of participants: (0 studies) | No evidence | | | | - |  |
| **Phlebitis/thrombophlebitis** in adults, children and adolescents, neonates № of participants: (0 studies) | No evidence | | | | - |  |
| ***The risk in the intervention group** (and its 95% CI) is based on the assumed risk in the comparison group and the **relative effect** of the intervention (and its 95% CI). **CABSI:** catheter-associated bloodstream infections; **CI:** confidence interval; **CRBSI:** catheter-related bloodstream infections; **(a)HR:** (adjusted) hazard ratio; **№:** number; **PICC:** peripherally inserted central catheter; **RR:** risk ratio. | | | | | | |
| **GRADE Working Group grades of evidence** **High certainty:** we are very confident that the true effect lies close to that of the estimate of the effect. **Moderate certainty:** we are moderately confident in the effect estimate: the true effect is likely to be close to the estimate of the effect, but there is a possibility that it is substantially different. **Low certainty:** our confidence in the effect estimate is limited: the true effect may be substantially different from the estimate of the effect. **Very low certainty:** we have very little confidence in the effect estimate: the true effect is likely to be substantially different from the estimate of effect. | | | | | | |

**Explanations**

a. Another case-control study by Pongruangporn et al., rated as some risk of bias concerns, reported also higher incidence with single-lumen catheters (14 vs 28%; OR [95% CI] 2.40 [1.54 to 3.74]).

b. Another cohort study by Al Raiy et al., rated as some risk of bias concerns, reported higher incidence with single-lumen catheters but did not mention the age group of participants (0% vs 25%)

c. Another study by Khalidi et al., rated as high risk of bias, reported similar incidence between single and double lumen catheters with a positive pressure valve (0% vs 5%) and a standard cap (0% vs 0%)

d. We used the Risk Of Bias In Non-randomized Studies – Of Interventions (ROBINS-I) to rate the risk of bias of the non-randomized studies. All studies have bias due to possible residual confounding and bias due to the participant selection. We downgraded 2 steps. No further downgrading was considered necessary.

e. Combined outcome (suspected and confirmed CRBSI); downgraded 1 step for indirectness

f. Number of events and number of participants per group not reported; downgraded 1 step for imprecision

g. Self-calculated

h. Very few events overall; downgraded 2 steps for imprecision.

## Table H13: Bundle compared to routine in participants requiring a PICC

| Outcome № of participants (studies) | Relative effect (95% CI) | **Anticipated absolute effects (95% CI)** | | | Certainty | What happens |
| --- | --- | --- | --- | --- | --- | --- |
|  |  | **routine** | **bundle** | **Difference** |  |  |
| CABSI in adults № of participants: 234 (1 before-after study)[76] | **RR 0.31** (0.07 to 1.36)^a^ | 5.8% | **1.8%** (0.4 to 7.9) | **4.0% fewer** (5.4 fewer to 2.1 more)^a^ | ⨁◯◯◯ VERY LOW^b,c^ | The evidence is very uncertain about the effect of bundle interventions compared to routine care/no intervention on CABSI/CRBSI in adults. |
| CABSI in children and adolescents № of participants: (0 studies) | No evidence | | | | - |  |
| CABSI in neonates № of participants: 102 (1 cohort study)[73] | Events per 1000 central line days: bundle 0; routine care 1.73^d^ | | | | ⨁◯◯◯ VERY LOW^e,f^ | The evidence is very uncertain about the effect of bundle interventions compared to routine care/no intervention on CABSI/CRBSI in neonates. |
| Bloodstream infection–related mortality in adults, children and adolescents, neonates № of participants: (0 studies) | No evidence | | | | - |  |
| Sepsis in adults, children and adolescents № of participants: (0 studies) | No evidence | | | | - |  |
| Sepsis in neonates № of participants: 84 (1 before-after study)[67] | **RR 0.09** (0.01 to 0.64)^a^ | 40.4% | **3.6%** (0.4 to 25.8) | **36.7% fewer** (39.9 fewer to 14.5 fewer)^a^ | ⨁◯◯◯ VERY LOW^b,c^ | The evidence is very uncertain about the effect of bundle interventions compared to routine care/no intervention on sepsis in neonates. |
| Local infections in adults № of participants: 1490 (1 RCT)[71] | **RR 0.47** (0.31 to 0.72)^a,g^ | 8.4% | **4.0%** (2.6 to 6.1) | **4.5% fewer** (5.8 fewer to 2.4 fewer)^a,g^ | ⨁⨁◯◯ LOW^h,i^ | Bundle interventions may reduce local infections in adults compared to routine care/no intervention. |
| Local infections in children and adolescents № of participants: (0 studies) | No evidence | | | | - |  |
| Local infections in neonates № of participants: 102 (1 cohort study)[73] | **RR 0.50** (0.05 to 5.34) | 3.9% | **2.0%** (0.2 to 20.9) | **2.0% fewer** (3.7 fewer to 17 more) | ⨁◯◯◯ VERY LOW^e,j^ | The evidence is very uncertain about the effect of bundle interventions compared to routine care/no intervention on local infections in neonates. |
| All-cause mortality in adults, children and adolescents № of participants: (0 studies) | No evidence | | | | - |  |
| All-cause mortality in neonates № of participants: 1336 (1 before-after study)[64] | **RR 1.32** (0.63 to 2.74)^a,k^ | 2.4% | **3.1%** (1.5 to 6.5) | **0.8% more** (0.9 fewer to 4.1 more)^a,k^ | ⨁◯◯◯ VERY LOW^b,i^ | The evidence is very uncertain about the effect of bundle interventions compared to routine care/no intervention on all-cause mortality in neonates. |
| Phlebitis/thrombophlebitis in adults № of participants: 1490 (1 RCT)[71] | **RR 0.35** (0.22 to 0.56)^a,l^ | 9.0% | **3.1%** (2 to 5) | **5.8% fewer** (7 fewer to 3.9 fewer)^a,l^ | ⨁⨁◯◯ LOW^h,i^ | Bundle interventions may reduce phlebitis/thrombophlebitis in adults compared to routine care/no intervention. |
| Phlebitis/thrombophlebitis in children and adolescents № of participants: (0 studies) | No evidence | | | | - |  |
| Phlebitis/thrombophlebitis in neonates № of participants: 102 (1 non-randomised study) [73] | **RR 0.20** (0.01 to 4.06)^a^ | 3.9% | **0.8%** (0 to 15.9) | **3.1% fewer** (3.9 fewer to 12 more)^a^ | ⨁◯◯◯ VERY LOW^e,j^ | The evidence is very uncertain about the effect of bundle interventions compared to routine care/no intervention on phlebitis/thrombophlebitis in neonates. |
| Overall adverse events in adults № of participants: 100 (1 RCT)[69] | **RR 0.22** (0.05 to 0.98)^a,m^ | 18.0% | **4.0%** (0.9 to 17.6) | **14.0% fewer** (17.1 fewer to 0.4 fewer)^a,m^ | ⨁◯◯◯ VERY LOW^c,h^ | The evidence is very uncertain about the effect of bundle interventions compared to routine care/no intervention on overall adverse events in adults. |
| Overall adverse events in children and adolescents  № of participants: (0 studies) | No evidence |  |  |  |  |  |
| Overall adverse events in neonates  № of participants: 102 (1 non-randomised study)[73] | **RR 0.33** (0.12 to 0.96)^a^ | 25.5% | **8.4%**  (3.1 to 24.5) | **17.1% fewer** (22.4 fewer to 1.0 fewer)^c,e^ | ⨁◯◯◯ VERY LOW^c,e^ | The evidence is very uncertain about the effect of bundle interventions compared to routine care/no intervention on overall adverse events in neonates. |
| ***The risk in the intervention group** (and its 95% confidence interval) is based on the assumed risk in the comparison group and the **relative effect** of the intervention (and its 95% CI). **CABSI:** catheter-associated bloodstream infection; **CI:** confidence interval; **CRBSI:** catheter-related bloodstream infection; **№:** number; **PICC:** peripherally inserted central catheter; **RR:** risk ratio. | | | | | | |
| **GRADE Working Group grades of evidence** **High certainty:** we are very confident that the true effect lies close to that of the estimate of the effect. **Moderate certainty:** we are moderately confident in the effect estimate: the true effect is likely to be close to the estimate of the effect, but there is a possibility that it is substantially different. **Low certainty:** our confidence in the effect estimate is limited: the true effect may be substantially different from the estimate of the effect. **Very low certainty:** we have very little confidence in the effect estimate: the true effect is likely to be substantially different from the estimate of effect. | | | | | | |

**Explanations**

a. Self-calculated

b. We used the Effective Public Health Practice Project (EPHPP) tool to assess risk of bias in the before–after studies and ITS. GRADE starts with low certainty of evidence. We did not further downgrade for risk of bias.

c. Very few events; downgraded 2 steps for imprecision.

d. Results based on the based methodological quality study (number of patients not reported); another retrospective controlled study by Ren, et al. 2022 reported similar results (1/51 (1.96%) vs. 3/51 (5.88%)). In addition, 3 before-after studies and an interrupted time series reported similar results.

e. We used ROBINS-I to rate the risk of bias of the non-randomized studies. All studies have bias due to possible residual confounding and bias due to the participant selection. We downgraded 2 steps. We further downgraded 1 step because of additional risk of bias.

f. Number of events and number of participants not reported; downgraded 1 step for imprecision.

g. Results based on the study by Liu et al. 2013. The second RCT by He et al. 2022 reported similar results: 1/50 (2%) vs. 3/50 (6%), p< 0.05.

h. Studie(s) rated as high risk of bias; downgraded 1 step for risk of bias

i. Few events but large cohort; downgraded 1 step for imprecision

j. Extremely few events; downgraded 3 steps for imprecision

k. Results based on the larger study by Bayoumi et al. 2021; another small before-after study by Golombek et al. 2002 reported higher incidence with intervention (3/27 (11%) vs. 0/57 (0%))

l. Results based on the larger study by Liu et al. 2013; another RCT by He et al. 2022 reported similar results (0/50 (0%) vs. 1/50 (2%))

m. Another before-after study by Tian et al. 2010 reported similar results: 19/165 (11.51%) vs. 21/69 (0.43%), p= 0.0004

## Table H14: Single lumen silicon catheter compared to double-lumen polyurethan catheter in participants requiring a PICC

| Outcome № of participants (studies) | Relative effect (95% CI) | **Anticipated absolute effects (95% CI)** | | | Certainty | What happens |
| --- | --- | --- | --- | --- | --- | --- |
|  |  | **Double-lumen polyurethan catheter** | **Single lumen silicon cathere** | **Difference** |  |  |
| CABSI/CRBSI in adults, children and adolescents № of participants: (0 studies) | No evidence | | | | - |  |
| CABSI/CRBSI in neonates № of participants: 401 (1 cohort study)[65] | **RR 0.26** (0.15 to 0.45) | 27.3% | **7.1%** (4.1 to 12.3) | **20.2% fewer** (23.2 fewer to 15 fewer) | ⨁◯◯◯ VERY LOW^a,b^ | The evidence is very uncertain about the effect of single lumen silicon catheters compared to double lumen polyurethan on CABSI/CRBSI in neonates. |
| BSI-related Mortality in adults, children and adolescents, neonates № of participants: (0 studies) | No evidence | | | | - |  |
| Sepsis in adults, children and adolescents, neonates № of participants: (0 studies) | No evidence | | | | - |  |
| Local infections in adults, children and adolescents, neonates № of participants: (0 studies) | No evidence | | | | - |  |
| All-cause mortality in adults, children and adolescents, neonates № of participants: (0 studies) | No evidence | | | | - |  |
| Phlebitis/thrombophlebitis in adults, children and adolescents, neonates № of participants: (0 studies) | No evidence | | | | - |  |
| Overall adverse events in adults, children and adolescents № of participants: (0 studies) | No evidence | | | | - |  |
| Overall adverse events in neonates № of participants: 270 (1 cohort study) [72] | **RR 0.78** (0.57 to 1.05)^c^ | 45.7% | **35.6%** (26 to 48) | **10.0% fewer** (19.6 fewer to 2.3 more)^c^ | ⨁◯◯◯ VERY LOW^b,d^ | The evidence is very uncertain about the effect of single lumen silicon catheters compared to double lumen polyurethan on overall adverse events in neonates. |
| ***The risk in the intervention group** (and its 95% confidence interval) is based on the assumed risk in the comparison group and the **relative effect** of the intervention (and its 95% CI). **CABSI:** catheter-associated bloodstream infection; **CI:** confidence interval; **CRBSI:** catheter-related bloodstream infection; **№:** number; **PICC:** peripherally inserted central catheter; **RR:** risk ratio. | | | | | | |
| **GRADE Working Group grades of evidence** **High certainty:** we are very confident that the true effect lies close to that of the estimate of the effect. **Moderate certainty:** we are moderately confident in the effect estimate: the true effect is likely to be close to the estimate of the effect, but there is a possibility that it is substantially different. **Low certainty:** our confidence in the effect estimate is limited: the true effect may be substantially different from the estimate of the effect. **Very low certainty:** we have very little confidence in the effect estimate: the true effect is likely to be substantially different from the estimate of effect. | | | | | | |

**Explanations**

a. We used the Risk of Bias In Non-randomized Studies – Of Interventions (ROBINS-I) to rate the risk of bias of the non-randomized studies. All studies have bias due to possible residual confounding and bias due to the participant selection. We downgraded 2 steps. We further downgraded 1 step for high risk of bias.

b. Very few events; downgraded 2 steps for imprecision

c. Self-calculated

d. We used the Risk of Bias In Non-randomized Studies – Of Interventions (ROBINS-I) to rate the risk of bias of the non-randomized studies. All studies have bias due to possible residual confounding and bias due to the participant selection. We downgraded 2 steps. No further downgrading was considered necessary.

# Supplement I- Forest plot

Figure I1. Phlebitis/thrombophlebitis in adults


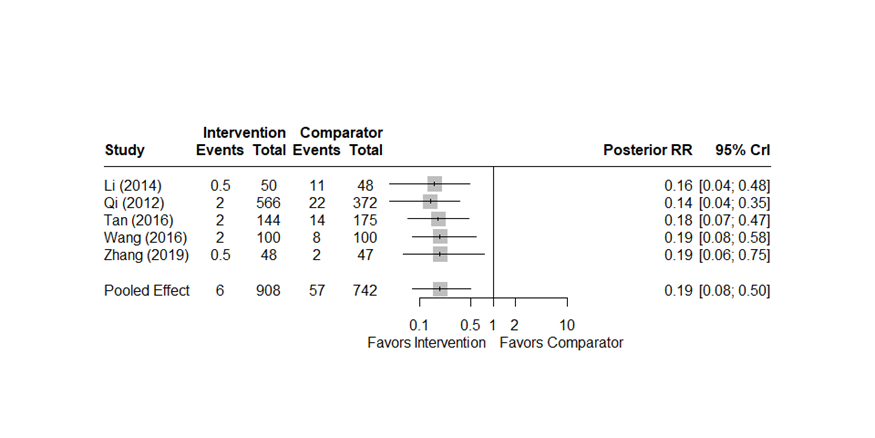


# References

1. Haynes RB, McKibbon KA, Wilczynski NL, Walter SD, Werre SR. Optimal search strategies for retrieving scientifically strong studies of treatment from Medline: analytical survey. Bmj **2005**; 330(7501): 1179.

2. Waffenschmidt S, Navarro-Ruan T, Hobson N, Hausner E, Sauerland S, Haynes RB. Development and validation of study filters for identifying controlled non-randomized studies in PubMed and Ovid MEDLINE. Res Synth Methods **2020**; 11(5): 617-26.

3. Clark JM, Sanders S, Carter M, et al. Improving the translation of search strategies using the Polyglot Search Translator: a randomized controlled trial. J Med Libr Assoc **2020**; 108(2): 195-207.

4. Garland JS, Alex CP, Uhing MR, Peterside IE, Rentz A, Harris MC. Pilot trial to compare tolerance of chlorhexidine gluconate to povidone-iodine antisepsis for central venous catheter placement in neonates. J Perinatol **2009**; 29(12): 808-13.

5. Kinoshita D, Hada S, Fujita R, Matsunaga N, Sakaki H, Ohki Y. Maximal sterile barrier precautions independently contribute to decreased central line-associated bloodstream infection in very low birth weight infants: A prospective multicenter observational study. Am J Infect Control **2019**; 47(11): 1365-9.

6. Balachander B, Rajesh D, Pinto BV, Stevens S, Rao Pn S. Simulation training to improve aseptic non-touch technique and success during intravenous cannulation-effect on hospital-acquired blood stream infection and knowledge retention after 6 months: The snowball effect theory. J **2021**; 22(3): 353-8.

7. Bozaan D, Skicki D, Brancaccio A, et al. Less Lumens-Less Risk: A Pilot Intervention to Increase the Use of Single-Lumen Peripherally Inserted Central Catheters. J Hosp Med **2019**; 14(1): 42-6.

8. Kun C, Yan J, Suwen X, et al. Effect of specialty training on nursing staff’s KAP on PICC and catheter maintenance. Biomedical Research (India) **2017**; 28(20): 9144-7.

9. Purran A, Weller G, Kerr C. Evaluation of a PICC care training programme. Nurs Stand **2016**; 30(20): 45-50.

10. Sakai H, Hirosue M, Iwata M, Terasawa T. The effect of introducing a nurse-practitioner-led peripherally inserted central venous catheter placement program on the utilization of central venous access device: A retrospective study in Japan. J **2023**.

11. Walters B, Price C. Quality Improvement Initiative Reduces the Occurrence of Complications in Peripherally Inserted Central Catheters. J Infus Nurs **2019**; 42(1): 29-36.

12. Zhang J, Tang S, He L, et al. [Effect of standardized PICC training and management on the clinical effect and complication of catheterization]. Zhong Nan Da Xue Xue Bao Yi Xue Ban **2014**; 39(6): 638-43.

13. Kaufman DA, Blackman A, Conaway MR, Sinkin RA. Nonsterile glove use in addition to hand hygiene to prevent late-onset infection in preterm infants: randomized clinical trial. JAMA Pediatr **2014**; 168(10): 909-16.

14. Barber JM, Booth DM, King JA, Chakraverty S. A nurse led peripherally inserted central catheter line insertion service is effective with radiological support. Clin Radiol **2002**; 57(5): 352-4.

15. Chen MH, Hwang WL, Chang KH, Chiang LCJ, Teng CLJ. Application of peripherally inserted central catheter in acute myeloid leukaemia patients undergoing induction chemotherapy. Eur J Cancer Care (Engl) **2017**; 26(6).

16. Gong P, Huang XE, Chen CY, Liu JH, Meng AF, Feng JF. Comparison of complications of peripherally inserted central catheters with ultrasound guidance or conventional methods in cancer patients. Asian Pac J Cancer Prev **2012**; 13(5): 1873-5.

17. Li J, Fan YY, Xin MZ, et al. A randomised, controlled trial comparing the long-term effects of peripherally inserted central catheter placement in chemotherapy patients using B-mode ultrasound with modified Seldinger technique versus blind puncture. Eur J Oncol Nurs **2014**; 18(1): 94-103.

18. Qi YZ, Guo Y, Xu XX, Zhang H, Li L. Comparison of placement of peripherally inserted central catheters using vascular ultrasound guidance system and traditional method in 938 tumor patients. Chinese Journal of Clinical Nutrition **2012**; 20(4): 253-5.

19. Tan J, Liu L, Xie J, Hu L, Yang Q, Wang H. Cost-effectiveness analysis of ultrasound-guided Seldinger peripherally inserted central catheters (PICC). Springerplus **2016**; 5(1): 2051.

20. Tang H, Xiang QF, Yu CH, Fu Y, Li JY. Vascular ultrasound combined with Seldinger technology improves the success rate of peripherally inserted central catheter and reduces potential complications. Chinese Journal of Clinical Nutrition **2012**; 20(3): 178-81.

21. Wang Q, Wang N, Sun Y. Clinical effect of peripherally inserted central catheters based on modified seldinger technique under guidance of vascular ultrasound. Pak J Med Sci **2016**; 32(5): 1179-83.

22. Yin T, Huo Y, Zhao Y, Li W, Gao H. Retrospective Study of the Application Value Analysis of Ultrasound-Guided Technology in Peripheral Deep Venous Catheterization of Neonates. Dis Markers **2022**; 2022: 1726906.

23. Yuan L, Fu R, Li R-M, Guo M-M, Chen X-Y. The Effects of 3 Peripherally Inserted Central Catheter Insertion Techniques in Chinese Patients With Different Vascular Status. Journal of the Association for Vascular Access **2013**; 18(4): 225-30.

24. Zhang L, Zhang X, Kang Y, Teng Y, Yang Y. Application of PICC Catheterization with Modified Seldinger Technique Under the Guidance of B-mode Ultrasound in Chemotherapy Patients. Anti-Tumor Pharmacy **2019**; 9(5): 779-82.

25. Pongruangporn M, Ajenjo MC, Russo AJ, et al. Patient- and device-specific risk factors for peripherally inserted central venous catheter-related bloodstream infections. Infect Control Hosp Epidemiol **2013**; 34(2): 184-9.

26. Razavinejad SM, Saeed N, Pourarian S, et al. Complications and Related Risk Factors of Peripherally Inserted Central Catheters in Neonates: A Historical Cohort Study. Archives of Iranian Medicine **2023**; 26(4): 218-25.

27. Aggarwal R, Downe L. Use of percutaneous silastic central venous catheters in the management of newborn infants. Indian Pediatr **2001**; 38(8): 889-92.

28. Bashir RA, Swarnam K, Vayalthrikkovil S, Yee W, Soraisham AS. Association between Peripherally Inserted Central Venous Catheter Insertion Site and Complication Rates in Preterm Infants. Am J Perinatol **2016**; 33(10): 945-50.

29. Bulbul A, Okan F, Nuhoglu A. Percutaneously inserted central catheters in the newborns: a center's experience in Turkey. J Matern Fetal Neonatal Med **2010**; 23(6): 529-35.

30. Callejas A, Osiovich H, Ting JY. Use of peripherally inserted central catheters (PICC) via scalp veins in neonates. J Matern Fetal Neonatal Med **2016**; 29(21): 3434-8.

31. Ekaputri DS, Sukmawati M, Sidiartha IGL, Nilawati GAP, Utama IMGDL, Gustawan IW. Peripherally Inserted Central Catheter Dwell Time as a Risk Factor of Central Line-Associated Bloodstream Infection in Neonates. Iranian Journal of Neonatology **2022**; 13(2): 39-45.

32. Elmekkawi A, Maulidi H, Mak W, Aziz A, Lee KS. Outcomes of upper extremity versus lower extremity placed peripherally inserted central catheters in a medical-surgical neonatal intensive care unit1. J Neonatal Perinatal Med **2019**; 12(1): 57-63.

33. Gai M, Wang Y, Chen J, et al. Effect of femoral PICC line insertion in neonates with digestive tract disease. Am J Transl Res **2022**; 14(10): 7487-93.

34. Hoang V, Sills J, Chandler M, Busalani E, Clifton-Koeppel R, Modanlou HD. Percutaneously inserted central catheter for total parenteral nutrition in neonates: complications rates related to upper versus lower extremity insertion. Pediatrics **2008**; 121(5): e1152-9.

35. Hu Y, Ling Y, Ye Y, et al. Analysis of risk factors of PICC-related bloodstream infection in newborns: implications for nursing care. Eur J Med Res **2021**; 26(1): 80.

36. Kisa P, Ting J, Callejas A, Osiovich H, Butterworth SA. Major thrombotic complications with lower limb PICCs in surgical neonates. J Pediatr Surg **2015**; 50(5): 786-9.

37. López Sastre JB, Fernández Colomer B, Coto Cotallo GD, Ramos Aparicio A. Prospective evolution of percutaneous central venous silastic catherters in newborn infants. "castrillo" hospital group. An Esp Pediatr **2000**; 53(2): 138-47.

38. Ma M, Garingo A, Jensen AR, Bliss D, Friedlich P. Complication risks associated with lower versus upper extremity peripherally inserted central venous catheters in neonates with gastroschisis. J Pediatr Surg **2015**; 50(4): 556-8.

39. Malinoski D, Ewing T, Bhakta A, et al. Which central venous catheters have the highest rate of catheter-associated deep venous thrombosis: a prospective analysis of 2,128 catheter days in the surgical intensive care unit. J Trauma Acute Care Surg **2013**; 74(2): 454-60; discussion 61-2.

40. Padilla-Sanchez C, Montejano-Lozoya R, Benavent-Taengua L, et al. Risk factors associated with adverse events in neonates with peripherally inserted central catheter. Enferm Intensiva (Engl Ed) **2019**; 30(4): 170-80.

41. Pet GC, Eickhoff JC, McNevin KE, Do J, McAdams RM. Risk factors for peripherally inserted central catheter complications in neonates. J Perinatol **2020**; 40(4): 581-8.

42. Tsai MH, Lien R, Wang JW, et al. Complication rates with central venous catheters inserted at femoral and non-femoral sites in very low birth weight infants. Pediatr Infect Dis J **2009**; 28(11): 966-70.

43. Wrightson DD. Peripherally inserted central catheter complications in neonates with upper versus lower extremity insertion sites. Adv Neonatal Care **2013**; 13(3): 198-204.

44. Linder LE, Curelaru I, Gustavsson B, Hansson HA, Stenqvist O, Wojciechowski J. Material thrombogenicity in central venous catheterization: a comparison between soft, antebrachial catheters of silicone elastomer and polyurethane. JPEN J Parenter Enteral Nutr **1984**; 8(4): 399-406.

45. Gomes de Souza NM, Silveira Rocha R, Pinheiro Ferreira R, Bastos da Silveira Reis C, Souza Bandeira RS, Facanha Melo AP. Comparing the use of silicone and polyurethane Peripherally Inserted Central Catheters in newborns: A retrospective study. J Clin Nurs **2021**; 30(23-24): 3439-47.

46. Ong CK, Venkatesh SK, Lau GB, Wang SC. Prospective randomized comparative evaluation of proximal valve polyurethane and distal valve silicone peripherally inserted central catheters. J Vasc Interv Radiol **2010**; 21(8): 1191-6.

47. Chico-Padron RM, Carrion-Garcia L, Delle-Vedove-Rosales L, et al. Comparative safety and costs of transparent versus gauze wound dressings in intravenous catheterization. J Nurs Care Qual **2011**; 26(4): 371-6.

48. Levit O, Shabanova V, Bizzarro M. Impact of a dedicated nursing team on central line-related complications in neonatal intensive care unit. J Matern Fetal Neonatal Med **2020**; 33(15): 2618-22.

49. Pitts S. Retrospective Analysis of a Pediatric Vascular Access Program and Clinical Outcomes. Journal of the Association for Vascular Access **2013**; 18(2): 114-20.

50. Yongshu LIU, Ting S, Lan S, et al. Evaluation of PICC team in prevention of peripherally inserted central catheter-related mechanical phlebitis among very-low-birth-weight premature infants. Nursing of Integrated Traditional Chinese & Western Medicine **2019**; 5(6): 5-8.

51. Araujo OR, Araujo MC, Silva JS, Barros MM. Intermittent heparin is not effective at preventing the occlusion of peripherally inserted central venous catheters in preterm and term neonates. Rev **2011**; 23(3): 335-40.

52. Morano SG, Latagliata R, Girmenia C, et al. Catheter-associated bloodstream infections and thrombotic risk in hematologic patients with peripherally inserted central catheters (PICC). Support Care Cancer **2015**; 23(11): 3289-95.

53. Reiter PD, Novak K, Valuck RJ, Rosenberg AA, Fish D. Effect of a closed drug-delivery system on the incidence of nosocomial and catheter-related bloodstream infections in infants. Epidemiol Infect **2006**; 134(2): 285-91.

54. Rundjan L, Rohsiswatmo R, Paramita TN, Oeswadi CA. Closed catheter access system implementation in reducing the bloodstream infection rate in low birth weight preterm infants. Front **2015**; 3: 20.

55. Yu K, Li J, Zhang D, et al. Types of peripherally inserted central catheters and the risk of unplanned removals in patients: A prospective cohort study. Int J Nurs Stud **2024**; 149: 104621.

56. Zerla PA, Canelli A, Caravella G, et al. Open- vs Closed-Tip Valved Peripherally Inserted Central Catheters and Midlines: Findings from a Vascular Access Database. Journal of the Association for Vascular Access **2015**; 20(3): 169-76.

57. Al Raiy B, Fakih MG, Bryan-Nomides N, et al. Peripherally inserted central venous catheters in the acute care setting: A safe alternative to high-risk short-term central venous catheters. Am J Infect Control **2010**; 38(2): 149-53.

58. Bae HS, Kim KY, Han YM. Comparison of complications between reverse-tapered and nontapered peripherally inserted central catheters. PLoS ONE **2023**; 18(5): e0285445.

59. Barrigah-Benissan K, Ory J, Simon C, et al. Clinical factors associated with peripherally inserted central catheters (PICC) related bloodstream infections: a single centre retrospective cohort. Antimicrob **2023**; 12(1): 5.

60. Chopra V, Ratz D, Kuhn L, Lopus T, Chenoweth C, Krein S. PICC-associated bloodstream infections: prevalence, patterns, and predictors. Am J Med **2014**; 127(4): 319-28.

61. Khalidi N, Kovacevich DS, Papke-O'Donnell LF, Btaiche I. Impact of the positive pressure valve on vascular access device occlusions and bloodstream infections. JAVA - Journal of the Association for Vascular Access **2009**; 14(2): 84-91.

62. Liscynesky C, Johnston J, Haydocy KE, Stevenson KB. Prospective evaluation of peripherally inserted central catheter complications in both inpatient and outpatient settings. Am J Infect Control **2017**; 45(9): 1046-9.

63. Rejane Rabelo-Silva E, Lourenco SA, Maestri RN, et al. Patterns, appropriateness and outcomes of peripherally inserted central catheter use in Brazil: a multicentre study of 12 725 catheters. BMJ Qual Saf **2022**; 31(9): 652-61.

64. Bayoumi MAA, Van Rens MFP, Chandra P, et al. Effect of implementing an Epicutaneo-Caval Catheter team in Neonatal Intensive Care Unit. J **2021**; 22(2): 243-53.

65. Costa P, Paiva ED, Kimura AF, Castro TEd. Fatores de risco para infecção de corrente sanguínea associada ao cateter central de inserção periférica em neonatos. Acta Paulista de Enfermagem **2016**; 29(2): 161-8.

66. Eturajulu RC, Ng KH, Tan MP, et al. Quality Improvement Report: Safety Program for Prevention of Central Line-associated Bloodstream Infections. Radiographics **2022**; 42(7): E216-E23.

67. Golombek SG, Rohan AJ, Parvez B, Salice AL, LaGamma EF. "Proactive" management of percutaneously inserted central catheters results in decreased incidence of infection in the ELBW population. J Perinatol **2002**; 22(3): 209-13.

68. Harnage SA. Achieving Zero Catheter Related Blood Stream Infections: 15 Months Success in a Community Based Medical Center. Journal of the Association for Vascular Access **2007**; 12(4): 218-24.

69. He B, Zhang A, He S. Therapeutic Effect of Ultrasound-Guided Peripherally Inserted Central Catheter Combined with Predictive Nursing in Patients with Large-Area Severe Burns. Comput Math Methods Med **2022**; 2022: 1019829.

70. Kaplan HC, Lannon C, Walsh MC, Donovan EF, Ohio Perinatal Quality C. Ohio statewide quality-improvement collaborative to reduce late-onset sepsis in preterm infants. Pediatrics **2011**; 127(3): 427-35.

71. Liu XY, Shen YY, Xu XH, Tang XH. Application of evidence-based nursing in the prevention of postoperative complications with PICC insertion. Chinese Journal of Clinical Nutrition **2013**; 21(5): 309-12.

72. Paiva ED, Kimura AF, Costa P, Magalhães TEdC, Toma E, Alves AMA. Complications related to the type of epicutaneous catheter in a cohort of neonates. Online Brazilian Journal of Nursing **2013**; 12(4): 942-52.

73. Ren C, Dong F, Du Y, Wang J. Implications of PDCA management for central venous line placement in neonates. Revista de Psiquiatria Clinica **2022**; 49(6): 124-7.

74. Royer T. Implementing a better bundle to achieve and sustain a zero central line-associated bloodstream infection rate. J Infus Nurs **2010**; 33(6): 398-406.

75. Steiner M, Langgartner M, Cardona F, et al. Significant Reduction of Catheter-associated Blood Stream Infections in Preterm Neonates After Implementation of a Care Bundle Focusing on Simulation Training of Central Line Insertion. Pediatr Infect Dis J **2015**; 34(11): 1193-6.

76. Tian G, Zhu Y, Qi L, Guo F, Xu H. Efficacy of multifaceted interventions in reducing complications of peripherally inserted central catheter in adult oncology patients. Support Care Cancer **2010**; 18(10): 1293-8.

77. Tong L, Zhong Y, Feng BL, Yu ZY. Preventing peripheral central venous catheter-related bloodstream infections through process management. Chinese Journal of Clinical Nutrition **2011**; 19(1): 56-8.
